# Epigenetics and adaptive phenotypic variation between habitats in an asexual snail

**DOI:** 10.1038/s41598-017-14673-6

**Published:** 2017-10-26

**Authors:** Jennifer L.M. Thorson, Mark Smithson, Daniel Beck, Ingrid Sadler-Riggleman, Eric Nilsson, Mark Dybdahl, Michael K. Skinner

**Affiliations:** Center for Reproductive Biology School of Biological Sciences Washington State University, Pullman, WA-99164-4236 USA

## Abstract

In neo-Darwinian theory, adaptation results from a response to selection on relatively slowly accumulating genetic variation. However, more rapid adaptive responses are possible if selectable or plastic phenotypic variation is produced by epigenetic differences in gene expression. This rapid path to adaptation may prove particularly important when genetic variation is lacking, such as in small, bottlenecked, or asexual populations. To examine the potential for an epigenetic contribution to adaptive variation, we examined morphological divergence and epigenetic variation in genetically impoverished asexual populations of a freshwater snail, *Potamopyrgus antipodarum*, from distinct habitats (two lakes versus two rivers). These populations exhibit habitat specific differences in shell shape, and these differences are consistent with adaptation to water current speed. Between these same habitats, we also found significant genome wide DNA methylation differences. The differences between habitats were an order of magnitude greater than the differences between replicate sites of the same habitat. These observations suggest one possible mechanism for the expression of adaptive shell shape differences between habitats involves environmentally induced epigenetic differences. This provides a potential explanation for the capacity of this asexual snail to spread by adaptive evolution or plasticity to different environments.

## Introduction

Genetic variation is classically considered a pre-requisite to heritable phenotypic variation, the raw material of adaptation by natural selection^1–4^. However, adaptive responses in some traits are too rapid to be explained by selection through rare genetic mutations, which require long periods of time to accumulate^5,6^. Alternatively, epigenetic alterations in the absence of genetic change can affect gene expression^7^. These epigenetic changes can result in regulation of genome activity independent of DNA sequence and are mitotically stable^8^. If epigenetic signals producing phenotypic variation are inherited, they can form the basis of adaptive evolutionary change^9,10^. Furthermore, if epigenetic changes are environmentally induced and adaptively biased, they can form the basis of adaptive phenotypic plasticity^11,12^. In either case, epigenetic changes that are independent of genetic sequences represent a potential mechanism for rapid adaptive responses to heterogeneous conditions.

Rapid adaptive responses based on epigenetics are possible because the rate of epimutations from DNA methylation has been shown to be orders of magnitude higher than the rate of genetic mutations^13,14^. Theoretical investigations have suggested that populations may respond to the environment through epigenetic variation before genetic mutations begin to accumulate^15^. Epigenetic variation such as DNA methylation is found in most species^16^ and variation is common among populations^17–19^. Some studies show how epigenetic variation is associated with phenotypic variation, for example in plants^20,21^ and more recently in animals^22,23^. Additionally, recent evidence has shown that some epigenetic marks can be inherited across generations^24–26^. Environmentally induced DNA methylation that produces adaptively biased phenotypic variation can provide the basis of an adaptive plastic response^27^.

Asexual organisms provide a unique opportunity to investigate the importance of epigenetic variation as a mechanism generating phenotypic and adaptive variation^27,28^. In the absence of genetic diversity in asexuals, the contribution of epigenetic diversity to the generation of a range of different phenotypes may provide an alternative pathway to adaptation by natural selection or plasticity^29^. For epigenetic variation to play a role in adaptation to different habitats in genetically depauperate asexuals, it would be important to demonstrate adaptive phenotypic differences by habitat, epigenetic differences by habitat, that these habitat-based differences are not random, and ultimately, a demonstration that the epigenetic differences directly lead to the phenotype differences. Here, we focus on epigenetic and phenotypic differences between habitats in an asexual snail.

We studied populations of the asexual New Zealand fresh water snail (*Potamopyrgus antipodarum*), an invasive species where a single clonal lineage exhibits divergence in shell shape fewer than 30 years (approximately 100 generations) after establishment in the western USA. Earlier studies showed that shell shape varies among river sites, with a larger aperture for a given length in faster water currents^30^. Several lines of evidence indicate that this variation is adaptive. First, the adaptive value of this shell shape response is supported by biomechanical considerations in fluid flow^31^. A larger shell aperture permits a larger relative foot muscle, providing more foot surface contact area to grip the substrate in faster current speeds. The better grip likely increases fitness by facilitating foraging in fast flow habitats. Snail foot muscle size is well known to correlate with the aperture height of the shell^32–34^. Second, these shell shape responses concord with adaptive differences observed in other snails^34–36^. Finally, the same pattern of shell shape variation across current speed regimes was observed in a native congener, which has a long evolutionary history in the same geographic region^37^.

We were interested in the source of variation for this adaptive shell shape divergence for two reasons. First, populations across the western US range comprise parthenogenetic females that express the same multilocus genotype (assayed by mtDNA, msDNA, and allozyme loci), suggesting limited genetic diversity from a single clonal founder^38^. Second, adaptive divergence in shell shape does not appear to be the result of within-generation plasticity because phenotypic adjustment to a common lab environment takes more than two generations (M Smithson, M Skinner, and M Dybdahl, *personal communication*). One possible explanation for adaptive phenotypic variation among habitats is variation in DNA methylation that mediates adaptive phenotypic plasticity or responds to natural selection.

## Results

**Figure 1:**
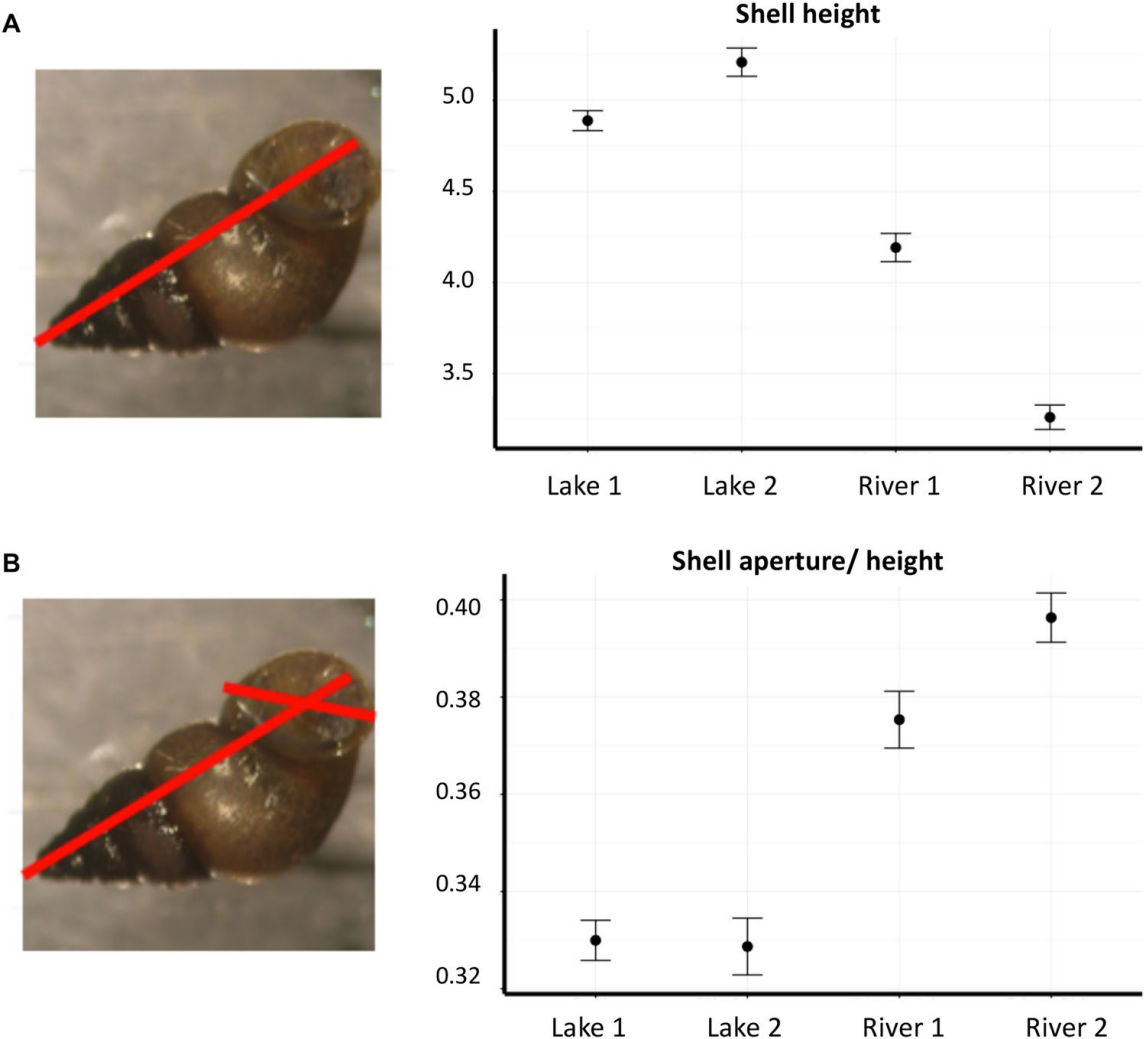
Shell shape phenotypic variation. Shell shape divergence in lake and river populations of the asexual snail *Potamopyrgus antipodarum*. Shell shape measurements are indicated by red lines in the inset picture. **(A)** Shell height is the total length from apex to tip of aperture. **(B)** Shell aperture/shell length measures the size of the shell opening relative to shell height. Statistical significance based on Student’s t-test; error bars represent one standard error.

To examine morphological divergence and methylation differences associated with different current speed habitats, we sampled snails from two rivers representing fast current speed habitat (River 1 site: main stem Snake River, ID at Ritter Island; River 2 site: a nearby tributary spring stream), and two lakes with no current (Lake 1 site: Lake Lytle, Rockaway Beach, OR; Lake 2 site: Lake Washington, Seattle, WA). River sites were about 5 km apart, lake sites were about 240 km apart, and lake and river sites were approximately 800 km apart. These sites were chosen to maximize current speed differences (lakes vs. rivers), even though this meant larger distances between habitats because the snails do not occupy both lakes and rivers in the same areas. We sampled at least 30 individuals from each site and measured shell spire height and aperture length as an index of shell shape differences and foot muscle size. In concordance with earlier results, we found that the snails exhibited the expected adaptive pattern in shell shape and that these differences were statistically significant (Table 1); river snails had a significantly smaller overall spire height than the lake snails (Fig. 1A), and a significantly larger aperture opening for a given spire height (Fig. 1B).

**Table 1:** Analysis of Variance for phenotypic traits.

Source of Variation	df	Sum of Squares	Mean Square	F value
ANOVA for shell height				
Habitat	1	78.78	78.78	867.61, p < 0.0001
Sample Site	2	18.93	9.46	104.22, p < 0.0001
DNA Pool	8	0.64	0.08	0.88, p = 0.628
error	138	12.53	0.09	
**ANOVA for shell aperture/shell height**				
Habitat	1	0.130	0.130	251.32, p < 0.0001
Sample Site	2	0.008	0.004	8.56, p = 0.0018
DNA Pool	8	0.004	0.001	0.874, p = 0.5838
error	138	0.001	0.001	

Shell height is the maximum length of the spire of the shell. Aperture/shell height is the maximum width of the aperture divided by the maximum height. There is a significant difference in shell shape for both of these traits between habitats. In the model, we account for variation between lake and river habitats (Habitat), between sites within habitat (Sample site), and among the pools within site (DNA pool).

To investigate the epigenetic response to the different habitats, we examined the differential levels of DNA methylation (DMR) using foot muscle tissue for each of the four sites. We chose to sample the foot muscle tissue for two reasons. First, foot muscle is easily accessible having a homogeneous histology such that a more purified cell population can be obtained (Supplemental Fig. S1) from the adult^39^. Second, we expect foot muscle size and shell aperture size to be correlated traits adapted to water current speed. To obtain foot muscle DNA, we dissected foot tissue from each individual after phenotypic measurements were taken, and extracted DNA from this tissue. Samples of 10 or 20 individuals were pooled and sonicated, and the DNA from 3 pools were prepared for each of the four populations for analysis.

**Figure 2:**
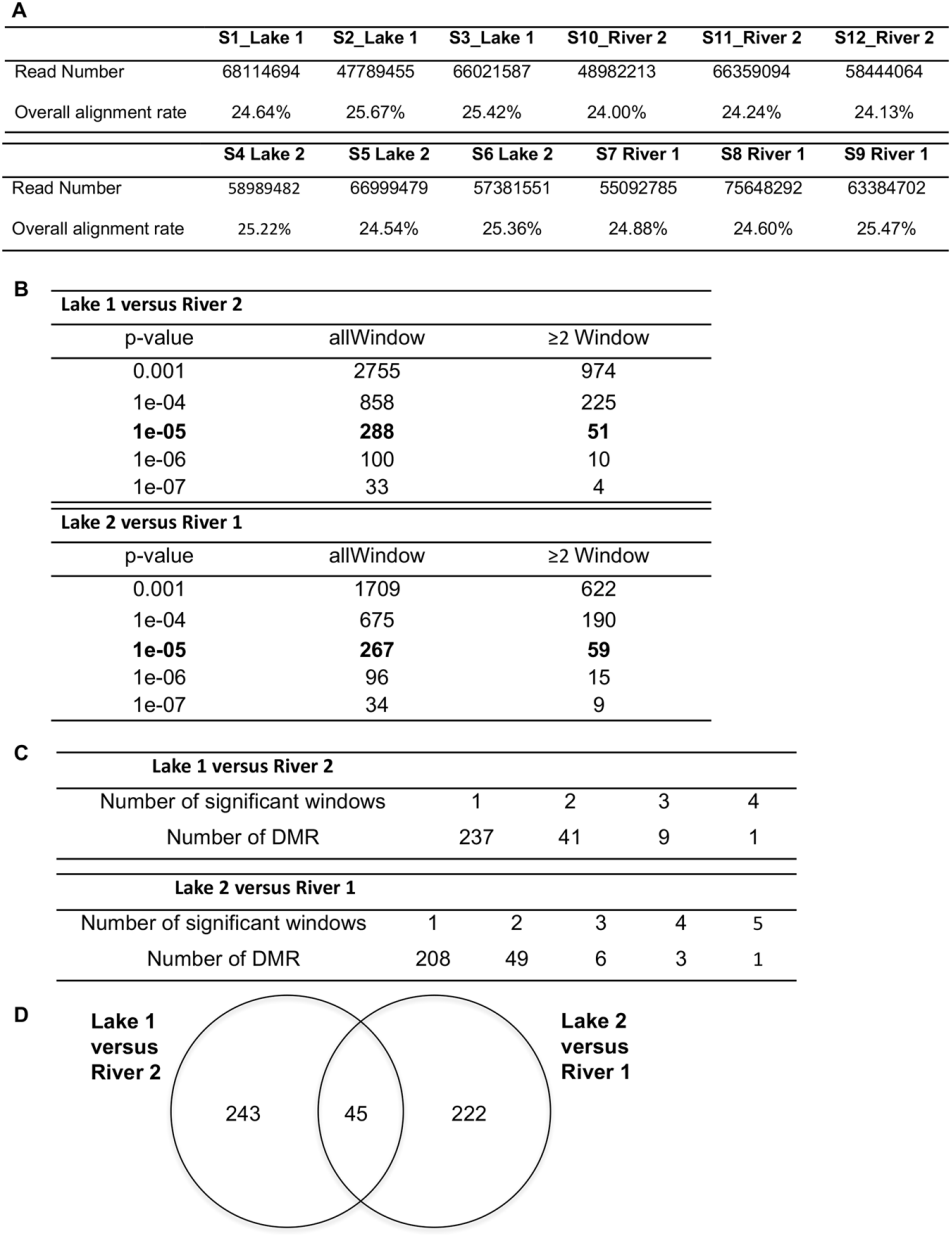
Epigenetic variation in DNA methylation. **(A)** The number of reads present for each sample and the overall alignment rate calculated by bowtie2. **(B)** The number of DMRs found using different p-value cutoff thresholds. The allWindow column shows all DMRs. The ≥2 Window column shows the number of DMRs containing at least two significant windows. **(C)** The number of DMR with each specific number of significant windows at a p-value threshold of 1e-05. **(D)** Venn diagram identifying the overlap for the DMRs between the two different lake versus river comparisons.

To determine the magnitude of differences in methylated DNA associated with different habitats and phenotypes, we analyzed differentially DNA methylated regions (DMR) between habitats and sites. DMRs are the windows that are differential methylated in all three pools from one population set compared to another (e.g. lake vs river). First, methylated DNA was isolated for each pool using methylated DNA immunoprecipitation (MeDIP) of the genomic DNA. MeDIP DNA pools were used to create libraries which were sequenced for a MeDIP-Seq procedure previously described^39^. The pools were sequenced on an Illumina HiSeq 2500 sequencing platform which yielded approximately 60 million reads per pool for comparison of the three pools each for lake and river sites (Fig. 2A). The reference genome used an expression cDNA library previously described^40^. DMRs were identified at a p-value threshold of p < 10^−5^ for single 100 bp sites and multiple adjacent ≥2 100 bp sites (Fig. 2B). The majority of DMRs were single windows with some having 2 or 3 multiple windows (Fig. 2C). An overlap of the two habitat comparisons (Lake 2 versus River 1 and Lake 1 versus River 2) demonstrated 153 overlapping DMRs which is approximately one third of the DMR detected, Fig. 2D.

**Figure 3:**
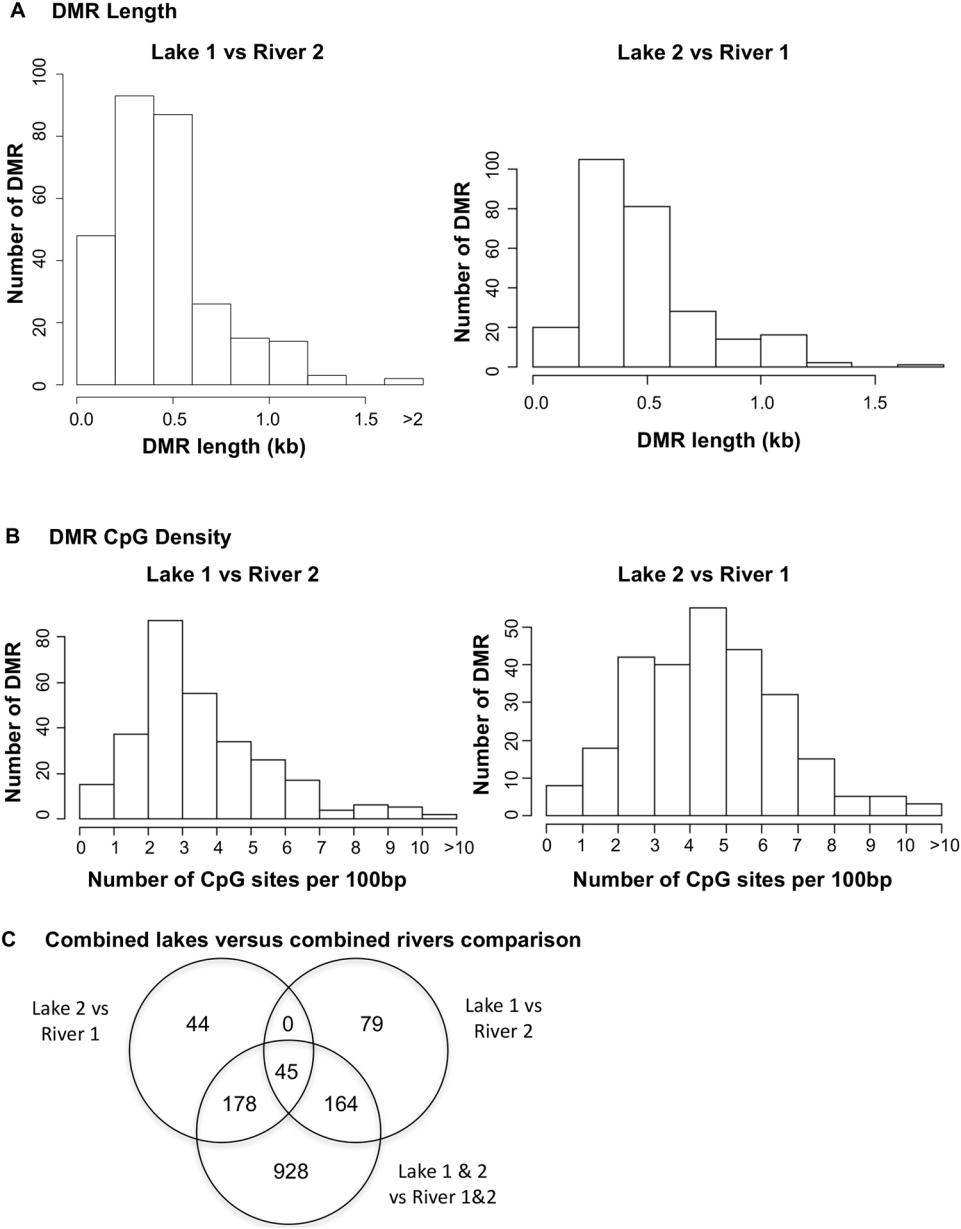
DMR genomic features. **(A)** Number of DMR versus the DMR lengths (kb). All DMRs at a p-value threshold of 1e-05 are shown. **(B)** Number of DMR versus the number of DMRs at different CpG densities. All DMRs at a p-value threshold of 1e-05 are shown. **(C)** DMR numbers (p < 1e-05) and overlap with combined lakes (1 & 2) versus combined rivers (1 & 2) comparison with the individual lake versus lake comparisons.

All DMRs identified using a p-value threshold of p < 10^−5^ were used for all windows for subsequent analysis. A mixture of DMRs with an increase or decrease in methylation involving a ratio of river/lake (% read depth for lake) are observed (data not shown). Further analysis of the genomic features of the DMRs demonstrated lengths of 200–1000 bp (Fig. 3A). The most predominant lengths for both comparisons was 400–600 bp (Fig. 3A). The DMR were located in CpG deserts with <10 CpG/100 bp and 1–5 CpG/100 bp being predominant (Fig. 3B). Therefore, the genomic features were similar to previous DMRs identified^41^.

To determine whether methylation differences were habitat specific or random, we compared DMRs between sites and habitats. As a measure of random epigenetic variation, we determined the number of DMRs between sites within a habitat (river vs river and lake vs lake). As a measure of habitat specific epigenetic variation, we determined the number of DMRs between habitats for each combination of sites (Table 2). The number of DMRs that are habitat specific and associated with phenotypic differences between habitats should be greater than the number of DMRs between sites within the same habitat. Results show that the number of DMRs between lake and river habitats was an order of magnitude larger than the number of DMRs between sites within a habitat type (Table 2).

**Table 2:** Significant DMRs identified in comparisons of lake and river sites.

Comparison		1 Window DMR	≥2 Window DMR
Between sites within habitat	Lake 1 v. Lake 2	21	6
	River 2 v. River 1	8	4
Between habitats for each combination	Lake 1 v. River 1	138	26
	Lake 1 v. River 2	288	51
	Lake 2 v. River 1	253	57
	Lake 2 v. River 2	292	63

The p-value thresholds for all analyses were 1e-05. A large majority of DMRs appear to be habitat specific and associated with adaptive phenotypic differences.

An additional comparison used the combined Lake 1 and 2 six pools versus the combined River 1 and 2 six pools to determine the DMRs and compare to the Lake 1 versus River 2 and Lake 2 versus River 1 comparisons. The overlap and comparisons are presented in Fig. 3C. The number of DMRs for combined comparison were higher, in part due to the increased read depth and sample number. The same DMR genomic features were also observed. The overlap demonstrated a reasonable DMR overlap between all the comparisons with the same 45 overlapping DMR as the two individual comparisons. Overall the same phenomenon was observed in having a significant epigenetic alteration in the comparisons of the combined lakes and rivers.

**Figure 4:**
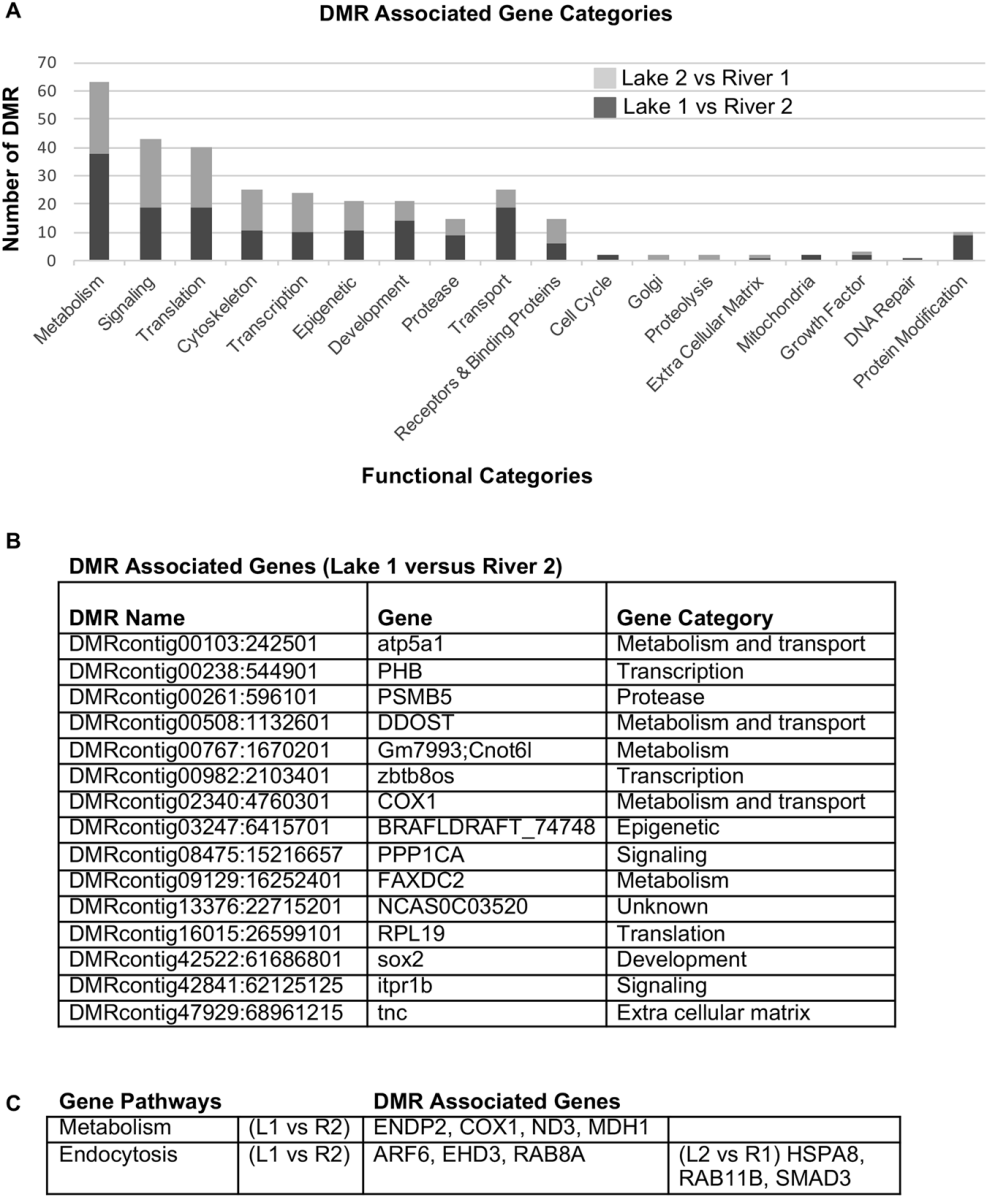
DMR associated genes. **(A)** Number of DMR associated genes correlated to specific gene categories using DMR at p < 10^−3^ threshold. **(B)** List of DMR associated genes using DMR p < 10^–5^ and **(c)** Gene pathways with a minimum number of three associated genes listed. The Lake 1 versus River 2 (L1 vs R2) and Lake 2 versus River 1 (L2 vs R1) indicated.

**Figure 5:**
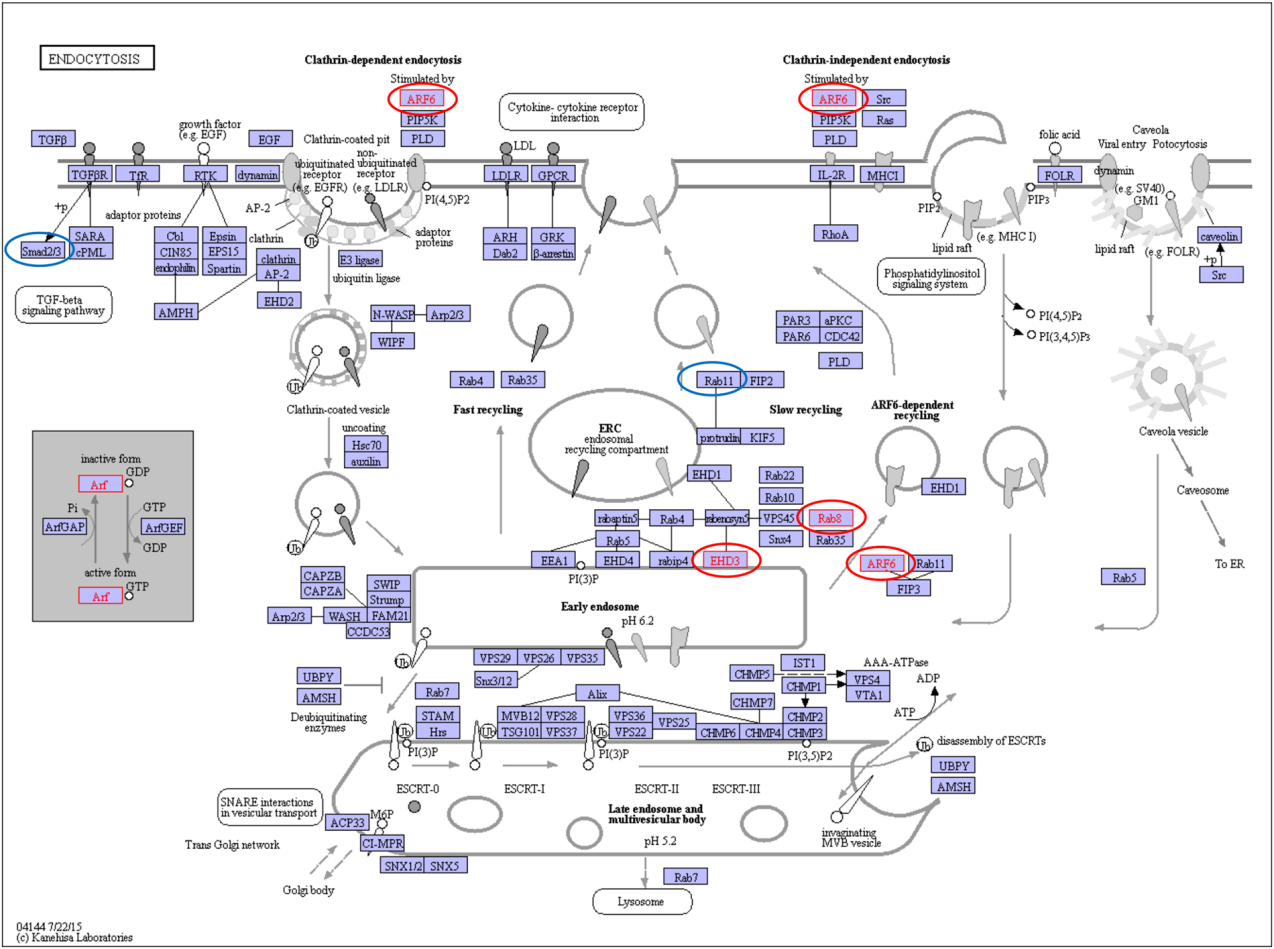
The Endocytosis pathway. The endocytosis pathway from KEGG is provided that identifies (red circle) Lake 1 versus River 2 and (blue circle) Lake 2 versus River 1 indicates the DMR associated genes within the pathway^59,60^.

To determine DMR associated genes the reference sequence at the DMR location was analyzed with a Blast batch search against the NCBI nt database as previously described^14^. Blast hits were altered to include only those with linked genes. From the Lake 1 versus River 2 2770 DMRs at p ≤ 10^−3^ there were 191 DMR associated genes that are listed in Supplemental Table S1, and at p ≤ 10^−5^ there were 15 DMR associated genes that are listed in Fig. 4B. From the Lake 2 versus River 1 at p ≤ 10^−3^ there were 1709 DMRs and associated genes are listed in Supplemental Table S2 and at p < 10^−5^ there were 267 DMRs, Fig. 2B. The gene categories are presented in Fig. 4A for the p ≤ 10^−3^ datasets and show metabolism, signaling, translation, transport and development are predominant gene categories for both lake versus river comparisons. The DMR associated genes for Lake 1 versus River 2 are shown in Fig. 4B and also demonstrate metabolism and transport genes are predominant. A pathway analysis for the DMR associated genes p < 10^−3^ data set identified metabolic and endocytosis pathways for both lake versus river comparisons, Figs 4C and 5. Different sets of genes in the different lake versus river comparison within the endocytosis pathway are affected, but both suggest this pathway is potentially influenced.

## Discussion

The potential for environmentally-induced epigenetic variation to generate heritable or plastic adaptive phenotypic variation, particularly in populations where genetic variation is lacking, is promising yet largely unexplored as an alternate path to adaptation. We observed a range-expanding population of invasive snails that exhibits adaptive morphological divergence in shell shape, with relative aperture size of populations in rivers with faster water current speed significantly larger than that of populations in lakes with no current. These phenotypic differences are observed even though all populations comprise a single parthenogenetic clone and therefore lack genotypic variation and genetic recombination. We also observed a habitat specific pattern of differentially methylated regions of the DNA (DMRs) that is much greater than random variation (between sites within habitats). The association between habitat specific phenotypic variation and habitat specific DNA methylation poses the possibility that this asexual population may respond to altered environmental conditions through phenotypic shifts which are influenced by epigenetic shifts.

Epigenetic shifts can represent a response by the population to the environmental conditions encountered^42^, or they can occur spontaneously and randomly^43^. If the generation or accumulation of epigenetic differences were random rather than specific to habitat, then we would expect to find as many DMRs between sites within a habitat (e.g. Lake 1 versus Lake 2) than we would find between habitats (e.g. lake versus river), all else equal. We found that a large number of differentially methylated DNA regions were habitat specific rather than random; there are many more DMRs observed between habitats than are found within a habitat (Table 2). It remains possible that the greater number of DMRs between habitats results from drift and migration acting on epimutations because of the greater distance between lakes and rivers compared to between sites within habitats. However, this explanation is viable only if methylation is heritable and responds to evolutionary forces analogous to genetic mutations. At this point, it is not clear whether the observed habitat-specific epigenetic variants indicate an evolved response or environmentally induced non-inherited responses.

When epigenetic responses are inherited, whether spontaneous or environmentally induced, the result is selectable phenotypic variation and adaptive evolution^11^. Alternatively, when epigenetic changes are not inherited, the result is phenotypic plasticity. However, if epigenetic changes are environmentally cued and shift the phenotype in an adaptively biased direction, the result is adaptive phenotypic plasticity mediated by epigenetic alterations^22,44,45^. We observed habitat specific responses in the methylation of DNA along with an adaptive difference in shell shape morphology in *P. antipodarum*. Whether the epigenetic variation observed is a mediator of adaptive phenotypic plasticity or generating heritable phenotypic variation which is then selected, we do not yet know. The potential of a parthenogenetic population such as the snails we study to accumulate epigenetic mutations is greater because these populations forego meiosis and the associated reset of methylation between generations^24,46^. Consistent with this mechanism, preliminary results suggest that, within isofemale clonal lineages, epigenetic variation is transmitted with little change in a novel environment for at least one generation, and that phenotypic adjustments take at least two generation (M Smithson, M Skinner, and M Dybdahl, *personal communication*). These results suggest that the observed habitat specific epigenetic responses might result from the action of evolutionary forces.

The effect of any habitat-specific DMR on snail phenotypes such as shell shape and foot muscle could derive from epigenetic alteration of gene expression due to methylation. Any alteration in the methylation of DNA in the germ cell will be expressed in all subsequent cells and tissues and thus any homogenous tissue type will provide a signature of population-specific epigenetic shift^47,48^. Using foot muscle tissue, which we expect to be correlated with relative shell aperture size and adapted to water current speed, we found that multiple genes affecting multiple gene pathways were associated with differential DNA methylation between lake and river habitats (Fig. 4). Our replicated between-habitat comparison showed that a fair proportion of DMR are shared (Fig. 2D), suggesting that these are most likely candidates to explain environmental or phenotypic differences. However, developing causal links between these differences and a particular phenotype is challenging. Expression levels of genes important to shell or foot development can be affected by regulatory regions that are megabases from expressed genes^49^ and are not represented in our limited reference genome. The epigenetic shifts documented here that may result in altered gene expression levels influencing the phenotypic differences require further investigation. Nevertheless, observations suggest that one possible explanation for the expression of adaptive shell shape variation is the generation or accumulation of epigenetic differences between habitats.

A limitation in the current analysis is that the only genome available is an expression library of the New Zealand mud snail. When the methylated DNA is immunoprecipitated (MeDIP) the DNA sequence obtained is then aligned to the expression library and only those sequence that aligned are examined. Observations indicate only 25% of the sequenced DNA aligned (Fig. 2), such that the majority is likely intergenic and not aligned to genes. Therefore, the DMRs identified are an under-representation of the DMRs present genome-wide. In addition, the DMR gene associations are now limited to gene bodies and not promoters or intergenic regions. Although evidence suggests that DNA methylation of gene bodies is involved with environmentally driven plasticity and gene expression differences in invertebrates^50,51^, a limitation to the current study is the gene associations are also an under-represented number of genes. In the future with a more complete genome, the raw data in the NCBI GEO #GSE9912 can be used to identify more complete genome-wide DMRs and associated genes. However, the current study provides the observations that epigenetic alterations exist between the different population sites.

The importance of habitat-specific patterns of methylation to adaptation is greater when genetic variation is lacking. A potential explanation for the persistence of genetically limited populations in heterogeneous environments is the generation of phenotypic variation through epigenetic alterations and changes in gene expression^27^. The associated shifts in this study between habitats in the methylation pattern and the adaptive phenotype suggest a potential connection between epigenetics and adaptive phenotypic variation. The causal link between the phenotypic shift and the epigenetic shift is yet to be determined in this system. The next step will include controlled lab induction experiments using isofemale clonal lines to determine the effect of water current speed on DNA methylation, and follow-up gene expression studies. A link between the effects of the altered methylation and the phenotypic shifts would support epigenetic variation as a path to increased phenotypic variation and ultimately rapid adaptation of a population.

## Methods

### Sample Collection

Snails were sampled from sites to maximize habitat differences related to current speed (lakes vs. rivers). We sampled from two lakes in western Oregon and Washington, and two river sites in the closest available river populations in central Idaho (snails have colonized lakes and not rivers in western Oregon and Washington, and the opposite inland)^38^. Lake snails were collected from Lake 1: Lake Lytle in Rockaway Beach, OR (45.6272°N, 123.9392°W) and a Lake 2: beach area of Lake Washington in Seattle, WA (47.6971°N, 122.2711°W). River snails were collected from River 2: a tributary spring stream of the Snake River at Ritter Island (42.7439°N, 114.8420°W) and River 1: the main channel of the river (42.7439°N, 114.8416°W) near Wendell, ID. These river sites are part of the 1000 Springs complex, a unique geological area where springs flow continuously from canyon walls that source in “lost rivers” of south-central Idaho. Samples were obtained by searching the substrate and scraping snails off the underside of rocks and woody debris. The samples were maintained on wet paper towels and kept cool until they reached the laboratory at Washington State University, Pullman, WA. In the laboratory, the samples are kept in water in an environmental chamber that is held at 14 °C with a 12:12 day/night cycle for several days until they were processed for phenotypic and molecular analysis.

### Shell Shape Measures and Analysis

Samples were photographed under a dissecting scope. These photographs were analyzed using ImageJ. Four phenotypic measures were taken. The first is shell height, the longest length from the base of the aperture to the tip of the spiral. The second phenotypic measure is the result of dividing the aperture width by the spire height. This measure gives an indication of how large the foot muscle is in relation to overall body size.

These two phenotypic traits were compared using a nested ANOVA (Table 1). In the model, we account for variation between lake and river habitats (Habitat), between sites within habitat (Sample site), and among the pools within site (DNA pool). Because the snail samples were split into 3 pools for each site for the molecular analysis, used the DNA pool term to verify the homogeneity of the mean phenotype among pools.

### Tissue

After obtaining phenotypic measures for each sample the shells were cracked open and the soft tissue was separated from the shell. A thin slice of the foot pad tissue was separated from the remainder of the soft tissue and this foot tissue was stored in Nanopure™ water and immediately frozen. We chose to examine foot tissue because histology of this tissue shows it to be a relatively homogeneous cell population. This characteristic is important for comparing methylation profiles among separate individuals and populations, so that cell-specific differences in methylation do not confound the analysis. Foot pad slices from each of 10 or 20 individuals were pooled and DNA was isolated from each of these pools. Because the DNA yield was low in some samples, we added additional individuals to make the DNA yield similar across all pools and all sampling sites.

### Genomic DNA Preparation

Genomic DNA from snail foot tissue was prepared as follows. Snail tissue was suspended in 100 μl of 1 × Phosphate Buffered Saline (PBS), then 820 μl DNA extraction buffer (50 mM Tris pH8, 10 mM EDTA pH8, 0.5% SDS) and 80 μl Proteinase K (20 mg/ml) was added and the sample incubated on a rotator at 55 C for 2–3 hours. After incubation, 300 μl of protein precipitation solution (Promega, A795A) was added, the sample mixed and incubated on ice for 15 minutes, then spun at 4 C at 13,000 rpm for 20 minutes. The supernatant was transferred to a fresh tube, then precipitated over night with the same volume 100% isopropanol and 2 μl glycoblue at −20 C. The sample was then centrifuged and the pellet washed with 75% ethanol, then air-dried and resuspended in 100 μl H_2_O. DNA concentration was measured using the Nanodrop (Thermo Fisher).

### Methylated DNA Immunoprecipitation MeDIP

Methylated DNA Immunoprecipitation (MeDIP) from genomic DNA was performed as follows. For the snail samples, approximately 6 μg of each genomic DNA pool was diluted to 130 μl with TE buffer into the appropriate Covaris tube. Covaris was set to 300 bp program. 10 μl of each sonicated DNA was run on 1.5% agarose gel to verify fragment size. The sonicated DNA was transferred from the Covaris tube to a 1.7 ml microfuge tube and the volume measured. The sonicated DNA was then diluted with TE buffer (10 mM Tris HCl, pH 7.5; 1 mM EDTA) to 400 μl, heat-denatured for 10 min at 95 °C, then immediately cooled on ice for 5 min. Then 100 μl of 5X IP buffer and 5 μg of antibody (monoclonal mouse anti 5-methyl cytidine; Diagenode #C15200006) were added to the denatured sonicated DNA. The DNA-antibody mixture was incubated overnight on a rotator at 4 °C.

The following day magnetic beads (Dynabeads M-280 Sheep anti-Mouse IgG; 11201D) were pre-washed as follows: The beads were resuspended in the vial, then the appropriate volume (50 μl per sample) was transferred to a microfuge tube. The same volume of Washing Buffer (at least 1 mL) was added and the bead sample was resuspended. Tube was then placed into a magnetic rack for 1–2 minutes and the supernatant discarded. The tube was removed from the magnetic rack and the washed beads were resuspended in the same volume of 1xIP buffer as the initial volume of beads. 50 μl of beads were added to the 500 μl of DNA-antibody mixture from the overnight incubation, then incubated for 2 h on a rotator at 4 °C.

After the incubation, the beads were washed three times with 1X IP buffer as follows: The tube was placed into magnetic rack for 1–2 minutes and the supernatant discarded, then washed with 1xIP buffer 3 times. The washed beads were then resuspended in 250 μl digestion buffer (50 mM Tris-HCI pH 8, 10 mM EDTA pH 8, 0.5% SDS) with 3.5 μl Proteinase K (20 mg/ml). The sample was then incubated for 2–3 hours on a rotator at 55 °C. After incubation, the tube was put again into the magnetic rack for 3 minutes and the supernatant removed to a new microfuge tube. The beads were discarded. 250 μl of buffered Phenol-Chloroform-Isoamyl alcohol solution was added and the tube vortexed for 30 sec then centrifuged at 14,000 rpm for 5 min at room temperature. The aqueous supernatant was carefully removed and transferred to a fresh microfuge tube. Then 250 μl chloroform were added to the supernatant from the previous step, vortexed for 30 sec and centrifuged at 14,000 rpm for 5 min at room temperature. The aqueous supernatant was removed and transferred to a fresh microfuge tube. To the supernatant 2 μl of glycoblue (20 mg/ml), 20 μl of 5 M NaCl and 500 μl ethanol were added and mixed well, then precipitated in −20 °C freezer for 1 hour to overnight.

The precipitate was centrifuged at 14,000 rpm for 20 min at 4 °C and the supernatant removed, while not disturbing the pellet. The pellet was washed with 500 μl cold 70% ethanol in −20 °C freezer for 15 min. then centrifuged again at 14,000 rpm for 5 min at 4 °C and the supernatant discarded. The tube was spun again briefly to collect residual ethanol at bottom of tube and then as much liquid as possible was removed with gel loading tip. Pellet was air-dried at RT until it looked dry (about 5 minutes) then resuspended in 25 μl H_2_O or TE. DNA concentration was measured in Qubit with ssDNA kit.

### MeDIP-Seq

Snail foot pad MeDIP pools were used to create libraries for next generation sequencing at Washington State University, Genomics Core Laboratory with an Illumina® platform. All the pools consisted of 10–20 individuals for the footpad (see above) experiment, with 3 pools each for the lake versus river populations. For library preparation, we used the NEBNext® Ultra™ RNA Library Prep Kit for Illumina® starting at step 1.4 of the manufacturer’s protocol to generate double stranded DNA. After this step the manufacturer’s protocol was followed. Each pool received a separate index primer. Next generation sequencing (NGS) was performed at the WSU Spokane Genomic Core laboratory using the Illumina® HiSeq. 2500 with a PE50 application, with a read size of approximately 50 bp and approximately 100 million reads per pool. Three libraries each were run in one lane comparing river and lake. All sequencing data has been deposited to NCBI GEO (GEO #GSE99912).

### Bioinformatics and Statistics

Basic read quality was verified using summaries produced by the FastQC program^52^. The reads for each sample for DMR analyses were mapped to a *P. antipodarum* transcriptome reference^40^ using Bowtie2^53^ with default parameter options. The mapped read files were then converted to sorted BAM files using SAMtools^54^. To identify DMR, the reference genome was broken into 100 bp windows. The MEDIPS R package^55^ was used to calculate differential coverage between lake and river sample groups. The edgeR p-value^56^ was used to determine the relative difference between the two groups for each genomic window. Windows with an edgeR p-value less than 10^−5^ were considered DMRs. The DMR edges were extended until no genomic window with an edgeR p-value less than 0.1 remained within 1000 bp of the DMR. CpG density and other information was then calculated for the DMR based on the reference genome.

DMR were annotated using a Blastn^57^ search against NCBI’s preformatted “nt” database. The rentrez^58^ R package was used to process the Blastn results and to download linked gene information from NCBI. The genes that overlapped with DMR were then input into the KEGG pathway search^59,60^ to identify associated pathways. The DMR associated genes were manually then sorted into functional groups by consulting information provided by the DAVID^61^, and Panther^62^, and databases incorporated into an internal curated database (www.skinner.wsu.edu under genomic data).

## Electronic supplementary material

Supplemental Material
