# Supplementary material for: Epigenetics and adaptive phenotypic variation between habitats in an asexual snail
Source: Sci Rep. 2017 Oct 26;7:14139. doi: 10.1038/s41598-017-14673-6 (PMC5658341; doi:10.1038/s41598-017-14673-6)
Supplement: Supplementary file 1 — Supplemental Material [file 41598_2017_14673_MOESM1_ESM.pdf]

Epigenetics and adaptive phenotypic variation between habitats in an asexual snail

Jennifer Madrid Thorson[1], Mark Smithson[1], Daniel Beck, Ingrid Sadler-Riggleman, Eric Nilsson, Mark Dybdahl*[2] and Michael K. Skinner*[2]

Supplemental Material

Supplemental Figure and Table Legends

Supplemental Figure S1. Snail foot pad histology. The snail foot pad histology (HE stain) is shown that identified the foot muscle cells and region dissected for the purified muscle cell population. The expanded snail morphology for the operculum foot pad and body loops with gonad are also shown. The 100μm indicator is provided for each micrograph.

Supplemental Table S1. The Lake 1 versus River 2 DMR characteristics and associated genes for $p<10^{-3}$ data set. The DMR name and associated genome contig, specific base pair start and stop with size, DMR length (bp), number of significant windows (#SigWin), minimum p-value, CpG number (#) and density, and associated gene with category listed. The NA indicates not applicable for associated gene.

Supplemental Table S2. The Lake 2 versus River 1 DMR characteristics and associated genes for p<10-3 data set. The DMR name and associated genome contig, specific base pair start and stop with size, DMR length (bp), number of significant windows (#SigWin), minimum p-value, CpG number (#) and density, and associated gene with category listed. The NA indicates not applicable for associated gene.

Supplemental Figure S1 (Color)

Snail Foot Pad Histology

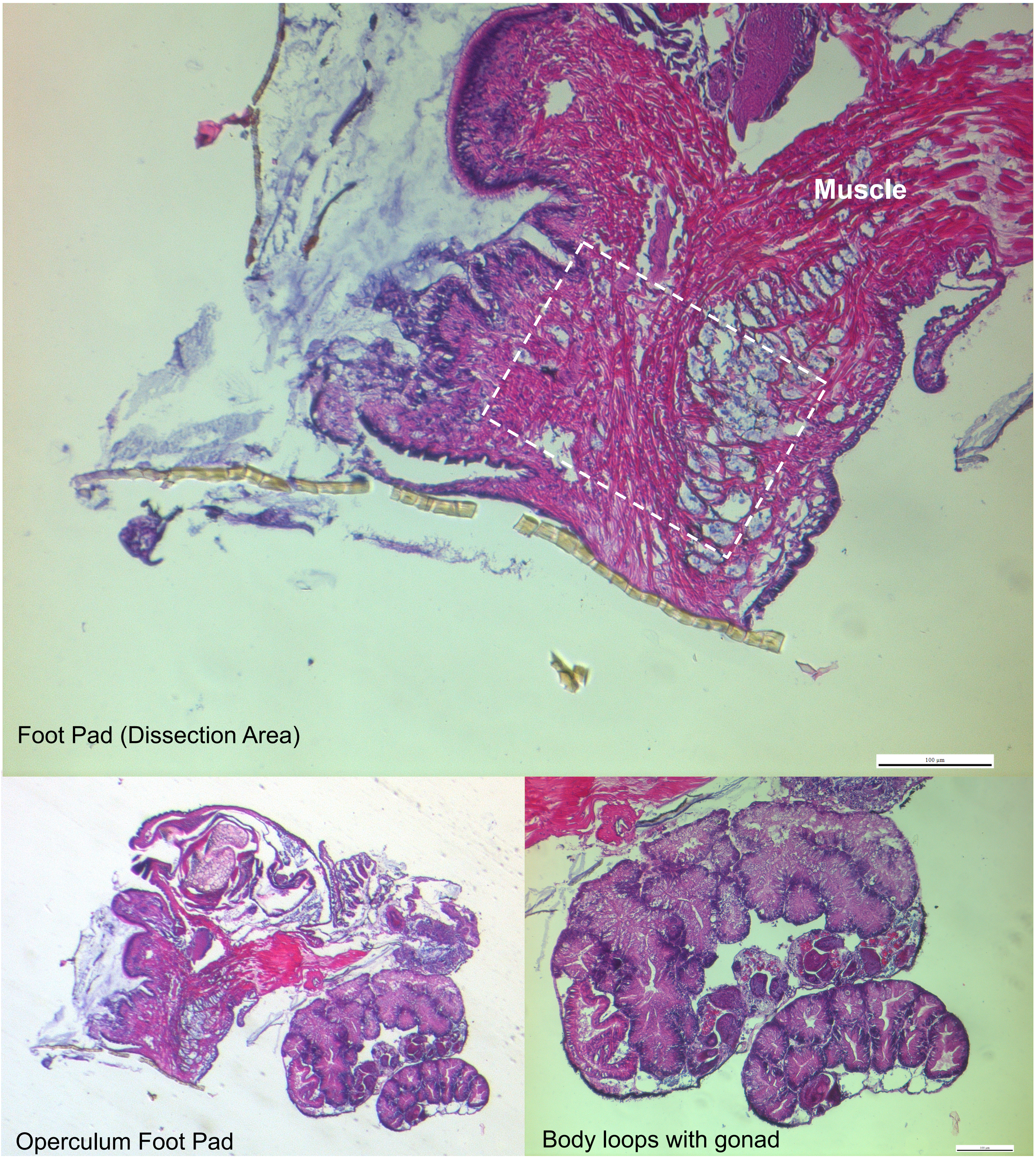

Supplemental Table S1
Lake 1 vs. River 2

DMR & Contig	Start	Stop	Length (bp)	# SigWin	minP-value	CpG #	CpG Density	Gene Annotation	Gene Category
DMRcontig00012:29574	29574	30400	827	1	0.00022678	16	1.934703748	COX1	metabolism and transport
DMRcontig00031:72901	72901	73500	600	1	0.000636648	34	5.666666667	NA	NA
DMRcontig00036:84501	84501	85400	900	2	1.85E-05	27	3	NA	NA
DMRcontig00039:91701	91701	92400	700	1	0.000814357	28	4	LOC105336924;eif2s3	translation and protein modification
DMRcontig00041:97401	97401	97900	500	1	0.000654548	22	4.4	NA	NA
DMRcontig00043:101601	101601	102100	500	1	6.85E-05	15	3	NA	NA
DMRcontig00056:135501	135501	135800	300	1	0.000596882	3	1	NA	NA
DMRcontig00059:142301	142301	143530	1230	2	1.04E-07	77	6.260162602	NA	NA
DMRcontig00061:147701	147701	148200	500	2	0.000418845	27	5.4	IscW_ISCW023076	translation and protein modification
DMRcontig00066:158501	158501	158700	200	1	0.000212543	16	8	NA	NA
DMRcontig00067:161201	161201	161400	200	1	2.12E-05	2	1	NA	NA
DMRcontig00068:162709	162709	165230	2522	16	6.34E-05	163	6.463124504	NA	NA
DMRcontig00070:169201	169201	170086	886	1	0.00046511	31	3.498871332	PSMA4	protease
DMRcontig00071:171101	171101	171500	400	1	7.60E-08	8	2	NA	NA
DMRcontig00073:175401	175401	176987	1587	3	1.03E-05	26	1.638311279	NA	NA
DMRcontig00078:187101	187101	187800	700	1	7.00E-05	16	2.285714286	NA	NA
DMRcontig00087:207201	207201	208000	800	3	1.59E-07	30	3.75	LOC107219277	cytoskeleton
DMRcontig00092:218307	218307	219300	994	4	2.24E-05	18	1.810865191	NA	NA
DMRcontig00094:223701	223701	223953	253	3	3.71E-07	13	5.138339921	LOC106135501	translation and protein modification
DMRcontig00098:231335	231335	232698	1364	1	0.000339295	24	1.759530792	NA	NA
DMRcontig00102:240101	240101	240500	400	1	0.000194281	26	6.5	LOC106179196;LOC106152685	metabolism and transport
DMRcontig00103:242501	242501	243300	800	2	7.15E-06	28	3.5	LOC103362722;atp5a1	metabolism and transport
DMRcontig00105:246801	246801	248300	1500	2	4.80E-05	71	4.733333333	NA	NA
DMRcontig00111:260701	260701	261200	500	2	2.42E-07	17	3.4	NA	NA
DMRcontig00112:262601	262601	263400	800	2	0.000512285	20	2.5	NA	NA
DMRcontig00122:285601	285601	286100	500	2	0.000104716	18	3.6	NA	NA
DMRcontig00141:329601	329601	330000	400	1	0.000684739	11	2.75	NA	NA
DMRcontig00148:344113	344113	344600	488	2	7.16E-05	13	2.663934426	gnb2l1	signaling
DMRcontig00164:379746	379746	380600	855	3	2.19E-05	21	2.456140351	NA	NA
DMRcontig00183:425101	425101	425797	697	1	0.000528472	17	2.43902439	NA	NA
DMRcontig00217:499101	499101	499500	400	2	4.01E-05	12	3	NA	NA
DMRcontig00224:514101	514101	514600	500	1	8.76E-06	18	3.6	NA	NA
DMRcontig00238:544901	544901	546680	1780	4	8.55E-06	41	2.303370787	BRAFLDRAFT_260655;PHB	miscellaneous
DMRcontig00261:596101	596101	596742	642	2	1.08E-06	13	2.024922118	LOC101407835;PSMB5	protease
DMRcontig00262:597743	597743	598900	1158	2	7.42E-05	27	2.331606218	NA	NA
DMRcontig00272:619570	619570	620100	531	2	0.000197797	36	6.779661017	LOC106602804;LOC106609995;rps6	translation and protein modification
DMRcontig00278:632501	632501	633500	1000	1	0.000851583	16	1.6	NA	NA
DMRcontig00281:639901	639901	640300	400	3	5.22E-06	6	1.5	NA	NA
DMRcontig00295:669943	669943	670100	158	1	0.000981487	3	1.898734177	Dsec\GM18298;Dyak\RpL12;RpL12	development
DMRcontig00307:696601	696601	698000	1400	3	0.000403599	41	2.928571429	NA	NA
DMRcontig00350:792601	792601	792900	300	1	0.000960866	15	5	NA	NA
DMRcontig00362:818001	818001	819100	1100	1	0.000541313	48	4.363636364	LOC104957502;Aldh2	metabolism
DMRcontig00375:845701	845701	846557	857	2	6.49E-06	55	6.417736289	NA	NA
DMRcontig00379:854401	854401	855200	800	1	0.000140829	42	5.25	NA	NA
DMRcontig00386:868919	868919	869100	182	1	2.41E-05	1	0.549450549	NA	NA
DMRcontig00396:891101	891101	891600	500	4	3.04E-06	13	2.6	NA	NA
DMRcontig00416:934311	934311	935100	790	2	0.00014278	27	3.417721519	NA	NA
DMRcontig00417:936701	936701	937600	900	3	0.000194839	41	4.555555556	NA	NA
DMRcontig00419:940901	940901	941100	200	1	0.000629524	4	2	NA	NA
DMRcontig00428:961001	961001	962000	1000	3	0.000140867	42	4.2	SPRG_12594	cytoskeleton
DMRcontig00431:967701	967701	968200	500	1	0.000376287	14	2.8	NA	NA
DMRcontig00435:975901	975901	976000	100	1	0.00098	10	10	NA	NA
DMRcontig00436:977801	977801	978500	700	1	0.000797097	16	2.285714286	NA	NA
DMRcontig00446:999201	999201	999700	500	1	0.000261754	36	7.2	NA	NA
DMRcontig00449:1005054	1005054	1005700	647	1	0.000744393	51	7.882534776	NA	NA
DMRcontig00457:1022301	1022301	1023300	1000	2	0.000102895	16	1.6	NA	NA
DMRcontig00458:1025101	1025101	1025300	200	2	6.90E-05	8	4	NA	NA
DMRcontig00460:1029001	1029001	1029400	400	1	0.000948988	5	1.25	NA	NA
DMRcontig00468:1046131	1046131	1046700	570	4	9.22E-06	7	1.228070175	NA	NA
DMRcontig00470:1050501	1050501	1051500	1000	1	0.000600303	34	3.4	LOC105334244;psmc5	protease
DMRcontig00472:1054601	1054601	1056800	2200	2	3.71E-05	43	1.954545455	NA	NA
DMRcontig00477:1066701	1066701	1067100	400	1	3.79E-06	11	2.75	NA	NA
DMRcontig00482:1076301	1076301	1076800	500	1	7.79E-05	21	4.2	HELRODRAFT_172351	miscellaneous
DMRcontig00495:1104301	1104301	1105200	900	3	5.56E-05	32	3.555555556	NA	NA
DMRcontig00498:1111401	1111401	1111900	500	3	1.95E-05	26	5.2	NA	NA
DMRcontig00508:1132601	1132601	1133700	1100	4	4.16E-06	56	5.090909091	DDOST	metabolism
DMRcontig00522:1161201	1161201	1161700	500	1	0.000509238	10	2	NA	NA
DMRcontig00542:1200801	1200801	1201900	1100	1	0.000631845	26	2.363636364	NA	NA
DMRcontig00548:1213265	1213265	1214000	736	4	5.59E-07	19	2.581521739	NA	NA
DMRcontig00549:1215165	1215165	1215300	136	1	0.000791193	3	2.205882353	Gm5461	miscellaneous
DMRcontig00550:1217801	1217801	1218000	200	1	0.000641798	4	2	NA	NA
DMRcontig00553:1223757	1223757	1223800	44	1	0.000981487	0	0	NA	NA
DMRcontig00565:1249429	1249429	1250300	872	1	0.000248325	50	5.733944954	LOC101169281	translation and protein modification
DMRcontig00575:1271201	1271201	1271500	300	1	0.000641026	16	5.333333333	NA	NA

DMRcontig00584:1290701	1290701	1291600	900	1	0.000103353	11	1.222222222	NA	NA
DMRcontig00586:1294601	1294601	1295100	500	2	6.63E-06	19	3.8	NA	NA
DMRcontig00587:1296201	1296201	1296400	200	1	0.000548731	2	1	NA	NA
DMRcontig00589:1300901	1300901	1301500	600	4	2.23E-05	13	2.166666667	NA	NA
DMRcontig00593:1308111	1308111	1308600	490	1	5.15E-06	14	2.857142857	NA	NA
DMRcontig00601:1325701	1325701	1326000	300	1	0.000146081	5	1.666666667	LOC104955137	development
DMRcontig00603:1329501	1329501	1330400	900	2	4.68E-05	32	3.555555556	NA	NA
DMRcontig00604:1331801	1331801	1332600	800	2	0.00093968	14	1.75	NA	NA
DMRcontig00611:1346001	1346001	1346800	800	2	0.000283132	16	2	NA	NA
DMRcontig00615:1355201	1355201	1355400	200	1	0.000198085	9	4.5	NA	NA
DMRcontig00665:1457124	1457124	1457900	777	1	0.000292564	19	2.445302445	NA	NA
DMRcontig00666:1459201	1459201	1460100	900	1	0.00088201	54	6	LOC104826917	metabolism and transport
DMRcontig00670:1467901	1467901	1468400	500	1	0.000118274	9	1.8	NA	NA
DMRcontig00680:1487501	1487501	1488000	500	1	0.000639665	25	5	Trnae-cuc	translation and protein modification
DMRcontig00690:1507701	1507701	1508300	600	1	0.000193818	8	1.333333333	NA	NA
DMRcontig00703:1534101	1534101	1534500	400	1	0.000490345	9	2.25	NA	NA
DMRcontig00708:1546401	1546401	1546649	249	2	0.000430207	5	2.008032129	NA	NA
DMRcontig00709:1548301	1548301	1549827	1527	2	0.000114406	60	3.929273084	NA	NA
DMRcontig00711:1552601	1552601	1553500	900	1	0.000613941	41	4.555555556	LOC101857978	receptors and binding proteins
DMRcontig00731:1594925	1594925	1596000	1076	1	0.000235945	37	3.43866171	NA	NA
DMRcontig00756:1648101	1648101	1648700	600	1	0.000563833	29	4.833333333	NA	NA
DMRcontig00757:1650101	1650101	1650500	400	2	1.82E-05	12	3	NA	NA
DMRcontig00763:1662301	1662301	1662600	300	2	1.34E-06	18	6	NA	NA
DMRcontig00767:1670201	1670201	1671600	1400	3	1.24E-06	6	0.428571429	Gm7993;Cnot6l	metabolism
DMRcontig00775:1686901	1686901	1687300	400	1	4.18E-05	26	6.5	NA	NA
DMRcontig00777:1690319	1690319	1690800	482	3	3.04E-06	14	2.904564315	NA	NA
DMRcontig00809:1753001	1753001	1753300	300	1	0.000234161	17	5.666666667	LOC104932872	translation
DMRcontig00816:1766701	1766701	1766900	200	1	0.000686943	6	3	LOC105017692	translation
DMRcontig00831:1796601	1796601	1796900	300	1	0.000340641	10	3.333333333	NA	NA
DMRcontig00849:1832401	1832401	1833301	901	2	7.83E-05	15	1.66481687	NA	NA
DMRcontig00873:1881501	1881501	1882400	900	4	1.30E-05	23	2.555555556	NA	NA
DMRcontig00885:1906722	1906722	1908500	1779	2	1.52E-05	38	2.136031478	NA	NA
DMRcontig00887:1911301	1911301	1912200	900	3	1.68E-07	22	2.444444444	NA	NA
DMRcontig00908:1953801	1953801	1954900	1100	1	0.000993925	17	1.545454545	NA	NA
DMRcontig00915:1967929	1967929	1968800	872	3	3.71E-06	23	2.637614679	NA	NA
DMRcontig00917:1972101	1972101	1972600	500	1	0.000989819	24	4.8	NA	NA
DMRcontig00930:1999201	1999201	1999800	600	2	9.32E-05	15	2.5	NA	NA
DMRcontig00936:2010301	2010301	2010700	400	1	0.000177417	23	5.75	NA	NA
DMRcontig00938:2014401	2014401	2015197	797	2	0.000145467	19	2.383939774	NA	NA
DMRcontig00952:2041601	2041601	2042900	1300	4	0.000133694	42	3.230769231	NA	NA
DMRcontig00958:2054701	2054701	2055598	898	4	1.26E-05	7	0.779510022	NA	NA
DMRcontig00965:2068436	2068436	2069310	875	5	1.70E-07	37	4.228571429	NA	NA
DMRcontig00968:2074513	2074513	2074700	188	1	0.000862153	5	2.659574468	NA	NA
DMRcontig00970:2079301	2079301	2079600	300	2	2.84E-05	5	1.666666667	NA	NA
DMRcontig00975:2089701	2089701	2090500	800	1	7.10E-05	44	5.5	NA	NA
DMRcontig00982:2103401	2103401	2103800	400	4	4.50E-11	8	2	zbtb8os	transcription
DMRcontig00983:2105482	2105482	2106200	719	3	0.000489076	55	7.649513213	NA	NA
DMRcontig00988:2116101	2116101	2116300	200	2	4.29E-05	1	0.5	NA	NA
DMRcontig00993:2126101	2126101	2126600	500	1	0.00082468	13	2.6	NA	NA
DMRcontig01001:2141145	2141145	2142500	1356	2	0.000133661	35	2.581120944	NA	NA
DMRcontig01002:2143566	2143566	2144300	735	1	0.000901859	14	1.904761905	NA	NA
DMRcontig01004:2147301	2147301	2147900	600	2	1.56E-05	45	7.5	NA	NA
DMRcontig01013:2168401	2168401	2168800	400	1	0.000721936	5	1.25	NA	NA
DMRcontig01014:2170401	2170401	2171264	864	1	0.000627174	36	4.166666667	NA	NA
DMRcontig01029:2199801	2199801	2200700	900	2	1.11E-05	22	2.444444444	NA	NA
DMRcontig01030:2202125	2202125	2203200	1076	1	0.000321487	72	6.691449814	LOC103372548;ARSJ	metabolism
DMRcontig01031:2204401	2204401	2204600	200	1	0.000748574	6	3	NA	NA
DMRcontig01042:2228201	2228201	2228800	600	1	0.000100435	20	3.333333333	NA	NA
DMRcontig01050:2247101	2247101	2247800	700	1	0.000974062	35	5	NA	NA
DMRcontig01054:2257001	2257001	2257400	400	2	0.000878641	19	4.75	NA	NA
DMRcontig01061:2269801	2269801	2270030	230	2	0.000641779	4	1.739130435	NA	NA
DMRcontig01074:2295128	2295128	2295900	773	1	0.000755324	38	4.915912031	LOC106106034	metabolism
DMRcontig01078:2302191	2302191	2303200	1010	3	8.14E-06	45	4.455445545	NA	NA
DMRcontig01081:2308201	2308201	2309000	800	1	0.000529197	29	3.625	NA	NA
DMRcontig01093:2331701	2331701	2332100	400	1	0.00069613	10	2.5	NA	NA
DMRcontig01099:2343201	2343201	2344300	1100	2	6.42E-05	19	1.727272727	NA	NA
DMRcontig01100:2346701	2346701	2347600	900	1	0.000786183	27	3	NA	NA
DMRcontig01101:2349601	2349601	2349900	300	2	5.24E-05	3	1	NA	NA
DMRcontig01102:2351401	2351401	2351976	576	1	0.000143794	9	1.5625	LOC106590555;sec61g	metabolism
DMRcontig01104:2355001	2355001	2355100	100	1	1.35E-05	1	1	NA	NA
DMRcontig01124:2393901	2393901	2394500	600	1	0.000424817	11	1.833333333	NA	NA
DMRcontig01143:2431051	2431051	2431800	750	2	1.01E-05	28	3.733333333	NA	NA
DMRcontig01148:2441801	2441801	2442600	800	1	0.000976639	22	2.75	LOC102045438;PUF60	translation
DMRcontig01149:2444001	2444001	2444300	300	1	0.000216467	12	4	LOC106588218;CSNK2B	signaling
DMRcontig01150:2445701	2445701	2447200	1500	1	0.000335154	59	3.933333333	LOC105383268;mdh1	metabolism and transport
DMRcontig01166:2477401	2477401	2477800	400	1	0.000286845	14	3.5	NA	NA
DMRcontig01167:2479460	2479460	2480600	1141	9	1.80E-06	50	4.382120947	LOC105888664	epigenetic
DMRcontig01183:2511850	2511850	2513100	1251	3	1.77E-05	76	6.075139888	NA	NA

DMRcontig01195:2536901	2536901	2538000	1100	2	1.12E-05	41	3.727272727	CpipJ_CPIJ012380;CpipJ_CPIJ012390;AgaP_AGAP012711;AgaP_AGAP003913	epigenetic
DMRcontig01198:2543101	2543101	2543976	876	1	0.00098	13	1.484018265	NA	NA
DMRcontig01200:2547201	2547201	2547700	500	3	6.27E-06	14	2.8	NA	NA
DMRcontig01213:2575301	2575301	2576000	700	2	1.19E-05	14	2	NA	NA
DMRcontig01225:2598101	2598101	2599400	1300	2	2.18E-05	61	4.692307692	NA	NA
DMRcontig01265:2679301	2679301	2680100	800	2	0.000154407	42	5.25	LOC101874104;RHOC	signaling
DMRcontig01266:2681215	2681215	2681400	186	1	0.00087782	0	0	NA	NA
DMRcontig01270:2688501	2688501	2688700	200	1	0.00014352	11	5.5	RPL35A	translation
DMRcontig01301:2749601	2749601	2750100	500	1	0.00031928	20	4	NA	NA
DMRcontig01308:2763401	2763401	2764200	800	5	3.38E-06	28	3.5	NA	NA
DMRcontig01316:2779001	2779001	2779700	700	1	0.0005916	12	1.714285714	Ubl5	protease
DMRcontig01318:2782501	2782501	2783000	500	2	0.000239796	16	3.2	NA	NA
DMRcontig01321:2788601	2788601	2789100	500	2	0.000306305	17	3.4	NA	NA
DMRcontig01330:2805801	2805801	2807566	1766	1	0.00086219	76	4.303510759	NA	NA
DMRcontig01333:2813001	2813001	2813100	100	1	0.000887811	6	6	LOC105223462	transcription
DMRcontig01342:2829501	2829501	2829600	100	1	0.000181304	6	6	NA	NA
DMRcontig01356:2856101	2856101	2856300	200	2	3.77E-07	19	9.5	NA	NA
DMRcontig01357:2857601	2857601	2859600	2000	2	0.000412735	30	1.5	NA	NA
DMRcontig01375:2894733	2894733	2894900	168	1	0.00098	25	14.88095238	NA	NA
DMRcontig01377:2898574	2898574	2898700	127	1	0.000419991	2	1.57480315	NA	NA
DMRcontig01385:2915301	2915301	2916200	900	2	5.23E-05	19	2.111111111	NA	NA
DMRcontig01392:2931001	2931001	2931200	200	1	0.000700832	12	6	LOC101852493;atp6v0d1	metabolism and transport
DMRcontig01422:2996101	2996101	2996185	85	1	7.99E-05	2	2.352941176	NA	NA
DMRcontig01431:3012901	3012901	3013100	200	1	0.000150641	7	3.5	NA	NA
DMRcontig01432:3014601	3014601	3015300	700	2	7.84E-05	19	2.714285714	NA	NA
DMRcontig01438:3027001	3027001	3027300	300	1	0.000739413	18	6	LOC103511100	cytoskeleton
DMRcontig01441:3033223	3033223	3035100	1878	1	0.000900017	43	2.289669862	LOC104225536;Pdia3	metabolism and transport
DMRcontig01446:3046101	3046101	3046700	600	1	0.000201463	9	1.5	NA	NA
DMRcontig01459:3070566	3070566	3071593	1028	1	0.00093122	21	2.042801556	NA	NA
DMRcontig01469:3088201	3088201	3089900	1700	3	7.46E-06	67	3.941176471	NA	NA
DMRcontig01473:3097301	3097301	3098100	800	2	1.17E-05	41	5.125	NA	NA
DMRcontig01483:3117201	3117201	3117900	700	1	0.00057084	38	5.428571429	LOC106068191	development
DMRcontig01488:3127701	3127701	3128700	1000	4	5.56E-05	44	4.4	calm1	signaling
DMRcontig01491:3135501	3135501	3136453	953	4	7.12E-08	22	2.308499475	NA	NA
DMRcontig01498:3150201	3150201	3150700	500	1	0.00080205	7	1.4	NA	NA
DMRcontig01510:3172714	3172714	3173100	387	1	0.000428863	22	5.684754522	si:ch73-359m17.2;trnap-agg	miscellaneous
DMRcontig01519:3189706	3189706	3190300	595	1	8.65E-05	34	5.714285714	NSDHL	metabolism and transport
DMRcontig01525:3201101	3201101	3201600	500	3	1.38E-05	21	4.2	NA	NA
DMRcontig01526:3203201	3203201	3204000	800	1	0.000604642	45	5.625	SLC34A1	metabolism and transport
DMRcontig01531:3212001	3212001	3212200	200	1	0.00048929	6	3	NA	NA
DMRcontig01534:3218001	3218001	3218200	200	1	0.000793538	2	1	LOC100701017;LOC100700746;LOC100700480;LOC100701290	unknown
DMRcontig01538:3225901	3225901	3226387	487	1	0.000775816	12	2.464065708	NA	NA
DMRcontig01543:3236001	3236001	3236800	800	1	4.84E-05	24	3	NA	NA
DMRcontig01554:3256701	3256701	3257100	400	1	0.000222805	12	3	NA	NA
DMRcontig01555:3258301	3258301	3260000	1700	5	4.87E-05	65	3.823529412	NA	NA
DMRcontig01572:3291801	3291801	3292200	400	1	0.000763362	15	3.75	NA	NA
DMRcontig01581:3307201	3307201	3307700	500	1	0.000551644	8	1.6	NA	NA
DMRcontig01591:3327901	3327901	3328180	280	1	0.000753573	7	2.5	NA	NA
DMRcontig01596:3336351	3336351	3336900	550	2	0.000286245	21	3.818181818	NA	NA
DMRcontig01603:3349012	3349012	3349600	589	4	7.89E-06	18	3.056027165	NA	NA
DMRcontig01607:3356220	3356220	3356400	181	1	4.57E-06	2	1.104972376	NA	NA
DMRcontig01623:3387851	3387851	3388400	550	1	0.00018018	17	3.090909091	NA	NA
DMRcontig01638:3417001	3417001	3417800	800	1	0.000100771	51	6.375	NA	NA
DMRcontig01643:3426701	3426701	3427500	800	5	2.56E-06	23	2.875	NA	NA
DMRcontig01646:3431901	3431901	3432300	400	1	0.000679929	25	6.25	CAOG_01404;casq1	miscellaneous
DMRcontig01649:3437001	3437001	3437300	300	1	0.000108997	16	5.333333333	NA	NA
DMRcontig01662:3461501	3461501	3462000	500	1	0.000478478	16	3.2	NA	NA
DMRcontig01666:3470615	3470615	3471100	486	1	0.000895728	23	4.732510288	NA	NA
DMRcontig01684:3504301	3504301	3505100	800	1	0.000222801	39	4.875	LOC105179649	receptors and binding proteins
DMRcontig01694:3524801	3524801	3525500	700	1	0.000156488	14	2	NA	NA
DMRcontig01695:3526866	3526866	3528900	2035	1	0.000425344	52	2.555282555	SAMD00019534_078930	miscellaneous
DMRcontig01703:3544001	3544001	3544600	600	1	0.000796734	26	4.333333333	NA	NA
DMRcontig01715:3568269	3568269	3568900	632	1	0.000932772	14	2.215189873	NA	NA
DMRcontig01717:3572301	3572301	3572945	645	2	0.000116937	14	2.170542636	NA	NA
DMRcontig01733:3600701	3600701	3602400	1700	3	3.73E-05	37	2.176470588	NA	NA
DMRcontig01743:3619201	3619201	3619500	300	1	0.000888491	7	2.333333333	NA	NA
DMRcontig01747:3627401	3627401	3628000	600	1	7.15E-05	14	2.333333333	NA	NA
DMRcontig01749:3631101	3631101	3632200	1100	4	4.05E-14	32	2.909090909	NA	NA
DMRcontig01763:3658501	3658501	3659340	840	4	1.05E-05	33	3.928571429	NA	NA
DMRcontig01769:3670422	3670422	3670900	479	2	0.000456391	14	2.922755741	NA	NA
DMRcontig01771:3674301	3674301	3674553	253	1	0.000349302	10	3.95256917	NA	NA
DMRcontig01775:3682401	3682401	3683400	1000	1	0.000470062	32	3.2	NA	NA
DMRcontig01793:3717701	3717701	3719000	1300	3	0.000438406	40	3.076923077	NA	NA
DMRcontig01795:3723101	3723101	3723590	490	1	0.000459316	6	1.224489796	NA	NA
DMRcontig01796:3724901	3724901	3725091	191	1	4.40E-07	4	2.094240838	NA	NA
DMRcontig01802:3736501	3736501	3737185	685	1	0.000419845	35	5.109489051	NA	NA
DMRcontig01804:3740101	3740101	3740600	500	1	0.000227635	26	5.2	NA	NA

DMRcontig01817:3768001	3768001	3768400	400	1	0.000167186	20	5	NA	NA
DMRcontig01820:3774301	3774301	3774700	400	1	0.000723758	7	1.75	LOC101860763;tubb4b	cytoskeleton
DMRcontig01829:3789601	3789601	3790600	1000	5	7.42E-06	27	2.7	NA	NA
DMRcontig01834:3799901	3799901	3800400	500	2	0.000218389	18	3.6	NA	NA
DMRcontig01839:3810401	3810401	3811300	900	1	0.000273705	41	4.555555556	NA	NA
DMRcontig01842:3816501	3816501	3817100	600	2	7.91E-05	30	5	NA	NA
DMRcontig01851:3833401	3833401	3834649	1249	5	7.00E-05	30	2.401921537	NA	NA
DMRcontig01852:3835650	3835650	3836300	651	3	2.38E-06	27	4.147465438	NA	NA
DMRcontig01859:3848601	3848601	3848819	219	1	0.000405587	4	1.826484018	NA	NA
DMRcontig01892:3913301	3913301	3913410	110	1	0.000766553	1	0.909090909	NA	NA
DMRcontig01909:3945001	3945001	3945300	300	1	0.000619461	14	4.666666667	NA	NA
DMRcontig01918:3961670	3961670	3962400	731	1	0.000309454	27	3.693570451	NA	NA
DMRcontig01935:3994001	3994001	3995300	1300	1	0.000538933	23	1.769230769	NA	NA
DMRcontig01945:4013801	4013801	4014400	600	1	0.000223205	20	3.333333333	Dvir\GJ16291	miscellaneous
DMRcontig01951:4025601	4025601	4025800	200	2	0.000113937	13	6.5	NA	NA
DMRcontig01964:4053401	4053401	4054140	740	3	3.04E-05	17	2.297297297	NA	NA
DMRcontig01981:4085101	4085101	4085500	400	1	0.00072504	4	1	NA	NA
DMRcontig01984:4090703	4090703	4091600	898	1	0.00053627	27	3.006681514	NA	NA
DMRcontig01988:4098601	4098601	4099100	500	1	0.000904783	22	4.4	NA	NA
DMRcontig01990:4103101	4103101	4103700	600	1	0.00021815	9	1.5	NA	NA
DMRcontig02006:4134901	4134901	4135200	300	2	8.56E-07	19	6.333333333	NA	NA
DMRcontig02010:4141601	4141601	4142300	700	1	0.000146022	13	1.857142857	NA	NA
DMRcontig02031:4180301	4180301	4181051	751	1	0.000856654	28	3.728362184	NA	NA
DMRcontig02038:4194201	4194201	4194900	700	1	0.000101967	38	5.428571429	LOTGIDRAFT_103737;ptdss2	miscellaneous
DMRcontig02043:4204301	4204301	4204900	600	1	0.000383011	24	4	NA	NA
DMRcontig02054:4223901	4223901	4224000	100	1	0.000860949	4	4	NA	NA
DMRcontig02055:4225706	4225706	4226207	502	1	0.000219209	8	1.593625498	ATP8;COX1	metabolism
DMRcontig02064:4243501	4243501	4244200	700	2	5.02E-05	35	5	LOC101656948;HIPK2	development
DMRcontig02078:4270267	4270267	4271235	969	2	0.000185585	53	5.469556244	NA	NA
DMRcontig02090:4294101	4294101	4294400	300	2	0.000230876	16	5.333333333	NA	NA
DMRcontig02101:4316101	4316101	4316300	200	1	0.000317306	14	7	LOC101846824	growth factor
DMRcontig02110:4333849	4333849	4335400	1552	5	3.93E-05	57	3.672680412	NA	NA
DMRcontig02121:4353901	4353901	4354500	600	2	0.000316999	35	5.833333333	NA	NA
DMRcontig02130:4369901	4369901	4370600	700	2	4.91E-05	40	5.714285714	LOC106583922;prmt1	metabolism and transport
DMRcontig02142:4392101	4392101	4392239	139	2	0.000248739	2	1.438848921	NA	NA
DMRcontig02152:4412001	4412001	4413100	1100	2	4.89E-06	24	2.181818182	NA	NA
DMRcontig02184:4472306	4472306	4472600	295	1	0.00018142	9	3.050847458	NA	NA
DMRcontig02207:4514701	4514701	4514885	185	1	0.000763362	8	4.324324324	NA	NA
DMRcontig02210:4519401	4519401	4520200	800	1	0.000607737	15	1.875	NA	NA
DMRcontig02217:4532701	4532701	4533300	600	1	0.000117703	8	1.333333333	NA	NA
DMRcontig02228:4553101	4553101	4553700	600	2	4.57E-09	28	4.666666667	NA	NA
DMRcontig02253:4600601	4600601	4601800	1200	1	0.000223205	33	2.75	NA	NA
DMRcontig02278:4646801	4646801	4647500	700	1	0.000714443	36	5.142857143	LOC100631697;LOC100633223	metabolism and transport
DMRcontig02283:4656001	4656001	4656600	600	2	1.06E-05	9	1.5	NA	NA
DMRcontig02304:4693901	4693901	4694700	800	1	0.00010192	14	1.75	NA	NA
DMRcontig02317:4717801	4717801	4718000	200	1	0.000908821	2	1	NA	NA
DMRcontig02323:4728401	4728401	4728700	300	1	0.000101207	1	0.333333333	NA	NA
DMRcontig02324:4729901	4729901	4730700	800	1	0.000162833	28	3.5	NA	NA
DMRcontig02340:4760301	4760301	4760961	661	6	9.91E-08	8	1.210287443	COX1	metabolism and transport
DMRcontig02341:4761962	4761962	4762630	669	1	9.59E-05	32	4.783258595	NA	NA
DMRcontig02345:4768540	4768540	4768800	261	1	0.000279925	17	6.513409962	NA	NA
DMRcontig02356:4788401	4788401	4788600	200	1	0.000506246	3	1.5	NA	NA
DMRcontig02377:4828349	4828349	4829100	752	2	4.06E-05	13	1.728723404	NA	NA
DMRcontig02378:4830501	4830501	4830900	400	3	0.000346496	10	2.5	NA	NA
DMRcontig02383:4839101	4839101	4839300	200	1	4.55E-05	2	1	NA	NA
DMRcontig02385:4842601	4842601	4843000	400	4	2.71E-05	5	1.25	BRAFLDRAFT_81006	unknown
DMRcontig02390:4853001	4853001	4854000	1000	1	5.87E-05	10	1	NA	NA
DMRcontig02393:4858301	4858301	4858500	200	1	0.000981487	10	5	NA	NA
DMRcontig02412:4894626	4894626	4895400	775	1	5.85E-05	23	2.967741935	NA	NA
DMRcontig02418:4906301	4906301	4907000	700	1	0.000932606	15	2.142857143	NA	NA
DMRcontig02436:4939501	4939501	4941100	1600	1	0.000692243	27	1.6875	NA	NA
DMRcontig02446:4958001	4958001	4958600	600	2	2.69E-05	12	2	NA	NA
DMRcontig02447:4960101	4960101	4960497	397	1	6.33E-05	12	3.022670025	NA	NA
DMRcontig02463:4989801	4989801	4990200	400	1	0.000976999	6	1.5	UO17_p33	miscellaneous
DMRcontig02469:4999701	4999701	5000100	400	1	4.75E-05	15	3.75	NA	NA
DMRcontig02476:5011501	5011501	5012500	1000	1	0.00047919	47	4.7	tcpb	development
DMRcontig02490:5038001	5038001	5038300	300	1	0.00012166	21	7	NA	NA
DMRcontig02503:5059001	5059001	5059500	500	1	0.000541745	4	0.8	NA	NA
DMRcontig02513:5075901	5075901	5076175	275	1	0.000718401	12	4.363636364	VOLCADRAFT_115993	miscellaneous
DMRcontig02519:5085270	5085270	5085700	431	2	0.000152059	21	4.872389791	NA	NA
DMRcontig02530:5104801	5104801	5105700	900	1	0.000135549	9	1	NA	NA
DMRcontig02531:5106832	5106832	5107700	869	2	0.000132227	18	2.071346375	NA	NA
DMRcontig02533:5111001	5111001	5111400	400	1	0.000689953	11	2.75	NA	NA
DMRcontig02552:5147801	5147801	5148391	591	2	0.000192145	20	3.384094755	NA	NA
DMRcontig02562:5164743	5164743	5166044	1302	1	0.000799041	30	2.304147465	NA	NA
DMRcontig02574:5190101	5190101	5190500	400	1	0.000824363	12	3	NA	NA
DMRcontig02579:5200201	5200201	5200300	100	1	0.000535474	5	5	NA	NA
DMRcontig02588:5216901	5216901	5217200	300	1	8.11E-07	5	1.666666667	NA	NA
DMRcontig02592:5223628	5223628	5224600	973	1	0.000506085	58	5.960945529	NA	NA
DMRcontig02609:5256801	5256801	5257869	1069	1	0.000998727	39	3.648269411	NA	NA

DMRcontig02615:5268234	5268234	5268400	167	1	0.000303032	11	6.586826347	NA	NA
DMRcontig02629:5292519	5292519	5294100	1582	4	0.000228358	64	4.04551201	LOC106052646	signaling
DMRcontig02664:5359142	5359142	5359600	459	1	0.000797504	11	2.396514161	NA	NA
DMRcontig02690:5406701	5406701	5406900	200	1	0.00060663	16	8	NA	NA
DMRcontig02701:5425946	5425946	5426400	455	1	5.28E-05	14	3.076923077	NA	NA
DMRcontig02745:5506504	5506504	5507200	697	1	8.62E-05	24	3.443328551	NA	NA
DMRcontig02752:5518901	5518901	5519400	500	1	0.000984309	18	3.6	NA	NA
DMRcontig02762:5537401	5537401	5537900	500	1	0.00074481	4	0.8	NA	NA
DMRcontig02784:5577837	5577837	5578600	764	1	0.000517574	22	2.879581152	NA	NA
DMRcontig02795:5597701	5597701	5598900	1200	1	0.00082767	82	6.833333333	NA	NA
DMRcontig02805:5617501	5617501	5617968	468	2	0.000207941	9	1.923076923	NA	NA
DMRcontig02806:5618969	5618969	5619500	532	3	0.000257907	14	2.631578947	NA	NA
DMRcontig02833:5668401	5668401	5669500	1100	2	5.59E-06	27	2.454545455	NA	NA
DMRcontig02864:5721601	5721601	5722300	700	3	3.91E-05	9	1.285714286	NA	NA
DMRcontig02872:5736801	5736801	5737000	200	1	0.000338332	11	5.5	LOC101168232	metabolism and transport
DMRcontig02890:5772901	5772901	5773500	600	1	0.0001098	11	1.833333333	NA	NA
DMRcontig02932:5851901	5851901	5852500	600	4	5.82E-05	18	3	NA	NA
DMRcontig02937:5860701	5860701	5860800	100	1	9.31E-06	1	1	NA	NA
DMRcontig02948:5879217	5879217	5879600	384	2	0.000139148	10	2.604166667	NA	NA
DMRcontig02972:5920656	5920656	5921600	945	3	0.000318251	24	2.53968254	NA	NA
DMRcontig02979:5933501	5933501	5933900	400	1	0.000111266	13	3.25	NA	NA
DMRcontig02983:5940001	5940001	5940100	100	1	0.000659206	3	3	NA	NA
DMRcontig02999:5968939	5968939	5969600	662	2	0.000131788	17	2.567975831	NA	NA
DMRcontig03004:5978301	5978301	5978799	499	2	6.40E-05	24	4.809619238	NA	NA
DMRcontig03022:6010701	6010701	6010859	159	1	0.000792202	9	5.660377358	NA	NA
DMRcontig03044:6050310	6050310	6050700	391	4	5.69E-06	23	5.882352941	NA	NA
DMRcontig03048:6058601	6058601	6058700	100	1	3.56E-05	1	1	NA	NA
DMRcontig03052:6065501	6065501	6065700	200	1	0.00068657	3	1.5	NA	NA
DMRcontig03064:6087301	6087301	6087574	274	1	0.000322286	4	1.459854015	NA	NA
DMRcontig03076:6108701	6108701	6109200	500	3	7.78E-08	15	3	NA	NA
DMRcontig03104:6157501	6157501	6158300	800	1	0.000165768	21	2.625	NA	NA
DMRcontig03111:6170001	6170001	6171300	1300	1	2.06E-05	66	5.076923077	NA	NA
DMRcontig03129:6204155	6204155	6204818	664	1	0.000119498	16	2.409638554	LOC105371236	unknown
DMRcontig03133:6210906	6210906	6211300	395	1	0.000172694	18	4.556962025	NA	NA
DMRcontig03136:6216159	6216159	6216600	442	2	2.43E-05	3	0.678733032	NA	NA
DMRcontig03142:6227201	6227201	6227400	200	1	8.83E-05	11	5.5	NA	NA
DMRcontig03145:6232321	6232321	6232800	480	2	7.00E-05	14	2.916666667	NA	NA
DMRcontig03147:6237401	6237401	6238300	900	1	0.000831708	40	4.444444444	NA	NA
DMRcontig03152:6246501	6246501	6247100	600	1	0.000126626	30	5	NA	NA
DMRcontig03173:6283001	6283001	6283200	200	1	0.000552245	15	7.5	NA	NA
DMRcontig03189:6309901	6309901	6310900	1000	3	0.000190785	17	1.7	NA	NA
DMRcontig03195:6321801	6321801	6322497	697	2	0.000177708	13	1.865136298	NA	NA
DMRcontig03213:6354801	6354801	6355247	447	1	4.32E-05	14	3.131991051	NA	NA
DMRcontig03242:6406001	6406001	6406400	400	1	0.000507124	7	1.75	NA	NA
DMRcontig03247:6415701	6415701	6415900	200	2	3.54E-06	5	2.5	BRAFLDRAFT_74748	epigenetic
DMRcontig03248:6418301	6418301	6419000	700	1	0.00059469	42	6	CNDP2	protease
DMRcontig03252:6425730	6425730	6426900	1171	1	0.000182225	56	4.782237404	NA	NA
DMRcontig03257:6435701	6435701	6436400	700	1	0.000347932	23	3.285714286	LOC105034078;rbm8a	translation
DMRcontig03266:6452101	6452101	6453199	1099	1	0.000862153	16	1.455868972	NA	NA
DMRcontig03272:6462801	6462801	6463236	436	1	0.000444407	6	1.376146789	NA	NA
DMRcontig03288:6490043	6490043	6490500	458	1	0.000904187	6	1.310043668	NA	NA
DMRcontig03290:6494212	6494212	6495100	889	1	0.000791298	11	1.237345332	NA	NA
DMRcontig03297:6506975	6506975	6507600	626	1	0.00066448	26	4.153354633	NA	NA
DMRcontig03307:6524688	6524688	6525630	943	1	0.000298985	32	3.393425239	NA	NA
DMRcontig03308:6526631	6526631	6526800	170	1	0.00058142	13	7.647058824	LOC108190067;mvk	miscellaneous
DMRcontig03315:6539801	6539801	6539947	147	1	0.000737453	3	2.040816327	NA	NA
DMRcontig03324:6554201	6554201	6554700	500	5	8.43E-06	43	8.6	LOC103291109;LOC103291110	epigenetic
DMRcontig03325:6556101	6556101	6556500	400	1	0.000314223	9	2.25	NA	NA
DMRcontig03327:6559201	6559201	6559600	400	1	0.000597106	12	3	NA	NA
DMRcontig03333:6569449	6569449	6569700	252	1	0.000538866	1	0.396825397	NA	NA
DMRcontig03338:6580101	6580101	6580600	500	1	0.000576786	17	3.4	NA	NA
DMRcontig03339:6581641	6581641	6581900	260	1	8.32E-05	12	4.615384615	NA	NA
DMRcontig03358:6616001	6616001	6616700	700	2	3.31E-06	8	1.142857143	NA	NA
DMRcontig03359:6617866	6617866	6618000	135	1	0.00074438	1	0.740740741	NA	NA
DMRcontig03360:6619701	6619701	6619900	200	1	0.000171469	9	4.5	NA	NA
DMRcontig03365:6628501	6628501	6628900	400	3	0.000245492	10	2.5	NA	NA
DMRcontig03374:6643275	6643275	6643800	526	1	0.000470605	32	6.08365019	NA	NA
DMRcontig03402:6693970	6693970	6694600	631	2	0.00037992	17	2.694136292	NA	NA
DMRcontig03406:6701401	6701401	6701800	400	1	0.000934972	23	5.75	NA	NA
DMRcontig03413:6713170	6713170	6714500	1331	2	8.20E-05	58	4.357625845	NA	NA
DMRcontig03423:6731601	6731601	6731700	100	1	0.000864338	5	5	NA	NA
DMRcontig03435:6751339	6751339	6752034	696	4	6.12E-05	10	1.436781609	NA	NA
DMRcontig03460:6793801	6793801	6794500	700	1	0.000152555	10	1.428571429	NA	NA
DMRcontig03468:6809001	6809001	6809300	300	1	0.000187411	28	9.333333333	LOC105451083;LOC105569752	protease
DMRcontig03477:6824642	6824642	6825300	659	1	0.000476838	15	2.276176024	NA	NA
DMRcontig03481:6832801	6832801	6833200	400	1	0.000840194	23	5.75	NA	NA
DMRcontig03487:6843646	6843646	6844400	755	3	4.80E-06	23	3.046357616	NA	NA
DMRcontig03488:6845418	6845418	6845998	581	1	0.000142441	11	1.893287435	NA	NA
DMRcontig03496:6860001	6860001	6860900	900	1	0.00093315	14	1.555555556	NA	NA
DMRcontig03502:6870401	6870401	6871300	900	4	5.93E-07	20	2.222222222	NA	NA

DMRcontig03524:6909401	6909401	6909800	400	2	3.30E-05	16	4	NA	NA
DMRcontig03533:6925116	6925116	6925500	385	2	2.11E-05	23	5.974025974	NA	NA
DMRcontig03535:6928582	6928582	6928700	119	1	0.00040396	10	8.403361345	NA	NA
DMRcontig03541:6938601	6938601	6939100	500	1	0.000975927	8	1.6	NA	NA
DMRcontig03543:6941701	6941701	6942100	400	1	0.000438779	17	4.25	NA	NA
DMRcontig03550:6956101	6956101	6956300	200	1	0.000638911	6	3	NA	NA
DMRcontig03551:6958401	6958401	6958997	597	1	0.000369698	21	3.51758794	NA	NA
DMRcontig03554:6964001	6964001	6964300	300	1	4.04E-06	16	5.333333333	NA	NA
DMRcontig03558:6970801	6970801	6971100	300	1	6.68E-05	10	3.333333333	NA	NA
DMRcontig03562:6977601	6977601	6977900	300	1	0.000931853	17	5.666666667	LOC102566348;SLC25A37	mitochondria
DMRcontig03565:6983201	6983201	6984000	800	1	0.000998244	42	5.25	NA	NA
DMRcontig03568:6989529	6989529	6990100	572	2	1.30E-06	9	1.573426573	NA	NA
DMRcontig03574:6999801	6999801	7.00E+06	200	1	0.000533124	4	2	Ssc5d	receptors and binding proteins
DMRcontig03595:7035001	7035001	7035600	600	1	0.000985105	16	2.666666667	NA	NA
DMRcontig03617:7072001	7072001	7072300	300	1	0.000607219	17	5.666666667	NA	NA
DMRcontig03624:7084001	7084001	7084388	388	2	5.20E-06	33	8.505154639	NA	NA
DMRcontig03628:7090434	7090434	7090764	331	1	0.000307459	5	1.510574018	NA	NA
DMRcontig03659:7144951	7144951	7145200	250	2	3.95E-05	4	1.6	NA	NA
DMRcontig03663:7151701	7151701	7152834	1134	2	1.25E-05	26	2.292768959	NA	NA
DMRcontig03673:7171001	7171001	7171135	135	1	0.00098	2	1.481481481	NA	NA
DMRcontig03679:7181601	7181601	7182200	600	2	8.64E-06	17	2.833333333	NA	NA
DMRcontig03688:7198401	7198401	7198500	100	1	0.000901972	0	0	NA	NA
DMRcontig03692:7205601	7205601	7206100	500	1	6.54E-06	27	5.4	NA	NA
DMRcontig03703:7225301	7225301	7226500	1200	1	0.000182225	26	2.166666667	NA	NA
DMRcontig03708:7235501	7235501	7235800	300	1	0.000874676	6	2	NA	NA
DMRcontig03715:7247201	7247201	7247689	489	1	0.000940261	27	5.521472393	NA	NA
DMRcontig03717:7250401	7250401	7251066	666	4	2.63E-05	18	2.702702703	NA	NA
DMRcontig03718:7252101	7252101	7252400	300	2	1.14E-07	7	2.333333333	NA	NA
DMRcontig03722:7258243	7258243	7258700	458	1	0.000745122	5	1.091703057	NA	NA
DMRcontig03723:7259801	7259801	7260300	500	3	6.09E-06	25	5	NA	NA
DMRcontig03724:7261601	7261601	7261900	300	2	0.000645689	10	3.333333333	NA	NA
DMRcontig03727:7267501	7267501	7267675	175	1	3.45E-05	6	3.428571429	NA	NA
DMRcontig03729:7270518	7270518	7271169	652	2	0.000102742	26	3.987730061	NA	NA
DMRcontig03732:7275801	7275801	7276092	292	2	0.000210475	16	5.479452055	NA	NA
DMRcontig03737:7284201	7284201	7284800	600	4	3.38E-07	15	2.5	NA	NA
DMRcontig03766:7335501	7335501	7335982	482	1	0.000490345	13	2.697095436	NA	NA
DMRcontig03771:7344801	7344801	7345468	668	1	0.000619903	23	3.443113772	NA	NA
DMRcontig03798:7392701	7392701	7393000	300	1	0.000335779	8	2.666666667	ETH_00034195	signaling
DMRcontig03809:7412233	7412233	7412500	268	1	0.000270549	4	1.492537313	NA	NA
DMRcontig03812:7417365	7417365	7418200	836	3	0.000222109	40	4.784688995	LOC106932392	unknown
DMRcontig03822:7435401	7435401	7436100	700	1	6.72E-06	5	0.714285714	NA	NA
DMRcontig03824:7439701	7439701	7440099	399	1	0.000566667	28	7.01754386	NA	NA
DMRcontig03837:7463601	7463601	7464238	638	1	0.000821044	13	2.037617555	NA	NA
DMRcontig03844:7476618	7476618	7477337	720	3	1.89E-05	17	2.361111111	NA	NA
DMRcontig03859:7503620	7503620	7503900	281	1	5.28E-05	14	4.982206406	NA	NA
DMRcontig03861:7507101	7507101	7507914	814	1	0.00019924	30	3.685503686	NA	NA
DMRcontig03864:7512043	7512043	7512540	498	5	7.91E-05	28	5.62248996	LOC105320412;LOC105320415;LOC105320418;LOC105320423;LOC105320427;LOC105320431;LOC105320440	epigenetic
DMRcontig03874:7529531	7529531	7530100	570	1	0.000817253	10	1.754385965	NA	NA
DMRcontig03924:7617301	7617301	7617594	294	2	0.000328537	10	3.401360544	NA	NA
DMRcontig03929:7625901	7625901	7626100	200	1	4.96E-05	5	2.5	NA	NA
DMRcontig03931:7629401	7629401	7629658	258	3	0.000187619	10	3.875968992	NA	NA
DMRcontig03947:7655801	7655801	7656100	300	1	0.000139713	10	3.333333333	NA	NA
DMRcontig03958:7674288	7674288	7674900	613	1	0.000547903	26	4.241435563	NA	NA
DMRcontig03959:7676084	7676084	7676500	417	2	0.000107241	12	2.877697842	NA	NA
DMRcontig03974:7702113	7702113	7703123	1011	3	4.08E-06	25	2.472799209	NA	NA
DMRcontig03976:7705535	7705535	7705800	266	1	0.000805097	9	3.383458647	NA	NA
DMRcontig03990:7731102	7731102	7731800	699	2	0.000306648	21	3.004291845	stk39.L	signaling
DMRcontig03994:7738601	7738601	7738894	294	1	0.000233106	16	5.442176871	NA	NA
DMRcontig04000:7749001	7749001	7750300	1300	2	0.000515301	69	5.307692308	NA	NA
DMRcontig04002:7753401	7753401	7754172	772	3	0.000189911	32	4.14507772	NA	NA
DMRcontig04009:7766153	7766153	7767200	1048	4	6.09E-05	42	4.007633588	NA	NA
DMRcontig04015:7776501	7776501	7777100	600	1	0.000284764	22	3.666666667	LOC101855319	receptors and binding proteins
DMRcontig04016:7778201	7778201	7778800	600	1	0.000287579	12	2	NA	NA
DMRcontig04032:7807632	7807632	7808100	469	1	0.000297035	12	2.558635394	NA	NA
DMRcontig04035:7812538	7812538	7812800	263	2	0.000425152	12	4.562737643	NA	NA
DMRcontig04037:7816207	7816207	7816900	694	1	0.000763477	24	3.458213256	kif1bp;vps26a	receptors and binding proteins
DMRcontig04057:7850501	7850501	7850900	400	1	2.28E-05	9	2.25	NA	NA
DMRcontig04064:7862101	7862101	7862353	253	1	0.00032025	10	3.95256917	NA	NA
DMRcontig04084:7897235	7897235	7897600	366	1	0.000352026	12	3.278688525	NA	NA
DMRcontig04133:7981501	7981501	7982300	800	1	0.000253647	25	3.125	NA	NA
DMRcontig04140:7994101	7994101	7994400	300	1	0.00047919	13	4.333333333	DIS3L	transcription
DMRcontig04145:8002811	8002811	8003290	480	2	4.78E-05	7	1.458333333	NA	NA
DMRcontig04157:8024101	8024101	8025300	1200	5	0.000142854	54	4.5	NA	NA
DMRcontig04162:8033801	8033801	8034185	385	2	0.000166445	8	2.077922078	NA	NA
DMRcontig04163:8035186	8035186	8035400	215	1	8.11E-05	3	1.395348837	NA	NA
DMRcontig04167:8041599	8041599	8041900	302	1	0.000118742	14	4.635761589	NA	NA
DMRcontig04192:8083201	8083201	8084078	878	1	0.000759766	27	3.075170843	gas2l2	cell cycle

DMRcontig04206:8108700	8108700	8109200	501	2	2.24E-06	26	5.189620758	NA	NA
DMRcontig04208:8112001	8112001	8112100	100	1	2.88E-05	4	4	NA	NA
DMRcontig04237:8161901	8161901	8162200	300	1	0.00022172	5	1.666666667	NA	NA
DMRcontig04240:8167101	8167101	8167200	100	1	0.000103692	1	1	NA	NA
DMRcontig04251:8185201	8185201	8185400	200	1	0.000603192	10	5	NA	NA
DMRcontig04254:8190501	8190501	8191300	800	1	0.000788046	35	4.375	NA	NA
DMRcontig04256:8194218	8194218	8194700	483	3	0.000333461	11	2.277432712	NA	NA
DMRcontig04261:8202801	8202801	8203435	635	3	7.29E-07	29	4.566929134	NA	NA
DMRcontig04280:8237901	8237901	8238100	200	1	0.000560441	1	0.5	NA	NA
DMRcontig04287:8249401	8249401	8249700	300	1	0.000258489	14	4.666666667	NA	NA
DMRcontig04344:8347801	8347801	8348092	292	1	5.32E-05	9	3.082191781	NA	NA
DMRcontig04363:8382409	8382409	8383800	1392	1	0.000786637	53	3.807471264	NA	NA
DMRcontig04381:8415001	8415001	8415500	500	1	0.000538866	23	4.6	NA	NA
DMRcontig04390:8431501	8431501	8431800	300	2	0.000119221	16	5.333333333	NA	NA
DMRcontig04391:8433401	8433401	8433600	200	1	0.000143585	7	3.5	NA	NA
DMRcontig04412:8469801	8469801	8470400	600	1	0.000540177	17	2.833333333	NA	NA
DMRcontig04415:8475156	8475156	8475800	645	2	0.000464302	26	4.031007752	NA	NA
DMRcontig04416:8476817	8476817	8477200	384	1	0.000946901	9	2.34375	NA	NA
DMRcontig04433:8505101	8505101	8505800	700	1	0.000396717	21	3	NA	NA
DMRcontig04436:8510661	8510661	8510900	240	1	6.05E-05	5	2.083333333	NA	NA
DMRcontig04439:8515845	8515845	8517000	1156	1	0.000416125	42	3.633217993	NA	NA
DMRcontig04476:8580801	8580801	8581866	1066	2	0.000201996	23	2.157598499	NA	NA
DMRcontig04484:8594401	8594401	8596200	1800	3	9.14E-05	98	5.444444444	NA	NA
DMRcontig04501:8624901	8624901	8625500	600	1	0.000568293	8	1.333333333	NA	NA
DMRcontig04526:8669401	8669401	8669787	387	2	0.000582356	7	1.80878553	NA	NA
DMRcontig04527:8670801	8670801	8671200	400	1	0.000320287	8	2	NA	NA
DMRcontig04542:8697601	8697601	8698089	489	2	2.08E-05	12	2.45398773	NA	NA
DMRcontig04554:8717113	8717113	8717200	88	1	2.59E-09	8	9.090909091	NA	NA
DMRcontig04569:8742113	8742113	8742600	488	1	0.000511566	12	2.459016393	NA	NA
DMRcontig04574:8750301	8750301	8750600	300	1	3.95E-05	0	0	NA	NA
DMRcontig04591:8778759	8778759	8778900	142	2	0.000182355	8	5.633802817	NA	NA
DMRcontig04600:8793323	8793323	8794700	1378	1	0.00071017	75	5.442670537	NA	NA
DMRcontig04621:8831101	8831101	8831702	602	4	6.79E-07	37	6.146179402	NA	NA
DMRcontig04638:8859101	8859101	8859500	400	1	8.81E-05	21	5.25	LOC104569172;fdps	miscellaneous
DMRcontig04646:8872701	8872701	8872963	263	1	0.00088282	7	2.661596958	NA	NA
DMRcontig04647:8874201	8874201	8874900	700	1	7.99E-05	31	4.428571429	NA	NA
DMRcontig04656:8890501	8890501	8890700	200	1	0.000489633	6	3	NA	NA
DMRcontig04658:8893301	8893301	8893806	506	2	0.000271176	13	2.56916996	NA	NA
DMRcontig04664:8903701	8903701	8904494	794	1	0.000252052	21	2.644836272	NA	NA
DMRcontig04667:8909601	8909601	8910400	800	1	0.000661787	30	3.75	NA	NA
DMRcontig04678:8928401	8928401	8929600	1200	1	5.78E-06	25	2.083333333	LOC101070845	metabolism and transport
DMRcontig04690:8950045	8950045	8950600	556	2	0.000396535	37	6.654676259	NA	NA
DMRcontig04694:8957501	8957501	8958300	800	2	0.000300341	12	1.5	NA	NA
DMRcontig04703:8973101	8973101	8973300	200	1	0.000100587	6	3	NA	NA
DMRcontig04714:8993250	8993250	8994000	751	3	1.07E-05	21	2.796271638	NA	NA
DMRcontig04730:9020401	9020401	9020977	577	1	0.000551224	22	3.812824957	NA	NA
DMRcontig04745:9046527	9046527	9047210	684	3	1.27E-06	8	1.169590643	NA	NA
DMRcontig04750:9055101	9055101	9056000	900	1	0.000797524	34	3.777777778	NA	NA
DMRcontig04752:9058501	9058501	9059000	500	2	4.29E-05	18	3.6	NA	NA
DMRcontig04756:9066901	9066901	9067800	900	1	0.000550361	43	4.777777778	LOC106612978	extra cellular matrix
DMRcontig04761:9076152	9076152	9076500	349	1	0.000964625	5	1.432664756	NA	NA
DMRcontig04764:9081901	9081901	9082400	500	1	0.000237305	27	5.4	NA	NA
DMRcontig04765:9083801	9083801	9084000	200	1	0.000918643	11	5.5	NA	NA
DMRcontig04782:9113501	9113501	9114200	700	2	1.16E-07	27	3.857142857	NA	NA
DMRcontig04790:9126601	9126601	9127100	500	3	2.18E-06	24	4.8	NA	NA
DMRcontig04814:9166701	9166701	9167500	800	1	0.000202232	13	1.625	NA	NA
DMRcontig04818:9174201	9174201	9174700	500	5	4.43E-06	15	3	NA	NA
DMRcontig04837:9206225	9206225	9206800	576	3	2.27E-06	13	2.256944444	NA	NA
DMRcontig04852:9232301	9232301	9232500	200	1	0.000552238	14	7	NA	NA
DMRcontig04871:9262501	9262501	9263300	800	3	1.06E-05	27	3.375	NA	NA
DMRcontig04912:9331501	9331501	9331900	400	1	2.49E-05	12	3	NA	NA
DMRcontig04925:9355301	9355301	9355771	471	1	4.14E-05	24	5.095541401	NA	NA
DMRcontig04930:9362801	9362801	9363300	500	2	0.000213555	15	3	NA	NA
DMRcontig04958:9410055	9410055	9410400	346	1	0.000345527	17	4.913294798	NA	NA
DMRcontig04964:9419916	9419916	9421100	1185	1	0.000472138	52	4.388185654	LOC107228172	metabolism
DMRcontig04979:9447701	9447701	9448384	684	1	0.000485405	15	2.192982456	NA	NA
DMRcontig04988:9463001	9463001	9463400	400	1	1.62E-06	20	5	BRAFLDRAFT_120096	epigenetic
DMRcontig05000:9483192	9483192	9483500	309	1	0.000905021	20	6.472491909	NA	NA
DMRcontig05029:9532001	9532001	9532480	480	4	0.000247076	13	2.708333333	NA	NA
DMRcontig05034:9540201	9540201	9540374	174	2	0.000327144	3	1.724137931	NA	NA
DMRcontig05052:9571002	9571002	9572000	999	1	0.000454151	20	2.002002002	NA	NA
DMRcontig05063:9589501	9589501	9590100	600	3	0.000442724	8	1.333333333	NA	NA
DMRcontig05069:9601001	9601001	9601500	500	1	0.000216176	8	1.6	NA	NA
DMRcontig05079:9619201	9619201	9619427	227	1	0.000888393	17	7.488986784	NA	NA
DMRcontig05096:9648236	9648236	9649100	865	5	0.000127014	31	3.583815029	NA	NA
DMRcontig05106:9666101	9666101	9666800	700	1	0.00039323	14	2	NA	NA
DMRcontig05110:9674401	9674401	9674900	500	2	7.75E-05	37	7.4	LOC106602896;EIF4A1	translation
DMRcontig05113:9679901	9679901	9680300	400	2	7.95E-06	10	2.5	NA	NA
DMRcontig05126:9703201	9703201	9704000	800	1	0.00053806	48	6	NA	NA
DMRcontig05127:9705016	9705016	9705600	585	1	1.11E-05	8	1.367521368	NA	NA

DMRcontig05132:9713401	9713401	9713800	400	1	0.000981487	6	1.5	NA	NA
DMRcontig05146:9737301	9737301	9737400	100	1	0.000197053	5	5	NA	NA
DMRcontig05151:9746425	9746425	9747100	676	1	0.000418462	26	3.846153846	NA	NA
DMRcontig05156:9754512	9754512	9755200	689	3	2.13E-05	24	3.483309144	NA	NA
DMRcontig05163:9766901	9766901	9767100	200	2	0.000235343	9	4.5	atg5	metabolism
DMRcontig05170:9778601	9778601	9778971	371	1	0.000146068	15	4.043126685	NA	NA
DMRcontig05175:9787501	9787501	9787971	471	1	0.000863798	5	1.061571125	NA	NA
DMRcontig05178:9792108	9792108	9792300	193	1	9.38E-05	13	6.735751295	NA	NA
DMRcontig05186:9806001	9806001	9806900	900	1	0.000392832	16	1.777777778	NA	NA
DMRcontig05204:9836801	9836801	9837100	300	2	4.84E-05	8	2.666666667	NA	NA
DMRcontig05210:9847301	9847301	9848031	731	2	0.000259426	52	7.113543092	NA	NA
DMRcontig05216:9856301	9856301	9856600	300	1	0.000675129	15	5	NA	NA
DMRcontig05235:9887262	9887262	9888319	1058	6	1.35E-06	27	2.551984877	NA	NA
DMRcontig05237:9890924	9890924	9891400	477	2	0.000397902	3	0.628930818	NA	NA
DMRcontig05245:9906701	9906701	9907068	368	1	3.51E-05	9	2.445652174	NA	NA
DMRcontig05261:9932401	9932401	9932546	146	1	0.000676338	12	8.219178082	NA	NA
DMRcontig05276:9958601	9958601	9959000	400	1	0.000519002	13	3.25	NA	NA
DMRcontig05280:9965701	9965701	9966000	300	1	2.12E-05	7	2.333333333	NA	NA
DMRcontig05302:10002014	10002014	10002500	487	1	0.000925024	36	7.392197125	NA	NA
DMRcontig05309:10013301	10013301	10013800	500	1	9.64E-06	30	6	NA	NA
DMRcontig05314:10022186	10022186	10022600	415	2	0.000258468	12	2.891566265	NA	NA
DMRcontig05322:10035601	10035601	10037209	1609	2	0.000339266	32	1.988812927	NA	NA
DMRcontig05343:10073201	10073201	10073800	600	2	6.93E-05	9	1.5	NA	NA
DMRcontig05359:10099901	10099901	10100100	200	1	0.000102538	2	1	NA	NA
DMRcontig05363:10106601	10106601	10106800	200	1	0.000325078	1	0.5	NA	NA
DMRcontig05367:10114201	10114201	10114600	400	2	1.33E-05	21	5.25	NA	NA
DMRcontig05378:10132201	10132201	10133100	900	1	0.000748611	42	4.666666667	NA	NA
DMRcontig05449:10251101	10251101	10251529	429	2	2.82E-06	8	1.864801865	NA	NA
DMRcontig05491:10321601	10321601	10322100	500	2	6.58E-06	21	4.2	NA	NA
DMRcontig05494:10326424	10326424	10326700	277	1	7.95E-05	6	2.166064982	NA	NA
DMRcontig05498:10332701	10332701	10333400	700	1	0.000289847	40	5.714285714	NA	NA
DMRcontig05502:10339401	10339401	10340400	1000	4	4.06E-06	31	3.1	NA	NA
DMRcontig05509:10352001	10352001	10353100	1100	2	2.70E-05	39	3.545454545	NA	NA
DMRcontig05532:10391201	10391201	10391500	300	1	0.000241446	12	4	NA	NA
DMRcontig05534:10395101	10395101	10395800	700	1	0.000339509	34	4.857142857	LOC106609801;LOC106609812;LOC106595659;LOC106596913;yeats4	transcription
DMRcontig05538:10402101	10402101	10402502	402	2	1.86E-08	28	6.965174129	NA	NA
DMRcontig05562:10442401	10442401	10443000	600	2	5.66E-07	34	5.666666667	NA	NA
DMRcontig05564:10446001	10446001	10446400	400	1	0.000277771	14	3.5	NA	NA
DMRcontig05572:10461801	10461801	10462000	200	1	0.000458813	11	5.5	NA	NA
DMRcontig05580:10475101	10475101	10475800	700	1	0.00036527	16	2.285714286	NA	NA
DMRcontig05582:10478501	10478501	10478800	300	1	0.000418397	12	4	NA	NA
DMRcontig05583:10480133	10480133	10480600	468	1	0.000714802	20	4.273504274	NA	NA
DMRcontig05597:10502330	10502330	10502800	471	2	8.69E-05	10	2.123142251	NA	NA
DMRcontig05632:10559601	10559601	10560000	400	1	0.000744189	21	5.25	AP1M1	metabolism
DMRcontig05639:10571529	10571529	10572000	472	1	0.00075198	4	0.847457627	NA	NA
DMRcontig05671:10624701	10624701	10625263	563	1	0.000355326	13	2.309058615	NA	NA
DMRcontig05684:10646501	10646501	10646800	300	1	0.000521849	1	0.333333333	NA	NA
DMRcontig05700:10674001	10674001	10674936	936	2	0.000600928	25	2.670940171	NA	NA
DMRcontig05708:10686901	10686901	10687379	479	2	6.59E-06	9	1.878914405	NA	NA
DMRcontig05714:10696837	10696837	10697930	1094	1	4.86E-05	25	2.285191956	NA	NA
DMRcontig05715:10699201	10699201	10699660	460	2	1.96E-05	18	3.913043478	GLOTRDRAFT_130935	miscellaneous
DMRcontig05723:10713201	10713201	10713955	755	3	5.86E-05	24	3.178807947	NA	NA
DMRcontig05738:10737901	10737901	10738723	823	2	2.29E-06	26	3.159173755	NA	NA
DMRcontig05753:10762801	10762801	10763700	900	3	0.000158127	63	7	NA	NA
DMRcontig05764:10782401	10782401	10782800	400	2	0.000133634	7	1.75	NA	NA
DMRcontig05769:10791101	10791101	10791300	200	1	1.13E-05	2	1	NA	NA
DMRcontig05773:10797001	10797001	10797429	429	3	3.58E-05	34	7.925407925	NA	NA
DMRcontig05794:10832701	10832701	10832900	200	2	4.13E-06	2	1	NA	NA
DMRcontig05809:10858101	10858101	10858392	292	1	0.00098368	6	2.054794521	NA	NA
DMRcontig05815:10867801	10867801	10868147	347	1	0.000632759	4	1.152737752	NA	NA
DMRcontig05829:10892301	10892301	10892600	300	1	0.00016694	11	3.666666667	NA	NA
DMRcontig05840:10911401	10911401	10912200	800	1	0.000876723	22	2.75	NA	NA
DMRcontig05842:10915108	10915108	10916300	1193	4	5.57E-06	29	2.430846605	NA	NA
DMRcontig05858:10942601	10942601	10943000	400	1	0.000310391	20	5	Ddx46	transcription
DMRcontig05872:10966501	10966501	10966900	400	2	0.0001903	15	3.75	NA	NA
DMRcontig05874:10969501	10969501	10969700	200	1	0.000918643	2	1	NA	NA
DMRcontig05877:10974501	10974501	10974900	400	1	0.000147691	16	4	copb1	metabolism
DMRcontig05882:10982901	10982901	10983700	800	1	0.000993147	29	3.625	NA	NA
DMRcontig05938:11078601	11078601	11078984	384	1	0.000413221	21	5.46875	NA	NA
DMRcontig05961:11115101	11115101	11115500	400	1	0.000381246	10	2.5	NA	NA
DMRcontig05978:11144101	11144101	11144300	200	1	7.60E-05	0	0	NA	NA
DMRcontig05990:11163801	11163801	11164200	400	1	0.000164618	11	2.75	NA	NA
DMRcontig06076:11308001	11308001	11308500	500	3	1.27E-05	13	2.6	NA	NA
DMRcontig06084:11320701	11320701	11321000	300	1	0.0005905	5	1.666666667	NA	NA
DMRcontig06125:11389953	11389953	11390545	593	1	9.87E-06	20	3.372681282	NA	NA
DMRcontig06133:11402401	11402401	11403000	600	1	0.000346387	9	1.5	NA	NA
DMRcontig06145:11422544	11422544	11423600	1057	1	0.000763362	33	3.122043519	NA	NA
DMRcontig06184:11490501	11490501	11491000	500	2	0.000157187	18	3.6	NA	NA

DMRcontig06187:11495401	11495401	11495900	500	4	6.23E-05	14	2.8	NA	NA
DMRcontig06191:11502901	11502901	11503087	187	1	0.000280602	3	1.604278075	NA	NA
DMRcontig06217:11544001	11544001	11544400	400	2	0.000236286	9	2.25	NA	NA
DMRcontig06221:11550231	11550231	11551600	1370	1	0.000206263	40	2.919708029	NA	NA
DMRcontig06247:11593901	11593901	11594100	200	1	0.000363838	6	3	NA	NA
DMRcontig06287:11661301	11661301	11661981	681	2	2.76E-05	8	1.174743025	LOC105379109	unknown
DMRcontig06312:11703601	11703601	11703900	300	2	0.000158264	19	6.333333333	NA	NA
DMRcontig06324:11724601	11724601	11725647	1047	1	3.93E-05	28	2.674307545	NA	NA
DMRcontig06362:11787101	11787101	11787300	200	1	0.000309534	11	5.5	NA	NA
DMRcontig06377:11812825	11812825	11813800	976	2	8.84E-07	21	2.151639344	NA	NA
DMRcontig06378:11814905	11814905	11815212	308	3	2.55E-05	10	3.246753247	NA	NA
DMRcontig06388:11829701	11829701	11830096	396	2	0.000155914	7	1.767676768	NA	NA
DMRcontig06392:11836301	11836301	11836419	119	1	0.000330643	9	7.56302521	NA	NA
DMRcontig06417:11876019	11876019	11876200	182	2	0.000775612	4	2.197802198	NA	NA
DMRcontig06424:11887601	11887601	11887800	200	1	0.000981487	7	3.5	NA	NA
DMRcontig06433:11902287	11902287	11903000	714	1	0.000699323	20	2.801120448	NA	NA
DMRcontig06436:11907101	11907101	11907700	600	3	2.15E-05	26	4.333333333	NA	NA
DMRcontig06442:11918301	11918301	11918600	300	3	9.95E-07	10	3.333333333	NA	NA
DMRcontig06476:11975030	11975030	11975200	171	1	0.000413924	10	5.847953216	NA	NA
DMRcontig06482:11985201	11985201	11985900	700	1	0.000940665	12	1.714285714	NA	NA
DMRcontig06486:11992301	11992301	11992576	276	1	0.000892659	19	6.884057971	NA	NA
DMRcontig06504:12021865	12021865	12022900	1036	3	2.01E-10	73	7.046332046	NA	NA
DMRcontig06510:12032382	12032382	12032900	519	2	2.69E-05	16	3.082851638	NA	NA
DMRcontig06512:12035501	12035501	12036700	1200	4	6.64E-05	31	2.583333333	NA	NA
DMRcontig06517:12044101	12044101	12044500	400	2	1.38E-05	17	4.25	NA	NA
DMRcontig06529:12064201	12064201	12064700	500	2	0.000596127	22	4.4	NA	NA
DMRcontig06536:12075401	12075401	12075700	300	1	3.18E-05	5	1.666666667	NA	NA
DMRcontig06572:12134407	12134407	12134900	494	3	5.05E-06	17	3.441295547	NA	NA
DMRcontig06581:12148001	12148001	12148770	770	1	0.000528005	24	3.116883117	NA	NA
DMRcontig06583:12151340	12151340	12151500	161	1	1.50E-05	0	0	NA	NA
DMRcontig06587:12158401	12158401	12158840	440	1	0.000253648	23	5.227272727	NA	NA
DMRcontig06588:12159901	12159901	12160200	300	2	9.31E-05	10	3.333333333	NA	NA
DMRcontig06591:12164701	12164701	12164900	200	2	4.64E-08	12	6	NA	NA
DMRcontig06599:12178601	12178601	12179320	720	1	2.38E-06	44	6.111111111	NA	NA
DMRcontig06603:12185564	12185564	12186000	437	1	0.000306392	10	2.288329519	NA	NA
DMRcontig06605:12188701	12188701	12188895	195	1	0.000454051	7	3.58974359	NA	NA
DMRcontig06623:12217448	12217448	12217600	153	1	0.000169659	3	1.960784314	NA	NA
DMRcontig06626:12221701	12221701	12222606	906	1	0.000139821	55	6.070640177	LOC105398638;hspa5	signaling
DMRcontig06637:12240401	12240401	12241100	700	1	0.000195717	24	3.428571429	NA	NA
DMRcontig06649:12260901	12260901	12261100	200	2	1.70E-06	13	6.5	NA	NA
DMRcontig06655:12269801	12269801	12270400	600	1	0.000670739	20	3.333333333	NA	NA
DMRcontig06660:12277901	12277901	12278100	200	1	0.000487247	9	4.5	NA	NA
DMRcontig06666:12286901	12286901	12287471	571	4	4.99E-05	26	4.553415061	NA	NA
DMRcontig06669:12291425	12291425	12291700	276	1	0.000955121	11	3.985507246	NA	NA
DMRcontig06672:12296607	12296607	12297300	694	1	0.000105358	34	4.899135447	NA	NA
DMRcontig06703:12348108	12348108	12348600	493	1	0.000238372	10	2.028397566	NA	NA
DMRcontig06717:12371201	12371201	12371900	700	1	0.00068347	24	3.428571429	UCRNP2_6158	miscellaneous
DMRcontig06739:12407201	12407201	12407490	290	2	5.76E-05	9	3.103448276	NA	NA
DMRcontig06740:12408501	12408501	12408800	300	2	0.000115085	8	2.666666667	NA	NA
DMRcontig06806:12515301	12515301	12515624	324	1	0.000933613	4	1.234567901	NA	NA
DMRcontig06808:12518292	12518292	12518900	609	1	3.73E-05	33	5.418719212	NA	NA
DMRcontig06816:12533201	12533201	12533400	200	1	2.69E-05	7	3.5	NA	NA
DMRcontig06824:12546310	12546310	12546500	191	1	4.88E-05	4	2.094240838	NA	NA
DMRcontig06837:12567184	12567184	12567691	508	1	0.000696712	7	1.377952756	NA	NA
DMRcontig06845:12579601	12579601	12579800	200	1	6.88E-06	18	9	NA	NA
DMRcontig06879:12635301	12635301	12635673	373	2	0.00027266	35	9.383378016	NA	NA
DMRcontig06886:12646401	12646401	12647500	1100	3	3.92E-06	19	1.727272727	NA	NA
DMRcontig06913:12690801	12690801	12691000	200	1	0.000806927	4	2	NA	NA
DMRcontig06916:12695801	12695801	12696814	1014	3	3.87E-06	27	2.662721893	NA	NA
DMRcontig06956:12760401	12760401	12761300	900	2	0.000913768	19	2.111111111	NA	NA
DMRcontig06975:12791701	12791701	12791900	200	1	0.000495626	3	1.5	NA	NA
DMRcontig06981:12801801	12801801	12802437	637	1	0.000412227	27	4.238618524	NA	NA
DMRcontig07017:12861901	12861901	12862947	1047	1	3.86E-05	37	3.533906399	NA	NA
DMRcontig07024:12874101	12874101	12874800	700	1	0.000332119	23	3.285714286	LOC106600329	unknown
DMRcontig07036:12892901	12892901	12893100	200	2	5.65E-05	3	1.5	NA	NA
DMRcontig07061:12932701	12932701	12933473	773	2	2.55E-05	12	1.552393273	UPF3B	development
DMRcontig07108:13010801	13010801	13011693	893	3	0.000307111	20	2.239641657	NA	NA
DMRcontig07109:13013101	13013101	13013300	200	2	1.91E-05	17	8.5	NA	NA
DMRcontig07114:13021001	13021001	13022000	1000	1	0.000530106	49	4.9	NA	NA
DMRcontig07119:13030301	13030301	13030546	246	1	0.000726243	6	2.43902439	NA	NA
DMRcontig07130:13047001	13047001	13047400	400	1	0.000860806	23	5.75	NA	NA
DMRcontig07158:13092901	13092901	13093263	363	1	6.04E-05	4	1.101928375	NA	NA
DMRcontig07163:13101101	13101101	13101400	300	1	0.000201617	11	3.666666667	NA	NA
DMRcontig07177:13122401	13122401	13122900	500	1	0.00076845	5	1	NA	NA
DMRcontig07185:13135401	13135401	13135700	300	1	5.07E-05	13	4.333333333	NA	NA
DMRcontig07187:13138601	13138601	13139400	800	6	7.16E-09	31	3.875	NA	NA
DMRcontig07197:13155211	13155211	13155800	590	2	2.44E-05	35	5.93220339	NA	NA
DMRcontig07203:13165101	13165101	13165292	192	1	0.000315271	3	1.5625	NA	NA
DMRcontig07222:13194921	13194921	13195000	80	1	0.000701691	0	0	NA	NA
DMRcontig07225:13199701	13199701	13199900	200	1	0.000193323	5	2.5	NA	NA

DMRcontig07229:13206701	13206701	13206800	100	1	4.04E-05	5	5	NA	NA
DMRcontig07232:13211401	13211401	13211700	300	1	0.000172184	13	4.333333333	NA	NA
DMRcontig07244:13229701	13229701	13230000	300	1	0.000694204	18	6	NA	NA
DMRcontig07268:13269301	13269301	13269768	468	2	1.03E-05	22	4.700854701	NA	NA
DMRcontig07305:13328201	13328201	13328600	400	1	5.53E-05	20	5	ass1	development
DMRcontig07330:13367301	13367301	13367600	300	1	0.000588462	8	2.666666667	NA	NA
DMRcontig07334:13373701	13373701	13374046	346	1	0.000505846	9	2.601156069	NA	NA
DMRcontig07346:13392419	13392419	13392700	282	2	3.86E-05	8	2.836879433	NA	NA
DMRcontig07349:13397059	13397059	13397500	442	3	0.000388798	8	1.809954751	NA	NA
DMRcontig07372:13434501	13434501	13434645	145	1	0.000372541	14	9.655172414	LOC587178	development
DMRcontig07382:13451701	13451701	13451800	100	1	0.00098	1	1	NA	NA
DMRcontig07393:13468101	13468101	13468400	300	1	0.000132147	7	2.333333333	NA	NA
DMRcontig07413:13500301	13500301	13500763	463	1	0.000903753	14	3.023758099	NA	NA
DMRcontig07416:13505001	13505001	13505800	800	2	7.14E-06	12	1.5	NA	NA
DMRcontig07434:13533601	13533601	13534000	400	1	5.21E-06	10	2.5	NA	NA
DMRcontig07447:13553603	13553603	13554000	398	2	1.64E-05	4	1.005025126	NA	NA
DMRcontig07448:13555501	13555501	13555900	400	1	0.000412084	8	2	NA	NA
DMRcontig07462:13578501	13578501	13579000	500	2	1.44E-05	12	2.4	NA	NA
DMRcontig07463:13580167	13580167	13580400	234	1	0.000612787	6	2.564102564	NA	NA
DMRcontig07468:13587191	13587191	13587839	649	1	0.000195265	38	5.855161787	NA	NA
DMRcontig07469:13589201	13589201	13589325	125	1	0.000769914	4	3.2	NA	NA
DMRcontig07485:13613201	13613201	13613750	550	1	0.000376847	17	3.090909091	NA	NA
DMRcontig07497:13632801	13632801	13632900	100	1	3.29E-05	6	6	LOC101862740;RAB8A	signaling
DMRcontig07510:13655001	13655001	13655500	500	1	0.000972023	14	2.8	NA	NA
DMRcontig07530:13687001	13687001	13687500	500	1	2.12E-05	15	3	NA	NA
DMRcontig07537:13698601	13698601	13699000	400	3	1.82E-06	8	2	NA	NA
DMRcontig07544:13709145	13709145	13709686	542	3	6.07E-07	25	4.612546125	NA	NA
DMRcontig07581:13769480	13769480	13769900	421	2	8.52E-06	12	2.850356295	NA	NA
DMRcontig07587:13778801	13778801	13779100	300	2	1.54E-05	8	2.666666667	NA	NA
DMRcontig07595:13791601	13791601	13792100	500	2	0.000330421	7	1.4	NA	NA
DMRcontig07598:13797001	13797001	13797700	700	1	2.33E-05	45	6.428571429	MNS1	development
DMRcontig07612:13821301	13821301	13821500	200	1	0.000645547	5	2.5	NA	NA
DMRcontig07615:13825431	13825431	13825900	470	1	6.73E-05	19	4.042553191	NA	NA
DMRcontig07622:13837201	13837201	13837369	169	2	0.000358298	9	5.325443787	NA	NA
DMRcontig07643:13869501	13869501	13869878	378	1	0.000100817	13	3.439153439	NA	NA
DMRcontig07647:13877001	13877001	13877200	200	1	0.000402059	9	4.5	NA	NA
DMRcontig07648:13878701	13878701	13878798	98	1	0.000158264	7	7.142857143	NA	NA
DMRcontig07651:13882537	13882537	13882800	264	1	5.38E-05	11	4.166666667	NA	NA
DMRcontig07656:13890201	13890201	13891296	1096	1	0.000248738	45	4.105839416	LOC102100396;ddx39a	translation
DMRcontig07662:13900301	13900301	13900600	300	2	4.20E-05	12	4	NA	NA
DMRcontig07680:13931801	13931801	13932735	935	1	5.24E-05	12	1.28342246	NA	NA
DMRcontig07687:13943529	13943529	13943800	272	3	2.81E-05	3	1.102941176	NA	NA
DMRcontig07711:13982501	13982501	13982988	488	2	8.54E-07	4	0.819672131	NA	NA
DMRcontig07714:13987701	13987701	13988500	800	2	2.07E-05	45	5.625	NA	NA
DMRcontig07721:13998634	13998634	13998800	167	1	0.000137109	10	5.988023952	NA	NA
DMRcontig07732:14016165	14016165	14016500	336	2	0.000421706	27	8.035714286	NA	NA
DMRcontig07779:14093801	14093801	14094500	700	1	3.24E-06	23	3.285714286	NA	NA
DMRcontig07815:14152301	14152301	14152800	500	1	0.000771593	21	4.2	NA	NA
DMRcontig07825:14169301	14169301	14169900	600	4	0.0001157	12	2	COX1;ND3	metabolism and transport
DMRcontig07826:14171301	14171301	14171600	300	1	4.25E-05	17	5.666666667	LOC107094001	metabolism
DMRcontig07832:14180701	14180701	14181000	300	1	0.000286268	7	2.333333333	NA	NA
DMRcontig07833:14182401	14182401	14182800	400	2	7.09E-05	10	2.5	NA	NA
DMRcontig07840:14193601	14193601	14194223	623	2	0.000552247	9	1.444622793	LOC103241789	protease
DMRcontig07851:14210943	14210943	14211800	858	1	0.000207652	19	2.214452214	NA	NA
DMRcontig07861:14227811	14227811	14228000	190	2	0.000357448	3	1.578947368	NA	NA
DMRcontig07862:14229501	14229501	14230114	614	2	0.000141993	4	0.651465798	NA	NA
DMRcontig07917:14317301	14317301	14317700	400	1	5.82E-05	15	3.75	NA	NA
DMRcontig07922:14325001	14325001	14325300	300	2	0.000223113	17	5.666666667	NA	NA
DMRcontig07932:14339830	14339830	14340600	771	1	0.000607537	21	2.723735409	NA	NA
DMRcontig07945:14359750	14359750	14359900	151	1	0.000529536	7	4.635761589	NA	NA
DMRcontig07947:14364001	14364001	14364338	338	1	0.000309573	15	4.437869822	NA	NA
DMRcontig07957:14381313	14381313	14381700	388	1	5.58E-05	14	3.608247423	NA	NA
DMRcontig07963:14391601	14391601	14392800	1200	1	0.000144158	27	2.25	NA	NA
DMRcontig07968:14399901	14399901	14400200	300	1	0.000101678	4	1.333333333	NA	NA
DMRcontig07993:14440001	14440001	14440434	434	1	0.000196176	21	4.838709677	NA	NA
DMRcontig08005:14458765	14458765	14459200	436	2	4.57E-05	7	1.605504587	NA	NA
DMRcontig08012:14470552	14470552	14471100	549	2	0.00055403	13	2.367941712	NA	NA
DMRcontig08026:14493486	14493486	14494109	624	1	0.000188973	13	2.083333333	NA	NA
DMRcontig08027:14495201	14495201	14495600	400	4	4.21E-09	37	9.25	NA	NA
DMRcontig08029:14498301	14498301	14498900	600	1	0.000263512	5	0.833333333	NA	NA
DMRcontig08088:14593001	14593001	14593300	300	1	0.000721526	4	1.333333333	NA	NA
DMRcontig08096:14606101	14606101	14606500	400	1	0.000832627	15	3.75	NA	NA
DMRcontig08098:14609201	14609201	14609700	500	1	0.000437134	20	4	NA	NA
DMRcontig08123:14647901	14647901	14648100	200	1	0.000827954	9	4.5	NA	NA
DMRcontig08165:14713501	14713501	14714200	700	2	0.000385584	18	2.571428571	NA	NA
DMRcontig08166:14715301	14715301	14715600	300	1	0.000733891	20	6.666666667	NA	NA
DMRcontig08172:14727501	14727501	14728000	500	1	8.72E-05	9	1.8	NA	NA
DMRcontig08185:14747801	14747801	14748000	200	2	0.000229236	11	5.5	NA	NA
DMRcontig08206:14781330	14781330	14781600	271	2	3.62E-06	12	4.42804428	NA	NA
DMRcontig08213:14793401	14793401	14793898	498	1	0.000391187	3	0.602409639	NA	NA

DMRcontig08222:14808601	14808601	14808800	200	1	0.000365392	5	2.5	NA	NA
DMRcontig08258:14865001	14865001	14865500	500	1	0.000514577	12	2.4	LOC106808264	development
DMRcontig08286:14912401	14912401	14912600	200	1	0.000187348	9	4.5	NA	NA
DMRcontig08290:14919401	14919401	14919500	100	1	0.000419945	2	2	NA	NA
DMRcontig08321:14967462	14967462	14968040	579	1	0.000239545	6	1.03626943	NA	NA
DMRcontig08324:14972601	14972601	14972900	300	1	0.000999551	8	2.666666667	VDAG_02253	dna repair
DMRcontig08328:14979301	14979301	14979600	300	1	0.000486424	5	1.666666667	LOC105632124	development
DMRcontig08329:14980801	14980801	14981600	800	2	0.000229707	23	2.875	NA	NA
DMRcontig08339:14997801	14997801	14998270	470	1	0.000381624	14	2.978723404	NA	NA
DMRcontig08357:15026601	15026601	15026900	300	1	4.51E-05	12	4	NA	NA
DMRcontig08377:15058601	15058601	15059100	500	1	0.000797273	11	2.2	NA	NA
DMRcontig08379:15062601	15062601	15062824	224	1	0.000239689	16	7.142857143	NA	NA
DMRcontig08417:15123401	15123401	15123600	200	1	0.000495376	2	1	NA	NA
DMRcontig08442:15163901	15163901	15164481	581	1	2.01E-05	22	3.786574871	NA	NA
DMRcontig08447:15172822	15172822	15173400	579	2	0.000817169	23	3.972366149	NA	NA
DMRcontig08448:15174901	15174901	15175100	200	1	7.87E-05	2	1	NA	NA
DMRcontig08449:15176801	15176801	15176969	169	1	2.93E-05	7	4.142011834	NA	NA
DMRcontig08475:15216657	15216657	15217000	344	3	8.64E-06	11	3.197674419	PHSY_005264;PPP1CA	miscellaneous
DMRcontig08477:15219801	15219801	15220500	700	1	0.000774046	8	1.142857143	NA	NA
DMRcontig08488:15237201	15237201	15237700	500	2	0.000640076	20	4	NA	NA
DMRcontig08505:15263711	15263711	15264100	390	1	0.000275447	9	2.307692308	NA	NA
DMRcontig08515:15278503	15278503	15278800	298	2	0.000272429	22	7.382550336	NA	NA
DMRcontig08523:15290837	15290837	15291200	364	1	0.000670862	6	1.648351648	NA	NA
DMRcontig08538:15316101	15316101	15316355	255	1	0.000650711	4	1.568627451	NA	NA
DMRcontig08555:15342601	15342601	15342700	100	1	0.00048317	5	5	NA	NA
DMRcontig08571:15368801	15368801	15369500	700	1	4.76E-05	45	6.428571429	NA	NA
DMRcontig08583:15388027	15388027	15388300	274	2	2.44E-05	12	4.379562044	NA	NA
DMRcontig08588:15396601	15396601	15397031	431	4	2.19E-06	15	3.480278422	NA	NA
DMRcontig08602:15418701	15418701	15419048	348	2	7.04E-05	15	4.310344828	NA	NA
DMRcontig08616:15441301	15441301	15441700	400	1	1.39E-05	6	1.5	NA	NA
DMRcontig08670:15528401	15528401	15528800	400	1	0.000588625	12	3	NA	NA
DMRcontig08684:15550701	15550701	15550977	277	1	0.000372508	1	0.36101083	NA	NA
DMRcontig08713:15597201	15597201	15597500	300	1	0.000328537	5	1.666666667	NA	NA
DMRcontig08729:15621501	15621501	15622000	500	2	1.23E-06	21	4.2	NA	NA
DMRcontig08752:15657301	15657301	15657597	297	2	0.00049602	8	2.693602694	NA	NA
DMRcontig08783:15707169	15707169	15708500	1332	3	9.45E-08	36	2.702702703	NA	NA
DMRcontig08787:15713944	15713944	15714500	557	2	4.02E-06	20	3.590664273	NA	NA
DMRcontig08791:15720501	15720501	15721100	600	1	0.000304944	11	1.833333333	NA	NA
DMRcontig08802:15737801	15737801	15738026	226	1	8.47E-05	13	5.752212389	LOC101857837;AP1B1	development
DMRcontig08820:15764434	15764434	15764800	367	1	0.000394307	7	1.907356948	NA	NA
DMRcontig08832:15782901	15782901	15783700	800	1	0.00023916	14	1.75	NA	NA
DMRcontig08837:15791101	15791101	15791572	472	1	2.45E-05	7	1.483050847	NA	NA
DMRcontig08850:15811171	15811171	15811591	421	2	0.000283906	9	2.137767221	NA	NA
DMRcontig08869:15841001	15841001	15841600	600	1	0.000249283	15	2.5	NA	NA
DMRcontig08896:15884401	15884401	15885200	800	1	0.000238868	32	4	SPRG_12594	cytoskeleton
DMRcontig08912:15909301	15909301	15909500	200	1	0.000679818	3	1.5	NA	NA
DMRcontig08913:15910701	15910701	15911279	579	5	5.88E-07	14	2.417962003	NA	NA
DMRcontig08919:15920401	15920401	15920900	500	1	2.75E-05	18	3.6	NA	NA
DMRcontig08937:15949701	15949701	15950200	500	1	7.49E-05	9	1.8	NA	NA
DMRcontig08953:15975301	15975301	15975400	100	1	1.69E-06	3	3	NA	NA
DMRcontig08959:15984501	15984501	15985200	700	2	7.11E-05	11	1.571428571	NA	NA
DMRcontig09006:16057701	16057701	16058000	300	1	0.000862153	17	5.666666667	NA	NA
DMRcontig09019:16077301	16077301	16078000	700	3	6.78E-08	21	3	NA	NA
DMRcontig09055:16135501	16135501	16135800	300	1	9.46E-05	4	1.333333333	NA	NA
DMRcontig09067:16152956	16152956	16153200	245	1	0.000887811	4	1.632653061	NA	NA
DMRcontig09069:16155781	16155781	16156200	420	1	0.000311208	24	5.714285714	NA	NA
DMRcontig09080:16173101	16173101	16173440	340	2	4.32E-05	14	4.117647059	NA	NA
DMRcontig09086:16182201	16182201	16182664	464	1	0.000133417	31	6.681034483	NA	NA
DMRcontig09087:16184001	16184001	16184368	368	2	4.53E-05	6	1.630434783	NA	NA
DMRcontig09108:16218101	16218101	16218200	100	1	0.000955121	1	1	NA	NA
DMRcontig09120:16238801	16238801	16238947	147	1	0.000990831	5	3.401360544	NA	NA
DMRcontig09125:16246301	16246301	16246435	135	1	0.00033102	6	4.444444444	NA	NA
DMRcontig09127:16249127	16249127	16249570	444	2	5.30E-05	12	2.702702703	NA	NA
DMRcontig09129:16252401	16252401	16252900	500	3	3.44E-07	33	6.6	CpipJ_CPIJ017745;FAXDC2	metabolism
DMRcontig09137:16265548	16265548	16265700	153	1	0.000679428	5	3.267973856	NA	NA
DMRcontig09139:16268435	16268435	16269000	566	1	0.000270104	28	4.946996466	NA	NA
DMRcontig09151:16288901	16288901	16289200	300	1	0.00016831	10	3.333333333	NA	NA
DMRcontig09153:16292301	16292301	16292867	567	5	0.000206431	37	6.525573192	NA	NA
DMRcontig09163:16308301	16308301	16308700	400	1	0.000347637	12	3	NA	NA
DMRcontig09168:16315977	16315977	16316400	424	1	0.000335657	9	2.122641509	NA	NA
DMRcontig09178:16332001	16332001	16332300	300	1	3.40E-05	11	3.666666667	LOTGIDRAFT_132224	unknown
DMRcontig09211:16383001	16383001	16383600	600	1	0.000599536	28	4.666666667	NA	NA
DMRcontig09212:16384682	16384682	16385599	918	9	1.72E-10	78	8.496732026	NA	NA
DMRcontig09216:16391501	16391501	16391600	100	1	0.000981487	1	1	NA	NA
DMRcontig09219:16395701	16395701	16396200	500	2	2.13E-05	25	5	NA	NA
DMRcontig09220:16397501	16397501	16398100	600	3	6.65E-05	19	3.166666667	NA	NA
DMRcontig09223:16402000	16402000	16402343	344	1	0.000826376	13	3.779069767	NA	NA
DMRcontig09228:16409674	16409674	16410200	527	1	4.53E-05	21	3.984819734	NA	NA
DMRcontig09236:16422101	16422101	16422412	312	1	0.000349113	17	5.448717949	NA	NA
DMRcontig09242:16430901	16430901	16431296	396	1	5.70E-05	5	1.262626263	si:dkey-31f5.8	miscellaneous

DMRcontig09271:16474601	16474601	16474800	200	1	0.000585134	3	1.5	NA	NA
DMRcontig09278:16485101	16485101	16486000	900	1	0.000106972	41	4.555555556	NA	NA
DMRcontig09280:16488701	16488701	16489200	500	2	8.12E-05	4	0.8	NA	NA
DMRcontig09294:16511601	16511601	16511800	200	1	0.000521849	4	2	NA	NA
DMRcontig09305:16529101	16529101	16529500	400	2	0.000176876	5	1.25	NA	NA
DMRcontig09309:16535022	16535022	16535400	379	1	0.000981487	7	1.846965699	NA	NA
DMRcontig09310:16536601	16536601	16536800	200	1	3.30E-05	9	4.5	NA	NA
DMRcontig09319:16550201	16550201	16550538	338	1	0.000950925	20	5.917159763	NA	NA
DMRcontig09322:16554507	16554507	16554900	394	1	0.000457636	7	1.776649746	NA	NA
DMRcontig09386:16658601	16658601	16659000	400	1	0.00048588	23	5.75	NA	NA
DMRcontig09394:16670701	16670701	16671500	800	1	0.000335182	34	4.25	NA	NA
DMRcontig09434:16733501	16733501	16733870	370	2	0.000163069	3	0.810810811	NA	NA
DMRcontig09440:16742701	16742701	16742800	100	1	2.25E-05	3	3	NA	NA
DMRcontig09444:16749001	16749001	16749798	798	2	0.000673098	25	3.13283208	NA	NA
DMRcontig09450:16759001	16759001	16759300	300	1	0.00072745	9	3	NA	NA
DMRcontig09458:16771017	16771017	16771556	540	1	0.000540859	7	1.296296296	NA	NA
DMRcontig09468:16787004	16787004	16787200	197	1	0.000336953	4	2.030456853	NA	NA
DMRcontig09481:16806801	16806801	16807100	300	1	0.000580663	7	2.333333333	NA	NA
DMRcontig09484:16811208	16811208	16812000	793	2	0.000550744	40	5.044136192	NA	NA
DMRcontig09526:16877166	16877166	16877800	635	1	0.000981487	18	2.834645669	NA	NA
DMRcontig09530:16883032	16883032	16883500	469	1	0.000241141	20	4.264392324	NA	NA
DMRcontig09547:16909669	16909669	16910245	577	5	7.60E-08	28	4.852686308	NA	NA
DMRcontig09562:16933070	16933070	16933600	531	1	0.000892535	15	2.824858757	NA	NA
DMRcontig09570:16945429	16945429	16945700	272	1	0.000146081	8	2.941176471	NA	NA
DMRcontig09579:16959556	16959556	16959800	245	2	0.000127092	9	3.673469388	NA	NA
DMRcontig09584:16967401	16967401	16968100	700	1	0.000262373	39	5.571428571	NA	NA
DMRcontig09586:16971019	16971019	16971565	547	4	4.32E-07	20	3.65630713	NA	NA
DMRcontig09618:17022801	17022801	17023100	300	1	7.90E-05	19	6.333333333	NA	NA
DMRcontig09632:17044963	17044963	17045472	510	2	3.38E-05	12	2.352941176	NA	NA
DMRcontig09634:17047910	17047910	17048400	491	2	2.77E-06	26	5.295315682	NA	NA
DMRcontig09649:17070965	17070965	17071600	636	1	0.000676751	26	4.088050314	NA	NA
DMRcontig09660:17087701	17087701	17088234	534	2	6.10E-06	18	3.370786517	LOC106070317	unknown
DMRcontig09661:17089301	17089301	17089500	200	1	0.000637073	4	2	NA	NA
DMRcontig09665:17095841	17095841	17096296	456	2	0.000172836	43	9.429824561	NA	NA
DMRcontig09666:17097301	17097301	17098100	800	1	0.000394242	14	1.75	NA	NA
DMRcontig09685:17126987	17126987	17127400	414	1	0.000440087	16	3.8647343	NA	NA
DMRcontig09690:17136601	17136601	17137239	639	2	0.000537816	14	2.190923318	NA	NA
DMRcontig09692:17139612	17139612	17139800	189	1	7.30E-05	12	6.349206349	NA	NA
DMRcontig09698:17149401	17149401	17149575	175	1	0.000308707	18	10.28571429	NA	NA
DMRcontig09699:17151001	17151001	17151249	249	1	1.80E-05	3	1.204819277	NA	NA
DMRcontig09725:17190201	17190201	17190500	300	2	4.32E-05	11	3.666666667	NA	NA
DMRcontig09737:17209101	17209101	17209300	200	1	0.000209203	3	1.5	NA	NA
DMRcontig09746:17224051	17224051	17224381	331	2	0.000317306	3	0.906344411	NA	NA
DMRcontig09747:17225501	17225501	17225812	312	1	0.000799033	4	1.282051282	NA	NA
DMRcontig09761:17247738	17247738	17248100	363	2	0.000148811	23	6.336088154	NA	NA
DMRcontig09766:17255507	17255507	17255900	394	1	0.000515301	8	2.030456853	NA	NA
DMRcontig09778:17274101	17274101	17274300	200	1	0.000138334	11	5.5	NA	NA
DMRcontig09780:17277501	17277501	17277700	200	2	0.000338813	6	3	NA	NA
DMRcontig09785:17285519	17285519	17286071	553	3	5.51E-05	10	1.808318264	NA	NA
DMRcontig09837:17366676	17366676	17366900	225	1	0.000921505	2	0.888888889	NA	NA
DMRcontig09844:17378301	17378301	17378466	166	2	0.000640885	4	2.409638554	NA	NA
DMRcontig09860:17403001	17403001	17403288	288	2	1.15E-05	15	5.208333333	NA	NA
DMRcontig09894:17453101	17453101	17453300	200	2	5.69E-07	7	3.5	NA	NA
DMRcontig09899:17460248	17460248	17460730	483	2	0.000347478	9	1.863354037	NA	NA
DMRcontig09922:17494701	17494701	17495200	500	2	0.000205655	14	2.8	NA	NA
DMRcontig09937:17517301	17517301	17517751	451	2	8.44E-05	14	3.10421286	NA	NA
DMRcontig09949:17536001	17536001	17536564	564	2	0.000125911	11	1.95035461	NA	NA
DMRcontig09950:17537565	17537565	17537800	236	1	0.00015825	4	1.694915254	fam222a	miscellaneous
DMRcontig09960:17553213	17553213	17553400	188	1	0.000409006	8	4.255319149	NA	NA
DMRcontig09973:17573501	17573501	17573800	300	1	4.71E-05	18	6	NA	NA
DMRcontig09975:17577301	17577301	17577600	300	1	0.000366056	11	3.666666667	NA	NA
DMRcontig09976:17578801	17578801	17579300	500	1	0.000413403	12	2.4	NA	NA
DMRcontig09988:17598701	17598701	17598900	200	1	0.000810816	5	2.5	NA	NA
DMRcontig09993:17606224	17606224	17606700	477	1	0.000536361	38	7.966457023	NA	NA
DMRcontig10018:17644201	17644201	17644700	500	1	0.000362139	12	2.4	NA	NA
DMRcontig10024:17654354	17654354	17654500	147	1	0.00022444	6	4.081632653	NA	NA
DMRcontig10035:17672046	17672046	17672400	355	1	0.000385045	13	3.661971831	NA	NA
DMRcontig10042:17683024	17683024	17683474	451	1	6.72E-05	10	2.2172949	NA	NA
DMRcontig10077:17737101	17737101	17737200	100	1	0.000834158	3	3	BRAFLDRAFT_290865;ALDH1L1	miscellaneous
DMRcontig10104:17778301	17778301	17778500	200	2	2.61E-05	4	2	NA	NA
DMRcontig10126:17812239	17812239	17812600	362	2	0.000118866	12	3.314917127	NA	NA
DMRcontig10142:17838201	17838201	17838837	637	1	0.0003588	9	1.412872841	NA	NA
DMRcontig10148:17847301	17847301	17848000	700	2	5.41E-05	8	1.142857143	NA	NA
DMRcontig10171:17883701	17883701	17884135	435	1	0.000833067	9	2.068965517	NA	NA
DMRcontig10188:17909901	17909901	17910400	500	2	0.00026975	21	4.2	NA	NA
DMRcontig10191:17914201	17914201	17915037	837	2	0.000154916	39	4.659498208	NA	NA
DMRcontig10194:17919101	17919101	17919400	300	1	0.000481206	18	6	NA	NA
DMRcontig10206:17937801	17937801	17938000	200	1	2.09E-05	0	0	NA	NA
DMRcontig10208:17940501	17940501	17940700	200	1	0.000592037	11	5.5	NA	NA
DMRcontig10213:17947807	17947807	17948800	994	3	8.11E-05	29	2.91750503	NA	NA

DMRcontig10221:17960301	17960301	17960866	566	1	8.28E-06	12	2.120141343	NA	NA
DMRcontig10225:17967101	17967101	17967500	400	1	0.000353536	20	5	NA	NA
DMRcontig10234:17980601	17980601	17980900	300	1	0.000286036	4	1.333333333	NA	NA
DMRcontig10235:17982001	17982001	17982400	400	1	0.000320756	12	3	NA	NA
DMRcontig10250:18004901	18004901	18005350	450	3	0.000385237	18	4	NA	NA
DMRcontig10260:18020401	18020401	18020500	100	1	4.60E-05	2	2	LOC105375416	unknown
DMRcontig10273:18040401	18040401	18040800	400	1	0.000194839	6	1.5	NA	NA
DMRcontig10288:18062388	18062388	18062700	313	1	7.04E-05	15	4.792332268	NA	NA
DMRcontig10293:18070532	18070532	18071700	1169	4	3.73E-05	39	3.336184773	NA	NA
DMRcontig10295:18074101	18074101	18074200	100	1	0.000541745	3	3	NA	NA
DMRcontig10320:18110701	18110701	18110900	200	1	0.000466534	6	3	NA	NA
DMRcontig10339:18139501	18139501	18139761	261	1	6.68E-05	5	1.915708812	NA	NA
DMRcontig10348:18152801	18152801	18153000	200	1	7.70E-05	14	7	NA	NA
DMRcontig10354:18162801	18162801	18163000	200	1	0.000987031	8	4	NA	NA
DMRcontig10355:18164163	18164163	18164500	338	1	5.26E-05	5	1.479289941	NA	NA
DMRcontig10361:18173701	18173701	18174100	400	1	0.000157352	14	3.5	NA	NA
DMRcontig10362:18175601	18175601	18176389	789	2	0.000704385	16	2.027883397	NA	NA
DMRcontig10375:18194901	18194901	18195400	500	1	0.000419437	13	2.6	NA	NA
DMRcontig10422:18266401	18266401	18267300	900	2	4.64E-05	48	5.333333333	BRAFLDRAFT_283521;psmd14	miscellaneous
DMRcontig10441:18295001	18295001	18295200	200	1	3.03E-05	9	4.5	LOC101143987;AP1S2	miscellaneous
DMRcontig10452:18312227	18312227	18312921	695	1	6.30E-05	32	4.604316547	LOC106577905;LOC103036640	translation and protein modification
DMRcontig10462:18327901	18327901	18328600	700	1	1.22E-05	14	2	NA	NA
DMRcontig10477:18350901	18350901	18351000	100	1	0.000413521	1	1	NA	NA
DMRcontig10490:18370601	18370601	18370781	181	2	4.48E-05	1	0.552486188	NA	NA
DMRcontig10530:18430512	18430512	18431278	767	1	8.83E-05	17	2.21642764	LOC105924796	mitochondria
DMRcontig10534:18436681	18436681	18437100	420	2	0.000212527	9	2.142857143	NA	NA
DMRcontig10545:18453801	18453801	18454100	300	1	6.50E-05	11	3.666666667	NA	NA
DMRcontig10547:18456701	18456701	18457100	400	2	5.32E-05	19	4.75	NA	NA
DMRcontig10556:18470001	18470001	18470500	500	1	3.46E-06	12	2.4	NA	NA
DMRcontig10560:18476401	18476401	18476900	500	1	0.000381778	26	5.2	LOC107031815	cell cycle
DMRcontig10572:18495613	18495613	18496200	588	2	0.000583334	23	3.911564626	NA	NA
DMRcontig10579:18507001	18507001	18507200	200	2	0.00054444	9	4.5	NA	NA
DMRcontig10591:18525641	18525641	18526300	660	1	0.000411451	6	0.909090909	NA	NA
DMRcontig10638:18599301	18599301	18600643	1343	2	0.000395168	33	2.457185406	NA	NA
DMRcontig10642:18606301	18606301	18606730	430	4	1.22E-05	30	6.976744186	NA	NA
DMRcontig10650:18617433	18617433	18618000	568	2	0.000197228	14	2.464788732	NA	NA
DMRcontig10665:18640361	18640361	18640858	498	1	0.000613869	14	2.81124498	NA	NA
DMRcontig10666:18641901	18641901	18642400	500	1	0.000996436	5	1	NA	NA
DMRcontig10668:18645101	18645101	18645327	227	1	0.000119084	17	7.488986784	NA	NA
DMRcontig10678:18660101	18660101	18660200	100	1	0.000923395	5	5	NA	NA
DMRcontig10706:18703709	18703709	18704000	292	1	0.000291065	5	1.712328767	NA	NA
DMRcontig10725:18733201	18733201	18734032	832	6	5.35E-05	45	5.408653846	NA	NA
DMRcontig10727:18736801	18736801	18737000	200	2	8.11E-05	4	2	NA	NA
DMRcontig10729:18739801	18739801	18740191	391	2	0.000302925	10	2.557544757	NA	NA
DMRcontig10739:18754601	18754601	18754800	200	1	0.000733581	4	2	NA	NA
DMRcontig10764:18793301	18793301	18793700	400	1	0.000305502	9	2.25	NA	NA
DMRcontig10777:18813899	18813899	18814300	402	2	4.92E-05	10	2.487562189	NA	NA
DMRcontig10811:18866101	18866101	18866319	219	3	2.29E-07	6	2.739726027	NA	NA
DMRcontig10815:18871427	18871427	18871800	374	2	0.000239797	19	5.080213904	NA	NA
DMRcontig10822:18881321	18881321	18881754	434	1	2.23E-05	12	2.764976959	NA	NA
DMRcontig10848:18921801	18921801	18922098	298	3	1.57E-05	13	4.362416107	NA	NA
DMRcontig10851:18926301	18926301	18926437	137	1	0.000492614	2	1.459854015	NA	NA
DMRcontig10860:18939864	18939864	18940200	337	2	0.000175695	25	7.418397626	LOC104952122	signaling
DMRcontig10862:18942798	18942798	18943100	303	1	0.00052427	8	2.640264026	NA	NA
DMRcontig10884:18976201	18976201	18976456	256	1	0.000377519	6	2.34375	NA	NA
DMRcontig10893:18989469	18989469	18989900	432	2	0.000604582	9	2.083333333	NA	NA
DMRcontig10900:18999614	18999614	18999800	187	1	2.66E-05	10	5.347593583	NA	NA
DMRcontig10907:19010304	19010304	19010445	142	2	0.000249686	2	1.408450704	NA	NA
DMRcontig10908:19011801	19011801	19012500	700	1	0.000887811	22	3.142857143	NA	NA
DMRcontig10917:19025501	19025501	19026000	500	1	0.000271284	23	4.6	NA	NA
DMRcontig10956:19085599	19085599	19085900	302	2	0.000109827	8	2.649006623	NA	NA
DMRcontig10975:19114720	19114720	19115033	314	2	9.98E-05	3	0.955414013	NA	NA
DMRcontig11000:19151801	19151801	19152296	496	1	0.000945605	8	1.612903226	NA	NA
DMRcontig11010:19167701	19167701	19167964	264	2	0.000111266	5	1.893939394	NA	NA
DMRcontig11064:19249601	19249601	19250026	426	3	1.30E-06	9	2.112676056	NA	NA
DMRcontig11074:19264801	19264801	19265000	200	1	0.000141165	14	7	NA	NA
DMRcontig11087:19284101	19284101	19284300	200	1	0.000295435	3	1.5	NA	NA
DMRcontig11089:19287001	19287001	19287100	100	1	0.000939136	3	3	NA	NA
DMRcontig11106:19313401	19313401	19313700	300	1	0.000625634	7	2.333333333	NA	NA
DMRcontig11125:19341863	19341863	19342000	138	1	7.14E-05	10	7.246376812	NA	NA
DMRcontig11138:19360601	19360601	19360839	239	1	0.000428604	3	1.255230126	NA	NA
DMRcontig11200:19454101	19454101	19454300	200	1	0.000466468	6	3	NA	NA
DMRcontig11207:19464706	19464706	19465400	695	1	0.000847674	17	2.446043165	NA	NA
DMRcontig11224:19491560	19491560	19492000	441	3	0.000326101	6	1.360544218	NA	NA
DMRcontig11253:19535301	19535301	19535500	200	1	0.000146081	11	5.5	NA	NA
DMRcontig11274:19567101	19567101	19567361	261	1	3.14E-05	11	4.214559387	NA	NA
DMRcontig11283:19580901	19580901	19581163	263	1	0.000745426	10	3.802281369	NA	NA
DMRcontig11285:19583727	19583727	19584276	550	1	0.00018996	9	1.636363636	NA	NA
DMRcontig11297:19602221	19602221	19602422	202	2	0.000143596	3	1.485148515	NA	NA
DMRcontig11313:19626779	19626779	19627000	222	2	9.67E-06	6	2.702702703	NA	NA

DMRcontig11330:19652001	19652001	19652100	100	1	0.000627116	5	5	NA	NA
DMRcontig11359:19695101	19695101	19695500	400	2	0.000206397	7	1.75	NA	NA
DMRcontig11364:19703006	19703006	19703200	195	1	9.19E-05	9	4.615384615	NA	NA
DMRcontig11368:19708701	19708701	19708900	200	1	0.000153267	3	1.5	NA	NA
DMRcontig11376:19719482	19719482	19719700	219	1	0.000619964	3	1.369863014	NA	NA
DMRcontig11378:19722901	19722901	19723315	415	2	9.42E-05	8	1.927710843	NA	NA
DMRcontig11411:19772328	19772328	19772400	73	1	0.000419966	0	0	NA	NA
DMRcontig11424:19792701	19792701	19792864	164	1	0.000421054	6	3.658536585	NA	NA
DMRcontig11431:19802501	19802501	19802800	300	1	0.000762459	7	2.333333333	NA	NA
DMRcontig11434:19807401	19807401	19807874	474	1	0.000299797	22	4.641350211	NA	NA
DMRcontig11455:19839919	19839919	19840400	482	1	0.000218048	25	5.186721992	NA	NA
DMRcontig11456:19841701	19841701	19841820	120	1	0.000728821	0	0	NA	NA
DMRcontig11468:19859417	19859417	19859700	284	1	0.00085942	7	2.464788732	NA	NA
DMRcontig11479:19876001	19876001	19876800	800	1	2.80E-05	19	2.375	NA	NA
DMRcontig11498:19905325	19905325	19905860	536	2	0.000188753	11	2.052238806	NA	NA
DMRcontig11509:19922801	19922801	19922944	144	1	0.000552245	2	1.388888889	NA	NA
DMRcontig11519:19938101	19938101	19938342	242	1	0.000344287	4	1.652892562	NA	NA
DMRcontig11520:19939401	19939401	19939700	300	3	7.95E-05	8	2.666666667	NA	NA
DMRcontig11529:19952501	19952501	19952700	200	2	0.000574665	18	9	NA	NA
DMRcontig11547:19979201	19979201	19979600	400	3	1.30E-06	16	4	NA	NA
DMRcontig11581:20029117	20029117	20029400	284	1	6.30E-06	4	1.408450704	NA	NA
DMRcontig11588:20039401	20039401	20039700	300	2	5.11E-05	4	1.333333333	NA	NA
DMRcontig11590:20042201	20042201	20042577	377	2	7.07E-06	24	6.366047745	NA	NA
DMRcontig11593:20046749	20046749	20047184	436	2	6.49E-06	13	2.981651376	NA	NA
DMRcontig11598:20054597	20054597	20054900	304	1	0.000552477	3	0.986842105	NA	NA
DMRcontig11619:20084301	20084301	20084500	200	1	0.000192429	4	2	NA	NA
DMRcontig11622:20088501	20088501	20088831	331	3	0.00019377	11	3.32326284	NA	NA
DMRcontig11627:20095801	20095801	20096000	200	1	0.000314223	4	2	NA	NA
DMRcontig11665:20153101	20153101	20153500	400	1	0.000881527	12	3	NA	NA
DMRcontig11669:20159801	20159801	20160396	596	2	0.000355228	15	2.516778523	NA	NA
DMRcontig11698:20203144	20203144	20203700	557	1	0.00079346	7	1.256732496	NA	NA
DMRcontig11706:20214825	20214825	20215100	276	2	0.000492614	5	1.811594203	NA	NA
DMRcontig11711:20222220	20222220	20222887	668	1	0.000944663	12	1.796407186	NA	NA
DMRcontig11721:20238101	20238101	20238400	300	1	0.000403372	4	1.333333333	NA	NA
DMRcontig11725:20244148	20244148	20244500	353	1	0.0005092	6	1.699716714	NA	NA
DMRcontig11737:20263201	20263201	20263633	433	2	1.70E-05	12	2.771362587	NA	NA
DMRcontig11756:20290708	20290708	20291151	444	1	0.000569079	8	1.801801802	NA	NA
DMRcontig11771:20313061	20313061	20313300	240	1	0.000525303	2	0.833333333	NA	NA
DMRcontig11772:20314701	20314701	20315006	306	1	0.00087298	11	3.594771242	NA	NA
DMRcontig11816:20382601	20382601	20383000	400	2	0.000496946	13	3.25	NA	NA
DMRcontig11825:20396101	20396101	20396400	300	1	0.000699017	13	4.333333333	NA	NA
DMRcontig11838:20416001	20416001	20416413	413	1	2.19E-06	10	2.421307506	NA	NA
DMRcontig11854:20440637	20440637	20441100	464	1	0.000476544	14	3.017241379	NA	NA
DMRcontig11865:20457701	20457701	20458000	300	2	5.15E-07	14	4.666666667	NA	NA
DMRcontig11873:20470201	20470201	20470539	339	1	0.000128624	10	2.949852507	NA	NA
DMRcontig11877:20475901	20475901	20476300	400	1	0.000194839	16	4	NA	NA
DMRcontig11903:20513308	20513308	20513600	293	1	0.000131499	7	2.389078498	NA	NA
DMRcontig11907:20520201	20520201	20520556	356	1	0.000579152	17	4.775280899	NA	NA
DMRcontig11925:20546901	20546901	20547479	579	2	4.00E-05	14	2.417962003	NA	NA
DMRcontig11931:20555663	20555663	20556000	338	1	0.000750648	8	2.366863905	NA	NA
DMRcontig11932:20557301	20557301	20557411	111	1	0.000665265	2	1.801801802	NA	NA
DMRcontig11956:20592910	20592910	20593100	191	2	0.000571309	1	0.523560209	NA	NA
DMRcontig11958:20596001	20596001	20596493	493	1	0.000808502	31	6.288032454	NA	NA
DMRcontig11982:20631303	20631303	20631963	661	6	4.13E-05	44	6.656580938	LOC106177743	epigenetic
DMRcontig11983:20633001	20633001	20633200	200	2	4.28E-06	7	3.5	NA	NA
DMRcontig11985:20635862	20635862	20636300	439	1	0.000897239	18	4.10022779	NA	NA
DMRcontig11999:20657501	20657501	20657682	182	1	2.83E-06	4	2.197802198	NA	NA
DMRcontig12002:20661601	20661601	20661700	100	1	0.000381293	1	1	NA	NA
DMRcontig12007:20669401	20669401	20669800	400	1	0.0005129	5	1.25	NA	NA
DMRcontig12018:20685101	20685101	20685500	400	1	3.17E-05	25	6.25	NA	NA
DMRcontig12021:20689621	20689621	20689966	346	1	5.27E-07	13	3.757225434	NA	NA
DMRcontig12029:20701346	20701346	20701856	511	1	0.000541787	18	3.522504892	NA	NA
DMRcontig12047:20730601	20730601	20731000	400	1	0.000349884	23	5.75	NA	NA
DMRcontig12053:20739701	20739701	20740200	500	3	4.26E-06	15	3	NA	NA
DMRcontig12119:20839771	20839771	20840200	430	1	2.03E-05	36	8.372093023	NA	NA
DMRcontig12170:20915901	20915901	20916100	200	1	0.000921417	6	3	NA	NA
DMRcontig12176:20924509	20924509	20924855	347	1	0.00098	4	1.152737752	NA	NA
DMRcontig12185:20937701	20937701	20938132	432	2	9.90E-05	10	2.314814815	NA	NA
DMRcontig12206:20969012	20969012	20969366	355	1	2.56E-05	13	3.661971831	NA	NA
DMRcontig12235:21014260	21014260	21014400	141	2	7.70E-05	4	2.836879433	NA	NA
DMRcontig12245:21029601	21029601	21030100	500	1	0.00036827	10	2	NA	NA
DMRcontig12271:21068401	21068401	21068753	353	1	0.000282233	14	3.966005666	NA	NA
DMRcontig12276:21076009	21076009	21076200	192	1	0.000673473	14	7.291666667	NA	NA
DMRcontig12282:21084501	21084501	21084800	300	2	0.000513813	6	2	NA	NA
DMRcontig12284:21087501	21087501	21088387	887	3	6.01E-05	51	5.749718151	NA	NA
DMRcontig12308:21123748	21123748	21124063	316	2	0.00044796	9	2.848101266	NA	NA
DMRcontig12312:21129928	21129928	21130200	273	1	0.000444022	8	2.93040293	NA	NA
DMRcontig12315:21134201	21134201	21134700	500	1	0.00037421	16	3.2	NA	NA
DMRcontig12338:21169969	21169969	21170677	709	1	0.000167761	38	5.359661495	NA	NA
DMRcontig12344:21180007	21180007	21180365	359	1	0.000100755	13	3.621169916	NA	NA

DMRcontig12426:21303145	21303145	21303700	556	5	5.48E-06	12	2.158273381	NA	NA
DMRcontig12455:21348801	21348801	21349200	400	3	1.79E-07	13	3.25	NA	NA
DMRcontig12486:21394847	21394847	21395729	883	4	0.000141376	79	8.946772367	NA	NA
DMRcontig12530:21461101	21461101	21461640	540	1	0.000544381	28	5.185185185	NA	NA
DMRcontig12541:21478196	21478196	21478400	205	3	1.79E-06	8	3.902439024	NA	NA
DMRcontig12558:21503701	21503701	21503992	292	1	0.000521849	12	4.109589041	NA	NA
DMRcontig12562:21509401	21509401	21509600	200	1	0.000799675	2	1	NA	NA
DMRcontig12619:21593801	21593801	21593900	100	1	0.000152136	2	2	NA	NA
DMRcontig12620:21595101	21595101	21595500	400	1	0.000900506	10	2.5	NA	NA
DMRcontig12630:21610301	21610301	21610500	200	1	9.83E-05	3	1.5	NA	NA
DMRcontig12653:21643801	21643801	21644184	384	2	1.46E-05	10	2.604166667	NA	NA
DMRcontig12666:21662301	21662301	21662989	689	1	0.000921417	28	4.063860668	NA	NA
DMRcontig12672:21671615	21671615	21672000	386	1	0.000170269	9	2.331606218	NA	NA
DMRcontig12679:21682001	21682001	21683200	1200	1	7.70E-05	38	3.166666667	NA	NA
DMRcontig12723:21746801	21746801	21747254	454	1	0.000866169	18	3.964757709	NA	NA
DMRcontig12724:21748255	21748255	21748400	146	1	0.000185479	1	0.684931507	NA	NA
DMRcontig12727:21752434	21752434	21752930	497	1	0.000298594	13	2.615694165	NA	NA
DMRcontig12737:21768201	21768201	21768600	400	1	0.000561237	4	1	NA	NA
DMRcontig12747:21782901	21782901	21783072	172	1	2.86E-05	8	4.651162791	NA	NA
DMRcontig12749:21785819	21785819	21786100	282	1	0.000940218	3	1.063829787	NA	NA
DMRcontig12761:21804201	21804201	21804500	300	1	0.000582736	11	3.666666667	NA	NA
DMRcontig12771:21818205	21818205	21818400	196	1	0.000547557	3	1.530612245	NA	NA
DMRcontig12810:21877001	21877001	21877534	534	3	5.19E-06	11	2.059925094	NA	NA
DMRcontig12823:21896101	21896101	21896347	247	1	0.00082598	6	2.429149798	NA	NA
DMRcontig12870:21964801	21964801	21965300	500	1	0.000544756	8	1.6	NA	NA
DMRcontig12871:21966401	21966401	21966600	200	1	0.000901571	4	2	NA	NA
DMRcontig12883:21983976	21983976	21984300	325	1	9.41E-05	12	3.692307692	NA	NA
DMRcontig12884:21985401	21985401	21985600	200	1	0.000744269	10	5	TM9SF4	metabolism
DMRcontig12931:22054201	22054201	22054900	700	1	5.81E-06	14	2	NA	NA
DMRcontig12932:22056101	22056101	22056500	400	2	0.000197184	15	3.75	NA	NA
DMRcontig13000:22155401	22155401	22155600	200	1	0.00098	1	0.5	NA	NA
DMRcontig13007:22165442	22165442	22165700	259	1	5.74E-06	7	2.702702703	NA	NA
DMRcontig13030:22200201	22200201	22200697	497	1	0.000339834	23	4.6277666	NA	NA
DMRcontig13077:22269336	22269336	22269500	165	1	1.24E-05	1	0.606060606	NA	NA
DMRcontig13089:22287201	22287201	22287500	300	1	7.54E-05	21	7	NA	NA
DMRcontig13105:22310801	22310801	22311200	400	1	0.000157134	4	1	NA	NA
DMRcontig13121:22334801	22334801	22335300	500	1	9.99E-05	34	6.8	NA	NA
DMRcontig13123:22338001	22338001	22338500	500	3	0.000195643	15	3	NA	NA
DMRcontig13131:22350526	22350526	22351059	534	3	1.57E-05	12	2.247191011	NA	NA
DMRcontig13165:22400001	22400001	22400600	600	3	2.94E-05	18	3	NA	NA
DMRcontig13189:22435501	22435501	22435700	200	2	0.000298985	4	2	NA	NA
DMRcontig13196:22446001	22446001	22446081	81	1	0.0001741	2	2.469135802	NA	NA
DMRcontig13228:22491801	22491801	22492200	400	1	9.92E-05	8	2	NA	NA
DMRcontig13234:22500401	22500401	22500900	500	1	0.000328962	14	2.8	NA	NA
DMRcontig13252:22526501	22526501	22526600	100	1	0.000957718	8	8	NA	NA
DMRcontig13273:22558180	22558180	22558802	623	2	0.000373345	30	4.81540931	NA	NA
DMRcontig13281:22570501	22570501	22570900	400	2	2.41E-05	5	1.25	NA	NA
DMRcontig13288:22581032	22581032	22581500	469	5	2.39E-06	25	5.330490405	NA	NA
DMRcontig13291:22585401	22585401	22585911	511	4	7.31E-06	17	3.326810176	NA	NA
DMRcontig13308:22610847	22610847	22611000	154	1	0.000503004	3	1.948051948	NA	NA
DMRcontig13314:22619759	22619759	22620000	242	1	0.000257989	15	6.198347107	NA	NA
DMRcontig13323:22634101	22634101	22635000	900	3	7.80E-05	52	5.777777778	NA	NA
DMRcontig13348:22671601	22671601	22671863	263	1	0.000774028	1	0.380228137	NA	NA
DMRcontig13362:22693801	22693801	22694421	621	2	7.47E-05	30	4.830917874	NA	NA
DMRcontig13376:22715201	22715201	22715391	191	2	1.04E-07	7	3.664921466	NCAS0C03520	miscellaneous
DMRcontig13381:22722401	22722401	22722500	100	1	0.00097296	5	5	NA	NA
DMRcontig13402:22753101	22753101	22753200	100	1	0.000768166	1	1	NA	NA
DMRcontig13413:22768680	22768680	22769200	521	3	9.70E-05	14	2.687140115	NA	NA
DMRcontig13428:22791062	22791062	22791311	250	1	0.00080502	7	2.8	NA	NA
DMRcontig13463:22841801	22841801	22842000	200	2	0.000805934	2	1	NA	NA
DMRcontig13467:22848001	22848001	22848200	200	1	9.63E-08	7	3.5	NA	NA
DMRcontig13498:22893401	22893401	22893801	401	1	0.00073914	27	6.733167082	NA	NA
DMRcontig13506:22906101	22906101	22906200	100	1	0.00017509	3	3	NA	NA
DMRcontig13515:22919901	22919901	22920161	261	1	0.0001996	7	2.681992337	NA	NA
DMRcontig13523:22931201	22931201	22931829	629	1	9.42E-05	22	3.497615262	NA	NA
DMRcontig13539:22955451	22955451	22956222	772	3	1.99E-05	20	2.590673575	NA	NA
DMRcontig13543:22961901	22961901	22962100	200	1	0.000155884	6	3	NA	NA
DMRcontig13555:22979701	22979701	22979800	100	1	0.000206597	1	1	NA	NA
DMRcontig13580:23016601	23016601	23016900	300	2	6.04E-05	22	7.333333333	NA	NA
DMRcontig13586:23025644	23025644	23026100	457	4	1.45E-05	15	3.282275711	NA	NA
DMRcontig13590:23032401	23032401	23032600	200	2	2.76E-07	6	3	NA	NA
DMRcontig13594:23038001	23038001	23038500	500	1	0.000728069	29	5.8	LOC100533391	cytoskeleton
DMRcontig13603:23052901	23052901	23053042	142	2	0.000314223	2	1.408450704	NA	NA
DMRcontig13607:23058801	23058801	23059200	400	1	0.000294687	8	2	NA	NA
DMRcontig13614:23069601	23069601	23069698	98	1	0.000893436	2	2.040816327	NA	NA
DMRcontig13617:23073901	23073901	23074100	200	1	0.000497912	7	3.5	NA	NA
DMRcontig13624:23084201	23084201	23084600	400	2	0.000343279	19	4.75	NA	NA
DMRcontig13652:23127001	23127001	23127380	380	2	0.000633893	6	1.578947368	NA	NA
DMRcontig13664:23145701	23145701	23146000	300	1	0.000181218	4	1.333333333	NA	NA
DMRcontig13677:23164223	23164223	23164981	759	4	9.00E-05	22	2.898550725	NA	NA

DMRcontig13689:23182101	23182101	23182200	100	1	0.000733581	9	9	NA	NA
DMRcontig13696:23192817	23192817	23193122	306	1	0.000492076	6	1.960784314	NA	NA
DMRcontig13729:23241101	23241101	23241200	100	1	0.000744642	1	1	NA	NA
DMRcontig13733:23247301	23247301	23247600	300	1	1.38E-05	12	4	NA	NA
DMRcontig13736:23251679	23251679	23251800	122	1	0.00043807	0	0	NA	NA
DMRcontig13754:23277350	23277350	23277600	251	1	0.000111395	17	6.772908367	NA	NA
DMRcontig13774:23307701	23307701	23308000	300	1	0.000782867	18	6	NA	NA
DMRcontig13779:23315113	23315113	23315300	188	1	0.000438112	3	1.595744681	NA	NA
DMRcontig13806:23355301	23355301	23356200	900	2	0.000676338	10	1.111111111	NA	NA
DMRcontig13807:23357472	23357472	23358002	531	2	0.00017383	7	1.31826742	NA	NA
DMRcontig13832:23394901	23394901	23395332	432	1	0.000183574	12	2.777777778	NA	NA
DMRcontig13852:23424231	23424231	23424858	628	1	0.000427135	16	2.547770701	NA	NA
DMRcontig13883:23469001	23469001	23469182	182	2	0.000423801	4	2.197802198	NA	NA
DMRcontig13901:23496039	23496039	23496200	162	1	0.000170175	15	9.259259259	NA	NA
DMRcontig13936:23548401	23548401	23548800	400	2	3.11E-07	8	2	LOC107987209;LOC107984688;LOC108168064;LOC107973554;HOXD10;Spon1;PDE1B	cytoskeleton
DMRcontig13949:23568601	23568601	23568800	200	1	0.000228264	7	3.5	NA	NA
DMRcontig13950:23570201	23570201	23570645	445	1	0.000139455	9	2.02247191	NA	NA
DMRcontig13979:23613358	23613358	23613500	143	1	0.000901891	9	6.293706294	NA	NA
DMRcontig13997:23640501	23640501	23640776	276	1	0.000290515	10	3.623188406	NA	NA
DMRcontig14014:23663801	23663801	23664095	295	2	6.33E-08	2	0.677966102	NA	NA
DMRcontig14032:23690501	23690501	23690900	400	1	0.000106166	11	2.75	NA	NA
DMRcontig14064:23738204	23738204	23738500	297	2	1.05E-05	11	3.703703704	NA	NA
DMRcontig14068:23744101	23744101	23744400	300	1	0.000672517	22	7.333333333	NA	NA
DMRcontig14078:23758901	23758901	23759141	241	1	0.000565773	4	1.659751037	NA	NA
DMRcontig14083:23766311	23766311	23766600	290	1	0.000527265	7	2.413793103	NA	NA
DMRcontig14097:23787101	23787101	23787500	400	1	0.000943619	6	1.5	NA	NA
DMRcontig14098:23788701	23788701	23788883	183	1	0.000229853	6	3.278688525	NA	NA
DMRcontig14129:23834701	23834701	23835000	300	1	0.000123654	14	4.666666667	LOC105398638	signaling
DMRcontig14135:23843072	23843072	23843300	229	1	0.000571834	6	2.620087336	NA	NA
DMRcontig14140:23850301	23850301	23850800	500	1	0.000260844	16	3.2	NA	NA
DMRcontig14141:23852101	23852101	23852309	209	2	1.48E-05	3	1.435406699	NA	NA
DMRcontig14177:23905501	23905501	23906100	600	2	0.000219953	12	2	NA	NA
DMRcontig14178:23907701	23907701	23908000	300	1	0.000117946	4	1.333333333	NA	NA
DMRcontig14193:23929729	23929729	23930300	572	1	0.000585635	23	4.020979021	NA	NA
DMRcontig14218:23965716	23965716	23966000	285	2	1.65E-05	1	0.350877193	NA	NA
DMRcontig14233:23987003	23987003	23988000	998	3	0.000117735	22	2.204408818	NA	NA
DMRcontig14244:24004201	24004201	24004477	277	1	0.00047031	17	6.137184116	NA	NA
DMRcontig14256:24021601	24021601	24022000	400	2	0.00031505	13	3.25	NA	NA
DMRcontig14260:24027266	24027266	24027700	435	1	0.000529283	15	3.448275862	NA	NA
DMRcontig14291:24073001	24073001	24073237	237	1	0.000595005	4	1.687763713	NA	NA
DMRcontig14299:24084701	24084701	24084800	100	1	0.000548258	4	4	NA	NA
DMRcontig14301:24087303	24087303	24087860	558	2	0.000152078	17	3.046594982	NA	NA
DMRcontig14305:24092806	24092806	24093440	635	1	0.000575034	18	2.834645669	NA	NA
DMRcontig14325:24122301	24122301	24122500	200	1	0.000363797	2	1	NA	NA
DMRcontig14338:24141427	24141427	24141600	174	1	0.000995474	6	3.448275862	NA	NA
DMRcontig14369:24186913	24186913	24187100	188	2	1.09E-05	6	3.191489362	PNEG_01177;PRKCSH	miscellaneous
DMRcontig14376:24197232	24197232	24197500	269	1	0.000543688	4	1.486988848	NA	NA
DMRcontig14382:24205923	24205923	24206350	428	2	5.92E-06	16	3.738317757	NA	NA
DMRcontig14398:24229801	24229801	24230000	200	2	0.000211365	5	2.5	NA	NA
DMRcontig14401:24234321	24234321	24234590	270	1	6.54E-06	5	1.851851852	NA	NA
DMRcontig14425:24271001	24271001	24271300	300	1	0.00043809	6	2	NA	NA
DMRcontig14432:24281177	24281177	24281400	224	2	0.000160434	5	2.232142857	NA	NA
DMRcontig14434:24284201	24284201	24284400	200	2	0.000259303	9	4.5	NA	NA
DMRcontig14479:24349958	24349958	24350100	143	1	0.000733577	7	4.895104895	NA	NA
DMRcontig14483:24355605	24355605	24356100	496	1	1.65E-05	7	1.411290323	NA	NA
DMRcontig14488:24363901	24363901	24364200	300	1	2.87E-05	19	6.333333333	NA	NA
DMRcontig14513:24400801	24400801	24401000	200	1	8.04E-06	17	8.5	NA	NA
DMRcontig14524:24416703	24416703	24417300	598	1	0.000228262	26	4.347826087	NA	NA
DMRcontig14558:24466601	24466601	24466832	232	1	0.000502786	3	1.293103448	NA	NA
DMRcontig14597:24524601	24524601	24524948	348	2	0.000241872	8	2.298850575	NA	NA
DMRcontig14602:24531642	24531642	24532037	396	1	7.34E-05	11	2.777777778	NA	NA
DMRcontig14615:24551401	24551401	24551500	100	1	0.00024536	2	2	NA	NA
DMRcontig14625:24566431	24566431	24566800	370	1	0.000799398	12	3.243243243	NA	NA
DMRcontig14627:24569301	24569301	24570100	800	1	0.000526995	32	4	NA	NA
DMRcontig14671:24632667	24632667	24632800	134	2	2.83E-08	2	1.492537313	NA	NA
DMRcontig14678:24643227	24643227	24643832	606	1	0.0009213	13	2.145214521	NA	NA
DMRcontig14721:24707101	24707101	24707300	200	1	0.000796521	6	3	NA	NA
DMRcontig14773:24784740	24784740	24785100	361	3	0.000153743	9	2.493074792	NA	NA
DMRcontig14782:24798401	24798401	24798600	200	1	0.000140247	11	5.5	NA	NA
DMRcontig14791:24811601	24811601	24811800	200	1	0.000885103	3	1.5	NA	NA
DMRcontig14793:24814264	24814264	24814858	595	2	0.000195787	39	6.554621849	NA	NA
DMRcontig14799:24823301	24823301	24823700	400	1	1.46E-05	5	1.25	NA	NA
DMRcontig14836:24878557	24878557	24879296	740	2	0.000152516	46	6.216216216	NCLIV_060450;NCLIV_060710;LHFPL3	miscellaneous
DMRcontig14857:24910101	24910101	24910300	200	1	0.000604072	9	4.5	NA	NA
DMRcontig14862:24917822	24917822	24918519	698	3	0.000266851	43	6.160458453	NA	NA
DMRcontig14872:24932101	24932101	24932359	259	1	3.83E-07	7	2.702702703	NA	NA
DMRcontig14873:24933360	24933360	24933600	241	2	0.0001396	16	6.639004149	NA	NA

DMRcontig14880:24943217	24943217	24943654	438	1	3.38E-05	10	2.283105023	NA	NA
DMRcontig14885:24950601	24950601	24951200	600	1	9.86E-05	20	3.333333333	NA	NA
DMRcontig14889:24956849	24956849	24957300	452	1	0.000552244	13	2.876106195	NA	NA
DMRcontig14893:24962695	24962695	24963200	506	2	3.53E-05	21	4.150197628	NA	NA
DMRcontig14907:24983601	24983601	24983800	200	1	0.000490345	8	4	NA	NA
DMRcontig14922:25005672	25005672	25005900	229	3	1.82E-06	7	3.056768559	NA	NA
DMRcontig14979:25090301	25090301	25090426	126	1	0.000403357	1	0.793650794	NA	NA
DMRcontig14980:25091901	25091901	25092173	273	1	0.000267243	18	6.593406593	NA	NA
DMRcontig14983:25095959	25095959	25096200	242	1	0.000830073	5	2.066115702	NA	NA
DMRcontig14999:25118501	25118501	25118700	200	1	0.000963897	10	5	NA	NA
DMRcontig15004:25125601	25125601	25125887	287	1	0.000292207	12	4.181184669	NA	NA
DMRcontig15010:25134001	25134001	25134200	200	2	0.000124266	0	0	NA	NA
DMRcontig15014:25140101	25140101	25140566	466	2	8.83E-05	25	5.364806867	NA	NA
DMRcontig15023:25153501	25153501	25153741	241	2	0.00048193	2	0.829875519	NA	NA
DMRcontig15046:25186701	25186701	25187000	300	1	0.000469833	7	2.333333333	NA	NA
DMRcontig15052:25194611	25194611	25194774	164	2	0.000128917	0	0	NA	NA
DMRcontig15062:25209701	25209701	25210200	500	2	0.000133161	24	4.8	NA	NA
DMRcontig15105:25274312	25274312	25275000	689	3	3.22E-05	32	4.644412192	NA	NA
DMRcontig15108:25278814	25278814	25279144	331	1	0.000791329	13	3.927492447	NA	NA
DMRcontig15123:25300101	25300101	25300400	300	1	0.000182499	10	3.333333333	NA	NA
DMRcontig15206:25423501	25423501	25423900	400	1	0.000246933	22	5.5	NA	NA
DMRcontig15241:25472943	25472943	25473363	421	2	0.000223352	9	2.137767221	NA	NA
DMRcontig15247:25481801	25481801	25482200	400	2	0.000198958	7	1.75	NA	NA
DMRcontig15248:25483301	25483301	25483573	273	1	0.000274502	17	6.227106227	IscW_ISCW018476	cytoskeleton
DMRcontig15258:25498701	25498701	25498900	200	1	0.000896168	6	3	NA	NA
DMRcontig15296:25553856	25553856	25554000	145	1	0.000669221	10	6.896551724	NA	NA
DMRcontig15300:25559601	25559601	25559969	369	2	2.74E-05	12	3.25203252	NA	NA
DMRcontig15302:25562468	25562468	25562690	223	1	0.000714823	3	1.34529148	NA	NA
DMRcontig15310:25573701	25573701	25574100	400	1	0.000165519	0	0	NA	NA
DMRcontig15312:25576901	25576901	25577900	1000	1	0.000207027	64	6.4	NA	NA
DMRcontig15313:25578941	25578941	25579700	760	1	0.000599082	31	4.078947368	LOC105330573;Pepck	metabolism
DMRcontig15314:25580901	25580901	25581300	400	4	4.59E-05	16	4	NA	NA
DMRcontig15320:25590401	25590401	25590700	300	1	0.000197763	4	1.333333333	NA	NA
DMRcontig15328:25602301	25602301	25602500	200	1	0.000850154	4	2	NA	NA
DMRcontig15337:25616101	25616101	25616273	173	1	0.00056961	0	0	NA	NA
DMRcontig15338:25617301	25617301	25617600	300	2	0.000154491	14	4.666666667	NA	NA
DMRcontig15350:25634039	25634039	25634560	522	3	5.41E-08	16	3.0651341	NA	NA
DMRcontig15370:25663101	25663101	25663300	200	1	0.00084332	5	2.5	NA	NA
DMRcontig15371:25664367	25664367	25665295	929	1	2.86E-06	52	5.597416577	NA	NA
DMRcontig15408:25719101	25719101	25719300	200	1	0.000286676	4	2	NA	NA
DMRcontig15434:25756101	25756101	25756500	400	2	1.35E-05	10	2.5	NA	NA
DMRcontig15461:25796101	25796101	25796600	500	5	2.43E-05	21	4.2	NA	NA
DMRcontig15485:25832216	25832216	25832600	385	1	2.49E-06	18	4.675324675	NA	NA
DMRcontig15506:25863601	25863601	25864400	800	4	9.21E-05	40	5	NA	NA
DMRcontig15507:25865441	25865441	25865669	229	1	0.000556288	9	3.930131004	NA	NA
DMRcontig15527:25893471	25893471	25893890	420	2	7.60E-06	9	2.142857143	NA	NA
DMRcontig15545:25918837	25918837	25919200	364	2	1.31E-05	11	3.021978022	NA	NA
DMRcontig15561:25942423	25942423	25942899	477	3	1.21E-07	4	0.838574423	NA	NA
DMRcontig15574:25961601	25961601	25961700	100	1	0.000552245	2	2	NA	NA
DMRcontig15604:26004793	26004793	26005200	408	2	0.000121506	9	2.205882353	NA	NA
DMRcontig15626:26037113	26037113	26037317	205	2	8.69E-05	8	3.902439024	NA	NA
DMRcontig15627:26038318	26038318	26038882	565	4	3.69E-07	13	2.300884956	NA	NA
DMRcontig15631:26044129	26044129	26044300	172	1	0.000134272	3	1.744186047	NA	NA
DMRcontig15635:26049624	26049624	26050062	439	2	7.53E-08	18	4.10022779	NA	NA
DMRcontig15663:26089701	26089701	26090075	375	1	2.11E-05	6	1.6	NA	NA
DMRcontig15674:26105438	26105438	26105772	335	1	0.000639363	8	2.388059701	NA	NA
DMRcontig15692:26131076	26131076	26131300	225	1	0.000452907	8	3.555555556	NA	NA
DMRcontig15731:26188008	26188008	26188363	356	3	6.19E-07	17	4.775280899	LOC106177743;LOC106177744;<br>LOC106177745	epigenetic
DMRcontig15734:26192201	26192201	26192795	595	3	1.44E-05	37	6.218487395	NA	NA
DMRcontig15736:26195147	26195147	26195491	345	2	0.000141022	17	4.927536232	NA	NA
DMRcontig15745:26207963	26207963	26208400	438	1	0.000912027	18	4.109589041	NA	NA
DMRcontig15747:26211012	26211012	26211200	189	1	0.000867505	4	2.116402116	NA	NA
DMRcontig15793:26277442	26277442	26277900	459	1	0.000544893	21	4.575163399	NA	NA
DMRcontig15821:26318601	26318601	26318700	100	1	4.99E-06	5	5	NA	NA
DMRcontig15823:26321301	26321301	26321900	600	1	0.000290487	13	2.166666667	NA	NA
DMRcontig15839:26345601	26345601	26345800	200	1	0.000876273	11	5.5	LOC106588560	transcription
DMRcontig15840:26347101	26347101	26347400	300	1	0.000818197	9	3	NA	NA
DMRcontig15842:26349908	26349908	26350200	293	1	0.000454674	12	4.09556314	NA	NA
DMRcontig15876:26399101	26399101	26399300	200	2	2.99E-08	7	3.5	NA	NA
DMRcontig15883:26409401	26409401	26409700	300	2	0.00034022	10	3.333333333	NA	NA
DMRcontig15923:26466401	26466401	26466524	124	1	0.0009418	1	0.806451613	NA	NA
DMRcontig15937:26486601	26486601	26487000	400	1	8.43E-05	11	2.75	NA	NA
DMRcontig15942:26494101	26494101	26494235	135	2	5.62E-05	5	3.703703704	NA	NA
DMRcontig15970:26532901	26532901	26533200	300	1	0.000445033	1	0.333333333	NA	NA
DMRcontig15987:26558501	26558501	26558900	400	3	2.19E-08	9	2.25	NA	NA
DMRcontig15989:26561501	26561501	26561857	357	1	0.000864852	7	1.960784314	NA	NA
DMRcontig15998:26574301	26574301	26574900	600	2	4.94E-05	9	1.5	NA	NA
DMRcontig16002:26579969	26579969	26580500	532	1	0.000298207	13	2.443609023	NA	NA
DMRcontig16005:26584101	26584101	26584400	300	1	0.000270855	14	4.666666667	NA	NA

DMRcontig16015:26599101	26599101	26599400	300	2	1.99E-06	6	2	RPL19	translation
DMRcontig16030:26621701	26621701	26622012	312	2	2.23E-05	6	1.923076923	NA	NA
DMRcontig16038:26633001	26633001	26633398	398	3	8.39E-07	7	1.75879397	NA	NA
DMRcontig16049:26649201	26649201	26649846	646	2	4.67E-07	22	3.405572755	NA	NA
DMRcontig16052:26653701	26653701	26653900	200	1	0.000819469	2	1	LOC107280826	unknown
DMRcontig16095:26716201	26716201	26716451	251	1	0.000599912	7	2.788844622	NA	NA
DMRcontig16107:26733240	26733240	26733970	731	1	0.000306706	26	3.556771546	NA	NA
DMRcontig16113:26742201	26742201	26742480	280	1	0.000181786	10	3.571428571	NA	NA
DMRcontig16116:26746901	26746901	26747047	147	2	1.80E-05	2	1.360544218	NA	NA
DMRcontig16134:26772401	26772401	26772700	300	1	0.000115682	10	3.333333333	NA	NA
DMRcontig16138:26777951	26777951	26778300	350	2	0.000269645	3	0.857142857	NA	NA
DMRcontig16165:26818701	26818701	26819000	300	3	6.61E-05	7	2.333333333	NA	NA
DMRcontig16192:26858401	26858401	26858600	200	2	0.000154674	3	1.5	NA	NA
DMRcontig16203:26874801	26874801	26875000	200	2	0.000189011	4	2	NA	NA
DMRcontig16221:26900301	26900301	26900640	340	2	0.000225265	18	5.294117647	NA	NA
DMRcontig16271:26973962	26973962	26974312	351	1	0.000655157	14	3.988603989	NA	NA
DMRcontig16277:26983001	26983001	26983199	199	1	0.000166956	1	0.502512563	NA	NA
DMRcontig16285:26995146	26995146	26995385	240	1	0.000251494	7	2.916666667	NA	NA
DMRcontig16290:27002119	27002119	27002400	282	2	0.000376591	17	6.028368794	NA	NA
DMRcontig16301:27017434	27017434	27017904	471	2	5.93E-07	12	2.547770701	NA	NA
DMRcontig16334:27066163	27066163	27066438	276	2	9.71E-05	3	1.086956522	NA	NA
DMRcontig16373:27123281	27123281	27123600	320	1	0.000215422	13	4.0625	NA	NA
DMRcontig16378:27130416	27130416	27130800	385	1	0.00099205	14	3.636363636	NA	NA
DMRcontig16403:27167101	27167101	27167260	160	2	0.000197637	9	5.625	NA	NA
DMRcontig16414:27182701	27182701	27183000	300	1	0.000320837	18	6	TRIVIDRAFT_208649	miscellaneous
DMRcontig16423:27195801	27195801	27196100	300	3	2.36E-05	5	1.666666667	NA	NA
DMRcontig16432:27209101	27209101	27209300	200	1	0.000413335	7	3.5	NA	NA
DMRcontig16444:27225802	27225802	27226000	199	1	0.000557382	3	1.507537688	NA	NA
DMRcontig16472:27266501	27266501	27266600	100	1	0.000121652	0	0	NA	NA
DMRcontig16486:27284748	27284748	27284936	189	1	0.000679094	1	0.529100529	NA	NA
DMRcontig16488:27287413	27287413	27287678	266	1	0.000485655	10	3.759398496	NA	NA
DMRcontig16493:27294101	27294101	27294551	451	2	6.76E-05	12	2.66075388	NA	NA
DMRcontig16497:27299720	27299720	27300100	381	3	3.48E-06	21	5.511811024	NA	NA
DMRcontig16501:27305469	27305469	27305600	132	2	0.000124715	13	9.848484848	NA	NA
DMRcontig16533:27353001	27353001	27353286	286	1	0.000396404	12	4.195804196	NA	NA
DMRcontig16538:27359801	27359801	27360282	482	1	1.46E-05	13	2.697095436	NA	NA
DMRcontig16545:27369201	27369201	27369900	700	1	0.000532776	33	4.714285714	NA	NA
DMRcontig16546:27371201	27371201	27371438	238	1	0.000801081	5	2.100840336	NA	NA
DMRcontig16582:27423601	27423601	27423991	391	1	0.000161255	10	2.557544757	NA	NA
DMRcontig16601:27450701	27450701	27450900	200	1	7.38E-05	2	1	NA	NA
DMRcontig16637:27502013	27502013	27502400	388	2	0.000113993	8	2.06185567	NA	NA
DMRcontig16648:27517501	27517501	27517700	200	1	9.99E-05	0	0	NA	NA
DMRcontig16697:27589276	27589276	27589800	525	1	0.000733581	12	2.285714286	NA	NA
DMRcontig16699:27592901	27592901	27593048	148	2	0.00035643	6	4.054054054	NA	NA
DMRcontig16700:27594101	27594101	27594300	200	1	0.000745588	7	3.5	NA	NA
DMRcontig16712:27611787	27611787	27612100	314	1	0.00024356	4	1.27388535	NA	NA
DMRcontig16719:27621626	27621626	27622000	375	4	1.38E-07	10	2.666666667	NA	NA
DMRcontig16729:27636801	27636801	27636928	128	1	0.000390119	0	0	CARUB_v10020777mg;L484_001827	miscellaneous
DMRcontig16750:27666750	27666750	27666967	218	3	2.79E-05	2	0.917431193	NA	NA
DMRcontig16765:27687701	27687701	27688000	300	2	0.000223268	6	2	NA	NA
DMRcontig16768:27692344	27692344	27692700	357	1	0.000128022	29	8.1232493	NA	NA
DMRcontig16775:27703201	27703201	27703363	163	1	2.49E-05	5	3.067484663	P38mapk	signaling
DMRcontig16782:27713201	27713201	27713800	600	1	0.000306954	28	4.666666667	NA	NA
DMRcontig16842:27800501	27800501	27801200	700	1	0.0009464	34	4.857142857	NA	NA
DMRcontig16855:27820101	27820101	27820468	368	1	0.000491635	7	1.902173913	NA	NA
DMRcontig16889:27868101	27868101	27868567	467	1	0.000811106	24	5.139186296	NA	NA
DMRcontig16891:27871033	27871033	27871500	468	1	0.000730453	9	1.923076923	NA	NA
DMRcontig16907:27894701	27894701	27895166	466	2	0.000457678	5	1.072961373	NA	NA
DMRcontig16914:27904814	27904814	27905380	567	4	3.96E-05	18	3.174603175	NA	NA
DMRcontig16975:27994201	27994201	27994500	300	1	0.000616185	8	2.666666667	NA	NA
DMRcontig16988:28013301	28013301	28013500	200	1	0.000375976	1	0.5	NA	NA
DMRcontig17005:28037501	28037501	28037669	169	1	0.000553797	15	8.875739645	LOC101852687	cytoskeleton
DMRcontig17015:28051162	28051162	28051400	239	1	0.00040749	18	7.531380753	NA	NA
DMRcontig17023:28062947	28062947	28063500	554	3	8.20E-07	26	4.693140794	NA	NA
DMRcontig17046:28095401	28095401	28095670	270	1	0.000730453	4	1.481481481	NA	NA
DMRcontig17069:28127186	28127186	28127600	415	1	0.000673701	12	2.891566265	NA	NA
DMRcontig17075:28135901	28135901	28136100	200	1	1.25E-05	5	2.5	NA	NA
DMRcontig17096:28166201	28166201	28166525	325	1	0.000166645	10	3.076923077	NA	NA
DMRcontig17113:28190860	28190860	28191100	241	1	0.000490345	5	2.074688797	NA	NA
DMRcontig17135:28221320	28221320	28221700	381	1	0.000425448	21	5.511811024	NA	NA
DMRcontig17138:28225601	28225601	28225843	243	1	0.000232113	4	1.646090535	NA	NA
DMRcontig17147:28238701	28238701	28238851	151	1	0.00064589	3	1.986754967	NA	NA
DMRcontig17180:28285101	28285101	28285400	300	1	6.59E-06	17	5.666666667	NA	NA
DMRcontig17214:28333401	28333401	28333940	540	1	2.30E-06	35	6.481481481	NA	NA
DMRcontig17219:28340501	28340501	28341300	800	2	2.65E-07	17	2.125	NA	NA
DMRcontig17258:28395801	28395801	28396600	800	1	0.000667153	23	2.875	NA	NA
DMRcontig17262:28401688	28401688	28402000	313	1	1.44E-05	9	2.875399361	NA	NA
DMRcontig17299:28455201	28455201	28455600	400	1	0.000307387	12	3	NA	NA
DMRcontig17323:28489201	28489201	28489300	100	1	0.000105137	0	0	NA	NA

DMRcontig17331:28500023	28500023	28500200	178	1	4.33E-05	2	1.123595506	NA	NA
DMRcontig17349:28525064	28525064	28525400	337	2	2.55E-06	13	3.857566766	NA	NA
DMRcontig17355:28533712	28533712	28534000	289	1	0.000212876	9	3.114186851	NA	NA
DMRcontig17363:28544501	28544501	28544943	443	1	0.000792504	11	2.483069977	NA	NA
DMRcontig17365:28547030	28547030	28547275	246	2	0.000220046	5	2.032520325	NA	NA
DMRcontig17380:28569101	28569101	28569188	88	1	0.000666144	1	1.136363636	NA	NA
DMRcontig17387:28579072	28579072	28579200	129	1	0.000939167	2	1.550387597	NA	NA
DMRcontig17403:28601601	28601601	28601900	300	1	0.000172007	8	2.666666667	NA	NA
DMRcontig17468:28692572	28692572	28692900	329	2	4.25E-05	16	4.863221884	NA	NA
DMRcontig17488:28721365	28721365	28722100	736	1	0.00078679	21	2.85326087	CAOG_05898;CAMK2B	signaling
DMRcontig17496:28732701	28732701	28732800	100	1	0.000942967	0	0	NA	NA
DMRcontig17506:28746601	28746601	28747400	800	2	0.000558721	13	1.625	NA	NA
DMRcontig17522:28768501	28768501	28769000	500	2	0.000290035	14	2.8	NA	NA
DMRcontig17523:28770260	28770260	28770560	301	3	6.79E-07	8	2.657807309	NA	NA
DMRcontig17543:28798701	28798701	28799259	559	1	6.24E-06	9	1.610017889	NA	NA
DMRcontig17561:28824901	28824901	28824985	85	1	0.00098	1	1.176470588	NA	NA
DMRcontig17563:28827208	28827208	28827482	275	1	0.000512129	1	0.363636364	NA	NA
DMRcontig17576:28845301	28845301	28845679	379	1	0.000795401	10	2.638522427	NA	NA
DMRcontig17583:28855201	28855201	28855700	500	1	0.000630395	7	1.4	NA	NA
DMRcontig17604:28886260	28886260	28886632	373	3	1.71E-06	8	2.144772118	NA	NA
DMRcontig17612:28898701	28898701	28899000	300	1	4.24E-05	16	5.333333333	NA	NA
DMRcontig17616:28904541	28904541	28905000	460	2	9.48E-05	12	2.608695652	NA	NA
DMRcontig17645:28945701	28945701	28946100	400	1	0.000303758	34	8.5	NA	NA
DMRcontig17719:29049136	29049136	29049300	165	1	0.000113986	1	0.606060606	NA	NA
DMRcontig17736:29073014	29073014	29073545	532	2	0.000230792	11	2.067669173	NA	NA
DMRcontig17762:29111601	29111601	29111800	200	1	0.000921417	11	5.5	NA	NA
DMRcontig17800:29166856	29166856	29167000	145	1	0.000887811	4	2.75862069	NA	NA
DMRcontig17814:29185901	29185901	29186100	200	2	0.000328537	1	0.5	NA	NA
DMRcontig17819:29192746	29192746	29192900	155	2	0.000219883	4	2.580645161	NA	NA
DMRcontig17826:29203101	29203101	29203300	200	1	0.000492614	5	2.5	NA	NA
DMRcontig17838:29219669	29219669	29219800	132	1	0.000108766	3	2.272727273	NA	NA
DMRcontig17856:29245901	29245901	29246100	200	1	0.000263962	18	9	LOC101399673;ARF6	translation and protein modification
DMRcontig17860:29251785	29251785	29252099	315	2	0.000430625	8	2.53968254	NA	NA
DMRcontig17881:29280901	29280901	29281200	300	2	0.000459114	1	0.333333333	NA	NA
DMRcontig17888:29290134	29290134	29290468	335	3	1.70E-05	12	3.582089552	NA	NA
DMRcontig17893:29296901	29296901	29297400	500	2	0.000443743	14	2.8	NA	NA
DMRcontig17955:29382352	29382352	29382960	609	1	0.000453015	30	4.926108374	NA	NA
DMRcontig17986:29427001	29427001	29427400	400	1	0.000864419	15	3.75	NA	NA
DMRcontig18025:29482701	29482701	29482900	200	1	0.000516521	4	2	NA	NA
DMRcontig18068:29540910	29540910	29541100	191	1	0.000325154	9	4.712041885	NA	NA
DMRcontig18079:29556101	29556101	29556500	400	1	0.00062767	16	4	NA	NA
DMRcontig18084:29563501	29563501	29563988	488	1	3.57E-05	13	2.663934426	NA	NA
DMRcontig18113:29604816	29604816	29605300	485	2	0.00034379	5	1.030927835	NA	NA
DMRcontig18114:29606401	29606401	29606900	500	1	0.000327122	32	6.4	NA	NA
DMRcontig18135:29637001	29637001	29637200	200	1	0.000107588	10	5	NA	NA
DMRcontig18171:29687401	29687401	29687733	333	1	0.000314223	8	2.402402402	NA	NA
DMRcontig18177:29695401	29695401	29696000	600	3	4.73E-05	21	3.5	NA	NA
DMRcontig18193:29719109	29719109	29719300	192	1	0.000559974	6	3.125	NA	NA
DMRcontig18225:29762201	29762201	29762300	100	1	0.000328971	1	1	NA	NA
DMRcontig18233:29773901	29773901	29774200	300	1	0.000167961	20	6.666666667	NA	NA
DMRcontig18236:29778201	29778201	29778516	316	2	0.000310829	3	0.949367089	NA	NA
DMRcontig18264:29818701	29818701	29819100	400	1	0.000227281	11	2.75	NA	NA
DMRcontig18269:29825801	29825801	29826400	600	1	0.00080987	31	5.166666667	NA	NA
DMRcontig18273:29831621	29831621	29832697	1077	5	1.41E-07	22	2.042711235	NA	NA
DMRcontig18296:29864201	29864201	29864700	500	1	0.000226768	24	4.8	NA	NA
DMRcontig18313:29888101	29888101	29888460	360	1	0.000645333	3	0.833333333	NA	NA
DMRcontig18315:29891201	29891201	29891300	100	1	0.000733581	1	1	NA	NA
DMRcontig18339:29925801	29925801	29926039	239	2	0.000261341	0	0	NA	NA
DMRcontig18359:29952506	29952506	29952800	295	1	3.18E-06	13	4.406779661	NA	NA
DMRcontig18404:30019101	30019101	30019294	194	1	0.000981487	2	1.030927835	NA	NA
DMRcontig18428:30052401	30052401	30052582	182	1	0.000719071	14	7.692307692	NA	NA
DMRcontig18433:30060001	30060001	30060500	500	1	0.000188887	6	1.2	LOC106584716	protease
DMRcontig18451:30085555	30085555	30086000	446	3	0.00014508	12	2.69058296	NA	NA
DMRcontig18464:30105501	30105501	30106551	1051	9	4.20E-05	75	7.136060894	NA	NA
DMRcontig18493:30148901	30148901	30149080	180	2	0.000536627	3	1.666666667	NA	NA
DMRcontig18531:30203101	30203101	30203400	300	1	1.98E-05	12	4	NA	NA
DMRcontig18533:30206501	30206501	30206700	200	1	0.00094233	3	1.5	NA	NA
DMRcontig18560:30244934	30244934	30245200	267	1	0.000945113	4	1.498127341	NA	NA
DMRcontig18561:30246601	30246601	30246782	182	2	0.000499212	3	1.648351648	NA	NA
DMRcontig18580:30274501	30274501	30275361	861	1	0.000905867	19	2.206736353	NA	NA
DMRcontig18588:30286501	30286501	30286818	318	2	3.47E-05	8	2.51572327	NA	NA
DMRcontig18601:30304631	30304631	30304800	170	1	0.000185479	0	0	NA	NA
DMRcontig18629:30343201	30343201	30343481	281	2	0.000298985	6	2.135231317	NA	NA
DMRcontig18703:30446801	30446801	30448600	1800	1	0.000912199	129	7.166666667	NA	NA
DMRcontig18743:30515801	30515801	30516200	400	1	0.000628214	29	7.25	NA	NA
DMRcontig18749:30526401	30526401	30526672	272	1	7.77E-05	17	6.25	NA	NA
DMRcontig18751:30529401	30529401	30529700	300	3	3.41E-06	1	0.333333333	NA	NA
DMRcontig18763:30547686	30547686	30547900	215	1	5.86E-05	0	0	NA	NA
DMRcontig18770:30559401	30559401	30559800	400	1	0.000887063	17	4.25	NA	NA
DMRcontig18803:30610301	30610301	30611400	1100	6	0.000548454	69	6.272727273	NA	NA

DMRcontig18808:30618428	30618428	30618644	217	1	0.000158216	2	0.921658986	NA	NA
DMRcontig18824:30644361	30644361	30644900	540	1	0.000665305	22	4.074074074	NA	NA
DMRcontig18848:30682601	30682601	30683200	600	3	2.40E-05	5	0.833333333	NA	NA
DMRcontig18853:30690601	30690601	30691099	499	1	0.000872864	9	1.803607214	NA	NA
DMRcontig18859:30700068	30700068	30700900	833	3	0.000153765	68	8.163265306	NA	NA
DMRcontig18872:30720634	30720634	30720800	167	2	3.87E-07	8	4.790419162	NA	NA
DMRcontig18926:30804120	30804120	30804600	481	1	0.000190706	3	0.623700624	NA	NA
DMRcontig18952:30843501	30843501	30843636	136	1	9.19E-05	8	5.882352941	NA	NA
DMRcontig18970:30870964	30870964	30871400	437	1	0.000858842	3	0.686498856	NA	NA
DMRcontig18973:30876032	30876032	30876231	200	3	1.70E-05	2	1	NA	NA
DMRcontig18980:30887301	30887301	30887458	158	1	0.000283898	1	0.632911392	NA	NA
DMRcontig18989:30900144	30900144	30900862	719	1	0.000452735	23	3.198887344	NA	NA
DMRcontig19008:30929501	30929501	30930094	594	1	0.000322368	19	3.198653199	NA	NA
DMRcontig19009:30931101	30931101	30931300	200	1	0.000756298	7	3.5	NA	NA
DMRcontig19031:30964005	30964005	30964200	196	2	0.000297584	7	3.571428571	NA	NA
DMRcontig19036:30972001	30972001	30972200	200	1	0.000385161	4	2	NA	NA
DMRcontig19039:30976301	30976301	30977247	947	3	4.51E-05	62	6.546990496	NA	NA
DMRcontig19048:30989914	30989914	30990300	387	1	0.000444787	15	3.875968992	NA	NA
DMRcontig19055:31000901	31000901	31001068	168	1	0.00024536	2	1.19047619	NA	NA
DMRcontig19087:31047901	31047901	31048100	200	1	0.000100479	6	3	NA	NA
DMRcontig19096:31060755	31060755	31060900	146	1	0.000668756	14	9.589041096	NA	NA
DMRcontig19176:31179401	31179401	31179653	253	1	0.000157304	9	3.557312253	NA	NA
DMRcontig19235:31267301	31267301	31267800	500	1	9.69E-08	35	7	NA	NA
DMRcontig19237:31270848	31270848	31270990	143	2	0.000153501	5	3.496503497	NA	NA
DMRcontig19262:31308253	31308253	31309000	748	1	2.36E-06	24	3.20855615	NA	NA
DMRcontig19283:31340701	31340701	31341100	400	1	0.000869	8	2	NA	NA
DMRcontig19309:31382501	31382501	31382800	300	2	0.000772546	6	2	NA	NA
DMRcontig19361:31458501	31458501	31458800	300	1	0.000412921	10	3.333333333	NA	NA
DMRcontig19365:31464601	31464601	31465200	600	2	5.21E-05	26	4.333333333	NA	NA
DMRcontig19369:31470101	31470101	31470300	200	1	0.000930459	11	5.5	NA	NA
DMRcontig19370:31471637	31471637	31472400	764	1	0.00058576	25	3.272251309	NA	NA
DMRcontig19392:31505201	31505201	31505339	139	1	0.000823707	1	0.71942446	NA	NA
DMRcontig19406:31524501	31524501	31524700	200	1	0.000339993	4	2	NA	NA
DMRcontig19414:31535359	31535359	31535800	442	2	1.54E-06	10	2.262443439	NA	NA
DMRcontig19426:31552675	31552675	31552700	26	1	2.13E-05	0	0	NA	NA
DMRcontig19431:31560601	31560601	31560766	166	2	0.00020018	8	4.819277108	NA	NA
DMRcontig19462:31606731	31606731	31607000	270	2	1.03E-05	13	4.814814815	NA	NA
DMRcontig19497:31658300	31658300	31658476	177	1	3.99E-05	5	2.824858757	NA	NA
DMRcontig19514:31682401	31682401	31683200	800	2	0.000104106	42	5.25	plekha5	signaling
DMRcontig19520:31692901	31692901	31693100	200	1	0.000189207	6	3	NA	NA
DMRcontig19552:31737434	31737434	31738100	667	3	0.000174739	29	4.347826087	NA	NA
DMRcontig19555:31742213	31742213	31742700	488	1	0.000185479	20	4.098360656	NA	NA
DMRcontig19576:31773201	31773201	31773346	146	1	0.000345894	11	7.534246575	NA	NA
DMRcontig19577:31774501	31774501	31774900	400	1	8.74E-05	27	6.75	NA	NA
DMRcontig19590:31792701	31792701	31792973	273	1	0.000616282	17	6.227106227	NA	NA
DMRcontig19604:31812101	31812101	31812400	300	1	0.000520592	9	3	NA	NA
DMRcontig19607:31816901	31816901	31817092	192	2	0.000178862	5	2.604166667	NA	NA
DMRcontig19651:31881043	31881043	31881100	58	1	0.000981487	0	0	NA	NA
DMRcontig19659:31892501	31892501	31892700	200	1	7.19E-05	6	3	NA	NA
DMRcontig19665:31900301	31900301	31900500	200	1	0.000750566	3	1.5	NA	NA
DMRcontig19669:31906401	31906401	31906607	207	1	0.00047333	5	2.415458937	NA	NA
DMRcontig19681:31924301	31924301	31924500	200	1	2.22E-06	16	8	NA	NA
DMRcontig19714:31970801	31970801	31971000	200	1	0.000906014	5	2.5	NA	NA
DMRcontig19728:31991308	31991308	31991849	542	3	0.000185479	11	2.029520295	NA	NA
DMRcontig19754:32030201	32030201	32030500	300	2	4.54E-05	5	1.666666667	NA	NA
DMRcontig19764:32044501	32044501	32044657	157	1	0.000252103	5	3.184713376	NA	NA
DMRcontig19795:32088723	32088723	32089100	378	2	4.49E-07	20	5.291005291	NA	NA
DMRcontig19812:32115101	32115101	32115364	264	2	0.000227712	3	1.136363636	NA	NA
DMRcontig19821:32128483	32128483	32129100	618	1	0.000981518	32	5.177993528	NA	NA
DMRcontig19834:32147443	32147443	32147628	186	3	5.11E-05	11	5.913978495	NA	NA
DMRcontig19862:32188401	32188401	32188575	175	2	9.85E-05	3	1.714285714	NA	NA
DMRcontig19863:32189576	32189576	32189713	138	2	1.37E-05	2	1.449275362	NA	NA
DMRcontig19870:32200601	32200601	32200795	195	2	0.000105355	13	6.666666667	NA	NA
DMRcontig19871:32201796	32201796	32202100	305	1	5.19E-05	2	0.655737705	NA	NA
DMRcontig19899:32244001	32244001	32244259	259	1	0.000678018	10	3.861003861	NA	NA
DMRcontig19917:32268401	32268401	32268700	300	1	1.25E-05	16	5.333333333	NA	NA
DMRcontig19922:32275218	32275218	32275383	166	1	0.000867453	2	1.204819277	NA	NA
DMRcontig19935:32295565	32295565	32295852	288	2	0.000286096	8	2.777777778	NA	NA
DMRcontig19946:32311601	32311601	32311993	393	2	9.46E-05	31	7.888040712	NA	NA
DMRcontig19964:32338901	32338901	32339300	400	1	0.000810312	26	6.5	NA	NA
DMRcontig19968:32345401	32345401	32345886	486	1	0.000730458	29	5.967078189	NA	NA
DMRcontig19988:32374301	32374301	32374500	200	1	0.000693065	21	10.5	NA	NA
DMRcontig19993:32382001	32382001	32382200	200	1	0.000762871	8	4	NA	NA
DMRcontig20010:32408001	32408001	32408300	300	1	0.000135369	19	6.333333333	NA	NA
DMRcontig20021:32425105	32425105	32425267	163	1	0.000508885	8	4.90797546	NA	NA
DMRcontig20037:32449101	32449101	32449760	660	1	0.000984055	20	3.03030303	NA	NA
DMRcontig20044:32459116	32459116	32459400	285	1	0.000383931	0	0	NA	NA
DMRcontig20045:32460801	32460801	32460948	148	1	0.000981487	5	3.378378378	NA	NA
DMRcontig20054:32475001	32475001	32475400	400	3	0.000261305	9	2.25	NA	NA
DMRcontig20060:32484301	32484301	32484600	300	2	1.81E-05	8	2.666666667	NA	NA

DMRcontig20072:32504901	32504901	32505300	400	1	0.000173524	26	6.5	LRP1	receptors and binding proteins
DMRcontig20083:32523001	32523001	32523623	623	5	1.97E-06	30	4.81540931	LOC105888664	epigenetic
DMRcontig20229:32752301	32752301	32753100	800	2	3.22E-05	17	2.125	NA	NA
DMRcontig20252:32788401	32788401	32788700	300	1	0.000733581	7	2.333333333	NA	NA
DMRcontig20280:32830101	32830101	32830600	500	2	4.01E-06	14	2.8	NA	NA
DMRcontig20296:32854849	32854849	32855375	527	3	2.17E-05	33	6.261859583	NA	NA
DMRcontig20332:32907501	32907501	32907700	200	1	0.000194315	7	3.5	NA	NA
DMRcontig20342:32922070	32922070	32922500	431	3	0.000126801	35	8.120649652	NA	NA
DMRcontig20365:32954701	32954701	32954993	293	2	3.48E-06	8	2.730375427	NA	NA
DMRcontig20372:32965001	32965001	32965200	200	1	0.000970801	4	2	NA	NA
DMRcontig20377:32972001	32972001	32972400	400	1	0.000736769	13	3.25	NA	NA
DMRcontig20396:32999001	32999001	32999294	294	1	9.37E-05	9	3.06122449	NA	NA
DMRcontig20401:33006110	33006110	33006436	327	1	0.000528317	8	2.44648318	NA	NA
DMRcontig20407:33014601	33014601	33014797	197	1	0.000914082	4	2.030456853	NA	NA
DMRcontig20431:33048280	33048280	33048500	221	1	1.56E-05	12	5.429864253	NA	NA
DMRcontig20459:33086709	33086709	33087123	415	4	3.09E-05	34	8.192771084	NA	NA
DMRcontig20488:33128401	33128401	33128500	100	1	0.000643662	1	1	NA	NA
DMRcontig20523:33174201	33174201	33174423	223	2	9.21E-06	6	2.69058296	NA	NA
DMRcontig20537:33193201	33193201	33193355	155	1	0.000926327	2	1.290322581	NA	NA
DMRcontig20601:33278001	33278001	33278268	268	2	8.27E-05	7	2.611940299	NA	NA
DMRcontig20610:33289320	33289320	33289500	181	2	4.21E-42	0	0	NA	NA
DMRcontig20618:33299708	33299708	33300000	293	2	1.05E-05	6	2.04778157	NA	NA
DMRcontig20664:33361001	33361001	33361432	432	2	0.000130822	7	1.62037037	NA	NA
DMRcontig20687:33391019	33391019	33391200	182	1	0.000257262	7	3.846153846	NA	NA
DMRcontig20745:33461109	33461109	33461173	65	1	0.00024536	2	3.076923077	NA	NA
DMRcontig20779:33499801	33499801	33501500	1700	4	0.000475894	117	6.882352941	NA	NA
DMRcontig20796:33525501	33525501	33526200	700	6	1.54E-05	42	6	NA	NA
DMRcontig20813:33548801	33548801	33549700	900	3	0.000150011	64	7.111111111	NA	NA
DMRcontig20837:33579622	33579622	33580000	379	2	0.000412998	4	1.055408971	NA	NA
DMRcontig20855:33602701	33602701	33602900	200	1	0.000545	5	2.5	NA	NA
DMRcontig20887:33643901	33643901	33644100	200	2	8.02E-07	0	0	NA	NA
DMRcontig20914:33677901	33677901	33678100	200	2	0.000399928	2	1	NA	NA
DMRcontig20925:33692357	33692357	33692800	444	2	1.25E-05	4	0.900900901	NA	NA
DMRcontig20938:33708301	33708301	33709041	741	5	0.000358503	50	6.747638327	NA	NA
DMRcontig20972:33751572	33751572	33751672	101	1	0.000742601	2	1.98019802	NA	NA
DMRcontig20988:33773377	33773377	33773924	548	2	0.000652101	16	2.919708029	NA	NA
DMRcontig20996:33784178	33784178	33784298	121	2	0.000580276	9	7.438016529	NA	NA
DMRcontig21015:33807701	33807701	33807961	261	1	0.000949208	17	6.513409962	NA	NA
DMRcontig21036:33832301	33832301	33832400	100	1	0.000521849	2	2	ETH_00034195	signaling
DMRcontig21058:33858609	33858609	33858650	42	1	0.000415247	1	2.380952381	NA	NA
DMRcontig21084:33890626	33890626	33890714	89	1	0.000235847	4	4.494382022	NA	NA
DMRcontig21141:33961801	33961801	33962358	558	1	0.000774824	33	5.913978495	NA	NA
DMRcontig21164:33994175	33994175	33994633	459	5	2.25E-05	27	5.882352941	NA	NA
DMRcontig21167:33998892	33998892	33999356	465	5	1.78E-06	27	5.806451613	NA	NA
DMRcontig21187:34028401	34028401	34028876	476	2	0.000154562	34	7.142857143	NA	NA
DMRcontig21203:34051877	34051877	34052320	444	1	0.000403844	25	5.630630631	NA	NA
DMRcontig21204:34053401	34053401	34053700	300	1	0.000756257	14	4.666666667	NA	NA
DMRcontig21208:34059039	34059039	34059500	462	1	0.00068041	27	5.844155844	NA	NA
DMRcontig21229:34089183	34089183	34089300	118	1	0.000328962	6	5.084745763	NA	NA
DMRcontig21468:34350880	34350880	34350938	59	1	0.000868055	0	0	NA	NA
DMRcontig21472:34355256	34355256	34355535	280	1	0.000267008	2	0.714285714	NA	NA
DMRcontig21516:34404470	34404470	34404519	50	2	0.000649369	0	0	NA	NA
DMRcontig21527:34416463	34416463	34416515	53	2	0.000611699	0	0	NA	NA
DMRcontig21587:34482301	34482301	34482412	112	1	0.000461901	0	0	NA	NA
DMRcontig21655:34557201	34557201	34557361	161	2	0.00030793	10	6.211180124	NA	NA
DMRcontig21700:34607401	34607401	34607586	186	1	0.000365042	3	1.612903226	NA	NA
DMRcontig21709:34617729	34617729	34617930	202	2	0.000322449	2	0.99009901	NA	NA
DMRcontig21739:34650701	34650701	34650796	96	1	0.000216772	0	0	NA	NA
DMRcontig21810:34731017	34731017	34731173	157	1	0.000869203	0	0	NA	NA
DMRcontig21845:34771201	34771201	34771337	137	1	0.000713727	2	1.459854015	NA	NA
DMRcontig22042:34994515	34994515	34994619	105	2	1.37E-05	0	0	NA	NA
DMRcontig22055:35009301	35009301	35009424	124	1	0.000959554	3	2.419354839	NA	NA
DMRcontig22183:35157283	35157283	35157361	79	1	0.000311483	0	0	NA	NA
DMRcontig22188:35163038	35163038	35163400	363	2	4.22E-06	2	0.550964187	NA	NA
DMRcontig22243:35225301	35225301	35225500	200	1	0.000177466	2	1	NA	NA
DMRcontig22264:35249501	35249501	35249676	176	1	0.000552999	12	6.818181818	NA	NA
DMRcontig22267:35252801	35252801	35253047	247	1	0.000752611	4	1.619433198	NA	NA
DMRcontig22278:35265701	35265701	35265828	128	1	0.00074383	2	1.5625	NA	NA
DMRcontig22318:35313314	35313314	35313356	43	1	0.000179079	0	0	NA	NA
DMRcontig22323:35319101	35319101	35319300	200	1	0.000362441	3	1.5	NA	NA
DMRcontig22380:35384801	35384801	35384908	108	1	0.000430345	4	3.703703704	NA	NA
DMRcontig22391:35397101	35397101	35397202	102	1	0.000146365	1	0.980392157	NA	NA
DMRcontig22429:35441176	35441176	35441230	55	2	0.000128327	0	0	NA	NA
DMRcontig22440:35453789	35453789	35454000	212	1	3.13E-05	6	2.830188679	NA	NA
DMRcontig22497:35521101	35521101	35521256	156	1	3.10E-06	3	1.923076923	NA	NA
DMRcontig22515:35542474	35542474	35542667	194	1	0.000956127	1	0.515463918	NA	NA
DMRcontig22578:35616284	35616284	35616357	74	1	4.71E-06	2	2.702702703	NA	NA
DMRcontig22604:35647201	35647201	35647390	190	1	0.000336255	1	0.526315789	NA	NA
DMRcontig22608:35652001	35652001	35652100	100	1	0.000471226	4	4	NA	NA
DMRcontig22617:35662928	35662928	35662988	61	1	0.000193978	1	1.639344262	NA	NA

DMRcontig22743:35811487	35811487	35811543	57	2	0.000379436	2	3.50877193	NA	NA
DMRcontig22765:35836768	35836768	35836866	99	1	0.000168658	1	1.01010101	NA	NA
DMRcontig22768:35840301	35840301	35840510	210	2	4.43E-05	7	3.333333333	NA	NA
DMRcontig22773:35846701	35846701	35846868	168	1	0.000658867	0	0	NA	NA
DMRcontig22793:35870201	35870201	35870600	400	2	5.84E-06	11	2.75	NA	NA
DMRcontig22810:35890340	35890340	35890485	146	2	2.79E-05	0	0	NA	NA
DMRcontig22848:35935475	35935475	35935574	100	1	0.000970154	0	0	NA	NA
DMRcontig22872:35963101	35963101	35963400	300	2	6.45E-06	25	8.333333333	NA	NA
DMRcontig22902:35998801	35998801	35998940	140	2	0.000208174	5	3.571428571	NA	NA
DMRcontig22912:36010401	36010401	36010700	300	1	0.000887811	2	0.666666667	NA	NA
DMRcontig22943:36045917	36045917	36046079	163	1	0.000352913	1	0.613496933	NA	NA
DMRcontig22976:36084821	36084821	36085000	180	1	0.000492614	3	1.666666667	NA	NA
DMRcontig22977:36086012	36086012	36086100	89	1	0.000844523	6	6.741573034	NA	NA
DMRcontig23010:36125301	36125301	36125398	98	1	0.000843337	1	1.020408163	NA	NA
DMRcontig23018:36134854	36134854	36134978	125	1	0.000545983	0	0	NA	NA
DMRcontig23020:36137023	36137023	36137300	278	1	0.000414157	6	2.158273381	NA	NA
DMRcontig23029:36147043	36147043	36147210	168	1	1.47E-06	3	1.785714286	NA	NA
DMRcontig23049:36170519	36170519	36170800	282	1	0.000288919	6	2.127659574	NA	NA
DMRcontig23088:36217701	36217701	36217900	200	1	0.000251658	2	1	NA	NA
DMRcontig23099:36230501	36230501	36230656	156	1	0.000860249	12	7.692307692	NA	NA
DMRcontig23165:36306733	36306733	36306796	64	1	0.000921646	7	10.9375	NA	NA
DMRcontig23201:36348301	36348301	36348500	200	1	0.000492771	9	4.5	LOC105448499	development
DMRcontig23299:36462638	36462638	36462815	178	3	5.07E-05	0	0	NA	NA
DMRcontig23337:36507090	36507090	36507187	98	1	0.000454536	0	0	NA	NA
DMRcontig23343:36513801	36513801	36514089	289	2	4.74E-05	5	1.730103806	NA	NA
DMRcontig23373:36549601	36549601	36549965	365	2	6.55E-05	22	6.02739726	NA	NA
DMRcontig23492:36687088	36687088	36687130	43	2	0.000432843	0	0	NA	NA
DMRcontig23515:36713601	36713601	36713709	109	1	0.000437172	3	2.752293578	NA	NA
DMRcontig23589:36798536	36798536	36798724	189	3	0.000112457	4	2.116402116	NA	NA
DMRcontig23622:36836519	36836519	36836915	397	4	1.24E-05	37	9.319899244	NA	NA
DMRcontig23630:36846181	36846181	36846266	86	2	1.85E-05	2	2.325581395	NA	NA
DMRcontig23683:36906901	36906901	36907154	254	1	0.000900432	4	1.57480315	NA	NA
DMRcontig23729:36960401	36960401	36960650	250	1	0.000609488	16	6.4	NA	NA
DMRcontig23744:36977301	36977301	36977424	124	1	0.000670615	4	3.225806452	NA	NA
DMRcontig23767:37003172	37003172	37003224	53	2	0.000123987	1	1.886792453	NA	NA
DMRcontig23797:37036901	37036901	37037020	120	1	0.000899844	2	1.666666667	NA	NA
DMRcontig23852:37098700	37098700	37098900	201	2	0.000114267	11	5.472636816	NA	NA
DMRcontig23875:37124938	37124938	37125001	64	1	5.13E-05	1	1.5625	NA	NA
DMRcontig23892:37145301	37145301	37145560	260	1	7.16E-05	8	3.076923077	NA	NA
DMRcontig23895:37148862	37148862	37148911	50	1	0.000651453	0	0	NA	NA
DMRcontig23923:37181301	37181301	37181386	86	1	0.0006166	0	0	NA	NA
DMRcontig23929:37187801	37187801	37187956	156	2	0.000296352	9	5.769230769	NA	NA
DMRcontig23989:37254379	37254379	37254418	40	2	0.000631062	0	0	NA	NA
DMRcontig24018:37293545	37293545	37294064	520	1	0.000516423	21	4.038461538	COX1;ATP8	metabolism and transport
DMRcontig24051:37347901	37347901	37348546	646	2	0.000234754	32	4.953560372	NA	NA
DMRcontig24093:37415101	37415101	37415264	164	1	0.000962954	1	0.609756098	NA	NA
DMRcontig24105:37432811	37432811	37433320	510	1	7.50E-05	8	1.568627451	NA	NA
DMRcontig24106:37434701	37434701	37434989	289	1	0.000875707	16	5.53633218	NA	NA
DMRcontig24120:37456901	37456901	37457100	200	1	0.000152462	3	1.5	NA	NA
DMRcontig24123:37461601	37461601	37462000	400	1	0.000733581	10	2.5	NA	NA
DMRcontig24132:37475401	37475401	37475600	200	1	0.000146081	3	1.5	NA	NA
DMRcontig24152:37505701	37505701	37505900	200	1	0.000223205	5	2.5	NA	NA
DMRcontig24164:37524073	37524073	37524442	370	1	0.000264224	13	3.513513514	NA	NA
DMRcontig24178:37544401	37544401	37544600	200	1	0.00022338	6	3	NA	NA
DMRcontig24211:37592801	37592801	37593100	300	1	8.70E-06	32	10.66666667	NA	NA
DMRcontig24213:37595801	37595801	37595974	174	1	0.000423872	2	1.149425287	NA	NA
DMRcontig24216:37600056	37600056	37600400	345	3	2.55E-07	15	4.347826087	NA	NA
DMRcontig24243:37640001	37640001	37640300	300	1	0.000910322	1	0.333333333	NA	NA
DMRcontig24273:37683701	37683701	37683900	200	1	3.43E-05	5	2.5	NA	NA
DMRcontig24323:37755301	37755301	37755500	200	2	0.000340959	13	6.5	NA	NA
DMRcontig24341:37780001	37780001	37780400	400	1	0.00027652	13	3.25	NA	NA
DMRcontig24351:37794107	37794107	37794951	845	9	1.17E-06	36	4.26035503	LOC105888664	epigenetic
DMRcontig24393:37852101	37852101	37852900	800	6	0.000388659	59	7.375	NA	NA
DMRcontig24419:37888742	37888742	37889100	359	1	0.000763386	3	0.835654596	NA	NA
DMRcontig24492:37986301	37986301	37986584	284	1	4.70E-05	7	2.464788732	NA	NA
DMRcontig24530:38035701	38035701	38035819	119	1	2.02E-05	0	0	NA	NA
DMRcontig24552:38064011	38064011	38064152	142	1	1.97E-05	1	0.704225352	NA	NA
DMRcontig24562:38077162	38077162	38077381	220	2	0.000538376	6	2.727272727	NA	NA
DMRcontig24653:38192130	38192130	38193275	1146	9	9.65E-05	73	6.369982548	NA	NA
DMRcontig24655:38195701	38195701	38196669	969	1	0.000799316	67	6.914344685	NA	NA
DMRcontig24658:38200501	38200501	38200700	200	1	0.000297014	15	7.5	NA	NA
DMRcontig24681:38229339	38229339	38230117	779	5	0.000272985	58	7.445442875	NA	NA
DMRcontig24702:38256829	38256829	38257400	572	3	0.000414387	45	7.867132867	NA	NA
DMRcontig24714:38271731	38271731	38272200	470	4	6.77E-05	29	6.170212766	NA	NA
DMRcontig24767:38338153	38338153	38338340	188	3	0.000211799	15	7.978723404	NA	NA
DMRcontig24782:38356967	38356967	38357400	434	3	6.82E-05	31	7.142857143	NA	NA
DMRcontig24810:38393073	38393073	38393200	128	2	0.000504836	6	4.6875	NA	NA
DMRcontig24831:38418794	38418794	38418902	109	1	0.000713788	1	0.917431193	NA	NA
DMRcontig24839:38429280	38429280	38429500	221	1	0.000465091	16	7.239819005	NA	NA
DMRcontig24841:38432005	38432005	38432200	196	1	0.000996509	0	0	NA	NA

DMRcontig24852:38445029	38445029	38445137	109	1	0.000511295	9	8.256880734	NA	NA
DMRcontig24868:38465644	38465644	38465737	94	2	0.000143602	0	0	NA	NA
DMRcontig24892:38493801	38493801	38494191	391	1	0.000643388	30	7.672634271	NA	NA
DMRcontig24895:38497716	38497716	38497900	185	2	0.000552245	6	3.243243243	NA	NA
DMRcontig24934:38545501	38545501	38545831	331	1	0.000910705	11	3.32326284	NA	NA
DMRcontig24954:38569549	38569549	38570154	606	1	0.000911787	42	6.930693069	NA	NA
DMRcontig24967:38588164	38588164	38588800	637	2	0.000773144	41	6.436420722	NA	NA
DMRcontig25004:38642263	38642263	38642800	538	2	0.000515874	36	6.691449814	NA	NA
DMRcontig25026:38671502	38671502	38671619	118	1	0.000499594	1	0.847457627	NA	NA
DMRcontig25155:38809701	38809701	38809866	166	2	0.000170105	3	1.807228916	NA	NA
DMRcontig25244:38907301	38907301	38907359	59	1	0.000751062	2	3.389830508	NA	NA
DMRcontig25251:38915201	38915201	38915305	105	2	2.48E-05	8	7.619047619	NA	NA
DMRcontig25252:38916306	38916306	38916435	130	2	0.000836364	1	0.769230769	NA	NA
DMRcontig25286:38953596	38953596	38953699	104	1	0.000631678	6	5.769230769	NA	NA
DMRcontig25288:38955779	38955779	38955840	62	1	0.000666619	1	1.612903226	NA	NA
DMRcontig25295:38963255	38963255	38963400	146	2	0.000206062	0	0	NA	NA
DMRcontig25374:39050674	39050674	39050800	127	1	0.000565923	4	3.149606299	NA	NA
DMRcontig25375:39051993	39051993	39052033	41	1	0.000703297	0	0	NA	NA
DMRcontig25388:39066129	39066129	39066200	72	1	0.00085586	0	0	NA	NA
DMRcontig25403:39082855	39082855	39083000	146	2	1.62E-06	3	2.054794521	NA	NA
DMRcontig25431:39114501	39114501	39114800	300	1	0.000436687	22	7.333333333	LEMA_P124570.1;LEMA_P124550.1	signaling
DMRcontig25467:39154163	39154163	39154271	109	2	0.000276696	10	9.174311927	NA	NA
DMRcontig25486:39174901	39174901	39175300	400	2	9.02E-05	14	3.5	NA	NA
DMRcontig25492:39182050	39182050	39182096	47	1	0.000717843	1	2.127659574	NA	NA
DMRcontig25503:39194601	39194601	39194728	128	1	0.000908103	3	2.34375	NA	NA
DMRcontig25519:39213164	39213164	39213284	121	1	0.000613456	0	0	NA	NA
DMRcontig25562:39262401	39262401	39262534	134	2	0.000760207	5	3.731343284	NA	NA
DMRcontig25564:39265301	39265301	39265465	165	1	0.000918643	12	7.272727273	NA	NA
DMRcontig25582:39285505	39285505	39285609	105	1	0.000314973	5	4.761904762	NA	NA
DMRcontig25584:39287701	39287701	39287924	224	2	0.000253584	8	3.571428571	NA	NA
DMRcontig25587:39291312	39291312	39291550	239	1	0.000440253	8	3.347280335	NA	NA
DMRcontig25597:39302718	39302718	39302800	83	1	0.000837956	1	1.204819277	NA	NA
DMRcontig25673:39390571	39390571	39390648	78	2	0.000526802	0	0	NA	NA
DMRcontig25675:39392724	39392724	39392900	177	1	8.90E-05	13	7.344632768	NA	NA
DMRcontig25699:39421670	39421670	39421900	231	2	0.000192561	9	3.896103896	NA	NA
DMRcontig25705:39428501	39428501	39428651	151	1	0.000714603	1	0.662251656	NA	NA
DMRcontig25718:39443375	39443375	39443658	284	1	0.000859607	11	3.873239437	NA	NA
DMRcontig25726:39453462	39453462	39453541	80	2	9.89E-05	0	0	NA	NA
DMRcontig25773:39507298	39507298	39507500	203	1	0.000285978	6	2.955665025	NA	NA
DMRcontig25783:39518747	39518747	39518860	114	1	3.27E-05	5	4.385964912	NA	NA
DMRcontig25784:39519901	39519901	39520120	220	1	0.000767257	6	2.727272727	NA	NA
DMRcontig25802:39541034	39541034	39541100	67	1	0.000831914	1	1.492537313	NA	NA
DMRcontig25821:39562801	39562801	39563059	259	3	5.97E-07	9	3.474903475	NA	NA
DMRcontig25823:39565313	39565313	39565431	119	1	0.000712659	2	1.680672269	NA	NA
DMRcontig25893:39644501	39644501	39644592	92	1	0.000170233	0	0	NA	NA
DMRcontig25919:39674931	39674931	39675372	442	4	1.61E-07	40	9.049773756	NA	NA
DMRcontig25939:39698501	39698501	39698628	128	1	0.000535808	3	2.34375	NA	NA
DMRcontig25958:39719901	39719901	39720253	353	1	0.000813679	12	3.399433428	NA	NA
DMRcontig25961:39723575	39723575	39723726	152	2	8.72E-05	1	0.657894737	NA	NA
DMRcontig25967:39730801	39730801	39730903	103	1	0.000648876	4	3.883495146	NA	NA
DMRcontig26030:39805174	39805174	39805351	178	1	3.21E-05	4	2.247191011	NA	NA
DMRcontig26048:39826901	39826901	39827079	179	2	0.000127417	11	6.145251397	NA	NA
DMRcontig26059:39840601	39840601	39840734	134	1	0.000120031	0	0	NA	NA
DMRcontig26127:39922601	39922601	39923000	400	1	0.000739381	19	4.75	NA	NA
DMRcontig26132:39928722	39928722	39929000	279	1	1.10E-05	10	3.584229391	NA	NA
DMRcontig26211:40023101	40023101	40023278	178	2	0.000116023	2	1.123595506	NA	NA
DMRcontig26235:40051541	40051541	40051593	53	1	0.000343508	1	1.886792453	NA	NA
DMRcontig26251:40070123	40070123	40070163	41	1	9.99E-05	0	0	NA	NA
DMRcontig26255:40075301	40075301	40075757	457	2	5.58E-05	19	4.157549234	NA	NA
DMRcontig26268:40090601	40090601	40090827	227	2	0.000616282	7	3.083700441	NA	NA
DMRcontig26275:40099201	40099201	40099338	138	1	0.000757384	4	2.898550725	NA	NA
DMRcontig26289:40115453	40115453	40115666	214	1	0.000956569	4	1.869158879	NA	NA
DMRcontig26323:40155401	40155401	40155730	330	2	8.06E-05	9	2.727272727	NA	NA
DMRcontig26339:40174955	40174955	40175017	63	2	0.000591224	0	0	NA	NA
DMRcontig26425:40276023	40276023	40276317	295	1	0.00021336	3	1.016949153	NA	NA
DMRcontig26444:40298618	40298618	40298800	183	2	0.000203605	6	3.278688525	NA	NA
DMRcontig26495:40358101	40358101	40358224	124	2	0.000150161	2	1.612903226	NA	NA
DMRcontig26498:40361365	40361365	40361465	101	1	0.000962603	6	5.940594059	NA	NA
DMRcontig26512:40377341	40377341	40377500	160	2	0.000287806	5	3.125	NA	NA
DMRcontig26525:40392122	40392122	40392196	75	1	3.76E-05	0	0	NA	NA
DMRcontig26571:40445704	40445704	40445757	54	1	0.000994081	0	0	NA	NA
DMRcontig26589:40467201	40467201	40467302	102	1	0.000298674	5	4.901960784	NA	NA
DMRcontig26701:40598255	40598255	40598342	88	1	0.000223205	0	0	NA	NA
DMRcontig26741:40644837	40644837	40644989	153	1	0.000846715	2	1.307189542	NA	NA
DMRcontig26750:40655101	40655101	40655246	146	1	0.000189382	2	1.369863014	NA	NA
DMRcontig26758:40664417	40664417	40664485	69	1	0.000944264	2	2.898550725	NA	NA
DMRcontig26773:40681801	40681801	40681910	110	2	1.46E-07	7	6.363636364	NA	NA
DMRcontig26839:40759927	40759927	40760155	229	1	0.000187577	2	0.873362445	NA	NA
DMRcontig26840:40761201	40761201	40761511	311	2	4.13E-05	17	5.466237942	NA	NA

DMRcontig26912:40846901	40846901	40847117	217	1	0.000929093	3	1.382488479	NA	NA
DMRcontig26917:40853023	40853023	40853255	233	2	3.73E-05	4	1.716738197	NA	NA
DMRcontig26930:40868700	40868700	40868800	101	2	0.000186529	2	1.98019802	NA	NA
DMRcontig26948:40890401	40890401	40890525	125	1	0.000235783	4	3.2	NA	NA
DMRcontig26963:40908701	40908701	40908843	143	2	0.000464123	5	3.496503497	NA	NA
DMRcontig26995:40945401	40945401	40945663	263	1	0.000788617	4	1.520912548	NA	NA
DMRcontig27014:40967829	40967829	40968243	415	2	9.11E-06	13	3.13253012	NA	NA
DMRcontig27025:40980144	40980144	40980398	255	3	1.93E-05	9	3.529411765	NA	NA
DMRcontig27026:40981501	40981501	40981640	140	1	5.17E-05	0	0	NA	NA
DMRcontig27090:41056401	41056401	41056472	72	1	8.63E-05	3	4.166666667	NA	NA
DMRcontig27098:41065336	41065336	41065444	109	2	0.000430076	3	2.752293578	NA	NA
DMRcontig27117:41087145	41087145	41087343	199	2	3.02E-06	7	3.51758794	NA	NA
DMRcontig27218:41204901	41204901	41205004	104	1	0.000448412	2	1.923076923	NA	NA
DMRcontig27284:41278876	41278876	41278925	50	2	0.000550699	4	8	NA	NA
DMRcontig27382:41423401	41423401	41423800	400	1	0.000146081	4	1	NA	NA
DMRcontig27398:41451101	41451101	41451625	525	2	6.38E-05	21	4	NA	NA
DMRcontig27405:41461856	41461856	41462300	445	1	0.000767075	9	2.02247191	NA	NA
DMRcontig27437:41513001	41513001	41514200	1200	1	0.00048894	21	1.75	NA	NA
DMRcontig27540:41674401	41674401	41675058	658	1	0.000125946	14	2.127659574	NA	NA
DMRcontig27543:41679109	41679109	41679447	339	1	0.000671771	19	5.604719764	NA	NA
DMRcontig27562:41707501	41707501	41707700	200	1	0.000108317	26	13	NA	NA
DMRcontig27588:41746401	41746401	41746600	200	1	0.000139136	7	3.5	NA	NA
DMRcontig27595:41756046	41756046	41756500	455	2	1.84E-06	18	3.956043956	NA	NA
DMRcontig27660:41852201	41852201	41852403	203	2	1.39E-07	14	6.896551724	NA	NA
DMRcontig27664:41858501	41858501	41858700	200	1	6.00E-05	2	1	NA	NA
DMRcontig27668:41864001	41864001	41864283	283	1	0.000867532	10	3.533568905	NA	NA
DMRcontig27701:41913901	41913901	41914355	455	2	5.87E-07	24	5.274725275	NA	NA
DMRcontig27755:41991634	41991634	41991925	292	2	0.000260378	19	6.506849315	NA	NA
DMRcontig27759:41996802	41996802	41997200	399	1	0.000813845	7	1.754385965	NA	NA
DMRcontig27769:42010676	42010676	42010972	297	1	0.000192114	24	8.080808081	NA	NA
DMRcontig27777:42022201	42022201	42022400	200	1	2.13E-05	19	9.5	NA	NA
DMRcontig27803:42058901	42058901	42059030	130	2	0.000146792	1	0.769230769	NA	NA
DMRcontig27823:42087701	42087701	42087900	200	1	0.00032364	2	1	NA	NA
DMRcontig27824:42089701	42089701	42089878	178	1	8.43E-05	2	1.123595506	NA	NA
DMRcontig27839:42110501	42110501	42110648	148	2	0.000740355	1	0.675675676	NA	NA
DMRcontig27844:42117801	42117801	42118442	642	3	0.000146604	3	0.46728972	NA	NA
DMRcontig27870:42153801	42153801	42154100	300	1	1.76E-05	26	8.666666667	NA	NA
DMRcontig27883:42172378	42172378	42172800	423	2	2.13E-05	21	4.964539007	NA	NA
DMRcontig27901:42198301	42198301	42198700	400	2	8.84E-05	11	2.75	NA	NA
DMRcontig27909:42210001	42210001	42210141	141	1	0.000336249	1	0.709219858	NA	NA
DMRcontig27916:42219701	42219701	42219804	104	2	0.000165751	2	1.923076923	NA	NA
DMRcontig27930:42239001	42239001	42239148	148	2	7.90E-05	10	6.756756757	NA	NA
DMRcontig27950:42264906	42264906	42265298	393	2	0.000194157	12	3.053435115	NA	NA
DMRcontig27976:42300701	42300701	42301150	450	1	5.85E-05	11	2.444444444	NA	NA
DMRcontig27977:42302151	42302151	42302523	373	3	8.34E-05	9	2.412868633	NA	NA
DMRcontig27981:42307401	42307401	42307534	134	2	0.000199563	0	0	NA	NA
DMRcontig28025:42364807	42364807	42364999	193	1	0.000405587	3	1.554404145	NA	NA
DMRcontig28062:42412901	42412901	42413100	200	1	0.00088423	6	3	NA	NA
DMRcontig28075:42429380	42429380	42429444	65	2	0.000363187	1	1.538461538	NA	NA
DMRcontig28086:42442715	42442715	42442775	61	1	1.41E-24	1	1.639344262	NA	NA
DMRcontig28091:42448301	42448301	42448500	200	1	0.000618451	2	1	NA	NA
DMRcontig28142:42511639	42511639	42512265	627	2	0.00066875	43	6.858054226	NA	NA
DMRcontig28170:42552206	42552206	42552676	471	5	2.94E-05	28	5.944798301	NA	NA
DMRcontig28177:42561971	42561971	42562817	847	2	0.000339789	13	1.534828808	NA	NA
DMRcontig28188:42577079	42577079	42577800	722	2	0.000611115	52	7.202216066	NA	NA
DMRcontig28204:42599701	42599701	42600089	389	1	0.000290302	5	1.285347044	NA	NA
DMRcontig28212:42610806	42610806	42611336	531	2	0.000191041	39	7.344632768	NA	NA
DMRcontig28241:42649301	42649301	42649690	390	2	0.000101382	23	5.897435897	NA	NA
DMRcontig28253:42665790	42665790	42665900	111	1	0.000395228	0	0	NA	NA
DMRcontig28261:42676736	42676736	42677200	465	4	2.66E-06	3	0.64516129	NA	NA
DMRcontig28285:42710101	42710101	42710300	200	2	0.000362249	1	0.5	NA	NA
DMRcontig28333:42773629	42773629	42773804	176	2	0.000120498	1	0.568181818	NA	NA
DMRcontig28345:42789501	42789501	42789652	152	1	0.000856154	3	1.973684211	NA	NA
DMRcontig28363:42813901	42813901	42814100	200	1	0.000969055	7	3.5	NA	NA
DMRcontig28370:42822126	42822126	42822293	168	2	0.000209538	0	0	NA	NA
DMRcontig28389:42845701	42845701	42846300	600	3	0.000446932	5	0.833333333	NA	NA
DMRcontig28426:42892951	42892951	42893072	122	1	0.000744837	0	0	NA	NA
DMRcontig28429:42896201	42896201	42896478	278	2	0.000483803	9	3.237410072	NA	NA
DMRcontig28460:42932501	42932501	42932617	117	1	0.00086975	9	7.692307692	NA	NA
DMRcontig28493:42972654	42972654	42972792	139	1	7.41E-05	3	2.158273381	NA	NA
DMRcontig28546:43043301	43043301	43043950	650	1	0.00065005	50	7.692307692	NA	NA
DMRcontig28570:43080801	43080801	43080861	61	1	0.000490345	1	1.639344262	NA	NA
DMRcontig28586:43103569	43103569	43103900	332	3	2.20E-05	17	5.120481928	NA	NA
DMRcontig28597:43120701	43120701	43121304	604	1	0.000599198	32	5.298013245	NA	NA
DMRcontig28613:43145253	43145253	43145834	582	2	0.000147781	39	6.701030928	NA	NA
DMRcontig28616:43150101	43150101	43150958	858	1	8.87E-05	29	3.37995338	NA	NA
DMRcontig28623:43161201	43161201	43161800	600	1	0.000452081	6	1	NA	NA
DMRcontig28650:43202701	43202701	43203149	449	1	0.000538866	26	5.79064588	BRAFLDRAFT_288811	unknown
DMRcontig28659:43216601	43216601	43217000	400	4	1.63E-07	6	1.5	NA	NA
DMRcontig28660:43218401	43218401	43218772	372	2	2.05E-05	10	2.688172043	NA	NA

DMRcontig28672:43235973	43235973	43236400	428	2	0.000754035	6	1.401869159	NA	NA
DMRcontig28680:43247406	43247406	43247800	395	1	0.000493633	12	3.037974684	NA	NA
DMRcontig28693:43263901	43263901	43264088	188	1	0.000577093	3	1.595744681	NA	NA
DMRcontig28701:43272455	43272455	43272543	89	2	0.000205212	8	8.988764045	NA	NA
DMRcontig28836:43417972	43417972	43418195	224	1	0.000908044	2	0.892857143	NA	NA
DMRcontig28851:43434801	43434801	43435141	341	3	0.000336767	11	3.225806452	NA	NA
DMRcontig28886:43474901	43474901	43475000	100	1	0.00054373	2	2	NA	NA
DMRcontig28928:43521244	43521244	43521299	56	1	0.000881453	0	0	NA	NA
DMRcontig28936:43530225	43530225	43530400	176	1	1.71E-05	2	1.136363636	NA	NA
DMRcontig28961:43558141	43558141	43558420	280	2	2.09E-06	14	5	NA	NA
DMRcontig28974:43573301	43573301	43573359	59	1	0.00028874	5	8.474576271	NA	NA
DMRcontig28979:43578627	43578627	43578703	77	2	0.000427987	1	1.298701299	NA	NA
DMRcontig28985:43585101	43585101	43585400	300	1	0.000960677	28	9.333333333	NA	NA
DMRcontig28988:43589179	43589179	43589498	320	2	0.000491871	13	4.0625	NA	NA
DMRcontig29070:43680577	43680577	43680997	421	1	0.000807927	6	1.425178147	NA	NA
DMRcontig29074:43685479	43685479	43685597	119	2	0.000105212	0	0	NA	NA
DMRcontig29081:43693674	43693674	43693800	127	1	0.000501398	6	4.724409449	NA	NA
DMRcontig29128:43747014	43747014	43747098	85	1	0.000610706	1	1.176470588	NA	NA
DMRcontig29199:43829073	43829073	43829147	75	2	0.000499979	3	4	NA	NA
DMRcontig29247:43884001	43884001	43884168	168	1	0.000326258	2	1.19047619	NA	NA
DMRcontig29286:43928527	43928527	43928700	174	2	1.65E-06	11	6.32183908	NA	NA
DMRcontig29289:43932101	43932101	43932185	85	1	0.000876499	1	1.176470588	NA	NA
DMRcontig29308:43953073	43953073	43953135	63	1	0.000937565	1	1.587301587	NA	NA
DMRcontig29315:43961001	43961001	43961167	167	2	0.000148101	5	2.994011976	NA	NA
DMRcontig29322:43968981	43968981	43969200	220	1	5.71E-06	6	2.727272727	NA	NA
DMRcontig29404:44062101	44062101	44062365	265	2	0.000260554	1	0.377358491	NA	NA
DMRcontig29468:44135629	44135629	44136064	436	1	0.000968374	10	2.293577982	NA	NA
DMRcontig29478:44147801	44147801	44147937	137	1	0.000677766	3	2.189781022	NA	NA
DMRcontig29514:44190501	44190501	44190626	126	1	0.000611561	4	3.174603175	NA	NA
DMRcontig29598:44291147	44291147	44291300	154	1	0.000406278	2	1.298701299	NA	NA
DMRcontig29616:44312365	44312365	44312700	336	1	0.000736221	14	4.166666667	NA	NA
DMRcontig29619:44316301	44316301	44316419	119	1	0.000731695	4	3.361344538	NA	NA
DMRcontig29629:44327959	44327959	44328098	140	1	0.000988276	2	1.428571429	NA	NA
DMRcontig29633:44332511	44332511	44332600	90	1	0.000232479	0	0	NA	NA
DMRcontig29671:44376011	44376011	44376100	90	1	0.000510285	8	8.888888889	NA	NA
DMRcontig29689:44397334	44397334	44397542	209	1	0.000389487	0	0	NA	NA
DMRcontig29719:44432494	44432494	44432536	43	2	0.000394935	0	0	NA	NA
DMRcontig29721:44434601	44434601	44434800	200	2	4.52E-05	1	0.5	NA	NA
DMRcontig29762:44483301	44483301	44483588	288	2	0.000240968	11	3.819444444	NA	NA
DMRcontig29779:44504338	44504338	44504500	163	1	0.000302206	4	2.45398773	NA	NA
DMRcontig29791:44519501	44519501	44519649	149	1	0.000363836	4	2.684563758	NA	NA
DMRcontig29802:44532303	44532303	44532407	105	1	0.000590203	1	0.952380952	NA	NA
DMRcontig29820:44553411	44553411	44553859	449	2	0.000139991	24	5.345211581	NA	NA
DMRcontig29839:44576253	44576253	44576400	148	1	3.46E-06	5	3.378378378	NA	NA
DMRcontig29864:44606001	44606001	44606143	143	1	0.000856773	7	4.895104895	NA	NA
DMRcontig29878:44621580	44621580	44621793	214	2	0.000198144	1	0.46728972	NA	NA
DMRcontig29929:44681699	44681699	44681946	248	1	0.00070238	1	0.403225806	NA	NA
DMRcontig29944:44699801	44699801	44700200	400	2	0.000253211	29	7.25	NA	NA
DMRcontig29964:44723044	44723044	44723172	129	1	0.000376373	0	0	NA	NA
DMRcontig29977:44737682	44737682	44737881	200	1	5.19E-05	7	3.5	NA	NA
DMRcontig29989:44751951	44751951	44752079	129	1	0.000949422	3	2.325581395	NA	NA
DMRcontig30032:44802701	44802701	44802843	143	1	5.38E-05	8	5.594405594	NA	NA
DMRcontig30035:44806042	44806042	44806454	413	1	0.000230995	16	3.87409201	NA	NA
DMRcontig30063:44839151	44839151	44839194	44	1	0.000259623	1	2.272727273	NA	NA
DMRcontig30064:44840195	44840195	44840400	206	1	0.000738522	6	2.912621359	Gm31501;ATG4C	miscellaneous
DMRcontig30065:44841565	44841565	44841630	66	2	0.000761696	0	0	NA	NA
DMRcontig30070:44847477	44847477	44847631	155	1	2.53E-05	3	1.935483871	NA	NA
DMRcontig30075:44853516	44853516	44853588	73	1	0.00016486	2	2.739726027	NA	NA
DMRcontig30076:44854901	44854901	44855041	141	1	0.000694188	5	3.546099291	NA	NA
DMRcontig30101:44884572	44884572	44884800	229	1	0.000145033	1	0.436681223	NA	NA
DMRcontig30121:44909701	44909701	44909837	137	1	0.000617298	6	4.379562044	NA	NA
DMRcontig30164:44960901	44960901	44961036	136	1	0.000891515	4	2.941176471	NA	NA
DMRcontig30199:45003529	45003529	45003913	385	2	0.000317472	12	3.116883117	NA	NA
DMRcontig30229:45039701	45039701	45039940	240	1	0.000970239	13	5.416666667	NA	NA
DMRcontig30256:45070613	45070613	45070687	75	1	0.0009017	0	0	NA	NA
DMRcontig30260:45075255	45075255	45075315	61	2	2.60E-05	1	1.639344262	NA	NA
DMRcontig30288:45108601	45108601	45108770	170	1	0.000317711	6	3.529411765	NA	NA
DMRcontig30318:45145419	45145419	45145484	66	1	0.000780799	0	0	NA	NA
DMRcontig30322:45150069	45150069	45150492	424	2	2.09E-05	26	6.132075472	NA	NA
DMRcontig30355:45189201	45189201	45189331	131	1	0.000711451	0	0	NA	NA
DMRcontig30365:45201401	45201401	45201537	137	1	0.000462171	4	2.919708029	NA	NA
DMRcontig30379:45218572	45218572	45218646	75	2	0.000245058	0	0	NA	NA
DMRcontig30431:45281858	45281858	45281902	45	1	0.000677607	1	2.222222222	NA	NA
DMRcontig30433:45283946	45283946	45284006	61	1	0.000109248	0	0	NA	NA
DMRcontig30469:45326707	45326707	45326780	74	1	0.000455039	2	2.702702703	NA	NA
DMRcontig30501:45365509	45365509	45365700	192	2	0.000243828	5	2.604166667	NA	NA
DMRcontig30524:45393101	45393101	45393228	128	1	0.000648457	2	1.5625	NA	NA
DMRcontig30565:45441001	45441001	45441183	183	2	2.13E-05	11	6.010928962	NA	NA
DMRcontig30571:45448101	45448101	45448252	152	1	0.000956931	0	0	NA	NA
DMRcontig30574:45451978	45451978	45452200	223	1	0.000718708	11	4.932735426	NA	NA

DMRcontig30594:45476801	45476801	45476937	137	2	6.66E-06	1	0.729927007	NA	NA
DMRcontig30598:45481211	45481211	45481359	149	1	7.66E-05	5	3.355704698	NA	NA
DMRcontig30605:45489522	45489522	45489700	179	2	1.97E-05	11	6.145251397	NA	NA
DMRcontig30618:45505306	45505306	45505600	295	1	0.000983627	6	2.033898305	NA	NA
DMRcontig30649:45542421	45542421	45542528	108	1	0.000810309	1	0.925925926	NA	NA
DMRcontig30661:45556368	45556368	45556411	44	2	7.75E-05	0	0	NA	NA
DMRcontig30678:45576805	45576805	45577100	296	1	0.000977337	13	4.391891892	NA	NA
DMRcontig30688:45588144	45588144	45588443	300	2	0.000835591	15	5	NA	NA
DMRcontig30697:45599221	45599221	45599290	70	1	0.000648255	2	2.857142857	NA	NA
DMRcontig30706:45610001	45610001	45610184	184	1	0.000790763	8	4.347826087	NA	NA
DMRcontig30709:45613530	45613530	45613800	271	1	5.35E-05	2	0.73800738	NA	NA
DMRcontig30725:45633101	45633101	45633352	252	1	0.000162507	15	5.952380952	NA	NA
DMRcontig30748:45661185	45661185	45661277	93	1	3.10E-05	3	3.225806452	NA	NA
DMRcontig30767:45684283	45684283	45684349	67	2	0.000170277	0	0	NA	NA
DMRcontig30770:45687875	45687875	45688266	392	1	0.000929807	22	5.612244898	NA	NA
DMRcontig30774:45692716	45692716	45692928	213	1	0.000601807	6	2.816901408	NA	NA
DMRcontig30779:45698908	45698908	45699240	333	4	6.72E-06	10	3.003003003	NA	NA
DMRcontig30791:45713755	45713755	45713796	42	1	0.000535994	0	0	NA	NA
DMRcontig30807:45733101	45733101	45733234	134	1	0.000436168	3	2.23880597	NA	NA
DMRcontig30808:45734235	45734235	45734318	84	1	0.000614229	1	1.19047619	NA	NA
DMRcontig30819:45747201	45747201	45747330	130	2	0.000606645	3	2.307692308	NA	NA
DMRcontig30826:45755201	45755201	45755585	385	2	0.000155622	6	1.558441558	NA	NA
DMRcontig30834:45765218	45765218	45765267	50	1	0.000705536	0	0	NA	NA
DMRcontig30842:45774473	45774473	45774518	46	2	0.000769577	0	0	NA	NA
DMRcontig30866:45803039	45803039	45803257	219	2	6.97E-05	6	2.739726027	NA	NA
DMRcontig30919:45864899	45864899	45865161	263	2	0.000167772	9	3.422053232	NA	NA
DMRcontig30923:45869501	45869501	45869677	177	1	0.000806958	6	3.389830508	NA	NA
DMRcontig30932:45879969	45879969	45880036	68	2	0.000511196	3	4.411764706	NA	NA
DMRcontig30944:45893501	45893501	45893667	167	1	0.000566893	6	3.592814371	NA	NA
DMRcontig30951:45902201	45902201	45902325	125	1	0.000731941	0	0	NA	NA
DMRcontig30990:45947532	45947532	45947673	142	1	0.000588894	3	2.112676056	NA	NA
DMRcontig30994:45951963	45951963	45952096	134	1	0.000273613	2	1.492537313	NA	NA
DMRcontig31004:45963601	45963601	45963818	218	1	0.000737997	6	2.752293578	NA	NA
DMRcontig31031:45995553	45995553	45995703	151	1	0.000712466	2	1.324503311	NA	NA
DMRcontig31041:46007301	46007301	46007468	168	1	0.000325567	2	1.19047619	NA	NA
DMRcontig31048:46015206	46015206	46015300	95	1	0.000607259	13	13.68421053	NA	NA
DMRcontig31050:46017819	46017819	46018040	222	1	0.00069568	4	1.801801802	NA	NA
DMRcontig31130:46110301	46110301	46110512	212	1	0.000474854	7	3.301886792	NA	NA
DMRcontig31132:46112901	46112901	46113089	189	1	8.65E-05	3	1.587301587	NA	NA
DMRcontig31140:46125701	46125701	46125900	200	1	0.000374935	14	7	NA	NA
DMRcontig31155:46150801	46150801	46151100	300	2	0.00010879	8	2.666666667	NA	NA
DMRcontig31165:46167301	46167301	46167600	300	3	1.06E-05	10	3.333333333	NA	NA
DMRcontig31212:46241612	46241612	46242169	558	6	4.42E-06	31	5.555555556	LOC105888664	epigenetic
DMRcontig31215:46246210	46246210	46246700	491	1	1.39E-05	20	4.073319756	NA	NA
DMRcontig31255:46307201	46307201	46307400	200	2	0.000313995	13	6.5	NA	NA
DMRcontig31262:46317901	46317901	46318400	500	1	0.000130163	26	5.2	NA	NA
DMRcontig31318:46401901	46401901	46402317	417	1	7.30E-06	12	2.877697842	NA	NA
DMRcontig31341:46435558	46435558	46435800	243	2	0.000407851	2	0.823045267	NA	NA
DMRcontig31347:46443855	46443855	46444100	246	2	0.00050763	4	1.62601626	NA	NA
DMRcontig31391:46506301	46506301	46506449	149	1	0.000147748	1	0.67114094	NA	NA
DMRcontig31392:46507501	46507501	46507714	214	1	2.33E-05	9	4.205607477	NA	NA
DMRcontig31397:46514301	46514301	46514600	300	1	0.000154753	25	8.333333333	NA	NA
DMRcontig31429:46559538	46559538	46559708	171	1	0.000479653	3	1.754385965	NA	NA
DMRcontig31437:46570401	46570401	46570500	100	1	0.0007245	1	1	NA	NA
DMRcontig31449:46587101	46587101	46587400	300	1	8.01E-05	19	6.333333333	NA	NA
DMRcontig31465:46608801	46608801	46609100	300	1	1.88E-06	4	1.333333333	NA	NA
DMRcontig31471:46617301	46617301	46617600	300	1	0.000582399	6	2	NA	NA
DMRcontig31505:46664201	46664201	46664371	171	1	0.000128199	6	3.50877193	NA	NA
DMRcontig31577:46761801	46761801	46762100	300	2	5.08E-06	14	4.666666667	NA	NA
DMRcontig31594:46784533	46784533	46784700	168	2	3.96E-05	9	5.357142857	NA	NA
DMRcontig31621:46819349	46819349	46819561	213	1	0.000761122	1	0.469483568	NA	NA
DMRcontig31721:46945434	46945434	46945696	263	2	0.000397796	2	0.760456274	NA	NA
DMRcontig31761:46991901	46991901	46992100	200	1	0.000462876	7	3.5	rpl27a	translation
DMRcontig31764:46996101	46996101	46996400	300	1	0.000502275	12	4	NA	NA
DMRcontig31907:47182601	47182601	47182742	142	1	0.000943773	11	7.746478873	NA	NA
DMRcontig31909:47184901	47184901	47185018	118	1	0.000657448	9	7.627118644	NA	NA
DMRcontig31969:47259062	47259062	47259200	139	2	0.000619712	0	0	NA	NA
DMRcontig32031:47333448	47333448	47333934	487	1	0.000588472	10	2.05338809	NA	NA
DMRcontig32079:47390701	47390701	47390841	141	1	0.000945662	0	0	NA	NA
DMRcontig32107:47428001	47428001	47428100	100	1	0.000492614	1	1	NA	NA
DMRcontig32112:47434955	47434955	47435172	218	1	0.000310084	5	2.293577982	NA	NA
DMRcontig32116:47440719	47440719	47441451	733	2	0.0001325	52	7.094133697	NA	NA
DMRcontig32128:47458226	47458226	47458700	475	1	0.000795317	28	5.894736842	NA	NA
DMRcontig32130:47461181	47461181	47461961	781	1	0.000644826	63	8.066581306	NA	NA
DMRcontig32135:47468935	47468935	47469466	532	3	7.79E-05	31	5.827067669	NA	NA
DMRcontig32186:47531402	47531402	47531527	126	1	0.000196747	2	1.587301587	NA	NA
DMRcontig32256:47607001	47607001	47607123	123	1	0.000457265	0	0	NA	NA
DMRcontig32281:47633521	47633521	47633775	255	1	0.000819703	1	0.392156863	NA	NA
DMRcontig32306:47660801	47660801	47660939	139	1	0.000536144	1	0.71942446	NA	NA
DMRcontig32311:47666136	47666136	47666412	277	2	0.000866031	8	2.888086643	NA	NA

DMRcontig32349:47707701	47707701	47707842	142	2	0.000170761	3	2.112676056	NA	NA
DMRcontig32421:47786401	47786401	47786800	400	1	0.000670419	15	3.75	NA	NA
DMRcontig32538:47915431	47915431	47915586	156	2	0.000506957	0	0	NA	NA
DMRcontig32587:47969763	47969763	47969900	138	2	6.81E-05	6	4.347826087	NA	NA
DMRcontig32654:48044901	48044901	48045098	198	2	0.000119314	9	4.545454545	NA	NA
DMRcontig32670:48063516	48063516	48063829	314	1	0.000294468	16	5.095541401	NA	NA
DMRcontig32701:48098210	48098210	48098700	491	1	0.000528838	16	3.258655804	NA	NA
DMRcontig32740:48143818	48143818	48143871	54	1	0.000502027	0	0	NA	NA
DMRcontig32766:48172701	48172701	48172993	293	1	0.000337451	4	1.365187713	NA	NA
DMRcontig32776:48184048	48184048	48184136	89	2	0.000180228	0	0	NA	NA
DMRcontig32795:48205701	48205701	48205969	269	1	0.00040204	9	3.345724907	NA	NA
DMRcontig32810:48223037	48223037	48223100	64	1	4.10E-05	1	1.5625	NA	NA
DMRcontig32854:48272701	48272701	48272800	100	1	0.000328962	2	2	NA	NA
DMRcontig32864:48283613	48283613	48283700	88	1	0.000360771	1	1.136363636	NA	NA
DMRcontig32917:48343382	48343382	48343495	114	2	6.14E-05	2	1.754385965	NA	NA
DMRcontig32937:48365781	48365781	48365927	147	1	0.000879234	5	3.401360544	NA	NA
DMRcontig32958:48390101	48390101	48390204	104	1	0.000557612	2	1.923076923	NA	NA
DMRcontig32962:48394505	48394505	48394559	55	1	0.000291142	0	0	NA	NA
DMRcontig33018:48457537	48457537	48457900	364	1	0.000220304	22	6.043956044	NA	NA
DMRcontig33026:48467301	48467301	48467499	199	2	4.99E-05	10	5.025125628	NA	NA
DMRcontig33061:48507601	48507601	48507886	286	2	0.000295312	6	2.097902098	NA	NA
DMRcontig33068:48515901	48515901	48516058	158	1	0.000299018	1	0.632911392	NA	NA
DMRcontig33156:48616612	48616612	48616740	129	1	0.000967159	1	0.775193798	NA	NA
DMRcontig33157:48617741	48617741	48618106	366	1	5.50E-05	7	1.912568306	NA	NA
DMRcontig33170:48633111	48633111	48633400	290	2	4.50E-05	7	2.413793103	NA	NA
DMRcontig33203:48671801	48671801	48671938	138	2	0.000185551	5	3.623188406	NA	NA
DMRcontig33218:48688361	48688361	48688429	69	1	0.000831571	2	2.898550725	NA	NA
DMRcontig33225:48695809	48695809	48695917	109	2	0.000151785	3	2.752293578	NA	NA
DMRcontig33273:48750274	48750274	48750600	327	2	6.57E-05	8	2.44648318	NA	NA
DMRcontig33282:48760342	48760342	48760462	121	2	9.38E-05	1	0.826446281	NA	NA
DMRcontig33324:48809156	48809156	48809244	89	1	0.000959188	0	0	NA	NA
DMRcontig33355:48844309	48844309	48844354	46	1	0.000750003	1	2.173913043	NA	NA
DMRcontig33360:48850301	48850301	48850700	400	1	3.63E-05	9	2.25	MAE_RS27860	miscellaneous
DMRcontig33398:48896001	48896001	48896273	273	1	3.36E-05	10	3.663003663	NA	NA
DMRcontig33407:48906274	48906274	48906415	142	3	2.15E-05	2	1.408450704	NA	NA
DMRcontig33476:48986720	48986720	48986895	176	1	0.00088759	8	4.545454545	NA	NA
DMRcontig33500:49013469	49013469	49013520	52	1	0.00069938	0	0	NA	NA
DMRcontig33515:49030386	49030386	49030881	496	2	3.25E-07	28	5.64516129	NA	NA
DMRcontig33573:49095336	49095336	49095432	97	2	0.000193814	1	1.030927835	NA	NA
DMRcontig33618:49146771	49146771	49146824	54	1	0.000935239	0	0	NA	NA
DMRcontig33619:49147825	49147825	49147885	61	1	0.00019159	1	1.639344262	NA	NA
DMRcontig33623:49152506	49152506	49152604	99	1	2.49E-05	0	0	NA	NA
DMRcontig33694:49236801	49236801	49237000	200	1	0.000150064	6	3	NA	NA
DMRcontig33707:49251872	49251872	49251943	72	2	3.12E-05	0	0	NA	NA
DMRcontig33781:49335901	49335901	49336073	173	2	1.98E-05	1	0.578034682	NA	NA
DMRcontig33826:49389062	49389062	49389113	52	1	0.000451945	0	0	NA	NA
DMRcontig33839:49404501	49404501	49404644	144	2	0.000244747	3	2.083333333	NA	NA
DMRcontig33845:49411401	49411401	49411600	200	1	0.000603328	8	4	NA	NA
DMRcontig33855:49423001	49423001	49423197	197	2	3.49E-06	5	2.538071066	NA	NA
DMRcontig33879:49450941	49450941	49451146	206	2	0.000124934	2	0.970873786	NA	NA
DMRcontig33902:49477602	49477602	49477699	98	1	0.000603351	0	0	NA	NA
DMRcontig33904:49480201	49480201	49480300	100	1	0.000645762	4	4	NA	NA
DMRcontig33928:49507601	49507601	49507654	54	1	0.000336206	0	0	NA	NA
DMRcontig33931:49510826	49510826	49511100	275	1	0.000588294	13	4.727272727	NA	NA
DMRcontig33941:49522099	49522099	49522176	78	1	0.000779916	3	3.846153846	NA	NA
DMRcontig34014:49605401	49605401	49605600	200	1	0.000197784	4	2	NA	NA
DMRcontig34022:49614801	49614801	49614874	74	1	4.98E-05	7	9.459459459	NA	NA
DMRcontig34023:49615875	49615875	49616046	172	2	0.000428578	2	1.162790698	NA	NA
DMRcontig34049:49645617	49645617	49645699	83	1	0.000989886	1	1.204819277	NA	NA
DMRcontig34055:49652446	49652446	49652526	81	2	0.000456488	0	0	NA	NA
DMRcontig34074:49674170	49674170	49674400	231	1	4.46E-05	3	1.298701299	NA	NA
DMRcontig34126:49737101	49737101	49737469	369	1	1.74E-05	6	1.62601626	NA	NA
DMRcontig34136:49749223	49749223	49749400	178	1	0.000600754	0	0	NA	NA
DMRcontig34152:49768017	49768017	49768126	110	2	4.33E-05	9	8.181818182	NA	NA
DMRcontig34167:49785301	49785301	49785568	268	1	0.000606726	12	4.47761194	NA	NA
DMRcontig34185:49806101	49806101	49806371	271	1	0.000733581	3	1.10701107	NA	NA
DMRcontig34217:49844701	49844701	49844871	171	2	0.000253831	13	7.602339181	NA	NA
DMRcontig34230:49860201	49860201	49860261	61	1	0.00072713	2	3.278688525	NA	NA
DMRcontig34248:49880177	49880177	49880362	186	1	8.98E-05	2	1.075268817	NA	NA
DMRcontig34332:49976401	49976401	49976529	129	1	0.000508687	0	0	NA	NA
DMRcontig34413:50069109	50069109	50069246	138	1	0.000731478	7	5.072463768	NA	NA
DMRcontig34435:50094505	50094505	50094613	109	2	7.55E-05	3	2.752293578	NA	NA
DMRcontig34511:50181501	50181501	50181657	157	1	0.000876307	5	3.184713376	NA	NA
DMRcontig34516:50187501	50187501	50187773	273	1	6.67E-05	10	3.663003663	NA	NA
DMRcontig34547:50223254	50223254	50223320	67	2	4.83E-06	1	1.492537313	NA	NA
DMRcontig34553:50230417	50230417	50230470	54	1	0.000381765	2	3.703703704	NA	NA
DMRcontig34566:50245755	50245755	50245840	86	2	0.000515615	2	2.325581395	NA	NA
DMRcontig34608:50295201	50295201	50295500	300	2	8.21E-05	15	5	NA	NA
DMRcontig34673:50371745	50371745	50371892	148	1	0.000473695	2	1.351351351	NA	NA
DMRcontig34727:50433501	50433501	50433698	198	1	0.000325023	4	2.02020202	NA	NA

DMRcontig34745:50454401	50454401	50454503	103	1	0.000496175	2	1.941747573	NA	NA
DMRcontig34761:50472935	50472935	50473100	166	1	0.000166	2	1.204819277	NA	NA
DMRcontig34791:50506632	50506632	50506679	48	1	0.000986347	2	4.166666667	NA	NA
DMRcontig34815:50533606	50533606	50533648	43	1	0.000487398	7	16.27906977	NA	NA
DMRcontig34888:50615963	50615963	50616033	71	2	0.000206236	0	0	NA	NA
DMRcontig35027:50798101	50798101	50798224	124	1	0.000647939	1	0.806451613	NA	NA
DMRcontig35039:50817917	50817917	50818200	284	1	1.06E-05	13	4.577464789	NA	NA
DMRcontig35166:51016901	51016901	51017500	600	2	8.86E-06	29	4.833333333	NA	NA
DMRcontig35218:51103501	51103501	51103710	210	1	0.000149409	12	5.714285714	NA	NA
DMRcontig35252:51157601	51157601	51157900	300	2	1.07E-05	15	5	NA	NA
DMRcontig35253:51159027	51159027	51159200	174	1	0.000521849	4	2.298850575	NA	NA
DMRcontig35266:51179401	51179401	51179800	400	1	0.000739274	13	3.25	NA	NA
DMRcontig35288:51212448	51212448	51212700	253	2	2.68E-05	12	4.743083004	NA	NA
DMRcontig35334:51282701	51282701	51283100	400	1	0.000490345	19	4.75	NA	NA
DMRcontig35335:51284260	51284260	51285100	841	1	6.87E-06	16	1.902497027	NA	NA
DMRcontig35345:51300601	51300601	51301220	620	1	1.86E-05	20	3.225806452	NA	NA
DMRcontig35375:51349601	51349601	51349800	200	1	0.000259215	2	1	NA	NA
DMRcontig35381:51358401	51358401	51359000	600	1	0.000139218	47	7.833333333	NA	NA
DMRcontig35410:51403039	51403039	51403200	162	1	0.000198078	2	1.234567901	NA	NA
DMRcontig35440:51449547	51449547	51450000	454	1	0.000665996	24	5.286343612	NA	NA
DMRcontig35443:51454501	51454501	51454765	265	1	0.000444216	0	0	NA	NA
DMRcontig35446:51458648	51458648	51459000	353	1	0.000174771	11	3.116147309	NA	NA
DMRcontig35458:51476868	51476868	51477100	233	1	0.00082426	18	7.725321888	NA	NA
DMRcontig35473:51500068	51500068	51500400	333	2	0.000343701	8	2.402402402	NA	NA
DMRcontig35512:51558913	51558913	51559300	388	1	0.000267209	14	3.608247423	NA	NA
DMRcontig35519:51568907	51568907	51569300	394	1	1.16E-05	24	6.091370558	NA	NA
DMRcontig35526:51579612	51579612	51580000	389	2	0.000245227	7	1.799485861	NA	NA
DMRcontig35537:51596701	51596701	51597000	300	1	0.000339228	2	0.666666667	NA	NA
DMRcontig35580:51662901	51662901	51663200	300	2	4.62E-05	5	1.666666667	NA	NA
DMRcontig35592:51680735	51680735	51681100	366	1	0.000831902	10	2.732240437	NA	NA
DMRcontig35668:51798301	51798301	51798800	500	2	8.02E-05	34	6.8	NA	NA
DMRcontig35681:51818840	51818840	51819200	361	1	0.000140506	23	6.371191136	NA	NA
DMRcontig35746:51917801	51917801	51918492	692	5	2.77E-05	19	2.74566474	NA	NA
DMRcontig35779:51968101	51968101	51968294	194	1	0.000544498	10	5.154639175	LOC105169637;FARSA	unknown
DMRcontig35795:51991851	51991851	51992418	568	4	2.67E-06	25	4.401408451	NA	NA
DMRcontig35857:52084701	52084701	52085000	300	3	8.97E-06	21	7	NA	NA
DMRcontig35887:52128901	52128901	52129398	498	2	8.31E-05	18	3.614457831	NA	NA
DMRcontig35941:52210041	52210041	52210200	160	2	4.05E-12	12	7.5	NA	NA
DMRcontig35945:52215901	52215901	52216200	300	1	0.000265757	6	2	NA	NA
DMRcontig35970:52252301	52252301	52252400	100	1	0.000665261	3	3	NA	NA
DMRcontig35976:52261601	52261601	52261860	260	1	0.000618185	5	1.923076923	NA	NA
DMRcontig35995:52290301	52290301	52290700	400	1	1.01E-05	6	1.5	NA	NA
DMRcontig36008:52309657	52309657	52310000	344	1	0.000176808	20	5.813953488	NA	NA
DMRcontig36041:52360001	52360001	52360368	368	3	1.99E-05	15	4.076086957	NA	NA
DMRcontig36048:52370649	52370649	52370900	252	2	1.67E-05	10	3.968253968	NA	NA
DMRcontig36076:52412733	52412733	52413158	426	1	0.000440215	23	5.399061033	NA	NA
DMRcontig36119:52476101	52476101	52476375	275	1	0.000957916	11	4	NA	NA
DMRcontig36121:52479201	52479201	52479500	300	1	0.000950317	4	1.333333333	NA	NA
DMRcontig36144:52513574	52513574	52513900	327	1	0.000186094	13	3.975535168	NA	NA
DMRcontig36232:52644201	52644201	52644400	200	1	0.000404917	14	7	NA	NA
DMRcontig36246:52665025	52665025	52665588	564	2	1.63E-06	30	5.319148936	NA	NA
DMRcontig36279:52715701	52715701	52716000	300	1	0.000613224	14	4.666666667	XRN1	transcription
DMRcontig36358:52829914	52829914	52830100	187	2	7.38E-05	5	2.673796791	NA	NA
DMRcontig36371:52848995	52848995	52849448	454	5	8.95E-05	32	7.04845815	NA	NA
DMRcontig36402:52895301	52895301	52895700	400	1	0.00024536	7	1.75	NA	NA
DMRcontig36403:52897001	52897001	52897291	291	1	0.000911459	15	5.154639175	NA	NA
DMRcontig36557:53125085	53125085	53125545	461	1	0.00080088	26	5.639913232	NA	NA
DMRcontig36560:53129601	53129601	53129900	300	1	0.000966449	2	0.666666667	NA	NA
DMRcontig36581:53161201	53161201	53161400	200	1	2.46E-05	3	1.5	NA	NA
DMRcontig36583:53164501	53164501	53164700	200	2	2.18E-05	2	1	NA	NA
DMRcontig36592:53177698	53177698	53177900	203	1	0.000102742	2	0.985221675	NA	NA
DMRcontig36598:53186901	53186901	53187200	300	1	9.02E-05	14	4.666666667	NA	NA
DMRcontig36600:53189901	53189901	53190162	262	2	3.64E-07	8	3.053435115	NA	NA
DMRcontig36652:53268425	53268425	53268700	276	1	0.000246606	11	3.985507246	NA	NA
DMRcontig36664:53286720	53286720	53287000	281	1	0.000987511	13	4.62633452	NA	NA
DMRcontig36733:53391001	53391001	53391269	269	2	0.000233562	25	9.293680297	NA	NA
DMRcontig36904:53641901	53641901	53642396	496	4	0.00013252	12	2.419354839	NA	NA
DMRcontig36936:53688801	53688801	53689000	200	1	0.000413443	2	1	NA	NA
DMRcontig36967:53734401	53734401	53734558	158	2	7.59E-07	2	1.265822785	NA	NA
DMRcontig36969:53737301	53737301	53737562	262	1	0.000981487	4	1.526717557	NA	NA
DMRcontig36996:53777301	53777301	53777500	200	1	0.000307911	4	2	NA	NA
DMRcontig37000:53782791	53782791	53783100	310	3	0.000110134	17	5.483870968	NA	NA
DMRcontig37014:53804106	53804106	53804524	419	2	2.47E-06	9	2.14797136	NA	NA
DMRcontig37067:53881001	53881001	53881500	500	1	0.00043474	23	4.6	NA	NA
DMRcontig37076:53894239	53894239	53894600	362	1	0.000801292	13	3.591160221	NA	NA
DMRcontig37109:53942706	53942706	53942900	195	1	0.000562515	12	6.153846154	NA	NA
DMRcontig37174:54039801	54039801	54039900	100	1	9.55E-06	4	4	NA	NA
DMRcontig37176:54042601	54042601	54042900	300	1	0.000262758	13	4.333333333	NA	NA
DMRcontig37187:54059225	54059225	54059745	521	5	4.74E-07	20	3.838771593	NA	NA
DMRcontig37196:54072601	54072601	54073100	500	4	1.01E-05	16	3.2	NA	NA

DMRcontig37248:54150701	54150701	54150882	182	2	4.22E-05	4	2.197802198	NA	NA
DMRcontig37257:54163301	54163301	54163794	494	1	0.000191818	34	6.882591093	NA	NA
DMRcontig37266:54176901	54176901	54177196	296	1	0.000340165	8	2.702702703	NA	NA
DMRcontig37281:54199201	54199201	54199400	200	1	5.89E-05	6	3	NA	NA
DMRcontig37289:54210955	54210955	54211100	146	2	0.00050435	11	7.534246575	NA	NA
DMRcontig37291:54214210	54214210	54214300	91	1	0.000123308	2	2.197802198	CPEB2	transcription
DMRcontig37293:54217210	54217210	54217600	391	1	0.000492614	3	0.767263427	NA	NA
DMRcontig37308:54239501	54239501	54239955	455	3	2.37E-05	16	3.516483516	NA	NA
DMRcontig37315:54249901	54249901	54250200	300	1	0.000189565	16	5.333333333	NA	NA
DMRcontig37338:54283501	54283501	54283900	400	2	2.24E-05	13	3.25	NA	NA
DMRcontig37366:54324901	54324901	54325082	182	1	0.000771444	4	2.197802198	NA	NA
DMRcontig37438:54427720	54427720	54428256	537	1	7.70E-05	34	6.331471136	KIF1A	cytoskeleton
DMRcontig37447:54440601	54440601	54441100	500	1	0.000579757	13	2.6	NA	NA
DMRcontig37467:54469643	54469643	54470000	358	1	0.000379813	10	2.793296089	NA	NA
DMRcontig37472:54477601	54477601	54477800	200	1	0.000560463	3	1.5	NA	NA
DMRcontig37481:54490744	54490744	54491193	450	3	1.67E-08	14	3.111111111	NA	NA
DMRcontig37487:54500301	54500301	54500600	300	1	0.000690126	13	4.333333333	NA	NA
DMRcontig37513:54539656	54539656	54540132	477	2	0.000284864	12	2.51572327	NA	NA
DMRcontig37575:54630344	54630344	54630600	257	1	9.09E-05	15	5.836575875	NA	NA
DMRcontig37596:54661101	54661101	54661800	700	1	0.000182225	38	5.428571429	LOC103188862	metabolism and transport
DMRcontig37608:54678701	54678701	54678900	200	1	7.45E-05	3	1.5	NA	NA
DMRcontig37640:54725030	54725030	54725200	171	2	5.09E-05	14	8.187134503	NA	NA
DMRcontig37641:54726401	54726401	54726800	400	1	4.01E-06	12	3	NA	NA
DMRcontig37643:54729901	54729901	54730080	180	2	4.36E-06	1	0.555555556	NA	NA
DMRcontig37657:54749901	54749901	54750200	300	2	0.000429949	6	2	LOC107190651	metabolism and transport
DMRcontig37665:54761101	54761101	54761597	497	4	1.03E-05	29	5.83501006	NA	NA
DMRcontig37671:54769901	54769901	54770100	200	1	0.00098	8	4	NA	NA
DMRcontig37676:54777401	54777401	54777700	300	2	3.45E-05	15	5	IscW_ISCW018041;aars	metabolism
DMRcontig37708:54824501	54824501	54824739	239	1	0.00040061	4	1.673640167	NA	NA
DMRcontig37763:54905201	54905201	54905400	200	1	4.53E-05	11	5.5	NA	NA
DMRcontig37786:54939701	54939701	54939900	200	1	0.000312811	9	4.5	NA	NA
DMRcontig37794:54951243	54951243	54951700	458	1	0.000113365	30	6.550218341	NA	NA
DMRcontig37801:54961701	54961701	54962200	500	1	0.000472329	15	3	NA	NA
DMRcontig37806:54968969	54968969	54969600	632	1	5.33E-05	34	5.379746835	NA	NA
DMRcontig37812:54978535	54978535	54978868	334	2	0.000298064	13	3.892215569	NA	NA
DMRcontig37827:55000384	55000384	55000600	217	1	4.80E-05	11	5.069124424	NA	NA
DMRcontig37829:55003129	55003129	55003900	772	1	8.17E-05	48	6.21761658	NA	NA
DMRcontig37846:55029105	55029105	55029408	304	3	0.000225087	10	3.289473684	NA	NA
DMRcontig37847:55030601	55030601	55030845	245	1	1.20E-05	10	4.081632653	NA	NA
DMRcontig37867:55059356	55059356	55060200	845	1	0.000448294	13	1.538461538	NA	NA
DMRcontig37939:55165927	55165927	55166100	174	1	0.000445367	2	1.149425287	NA	NA
DMRcontig37979:55225301	55225301	55225599	299	2	1.93E-05	3	1.003344482	NA	NA
DMRcontig37992:55244101	55244101	55244373	273	2	0.000853763	18	6.593406593	NA	NA
DMRcontig38054:55334680	55334680	55334900	221	1	5.86E-05	18	8.14479638	NA	NA
DMRcontig38094:55393537	55393537	55393996	460	2	0.000127831	16	3.47826087	NA	NA
DMRcontig38098:55399601	55399601	55400010	410	1	0.000366433	29	7.073170732	NA	NA
DMRcontig38162:55494177	55494177	55494636	460	1	0.000601719	27	5.869565217	NA	NA
DMRcontig38196:55542901	55542901	55543100	200	1	0.000186085	3	1.5	NA	NA
DMRcontig38213:55567807	55567807	55568288	482	1	0.000800524	17	3.526970954	TRIATDRAFT_160795	unknown
DMRcontig38225:55586501	55586501	55586644	144	2	0.000187506	3	2.083333333	NA	NA
DMRcontig38250:55622901	55622901	55623331	431	3	0.000182467	26	6.032482599	NA	NA
DMRcontig38252:55625742	55625742	55626160	419	1	0.000188078	23	5.489260143	LOC107005161;LOC107004584; LOC102598453	unknown
DMRcontig38255:55630301	55630301	55630500	200	2	0.000482868	4	2	NA	NA
DMRcontig38267:55647901	55647901	55648070	170	1	0.00098786	0	0	NA	NA
DMRcontig38268:55649301	55649301	55649491	191	1	0.000852384	4	2.094240838	NA	NA
DMRcontig38280:55666305	55666305	55666778	474	1	0.00063692	14	2.953586498	NA	NA
DMRcontig38295:55688301	55688301	55688500	200	1	0.000125315	13	6.5	NA	NA
DMRcontig38379:55810622	55810622	55811092	471	2	3.71E-06	31	6.581740977	NA	NA
DMRcontig38408:55852928	55852928	55853300	373	1	0.000468502	15	4.021447721	NA	NA
DMRcontig38434:55891008	55891008	55891400	393	1	0.000368173	15	3.816793893	LOC107029121	unknown
DMRcontig38462:55931701	55931701	55931878	178	1	0.000882706	3	1.685393258	NA	NA
DMRcontig38464:55934321	55934321	55934600	280	1	0.000304716	3	1.071428571	NA	NA
DMRcontig38482:55960405	55960405	55960596	192	1	0.000558308	2	1.041666667	NA	NA
DMRcontig38497:55981512	55981512	55982223	712	1	6.68E-05	17	2.387640449	NA	NA
DMRcontig38517:56011022	56011022	56011200	179	1	0.000728424	3	1.675977654	NA	NA
DMRcontig38542:56047341	56047341	56047800	460	2	0.000310815	18	3.913043478	NA	NA
DMRcontig38556:56068101	56068101	56068300	200	1	0.000616282	8	4	NA	NA
DMRcontig38559:56072134	56072134	56072547	414	2	0.000464614	7	1.690821256	NA	NA
DMRcontig38560:56073901	56073901	56074003	103	1	0.000807141	6	5.825242718	NA	NA
DMRcontig38564:56079271	56079271	56079700	430	2	5.14E-07	17	3.953488372	NA	NA
DMRcontig38579:56100901	56100901	56101400	500	1	0.000887811	18	3.6	NA	NA
DMRcontig38590:56116601	56116601	56116800	200	1	0.000299638	4	2	NA	NA
DMRcontig38612:56148301	56148301	56148500	200	1	9.96E-05	4	2	NA	NA
DMRcontig38618:56156747	56156747	56157184	438	1	0.000308109	7	1.598173516	NA	NA
DMRcontig38627:56169801	56169801	56169986	186	1	0.000207327	4	2.150537634	NA	NA
DMRcontig38656:56211226	56211226	56211500	275	1	6.43E-05	12	4.363636364	NA	NA
DMRcontig38682:56248932	56248932	56249500	569	1	0.000692439	31	5.448154657	NA	NA
DMRcontig38751:56349101	56349101	56349540	440	2	0.000331298	19	4.318181818	NA	NA
DMRcontig38781:56392256	56392256	56392900	645	2	5.20E-06	24	3.720930233	NA	NA

DMRcontig38784:56397101	56397101	56397300	200	1	0.00044933	10	5	NA	NA
DMRcontig38814:56440442	56440442	56440800	359	2	0.000457217	31	8.635097493	NA	NA
DMRcontig38818:56446201	56446201	56446600	400	2	3.84E-05	7	1.75	NA	NA
DMRcontig38823:56453601	56453601	56453900	300	1	0.000328537	6	2	NA	NA
DMRcontig38834:56469430	56469430	56469848	419	1	8.40E-06	14	3.341288783	NA	NA
DMRcontig38838:56475501	56475501	56475600	100	1	0.000198085	4	4	LOC102800180	signaling
DMRcontig38842:56481201	56481201	56481397	197	1	0.000981487	5	2.538071066	NA	NA
DMRcontig38856:56501501	56501501	56501700	200	1	0.000524949	7	3.5	NA	NA
DMRcontig38896:56559701	56559701	56559800	100	1	0.000390738	2	2	NA	NA
DMRcontig38904:56570801	56570801	56571200	400	1	0.000533639	16	4	NA	NA
DMRcontig38987:56692201	56692201	56692437	237	1	0.000669582	6	2.53164557	NA	NA
DMRcontig39012:56728092	56728092	56728300	209	1	0.000135732	6	2.870813397	NA	NA
DMRcontig39048:56780101	56780101	56780469	369	1	0.000362548	5	1.35501355	NA	NA
DMRcontig39091:56842056	56842056	56842473	418	1	0.000362874	11	2.631578947	NA	NA
DMRcontig39100:56855204	56855204	56855894	691	2	0.000109173	16	2.315484805	NA	NA
DMRcontig39113:56874030	56874030	56874499	470	2	6.15E-05	23	4.893617021	NA	NA
DMRcontig39130:56898501	56898501	56898700	200	1	0.000676062	13	6.5	NA	NA
DMRcontig39159:56940626	56940626	56941040	415	3	0.000123354	11	2.65060241	NA	NA
DMRcontig39167:56951966	56951966	56952341	376	2	5.13E-07	10	2.659574468	NA	NA
DMRcontig39189:56983601	56983601	56983744	144	1	0.000703276	2	1.388888889	NA	NA
DMRcontig39202:57001518	57001518	57001960	443	1	0.000470679	3	0.677200903	NA	NA
DMRcontig39222:57030253	57030253	57030400	148	1	0.000995331	9	6.081081081	NA	NA
DMRcontig39244:57061601	57061601	57061900	300	1	0.000466249	14	4.666666667	NA	NA
DMRcontig39260:57084510	57084510	57085000	491	1	4.35E-05	16	3.258655804	NA	NA
DMRcontig39263:57089001	57089001	57089300	300	1	0.000380745	7	2.333333333	NA	NA
DMRcontig39273:57103301	57103301	57103463	163	1	0.00098	4	2.45398773	NA	NA
DMRcontig39295:57134292	57134292	57134400	109	1	0.00024536	1	0.917431193	NA	NA
DMRcontig39308:57153043	57153043	57153400	358	1	0.00098241	14	3.910614525	NA	NA
DMRcontig39313:57160301	57160301	57160592	292	1	8.46E-05	17	5.821917808	NA	NA
DMRcontig39319:57168601	57168601	57168800	200	1	0.000390626	10	5	NA	NA
DMRcontig39336:57192901	57192901	57193369	469	3	0.00024444	24	5.117270789	NA	NA
DMRcontig39352:57216034	57216034	57216200	167	1	9.45E-05	3	1.796407186	NA	NA
DMRcontig39383:57260001	57260001	57260300	300	1	0.000224433	12	4	NA	NA
DMRcontig39388:57267004	57267004	57267398	395	2	0.000165858	9	2.278481013	NA	NA
DMRcontig39397:57280478	57280478	57280897	420	3	0.000115474	24	5.714285714	NA	NA
DMRcontig39421:57315301	57315301	57315588	288	1	0.000122318	3	1.041666667	NA	NA
DMRcontig39457:57368150	57368150	57368500	351	3	4.97E-05	14	3.988603989	NA	NA
DMRcontig39489:57414301	57414301	57414500	200	1	0.000286244	15	7.5	NA	NA
DMRcontig39515:57450934	57450934	57451100	167	1	6.34E-05	6	3.592814371	NA	NA
DMRcontig39524:57464301	57464301	57464666	366	1	0.000586496	3	0.819672131	NA	NA
DMRcontig39528:57469801	57469801	57470100	300	1	0.000116138	16	5.333333333	NA	NA
DMRcontig39532:57475349	57475349	57475800	452	1	0.000384228	33	7.300884956	NA	NA
DMRcontig39551:57503116	57503116	57503500	385	2	9.57E-05	14	3.636363636	NA	NA
DMRcontig39583:57549501	57549501	57549754	254	1	0.000192253	6	2.362204724	NA	NA
DMRcontig39594:57565200	57565200	57565400	201	3	2.52E-06	3	1.492537313	NA	NA
DMRcontig39604:57579628	57579628	57580100	473	3	3.50E-05	25	5.285412262	NA	NA
DMRcontig39643:57635451	57635451	57635700	250	1	0.000933521	14	5.6	NA	NA
DMRcontig39710:57730901	57730901	57731100	200	1	0.000403273	3	1.5	NA	NA
DMRcontig39733:57764501	57764501	57764774	274	1	0.000937334	5	1.824817518	TDRD15;APOB	transcription
DMRcontig39750:57788701	57788701	57788800	100	1	0.000867024	3	3	NA	NA
DMRcontig39798:57856375	57856375	57856500	126	1	0.000270586	1	0.793650794	NA	NA
DMRcontig39819:57886926	57886926	57887396	471	3	0.00028518	17	3.609341826	NA	NA
DMRcontig39858:57942501	57942501	57942800	300	1	0.000656861	14	4.666666667	LOC106933862;kank1	metabolism
DMRcontig39886:57982501	57982501	57982900	400	1	4.01E-05	23	5.75	NA	NA
DMRcontig39892:57990969	57990969	57991400	432	1	0.000176806	18	4.166666667	NA	NA
DMRcontig39909:58015601	58015601	58015858	258	1	0.000425505	8	3.100775194	NA	NA
DMRcontig40005:58154414	58154414	58154800	387	2	0.000328011	1	0.258397933	NA	NA
DMRcontig40007:58157301	58157301	58157500	200	1	0.000726011	11	5.5	NA	NA
DMRcontig40014:58166674	58166674	58167400	727	1	7.98E-05	14	1.925722146	NA	NA
DMRcontig40039:58203401	58203401	58203700	300	1	0.000683956	6	2	NA	NA
DMRcontig40075:58253901	58253901	58254100	200	2	9.01E-05	3	1.5	NA	NA
DMRcontig40096:58284601	58284601	58284765	165	2	6.04E-05	4	2.424242424	NA	NA
DMRcontig40255:58510379	58510379	58510600	222	1	0.000712259	13	5.855855856	NA	NA
DMRcontig40257:58513301	58513301	58513400	100	1	0.000659331	2	2	NA	NA
DMRcontig40277:58542001	58542001	58542354	354	1	0.000756939	14	3.95480226	NA	NA
DMRcontig40283:58551001	58551001	58551300	300	1	0.000760231	14	4.666666667	NA	NA
DMRcontig40296:58569601	58569601	58569700	100	1	0.000483338	3	3	NA	NA
DMRcontig40299:58574101	58574101	58574238	138	1	0.000763362	1	0.724637681	NA	NA
DMRcontig40301:58576701	58576701	58576982	282	1	9.09E-05	12	4.255319149	LOC101849745;ATG3	protease
DMRcontig40313:58593901	58593901	58594100	200	1	0.000377794	10	5	NA	NA
DMRcontig40315:58596601	58596601	58596999	399	1	0.000896068	11	2.756892231	NA	NA
DMRcontig40317:58599601	58599601	58599700	100	1	0.000154155	5	5	NA	NA
DMRcontig40338:58629089	58629089	58629300	212	2	0.000240549	12	5.660377358	NA	NA
DMRcontig40349:58644619	58644619	58644900	282	1	2.59E-05	13	4.609929078	NA	NA
DMRcontig40378:58686201	58686201	58686482	282	2	3.42E-06	6	2.127659574	NA	NA
DMRcontig40390:58702963	58702963	58703100	138	1	0.000655069	1	0.724637681	NA	NA
DMRcontig40404:58723040	58723040	58723568	529	1	0.000647557	25	4.725897921	NA	NA
DMRcontig40412:58734601	58734601	58734966	366	1	0.000370312	20	5.464480874	NA	NA
DMRcontig40429:58758801	58758801	58759100	300	1	0.000619663	16	5.333333333	NA	NA
DMRcontig40436:58768701	58768701	58769084	384	3	7.01E-07	12	3.125	NA	NA

DMRcontig40498:58856001	58856001	58856346	346	1	0.000632455	14	4.046242775	NA	NA
DMRcontig40504:58864501	58864501	58864692	192	1	0.000342807	10	5.208333333	NA	NA
DMRcontig40513:58876829	58876829	58877000	172	1	5.60E-05	7	4.069767442	NA	NA
DMRcontig40588:58982859	58982859	58983000	142	1	1.18E-06	8	5.633802817	NA	NA
DMRcontig40625:59035222	59035222	59035400	179	1	0.000638607	1	0.558659218	NA	NA
DMRcontig40637:59051720	59051720	59052112	393	1	0.000773489	12	3.053435115	NA	NA
DMRcontig40641:59057001	59057001	59057200	200	1	3.85E-07	3	1.5	NA	NA
DMRcontig40704:59145201	59145201	59145637	437	1	0.00045124	18	4.118993135	LOC101392574;EHD3	metabolism
DMRcontig40749:59209342	59209342	59209500	159	1	0.00094984	8	5.031446541	NA	NA
DMRcontig40780:59251905	59251905	59252374	470	1	0.000981487	7	1.489361702	NA	NA
DMRcontig40783:59256301	59256301	59256500	200	1	0.000122291	7	3.5	NA	NA
DMRcontig40808:59291760	59291760	59292200	441	3	3.66E-06	23	5.215419501	NA	NA
DMRcontig40809:59293310	59293310	59293679	370	1	0.000430448	13	3.513513514	NA	NA
DMRcontig40819:59306901	59306901	59307100	200	1	0.000279712	2	1	NA	NA
DMRcontig40827:59317816	59317816	59318000	185	2	5.53E-05	9	4.864864865	NA	NA
DMRcontig40853:59354701	59354701	59354900	200	2	1.84E-06	6	3	NA	NA
DMRcontig40858:59361644	59361644	59361990	347	1	0.000158361	23	6.628242075	NA	NA
DMRcontig40864:59370501	59370501	59370600	100	1	0.000553343	4	4	NA	NA
DMRcontig40903:59425719	59425719	59425900	182	2	0.000335384	13	7.142857143	NA	NA
DMRcontig40971:59521353	59521353	59521500	148	1	0.000946379	2	1.351351351	NA	NA
DMRcontig40984:59539783	59539783	59540300	518	2	5.20E-05	12	2.316602317	NA	NA
DMRcontig41114:59722946	59722946	59723200	255	1	0.00098621	18	7.058823529	NA	NA
DMRcontig41152:59776001	59776001	59776256	256	1	7.22E-06	7	2.734375	NA	NA
DMRcontig41168:59798833	59798833	59799100	268	1	0.00033868	18	6.71641791	NA	NA
DMRcontig41194:59835201	59835201	59835300	100	1	0.000224596	4	4	NA	NA
DMRcontig41211:59859562	59859562	59859800	239	2	8.17E-06	6	2.510460251	NA	NA
DMRcontig41301:59985504	59985504	59985967	464	2	7.18E-05	26	5.603448276	NA	NA
DMRcontig41329:60024501	60024501	60024884	384	1	0.000273103	6	1.5625	NA	NA
DMRcontig41345:60047401	60047401	60047700	300	1	0.000315626	6	2	NA	NA
DMRcontig41364:60074457	60074457	60074700	244	2	7.31E-05	12	4.918032787	NA	NA
DMRcontig41393:60115501	60115501	60115853	353	1	0.000418976	8	2.266288952	NA	NA
DMRcontig41419:60152101	60152101	60152390	290	1	0.000751509	12	4.137931034	NA	NA
DMRcontig41451:60197186	60197186	60197400	215	1	0.000400876	2	0.930232558	NA	NA
DMRcontig41495:60258701	60258701	60258900	200	1	0.000941398	10	5	NA	NA
DMRcontig41498:60262601	60262601	60262772	172	2	4.61E-05	1	0.581395349	NA	NA
DMRcontig41519:60291501	60291501	60291732	232	1	0.000762226	1	0.431034483	NA	NA
DMRcontig41526:60301001	60301001	60301361	361	1	3.94E-06	4	1.108033241	NA	NA
DMRcontig41585:60381701	60381701	60381900	200	1	0.000733581	4	2	NA	NA
DMRcontig41597:60398401	60398401	60398600	200	2	0.000423565	10	5	NA	NA
DMRcontig41731:60584201	60584201	60584500	300	1	0.000511007	8	2.666666667	NA	NA
DMRcontig41768:60635101	60635101	60635486	386	1	0.0002517	28	7.25388601	bcl11a	transcription
DMRcontig41857:60760101	60760101	60760600	500	4	8.21E-06	14	2.8	NA	NA
DMRcontig41883:60796201	60796201	60796660	460	3	0.000166046	12	2.608695652	NA	NA
DMRcontig41902:60822401	60822401	60822700	300	2	7.64E-08	22	7.333333333	NA	NA
DMRcontig41960:60903401	60903401	60903599	199	1	0.000240339	1	0.502512563	NA	NA
DMRcontig42028:60999601	60999601	60999701	101	2	7.70E-05	0	0	NA	NA
DMRcontig42088:61082646	61082646	61083000	355	2	0.000109201	22	6.197183099	NA	NA
DMRcontig42111:61114962	61114962	61115200	239	1	0.000553618	3	1.255230126	naxd;inpp4b	metabolism
DMRcontig42122:61130301	61130301	61130600	300	2	5.00E-05	9	3	NA	NA
DMRcontig42125:61134453	61134453	61134900	448	2	0.000172388	17	3.794642857	NA	NA
DMRcontig42129:61140674	61140674	61140900	227	1	0.000363584	2	0.881057269	NA	NA
DMRcontig42132:61145001	61145001	61145300	300	1	0.00024536	2	0.666666667	NA	NA
DMRcontig42133:61146410	61146410	61146616	207	1	4.94E-05	3	1.449275362	NA	NA
DMRcontig42158:61180822	61180822	61181167	346	3	9.02E-06	14	4.046242775	NA	NA
DMRcontig42197:61233968	61233968	61234243	276	1	0.000576283	5	1.811594203	NA	NA
DMRcontig42372:61478001	61478001	61478113	113	1	6.87E-05	2	1.769911504	NA	NA
DMRcontig42384:61494301	61494301	61494330	30	1	0.00098	0	0	NA	NA
DMRcontig42385:61495331	61495331	61495471	141	1	0.00043882	3	2.127659574	NA	NA
DMRcontig42389:61500603	61500603	61500800	198	1	0.000291991	18	9.090909091	NA	NA
DMRcontig42522:61686801	61686801	61687185	385	2	2.53E-06	19	4.935064935	IscW_ISCW023383;sox2	development
DMRcontig42558:61736101	61736101	61736500	400	1	0.000166097	17	4.25	NA	NA
DMRcontig42576:61761101	61761101	61761292	192	1	0.000733581	1	0.520833333	NA	NA
DMRcontig42601:61795401	61795401	61795600	200	1	0.000276368	7	3.5	NA	NA
DMRcontig42670:61889347	61889347	61889600	254	1	0.000360293	3	1.181102362	NA	NA
DMRcontig42685:61910701	61910701	61910900	200	1	3.78E-06	2	1	NA	NA
DMRcontig42717:61954901	61954901	61955200	300	1	2.55E-05	22	7.333333333	NA	NA
DMRcontig42752:62003201	62003201	62003800	600	1	2.95E-05	20	3.333333333	NA	NA
DMRcontig42773:62032605	62032605	62032700	96	1	0.000595343	1	1.041666667	NA	NA
DMRcontig42783:62046201	62046201	62046552	352	2	0.000139322	15	4.261363636	NA	NA
DMRcontig42831:62112166	62112166	62112401	236	2	0.000161648	7	2.966101695	NA	NA
DMRcontig42833:62114772	62114772	62115000	229	1	0.000745848	15	6.550218341	NA	NA
DMRcontig42841:62125125	62125125	62125530	406	4	1.59E-06	11	2.709359606	itpr1b	signaling
DMRcontig42848:62135101	62135101	62135400	300	1	0.000177375	10	3.333333333	NA	NA
DMRcontig42924:62240701	62240701	62241000	300	1	0.000792521	5	1.666666667	NA	NA
DMRcontig43007:62355901	62355901	62356000	100	1	0.000733581	3	3	NA	NA
DMRcontig43044:62405807	62405807	62406014	208	2	0.000397322	6	2.884615385	LOC100615600;LOC107985520	unknown
DMRcontig43062:62429901	62429901	62430169	269	2	8.17E-06	3	1.115241636	NA	NA
DMRcontig43064:62432701	62432701	62432900	200	2	8.44E-05	7	3.5	NA	NA
DMRcontig43079:62453101	62453101	62453200	100	1	3.84E-06	7	7	NA	NA
DMRcontig43080:62454484	62454484	62454708	225	2	0.000203013	10	4.444444444	NA	NA

DMRcontig43092:62471118	62471118	62471400	283	1	0.000267803	20	7.067137809	NA	NA
DMRcontig43094:62474001	62474001	62474300	300	1	0.000204614	9	3	NA	NA
DMRcontig43095:62475401	62475401	62475800	400	1	0.000188532	12	3	NA	NA
DMRcontig43097:62478137	62478137	62478514	378	1	0.000484827	12	3.174603175	NA	NA
DMRcontig43100:62482335	62482335	62482755	421	2	0.000118666	19	4.513064133	NA	NA
DMRcontig43119:62508643	62508643	62508800	158	2	0.000393545	14	8.860759494	NA	NA
DMRcontig43130:62523653	62523653	62523800	148	2	0.000172498	14	9.459459459	NA	NA
DMRcontig43139:62535101	62535101	62535473	373	2	0.000362658	9	2.412868633	NA	NA
DMRcontig43151:62551701	62551701	62552100	400	1	1.24E-05	18	4.5	NA	NA
DMRcontig43162:62566901	62566901	62567100	200	1	0.000210704	2	1	NA	NA
DMRcontig43182:62594101	62594101	62594988	888	2	9.53E-08	51	5.743243243	NA	NA
DMRcontig43224:62652407	62652407	62652900	494	1	7.86E-05	18	3.643724696	NA	NA
DMRcontig43249:62686601	62686601	62686893	293	1	0.000715069	23	7.849829352	NA	NA
DMRcontig43329:62795801	62795801	62796149	349	1	0.000109484	9	2.578796562	NA	NA
DMRcontig43414:62912964	62912964	62913100	137	1	0.000259988	8	5.839416058	RPL7A	translation
DMRcontig43428:62932201	62932201	62932470	270	1	3.45E-05	7	2.592592593	NA	NA
DMRcontig43454:62968260	62968260	62968500	241	1	0.000106414	8	3.319502075	NA	NA
DMRcontig43522:63061201	63061201	63061385	185	1	0.000490345	15	8.108108108	NA	NA
DMRcontig43528:63068659	63068659	63068800	142	2	0.000476502	1	0.704225352	NA	NA
DMRcontig43545:63091573	63091573	63092095	523	4	1.02E-05	22	4.206500956	NA	NA
DMRcontig43621:63195207	63195207	63195700	494	2	0.000251291	17	3.441295547	NA	NA
DMRcontig43636:63215270	63215270	63215600	331	1	0.000130196	16	4.833836858	NA	NA
DMRcontig43659:63247101	63247101	63247200	100	1	0.000362747	3	3	NA	NA
DMRcontig43681:63277737	63277737	63278000	264	1	0.000691695	8	3.03030303	NA	NA
DMRcontig43729:63345401	63345401	63345673	273	1	0.000105137	6	2.197802198	NA	NA
DMRcontig43787:63423914	63423914	63424100	187	1	0.000973723	7	3.743315508	NA	NA
DMRcontig43806:63449301	63449301	63449561	261	1	0.000538866	5	1.915708812	NA	NA
DMRcontig43916:63599266	63599266	63599400	135	2	1.04E-05	5	3.703703704	NA	NA
DMRcontig43917:63600715	63600715	63600900	186	1	1.12E-05	11	5.913978495	NA	NA
DMRcontig43942:63633834	63633834	63634000	167	1	0.000862153	6	3.592814371	NA	NA
DMRcontig43956:63652701	63652701	63652800	100	1	0.000931529	7	7	NA	NA
DMRcontig43961:63659301	63659301	63659498	198	1	0.000955079	11	5.555555556	NA	NA
DMRcontig43979:63684201	63684201	63684400	200	2	1.43E-05	5	2.5	NA	NA
DMRcontig44004:63719101	63719101	63719254	154	2	0.000621393	3	1.948051948	NA	NA
DMRcontig44009:63725601	63725601	63725900	300	1	0.00078564	13	4.333333333	NA	NA
DMRcontig44061:63796501	63796501	63796729	229	1	0.000101959	8	3.493449782	NA	NA
DMRcontig44108:63860701	63860701	63861100	400	2	6.42E-05	25	6.25	NA	NA
DMRcontig44112:63866644	63866644	63866939	296	2	0.000196669	10	3.378378378	NA	NA
DMRcontig44121:63878161	63878161	63878400	240	1	0.000733581	5	2.083333333	NA	NA
DMRcontig44174:63949901	63949901	63950025	125	1	0.000280334	9	7.2	LOC101860977	unknown
DMRcontig44178:63955701	63955701	63955800	100	1	6.07E-05	6	6	NA	NA
DMRcontig44215:64007401	64007401	64007488	88	1	0.000238459	1	1.136363636	NA	NA
DMRcontig44315:64146150	64146150	64146300	151	1	0.000490345	8	5.298013245	NA	NA
DMRcontig44330:64166821	64166821	64167100	280	2	4.82E-05	11	3.928571429	NA	NA
DMRcontig44341:64181601	64181601	64181800	200	1	0.000921417	8	4	NA	NA
DMRcontig44368:64216901	64216901	64217133	233	1	0.000132434	2	0.858369099	NA	NA
DMRcontig44374:64225601	64225601	64225737	137	1	0.000620269	2	1.459854015	RPL23AP84;Gm32856;RNU6-744P;D616_p158002	translation and protein modification
DMRcontig44382:64236101	64236101	64236300	200	1	0.00036634	8	4	NA	NA
DMRcontig44418:64285382	64285382	64285580	199	2	4.19E-05	3	1.507537688	NA	NA
DMRcontig44539:64447787	64447787	64448200	414	4	2.86E-05	12	2.898550725	NA	NA
DMRcontig44586:64510601	64510601	64510984	384	3	4.14E-06	13	3.385416667	NA	NA
DMRcontig44640:64583301	64583301	64583417	117	1	3.93E-05	4	3.418803419	NA	NA
DMRcontig44649:64595315	64595315	64595583	269	1	0.00081384	15	5.576208178	NA	NA
DMRcontig44660:64610101	64610101	64610300	200	1	0.000425397	7	3.5	NA	NA
DMRcontig44672:64626370	64626370	64626600	231	1	0.000405587	3	1.298701299	NA	NA
DMRcontig44744:64726001	64726001	64726109	109	1	0.000294888	0	0	NA	NA
DMRcontig44752:64736132	64736132	64736300	169	2	0.000725215	12	7.100591716	NA	NA
DMRcontig44778:64771701	64771701	64771900	200	1	0.000490715	0	0	NA	NA
DMRcontig44794:64793201	64793201	64793300	100	1	0.00015575	1	1	NA	NA
DMRcontig44801:64802612	64802612	64802979	368	2	4.69E-05	8	2.173913043	NA	NA
DMRcontig44811:64816501	64816501	64816643	143	1	0.00074251	4	2.797202797	NA	NA
DMRcontig44819:64827701	64827701	64827900	200	1	0.00024701	12	6	NA	NA
DMRcontig44836:64850101	64850101	64850400	300	1	0.000887361	6	2	NA	NA
DMRcontig44849:64868318	64868318	64868400	83	1	0.000538866	7	8.43373494	NA	NA
DMRcontig44869:64894101	64894101	64894400	300	1	0.000390514	8	2.666666667	NA	NA
DMRcontig44988:65053701	65053701	65053981	281	2	0.000107396	11	3.914590747	NA	NA
DMRcontig44990:65056401	65056401	65056596	196	1	0.000376974	5	2.551020408	NA	NA
DMRcontig45032:65112415	65112415	65112800	386	1	0.000962842	7	1.813471503	NA	NA
DMRcontig45053:65141129	65141129	65141615	487	4	7.53E-05	19	3.901437372	NA	NA
DMRcontig45060:65150701	65150701	65150881	181	2	2.04E-05	2	1.104972376	NA	NA
DMRcontig45134:65250050	65250050	65250417	368	1	0.000260927	16	4.347826087	NA	NA
DMRcontig45169:65298718	65298718	65299100	383	2	8.39E-05	10	2.610966057	NA	NA
DMRcontig45187:65324301	65324301	65324500	200	1	0.000135723	5	2.5	NA	NA
DMRcontig45239:65395301	65395301	65395500	200	1	0.000960041	1	0.5	NA	NA
DMRcontig45302:65478904	65478904	65479053	150	1	0.000621783	0	0	NA	NA
DMRcontig45318:65499459	65499459	65499600	142	1	0.00016547	1	0.704225352	NA	NA
DMRcontig45327:65511051	65511051	65511300	250	1	0.000502602	7	2.8	NA	NA
DMRcontig45337:65524409	65524409	65524700	292	1	0.00077791	10	3.424657534	NA	NA
DMRcontig45341:65529801	65529801	65529979	179	1	0.000468874	4	2.234636872	NA	NA

DMRcontig45377:65577135	65577135	65577300	166	2	0.000273256	6	3.614457831	NA	NA
DMRcontig45423:65641301	65641301	65641700	400	1	0.000940368	8	2	NA	NA
DMRcontig45427:65646853	65646853	65647541	689	1	7.57E-05	20	2.90275762	NA	NA
DMRcontig45453:65682529	65682529	65682703	175	2	8.09E-06	10	5.714285714	NA	NA
DMRcontig45489:65730101	65730101	65730400	300	1	0.000414693	4	1.333333333	NA	NA
DMRcontig45542:65801701	65801701	65801829	129	2	0.000189161	1	0.775193798	NA	NA
DMRcontig45587:65861901	65861901	65862037	137	1	0.000636807	11	8.02919708	NA	NA
DMRcontig45598:65875701	65875701	65876000	300	2	0.000138336	3	1	NA	NA
DMRcontig45600:65878338	65878338	65878509	172	2	4.56E-06	8	4.651162791	NA	NA
DMRcontig45615:65898001	65898001	65898256	256	2	0.000146081	2	0.78125	NA	NA
DMRcontig45679:65983614	65983614	65984189	576	1	0.000138857	24	4.166666667	NA	NA
DMRcontig45684:65990501	65990501	65990669	169	2	0.000447451	4	2.366863905	NA	NA
DMRcontig45692:66000601	66000601	66000800	200	1	3.34E-05	3	1.5	NA	NA
DMRcontig45702:66014179	66014179	66014300	122	2	0.000465767	3	2.459016393	NA	NA
DMRcontig45731:66053301	66053301	66053509	209	1	0.000648031	8	3.827751196	NA	NA
DMRcontig45758:66088301	66088301	66088700	400	1	0.000106416	19	4.75	NA	NA
DMRcontig45835:66190501	66190501	66191298	798	3	0.000104049	20	2.506265664	NA	NA
DMRcontig45862:66226665	66226665	66226900	236	1	0.000363802	3	1.271186441	NA	NA
DMRcontig45874:66243401	66243401	66243555	155	1	0.000340824	11	7.096774194	NA	NA
DMRcontig45910:66291805	66291805	66291970	166	2	3.84E-05	3	1.807228916	NA	NA
DMRcontig45927:66314945	66314945	66315100	156	1	0.000122686	2	1.282051282	NA	NA
DMRcontig46026:66446901	66446901	66447300	400	1	0.000873036	17	4.25	NA	NA
DMRcontig46033:66457701	66457701	66457788	88	1	0.000726069	2	2.272727273	NA	NA
DMRcontig46036:66461401	66461401	66461700	300	1	7.14E-05	8	2.666666667	NA	NA
DMRcontig46088:66530301	66530301	66530600	300	1	0.000541745	6	2	NA	NA
DMRcontig46097:66543401	66543401	66543600	200	1	0.000547533	2	1	NA	NA
DMRcontig46126:66583531	66583531	66583700	170	1	0.00016248	9	5.294117647	NA	NA
DMRcontig46141:66603001	66603001	66603200	200	1	3.86E-05	3	1.5	NA	NA
DMRcontig46179:66653601	66653601	66654000	400	1	0.000955121	11	2.75	NA	NA
DMRcontig46233:66725901	66725901	66726300	400	1	0.000852864	15	3.75	NA	NA
DMRcontig46250:66748252	66748252	66748500	249	1	0.000147835	3	1.204819277	NA	NA
DMRcontig46318:66841210	66841210	66841559	350	1	0.000335443	13	3.714285714	NA	NA
DMRcontig46341:66871701	66871701	66872000	300	2	9.42E-06	28	9.333333333	NA	NA
DMRcontig46350:66884001	66884001	66884151	151	1	0.000650113	2	1.324503311	NA	NA
DMRcontig46352:66886240	66886240	66886400	161	1	0.000356989	6	3.726708075	NA	NA
DMRcontig46367:66905901	66905901	66906100	200	1	7.70E-05	10	5	NA	NA
DMRcontig46400:66949733	66949733	66950200	468	2	0.000227862	12	2.564102564	NA	NA
DMRcontig46417:66971088	66971088	66971200	113	1	0.000538866	4	3.539823009	NA	NA
DMRcontig46473:67046401	67046401	67046713	313	1	0.00072715	11	3.514376997	NA	NA
DMRcontig46497:67078004	67078004	67078150	147	1	0.000158101	6	4.081632653	NA	NA
DMRcontig46522:67110081	67110081	67110435	355	1	0.000989444	5	1.408450704	NA	NA
DMRcontig46532:67122901	67122901	67123100	200	1	0.000490345	0	0	NA	NA
DMRcontig46538:67130742	67130742	67131200	459	1	0.000787389	29	6.318082789	NA	NA
DMRcontig46564:67165380	67165380	67165900	521	2	8.21E-05	25	4.798464491	NA	NA
DMRcontig46613:67231655	67231655	67231736	82	1	0.00098	1	1.219512195	NA	NA
DMRcontig46622:67243101	67243101	67243632	532	1	0.000153916	16	3.007518797	NA	NA
DMRcontig46641:67268201	67268201	67268623	423	1	0.000315797	11	2.600472813	NA	NA
DMRcontig46644:67272285	67272285	67272500	216	2	0.000362321	5	2.314814815	NA	NA
DMRcontig46685:67324401	67324401	67324666	266	1	9.72E-05	5	1.879699248	NA	NA
DMRcontig46699:67342297	67342297	67342462	166	1	0.00091598	3	1.807228916	NA	NA
DMRcontig46781:67451301	67451301	67451600	300	1	0.000698033	3	1	NA	NA
DMRcontig46839:67528349	67528349	67528467	119	1	0.000718165	4	3.361344538	NA	NA
DMRcontig46852:67544409	67544409	67544527	119	1	0.000677403	5	4.201680672	NA	NA
DMRcontig46968:67699001	67699001	67699400	400	2	2.60E-05	8	2	NA	NA
DMRcontig46980:67714355	67714355	67714586	232	1	0.000719607	7	3.017241379	NA	NA
DMRcontig47019:67765001	67765001	67765387	387	1	0.000196521	11	2.842377261	NA	NA
DMRcontig47032:67781866	67781866	67782086	221	1	0.000251992	2	0.904977376	NA	NA
DMRcontig47036:67786875	67786875	67787200	326	1	0.000761912	23	7.055214724	NA	NA
DMRcontig47044:67798401	67798401	67798500	100	1	0.000611802	6	6	NA	NA
DMRcontig47142:67924008	67924008	67924300	293	1	0.000307311	9	3.071672355	NA	NA
DMRcontig47147:67930519	67930519	67930666	148	1	0.000384062	2	1.351351351	NA	NA
DMRcontig47173:67965447	67965447	67965700	254	1	0.000631146	6	2.362204724	NA	NA
DMRcontig47197:67998158	67998158	67998480	323	1	0.000405504	4	1.238390093	NA	NA
DMRcontig47204:68007314	68007314	68007722	409	2	0.000195922	23	5.623471883	NA	NA
DMRcontig47217:68024563	68024563	68024800	238	1	2.72E-05	14	5.882352941	NA	NA
DMRcontig47231:68043801	68043801	68043900	100	1	0.000117103	3	3	NA	NA
DMRcontig47265:68089301	68089301	68089700	400	2	0.000652601	11	2.75	NA	NA
DMRcontig47271:68097758	68097758	68097900	143	1	9.29E-05	3	2.097902098	NA	NA
DMRcontig47290:68122774	68122774	68122900	127	2	2.02E-05	0	0	NA	NA
DMRcontig47295:68129054	68129054	68129200	147	2	1.53E-05	3	2.040816327	NA	NA
DMRcontig47329:68173945	68173945	68174127	183	1	0.00069692	3	1.639344262	NA	NA
DMRcontig47357:68211505	68211505	68211700	196	1	0.000525442	6	3.06122449	NA	NA
DMRcontig47379:68240664	68240664	68240790	127	1	0.000374657	0	0	NA	NA
DMRcontig47429:68304301	68304301	68304510	210	2	8.90E-07	8	3.80952381	NA	NA
DMRcontig47442:68321701	68321701	68321955	255	1	0.000743096	3	1.176470588	NA	NA
DMRcontig47448:68328931	68328931	68329100	170	1	0.000284771	2	1.176470588	NA	NA
DMRcontig47459:68343279	68343279	68343491	213	2	1.84E-06	6	2.816901408	NA	NA
DMRcontig47482:68374338	68374338	68374500	163	1	0.000796584	1	0.613496933	NA	NA
DMRcontig47490:68384201	68384201	68384353	153	1	6.14E-05	5	3.267973856	NA	NA
DMRcontig47578:68501701	68501701	68502100	400	2	0.000409813	10	2.5	NA	NA

DMRcontig47590:68518206	68518206	68518400	195	1	0.000918643	3	1.538461538	NA	NA
DMRcontig47593:68521992	68521992	68522100	109	1	0.000440641	3	2.752293578	NA	NA
DMRcontig47614:68549766	68549766	68549900	135	1	0.000460801	3	2.222222222	NA	NA
DMRcontig47633:68574501	68574501	68574571	71	1	0.00045817	2	2.816901408	NA	NA
DMRcontig47640:68583618	68583618	68583900	283	1	0.000821203	8	2.826855124	NA	NA
DMRcontig47651:68598801	68598801	68599000	200	2	0.00028891	8	4	NA	NA
DMRcontig47736:68709501	68709501	68709700	200	2	0.000521849	4	2	NA	NA
DMRcontig47741:68716301	68716301	68716707	407	1	0.000925328	22	5.405405405	NA	NA
DMRcontig47772:68754194	68754194	68754300	107	1	0.000169318	3	2.803738318	NA	NA
DMRcontig47820:68817411	68817411	68817680	270	2	4.17E-07	7	2.592592593	NA	NA
DMRcontig47829:68829601	68829601	68830200	600	4	1.29E-05	5	0.833333333	NA	NA
DMRcontig47859:68870001	68870001	68870123	123	2	2.70E-05	1	0.81300813	NA	NA
DMRcontig47892:68913701	68913701	68913900	200	1	0.000160809	5	2.5	NA	NA
DMRcontig47902:68925983	68925983	68926100	118	1	0.000555581	3	2.542372881	NA	NA
DMRcontig47907:68932401	68932401	68932800	400	1	6.41E-05	15	3.75	NA	NA
DMRcontig47929:68961215	68961215	68961500	286	2	1.38E-06	33	11.53846154	SORBIDRAFT_02g000380;tnc	miscellaneous
DMRcontig47977:69023609	69023609	69023800	192	1	0.000163792	0	0	NA	NA
DMRcontig47993:69045201	69045201	69045384	184	1	0.000599716	9	4.891304348	NA	NA
DMRcontig47999:69052267	69052267	69052575	309	1	0.000157889	8	2.588996764	NA	NA
DMRcontig48081:69159401	69159401	69159541	141	1	0.000631014	2	1.418439716	NA	NA
DMRcontig48125:69216359	69216359	69216478	120	1	0.000838614	5	4.166666667	NA	NA
DMRcontig48224:69343416	69343416	69343700	285	2	0.00017182	5	1.754385965	NA	NA
DMRcontig48250:69377801	69377801	69378262	462	1	0.000137931	14	3.03030303	NA	NA
DMRcontig48261:69392501	69392501	69392700	200	1	0.000592932	6	3	NA	NA
DMRcontig48273:69408301	69408301	69408500	200	1	0.000569242	8	4	NA	NA
DMRcontig48307:69451376	69451376	69451647	272	1	7.55E-06	5	1.838235294	NA	NA
DMRcontig48321:69471068	69471068	69471176	109	1	0.00062286	1	0.917431193	NA	NA
DMRcontig48325:69475801	69475801	69476100	300	1	0.000854023	19	6.333333333	NA	NA
DMRcontig48365:69527401	69527401	69527744	344	2	6.89E-06	9	2.61627907	NA	NA
DMRcontig48430:69613453	69613453	69613700	248	3	3.64E-05	9	3.629032258	NA	NA
DMRcontig48473:69668401	69668401	69668655	255	2	0.000135837	12	4.705882353	NA	NA
DMRcontig48653:69900678	69900678	69900893	216	3	1.39E-05	2	0.925925926	NA	NA
DMRcontig48674:69929001	69929001	69929294	294	2	4.70E-05	9	3.06122449	NA	NA
DMRcontig48691:69951801	69951801	69951961	161	1	5.45E-05	6	3.726708075	NA	NA
DMRcontig48715:69983515	69983515	69983813	299	1	1.91E-05	11	3.678929766	NA	NA
DMRcontig48742:70018001	70018001	70018338	338	1	2.69E-05	9	2.662721893	NA	NA
DMRcontig48755:70034401	70034401	70034546	146	1	0.000986777	5	3.424657534	NA	NA
DMRcontig48757:70036946	70036946	70037162	217	2	1.56E-05	9	4.147465438	NA	NA
DMRcontig48788:70076601	70076601	70076700	100	1	0.000199292	5	5	NA	NA
DMRcontig48789:70077801	70077801	70078100	300	2	3.93E-05	11	3.666666667	NA	NA
DMRcontig48805:70099801	70099801	70100098	298	1	0.0006955	11	3.691275168	NA	NA
DMRcontig48811:70107561	70107561	70107943	383	1	0.000218761	6	1.566579634	NA	NA
DMRcontig48813:70110301	70110301	70110447	147	2	6.68E-05	14	9.523809524	NA	NA
DMRcontig48842:70147954	70147954	70148400	447	2	5.83E-05	9	2.013422819	NA	NA
DMRcontig48895:70213701	70213701	70214000	300	2	0.000324796	14	4.666666667	NA	NA
DMRcontig48926:70253376	70253376	70253500	125	1	0.000471453	0	0	NA	NA
DMRcontig48985:70328701	70328701	70329100	400	2	6.68E-05	13	3.25	NA	NA
DMRcontig48997:70344001	70344001	70344244	244	1	0.000577203	11	4.508196721	NA	NA
DMRcontig49011:70360669	70360669	70361172	504	1	1.45E-07	9	1.785714286	NA	NA
DMRcontig49020:70371462	70371462	70371693	232	1	0.000331451	11	4.74137931	LDAH	miscellaneous
DMRcontig49055:70415401	70415401	70416182	782	1	0.000492949	45	5.754475703	NA	NA
DMRcontig49161:70551149	70551149	70551400	252	1	0.000203661	12	4.761904762	NA	NA
DMRcontig49162:70552555	70552555	70552727	173	2	5.06E-05	4	2.312138728	NA	NA
DMRcontig49176:70569501	70569501	70569693	193	1	0.000600588	7	3.626943005	NA	NA
DMRcontig49300:70728552	70728552	70729000	449	1	0.000121711	13	2.89532294	NA	NA
DMRcontig49338:70777514	70777514	70777663	150	1	0.000659779	3	2	NA	NA
DMRcontig49342:70782501	70782501	70782800	300	2	0.000461663	7	2.333333333	NA	NA
DMRcontig49357:70801701	70801701	70801888	188	1	0.000177817	4	2.127659574	NA	NA
DMRcontig49379:70829001	70829001	70829700	700	1	0.00075885	14	2	NA	NA
DMRcontig49399:70853101	70853101	70853400	300	2	5.69E-05	17	5.666666667	NA	NA
DMRcontig49422:70882801	70882801	70883400	600	1	0.00098	30	5	NA	NA
DMRcontig49459:70930601	70930601	70930800	200	1	4.87E-05	8	4	NA	NA
DMRcontig49494:70976984	70976984	70977200	217	1	1.58E-05	10	4.608294931	HPCA	signaling
DMRcontig49501:70986651	70986651	70986710	60	1	0.000483057	0	0	NA	NA
DMRcontig49553:71051350	71051350	71051700	351	2	0.000205825	8	2.279202279	NA	NA
DMRcontig49569:71071701	71071701	71071810	110	1	0.00045449	4	3.636363636	NA	NA
DMRcontig49893:71473501	71473501	71473700	200	1	0.000290156	8	4	NA	NA
DMRcontig49944:71537301	71537301	71537600	300	1	5.67E-06	15	5	NA	NA
DMRcontig50017:71629643	71629643	71629966	324	1	0.00023252	22	6.790123457	NA	NA
DMRcontig50038:71654101	71654101	71654400	300	2	0.00015467	9	3	NA	NA
DMRcontig50069:71691101	71691101	71691700	600	1	0.000996843	19	3.166666667	NA	NA
DMRcontig50099:71729701	71729701	71730175	475	1	3.01E-05	11	2.315789474	NA	NA
DMRcontig50123:71759401	71759401	71759500	100	1	0.000627384	3	3	NA	NA
DMRcontig50144:71785278	71785278	71785364	87	2	0.000114783	1	1.149425287	NA	NA
DMRcontig50156:71798701	71798701	71799074	374	1	0.000554556	17	4.545454545	NA	NA
DMRcontig50268:71939101	71939101	71939800	700	1	0.000941974	23	3.285714286	NA	NA
DMRcontig50274:71946622	71946622	71947000	379	2	0.000126686	6	1.583113456	NA	NA
DMRcontig50303:71981701	71981701	71982700	1000	5	8.99E-05	65	6.5	NA	NA
DMRcontig50305:71985129	71985129	71985677	549	5	0.000116115	34	6.193078324	NA	NA
DMRcontig50307:71988122	71988122	71988600	479	2	0.000234181	38	7.933194154	NA	NA

DMRcontig50312:71995701	71995701	71996000	300	2	5.40E-05	16	5.333333333	NA	NA
DMRcontig50324:72012401	72012401	72012500	100	1	6.68E-05	1	1	EMIHUDRAFT_254823	cytoskeleton
DMRcontig50428:72143264	72143264	72143600	337	2	0.000884452	27	8.011869436	NA	NA
DMRcontig50462:72187516	72187516	72187678	163	2	3.12E-05	4	2.45398773	NA	NA
DMRcontig50466:72192801	72192801	72193030	230	1	0.00077004	5	2.173913043	NA	NA
DMRcontig50509:72249165	72249165	72249700	536	2	0.000852042	22	4.104477612	NA	NA
DMRcontig50569:72325794	72325794	72326062	269	2	2.97E-06	6	2.230483271	NA	NA
DMRcontig50606:72375001	72375001	72375100	100	1	0.00048771	3	3	NA	NA
DMRcontig50665:72447601	72447601	72448100	500	1	0.000199031	24	4.8	NA	NA
DMRcontig50685:72474539	72474539	72474735	197	1	0.00045836	8	4.060913706	NA	NA
DMRcontig50688:72478560	72478560	72478657	98	2	0.000617327	4	4.081632653	NA	NA
DMRcontig50708:72503501	72503501	72503587	87	1	0.000532252	4	4.597701149	NA	NA
DMRcontig50719:72517218	72517218	72517600	383	2	9.53E-05	23	6.005221932	NA	NA
DMRcontig50723:72522414	72522414	72522569	156	1	0.000478513	4	2.564102564	NA	NA
DMRcontig50727:72527417	72527417	72527600	184	1	0.000703937	1	0.543478261	NA	NA
DMRcontig50762:72575801	72575801	72575995	195	1	0.000548572	3	1.538461538	NA	NA
DMRcontig50831:72666474	72666474	72666564	91	2	2.92E-05	2	2.197802198	NA	NA
DMRcontig50834:72669689	72669689	72669900	212	1	0.000178859	12	5.660377358	NA	NA
DMRcontig50863:72707577	72707577	72708000	424	1	0.000448109	17	4.009433962	NA	NA
DMRcontig50869:72715432	72715432	72715557	126	1	0.000551692	5	3.968253968	NA	NA
DMRcontig50922:72783901	72783901	72784023	123	2	0.000283107	2	1.62601626	NA	NA
DMRcontig50942:72809289	72809289	72809400	112	1	0.000620737	5	4.464285714	NA	NA
DMRcontig50999:72879001	72879001	72879336	336	2	0.000530753	14	4.166666667	IK	growth factor
DMRcontig51015:72899801	72899801	72899982	182	1	0.000405587	2	1.098901099	NA	NA
DMRcontig51029:72916430	72916430	72916600	171	1	0.000134203	4	2.339181287	NA	NA
DMRcontig51112:73024580	73024580	73024700	121	1	0.000332427	7	5.785123967	NA	NA
DMRcontig51121:73036001	73036001	73036641	641	1	0.000224254	25	3.900156006	NA	NA
DMRcontig51130:73048301	73048301	73048451	151	1	0.000738635	3	1.986754967	NA	NA
DMRcontig51151:73075864	73075864	73076100	237	1	0.000554031	7	2.953586498	NA	NA
DMRcontig51199:73134855	73134855	73135054	200	3	1.03E-05	13	6.5	NA	NA
DMRcontig51228:73169201	73169201	73169436	236	1	1.45E-05	12	5.084745763	NA	NA
DMRcontig51258:73206617	73206617	73207000	384	3	1.38E-06	13	3.385416667	NA	NA
DMRcontig51282:73236086	73236086	73236311	226	3	3.75E-42	6	2.654867257	NA	NA
DMRcontig51292:73249201	73249201	73249700	500	4	2.51E-08	28	5.6	NA	NA
DMRcontig51296:73254701	73254701	73255122	422	1	0.000955195	20	4.739336493	NA	NA
DMRcontig51315:73279001	73279001	73279185	185	1	0.000453624	2	1.081081081	NA	NA
DMRcontig51351:73324201	73324201	73324400	200	2	0.000548072	1	0.5	NA	NA
DMRcontig51376:73354501	73354501	73354783	283	1	0.000253009	7	2.473498233	NA	NA
DMRcontig51378:73357301	73357301	73357400	100	1	0.000217781	3	3	NA	NA
DMRcontig51413:73400960	73400960	73401200	241	2	0.000132134	12	4.979253112	NA	NA
DMRcontig51439:73435429	73435429	73435500	72	1	0.000689953	2	2.777777778	NA	NA
DMRcontig51459:73463480	73463480	73463550	71	1	0.000450145	4	5.633802817	NA	NA
DMRcontig51468:73474901	73474901	73475000	100	1	0.000114255	2	2	NA	NA
DMRcontig51482:73492567	73492567	73492800	234	1	0.000551915	7	2.991452991	NA	NA
DMRcontig51483:73494001	73494001	73494300	300	1	0.000159054	5	1.666666667	NA	NA
DMRcontig51489:73502102	73502102	73502675	574	1	0.00098	3	0.522648084	NA	NA
DMRcontig51500:73517019	73517019	73517150	132	1	4.60E-05	2	1.515151515	NA	NA
DMRcontig51522:73545001	73545001	73545300	300	2	7.02E-06	15	5	NA	NA
DMRcontig51544:73573501	73573501	73573700	200	1	0.000413708	3	1.5	NA	NA
DMRcontig51566:73603657	73603657	73603900	244	1	4.76E-06	6	2.459016393	NA	NA
DMRcontig51573:73612707	73612707	73613089	383	1	0.000779663	7	1.82767624	NA	NA
DMRcontig51583:73625001	73625001	73625200	200	2	0.000258901	7	3.5	NA	NA
DMRcontig51622:73674601	73674601	73674700	100	1	0.00024536	0	0	NA	NA
DMRcontig51628:73681601	73681601	73682000	400	2	0.000232054	16	4	NA	NA
DMRcontig51684:73754901	73754901	73755249	349	1	0.000364014	16	4.584527221	NA	NA
DMRcontig51734:73817001	73817001	73817300	300	2	7.19E-07	7	2.333333333	NA	NA
DMRcontig51739:73823101	73823101	73823583	483	1	0.000796027	9	1.863354037	NA	NA
DMRcontig51823:73928801	73928801	73929000	200	1	0.000109601	11	5.5	NUO5	metabolism and transport
DMRcontig51827:73934801	73934801	73934872	72	1	0.000699073	1	1.388888889	NA	NA

Supplemental Table S2
Lake 2 vs. River 1

DMR & Contig	Start	Stop	Length (bp)	# SigWin	minP-value	CpG #	CpG Density	Gene Annotation	Gene Category
DMRcontig00010:25402	25402	25800	399	1	0.000393292	19	4.761904762	LOC101206387;ZMAT2	Transcription
DMRcontig00012:29574	29574	30100	527	1	0.000863528	11	2.087286528	COX1	Metabolism
DMRcontig00015:37301	37301	37700	400	1	5.77E-06	27	6.75	NA	NA
DMRcontig00016:39401	39401	39900	500	3	6.47E-07	28	5.6	NA	NA
DMRcontig00031:72901	72901	73500	600	1	0.00010687	34	5.666666667	NA	NA
DMRcontig00039:91501	91501	92400	900	2	0.000450777	35	3.888888889	eif2s3	Translation
DMRcontig00041:97101	97101	97900	800	1	0.000300344	39	4.875	NA	NA
DMRcontig00044:104201	104201	104768	568	1	0.00097054	27	4.753521127	NA	NA
DMRcontig00059:143401	143401	143530	130	1	0.000903974	10	7.692307692	NA	NA
DMRcontig00069:166401	166401	167700	1300	1	0.000674292	73	5.615384615	ddx39ab;Ddx39b	Unknown
DMRcontig00073:176201	176201	176700	500	2	1.49E-05	10	2	NA	NA
DMRcontig00074:178201	178201	178600	400	1	0.000543556	15	3.75	NA	NA
DMRcontig00083:198701	198701	199540	840	7	2.97E-06	45	5.357142857	PIGU	Signaling
DMRcontig00087:206746	206746	208195	1450	3	1.94E-05	74	5.103448276	LOC107219277	Cytoskeleton
DMRcontig00100:235701	235701	236600	900	1	0.000534818	31	3.444444444	NA	NA
DMRcontig00102:240201	240201	240800	600	1	0.000603044	43	7.166666667	LOC106179196;LOC106152685	Signaling
DMRcontig00103:242101	242101	243200	1100	1	2.40E-05	44	4	LOC103362722;atp5a1	Metabolism;Transport
DMRcontig00105:246801	246801	247200	400	2	4.84E-05	14	3.5	NA	NA
DMRcontig00111:260801	260801	261200	400	1	3.37E-05	14	3.5	NA	NA
DMRcontig00112:262701	262701	263300	600	2	0.000148032	17	2.833333333	NA	NA
DMRcontig00121:283901	283901	284200	300	1	0.000867311	12	4	Dere\GG15409	Unknown
DMRcontig00123:288101	288101	288400	300	2	4.12E-05	28	9.333333333	NA	NA
DMRcontig00124:290001	290001	290200	200	2	1.96E-05	7	3.5	NA	NA
DMRcontig00153:355601	355601	356600	1000	2	7.17E-07	45	4.5	NA	NA
DMRcontig00155:360401	360401	360800	400	1	5.29E-05	4	1	NA	NA
DMRcontig00170:396001	396001	396284	284	2	7.81E-05	10	3.521126761	NA	NA
DMRcontig00181:420001	420001	421327	1327	1	8.80E-06	57	4.295403165	LOC101878657;COL4A3BP	Binding Protein
DMRcontig00200:461344	461344	462200	857	1	0.000420875	16	1.86697783	opn1sw;arf5	Signaling;Receptor
DMRcontig00238:544801	544801	546600	1800	1	0.000131248	43	2.388888889	BRAFLDRAFT_260655;PHB	Unknown
DMRcontig00256:585501	585501	586000	500	4	4.47E-05	39	7.8	NA	NA
DMRcontig00265:604101	604101	605100	1000	1	0.000140294	54	5.4	NA	NA
DMRcontig00272:619570	619570	619900	331	2	4.73E-08	21	6.344410876	LOC106602804;LOC106609995; rps6	Translation
DMRcontig00278:633001	633001	633300	300	1	0.00051191	7	2.333333333	NA	NA
DMRcontig00284:645901	645901	646000	100	1	0.000516769	4	4	NA	NA
DMRcontig00285:648501	648501	649100	600	1	0.000415126	8	1.333333333	COX1	Metabolism
DMRcontig00305:692901	692901	693100	200	1	0.000621357	3	1.5	NA	NA
DMRcontig00307:696701	696701	698000	1300	2	0.000243969	41	3.153846154	NA	NA
DMRcontig00309:701601	701601	702300	700	3	3.63E-07	36	5.142857143	NA	NA
DMRcontig00355:802816	802816	804300	1485	3	0.00018967	90	6.060606061	NA	NA
DMRcontig00370:835501	835501	835877	377	1	2.68E-05	9	2.387267905	NA	NA
DMRcontig00373:841601	841601	842300	700	5	5.58E-07	39	5.571428571	LOC101061694;TUBB4B	Cytoskeleton
DMRcontig00375:845701	845701	846557	857	6	1.09E-05	55	6.417736289	NA	NA
DMRcontig00379:854401	854401	855400	1000	1	2.46E-05	47	4.7	NA	NA
DMRcontig00382:860531	860531	861500	970	3	2.79E-05	49	5.051546392	NA	NA
DMRcontig00392:882101	882101	882300	200	1	0.000120195	8	4	LOC101853885	Translation
DMRcontig00396:891201	891201	891600	400	1	0.000414386	10	2.5	NA	NA
DMRcontig00410:922001	922001	922896	896	2	8.36E-06	42	4.6875	CAOG_01866;Pepck	Signaling;Metabolism
DMRcontig00415:932101	932101	933200	1100	1	0.000124628	41	3.727272727	NA	NA
DMRcontig00416:934601	934601	935384	784	1	1.42E-06	23	2.933673469	NA	NA
DMRcontig00427:959301	959301	959800	500	1	4.44E-06	32	6.4	Dere\GG14870;RpL8	Unknown;Transcription
DMRcontig00428:960901	960901	961600	700	3	3.01E-05	16	2.285714286	SPRG_12594	Cytoskeleton
DMRcontig00437:979901	979901	980161	261	2	5.14E-05	10	3.831417625	NA	NA
DMRcontig00447:1001001	1001001	1002100	1100	1	0.000539776	32	2.909090909	NA	NA
DMRcontig00449:1005401	1005401	1005700	300	3	9.66E-10	29	9.666666667	NA	NA
DMRcontig00459:1026969	1026969	1027600	632	1	0.000108094	8	1.265822785	trnaw-cca	Translation
DMRcontig00468:1046201	1046201	1046600	400	2	7.60E-06	7	1.75	NA	NA
DMRcontig00470:1050501	1050501	1051500	1000	1	0.000451034	34	3.4	LOC106483173;psmc5	Protease
DMRcontig00477:1066801	1066801	1067100	300	1	0.00032125	9	3	NA	NA
DMRcontig00482:1076301	1076301	1076800	500	1	4.15E-05	21	4.2	HELRODRAFT_172351	Unknown
DMRcontig00486:1083801	1083801	1084393	593	1	4.10E-06	35	5.902192243	NCLIV_015160;RPL9	Unknown;Translation
DMRcontig00487:1085801	1085801	1086100	300	1	4.85E-05	12	4	NA	NA
DMRcontig00492:1096901	1096901	1098000	1100	1	0.000956873	27	2.454545455	NA	NA
DMRcontig00495:1104301	1104301	1105500	1200	1	0.000920989	41	3.416666667	NA	NA
DMRcontig00498:1111401	1111401	1111900	500	2	3.99E-05	26	5.2	NA	NA
DMRcontig00508:1132701	1132701	1133700	1000	5	2.97E-06	46	4.6	DDOST	Metabolism
DMRcontig00512:1141501	1141501	1141800	300	1	9.36E-05	23	7.666666667	EIF4E2	Translation
DMRcontig00513:1143301	1143301	1143600	300	1	0.000895801	4	1.333333333	NA	NA
DMRcontig00530:1177801	1177801	1178000	200	1	0.000157298	16	8	NA	NA
DMRcontig00538:1193001	1193001	1193800	800	2	0.000123647	30	3.75	NA	NA
DMRcontig00540:1197501	1197501	1198000	500	1	0.000100026	21	4.2	LOC101853112	Golgi
DMRcontig00548:1213265	1213265	1213900	636	5	3.48E-05	18	2.830188679	NA	NA

DMRcontig00556:1229901	1229901	1230200	300	1	0.000577812	11	3.666666667	NA	NA
DMRcontig00564:1247601	1247601	1247800	200	1	0.000857075	12	6	NA	NA
DMRcontig00586:1294601	1294601	1295167	567	2	4.90E-05	21	3.703703704	NA	NA
DMRcontig00589:1300901	1300901	1301700	800	1	0.000771824	18	2.25	NA	NA
DMRcontig00603:1329601	1329601	1330438	838	1	0.000691724	29	3.460620525	NA	NA
DMRcontig00608:1341201	1341201	1341531	331	2	9.54E-06	16	4.833836858	NA	NA
DMRcontig00611:1346101	1346101	1347000	900	1	0.00059935	11	1.222222222	NA	NA
DMRcontig00619:1363401	1363401	1363800	400	2	0.000195753	25	6.25	LOC101848543	Unknown
DMRcontig00631:1390001	1390001	1390592	592	1	7.49E-05	27	4.560810811	NA	NA
DMRcontig00637:1402101	1402101	1402700	600	2	1.33E-08	20	3.333333333	NA	NA
DMRcontig00648:1423748	1423748	1423900	153	1	0.000932159	13	8.496732026	NA	NA
DMRcontig00653:1434001	1434001	1434298	298	2	0.000296002	7	2.348993289	NA	NA
DMRcontig00656:1440101	1440101	1440400	300	1	0.000659712	9	3	NA	NA
DMRcontig00665:1457201	1457201	1458000	800	1	0.000967347	20	2.5	NA	NA
DMRcontig00666:1459201	1459201	1459500	300	2	9.35E-08	20	6.666666667	LOC104826917	Metabolism;Epigenetics
DMRcontig00680:1487701	1487701	1488003	303	1	3.64E-06	18	5.940594059	Dvir\GJ22302	Unknown
DMRcontig00681:1489301	1489301	1490200	900	1	0.000666067	43	4.777777778	NA	NA
DMRcontig00705:1539001	1539001	1539863	863	2	2.63E-05	46	5.330243337	NA	NA
DMRcontig00711:1553001	1553001	1553972	972	3	1.69E-05	29	2.983539095	LOC101857978	Binding Protein
DMRcontig00726:1585001	1585001	1585300	300	2	5.63E-05	7	2.333333333	NA	NA
DMRcontig00729:1591101	1591101	1591300	200	1	0.00072575	14	7	NA	NA
DMRcontig00731:1595001	1595001	1596100	1100	1	3.87E-05	38	3.454545455	NA	NA
DMRcontig00734:1601866	1601866	1602900	1035	1	2.85E-06	57	5.507246377	LOC101846557;vha-13	Transport
DMRcontig00743:1620401	1620401	1621100	700	2	1.43E-05	28	4	NA	NA
DMRcontig00757:1650301	1650301	1650700	400	1	0.000451034	12	3	NA	NA
DMRcontig00758:1652001	1652001	1653100	1100	1	1.61E-05	21	1.909090909	LOC106818347;emc4	Golgi
DMRcontig00763:1662301	1662301	1662500	200	1	5.29E-07	12	6	NA	NA
DMRcontig00767:1670201	1670201	1671600	1400	2	0.000227913	6	0.428571429	Gm7993;Cnot6l	Transcription;Unknown
DMRcontig00771:1678901	1678901	1679300	400	2	4.67E-05	11	2.75	NA	NA
DMRcontig00774:1685101	1685101	1685700	600	1	0.000502281	30	5	NA	NA
DMRcontig00830:1794901	1794901	1795000	100	1	0.000488783	6	6	rps16	Translation
DMRcontig00831:1796601	1796601	1796900	300	1	0.000296438	10	3.333333333	NA	NA
DMRcontig00846:1826501	1826501	1826900	400	1	7.06E-05	18	4.5	BRAFLDRAFT_99641;Rps15	Unknown;Translation
DMRcontig00849:1832301	1832301	1833200	900	1	0.000452782	16	1.777777778	NA	NA
DMRcontig00863:1860901	1860901	1861100	200	1	0.000884804	10	5	NA	NA
DMRcontig00882:1900401	1900401	1901400	1000	1	0.000172087	52	5.2	LOC102061525;HSPA8	Protein Binding
DMRcontig00887:1910943	1910943	1912000	1058	3	0.000308263	31	2.930056711	NA	NA
DMRcontig00900:1936501	1936501	1937600	1100	2	0.00066917	33	3	F751_6051	Binding Protein
DMRcontig00917:1971801	1971801	1972600	800	2	0.000119411	40	5	NA	NA
DMRcontig00923:1984901	1984901	1985400	500	1	0.000944803	21	4.2	bcas2	Translation
DMRcontig00929:1997501	1997501	1997700	200	1	0.000188596	7	3.5	NA	NA
DMRcontig00938:2014401	2014401	2015197	797	3	4.68E-06	19	2.383939774	NA	NA
DMRcontig00952:2041901	2041901	2042900	1000	2	1.98E-06	32	3.2	NA	NA
DMRcontig00954:2045733	2045733	2046400	668	1	8.82E-05	36	5.389221557	LOC106066852	Unknown
DMRcontig00967:2073001	2073001	2073300	300	2	2.76E-08	18	6	NA	NA
DMRcontig00978:2095601	2095601	2096200	600	1	0.000628165	22	3.666666667	STEHIDRAFT_152570;rpt-2	Unknown;Proteolysis
DMRcontig00993:2125801	2125801	2126600	800	2	6.21E-06	16	2	NA	NA
DMRcontig01001:2141201	2141201	2142400	1200	2	0.000514992	34	2.833333333	NA	NA
DMRcontig01002:2143566	2143566	2144300	735	2	0.00021893	14	1.904761905	NA	NA
DMRcontig01007:2154301	2154301	2155000	700	1	0.000150834	22	3.142857143	NA	NA
DMRcontig01044:2233101	2233101	2233400	300	1	0.000680484	4	1.333333333	LOC106492973	Unknown
DMRcontig01050:2247101	2247101	2247800	700	2	1.61E-05	35	5	NA	NA
DMRcontig01054:2257001	2257001	2257400	400	1	0.000971916	19	4.75	NA	NA
DMRcontig01069:2285401	2285401	2285900	500	1	0.000352075	6	1.2	COX1;ATP8	Metabolism
DMRcontig01078:2302501	2302501	2303000	500	1	0.000259134	23	4.6	NA	NA
DMRcontig01093:2331601	2331601	2332497	897	4	0.000377363	14	1.560758082	NA	NA
DMRcontig01100:2347201	2347201	2348000	800	2	6.54E-05	35	4.375	NA	NA
DMRcontig01115:2376801	2376801	2377976	1176	1	1.27E-06	53	4.506802721	NA	NA
DMRcontig01136:2417701	2417701	2418134	434	2	2.22E-05	25	5.760368664	LOC101859096	Translation
DMRcontig01144:2433701	2433701	2434300	600	1	0.000985438	25	4.166666667	NA	NA
DMRcontig01148:2442001	2442001	2442400	400	1	0.00014372	13	3.25	LOC102045438;PUF60	Translation
DMRcontig01150:2445701	2445701	2447000	1300	1	0.000925752	55	4.230769231	LOC105383268;mdh1	Metabolism
DMRcontig01156:2458601	2458601	2459300	700	2	4.16E-05	21	3	NA	NA
DMRcontig01183:2511850	2511850	2513261	1412	5	2.54E-05	89	6.303116147	NA	NA
DMRcontig01192:2530901	2530901	2532000	1100	3	9.38E-07	70	6.363636364	NA	NA
DMRcontig01200:2547401	2547401	2547700	300	1	3.87E-06	11	3.666666667	NA	NA
DMRcontig01209:2566096	2566096	2566600	505	1	0.000481896	33	6.534653465	NA	NA
DMRcontig01219:2586327	2586327	2586800	474	1	0.000608572	6	1.265822785	NA	NA
DMRcontig01224:2596101	2596101	2596400	300	2	5.62E-05	13	4.333333333	NA	NA
DMRcontig01234:2616401	2616401	2617652	1252	4	1.00E-07	79	6.309904153	NA	NA
DMRcontig01244:2637301	2637301	2637400	100	1	0.000757533	4	4	LOC106058859;LOC106062633	Unknown
DMRcontig01248:2644701	2644701	2645400	700	1	0.000142841	19	2.714285714	NA	NA
DMRcontig01254:2658001	2658001	2658700	700	1	8.28E-05	18	2.571428571	NA	NA
DMRcontig01256:2661901	2661901	2663000	1100	3	9.58E-06	75	6.818181818	NA	NA
DMRcontig01265:2679301	2679301	2680000	700	5	1.43E-08	39	5.571428571	LOC101874104;RHOC	Signaling
DMRcontig01286:2720301	2720301	2720800	500	1	3.13E-06	11	2.2	NA	NA

DMRcontig01308:2763401	2763401	2764000	600	2	2.34E-07	23	3.833333333	NA	NA
DMRcontig01312:2770484	2770484	2771000	517	2	0.000142991	25	4.835589942	mlnl;NOP56	Unknown;Translation
DMRcontig01314:2775501	2775501	2775900	400	2	3.41E-06	23	5.75	NA	NA
DMRcontig01351:2845601	2845601	2846000	400	1	0.000369332	29	7.25	NA	NA
DMRcontig01357:2857701	2857701	2859500	1800	1	0.000905932	28	1.555555556	NA	NA
DMRcontig01359:2863001	2863001	2864200	1200	1	1.53E-05	46	3.833333333	PSMB4	Proteolysis
DMRcontig01386:2917701	2917701	2918000	300	1	0.000176188	12	4	NA	NA
DMRcontig01390:2926401	2926401	2926900	500	1	6.59E-06	34	6.8	NA	NA
DMRcontig01423:2997301	2997301	2998400	1100	1	0.000335047	58	5.272727273	NA	NA
DMRcontig01427:3004601	3004601	3005100	500	3	1.92E-05	17	3.4	NA	NA
DMRcontig01438:3027001	3027001	3027300	300	1	5.39E-05	18	6	LOC103511100	Cytoskeleton
DMRcontig01445:3043001	3043001	3043900	900	1	0.000345101	31	3.444444444	NA	NA
DMRcontig01446:3046501	3046501	3046700	200	1	0.000321238	3	1.5	NA	NA
DMRcontig01453:3059201	3059201	3059600	400	2	5.85E-06	10	2.5	NA	NA
DMRcontig01459:3070901	3070901	3071200	300	1	0.00031503	11	3.666666667	NA	NA
DMRcontig01472:3095201	3095201	3095600	400	1	0.000132391	28	7	LOC109405848;RPS3	Translation
DMRcontig01473:3097101	3097101	3097800	700	1	0.000303574	40	5.714285714	NA	NA
DMRcontig01488:3127601	3127601	3128700	1100	2	2.79E-07	50	4.545454545	calm1	Signaling
DMRcontig01509:3170901	3170901	3171300	400	1	0.000728636	10	2.5	NA	NA
DMRcontig01511:3174586	3174586	3175200	615	1	6.51E-05	11	1.788617886	NA	NA
DMRcontig01519:3189801	3189801	3190300	500	2	0.00010098	33	6.6	NSDHL	Metabolism
DMRcontig01525:3201301	3201301	3201500	200	1	0.000353318	16	8	NA	NA
DMRcontig01526:3203172	3203172	3204120	949	1	6.92E-05	51	5.374077977	SLC34A1	Transport
DMRcontig01537:3223701	3223701	3224200	500	3	7.08E-09	23	4.6	NA	NA
DMRcontig01538:3226101	3226101	3226387	287	1	5.81E-05	7	2.43902439	NA	NA
DMRcontig01542:3233601	3233601	3234806	1206	1	0.000126216	46	3.814262023	NA	NA
DMRcontig01543:3236201	3236201	3236600	400	2	0.000563559	9	2.25	NA	NA
DMRcontig01555:3258201	3258201	3260000	1800	3	1.02E-06	72	4	NA	NA
DMRcontig01567:3282701	3282701	3283700	1000	1	1.90E-05	46	4.6	LOC101863981	Transcription
DMRcontig01603:3349012	3349012	3349600	589	3	4.51E-07	18	3.056027165	NA	NA
DMRcontig01623:3387901	3387901	3388400	500	2	0.000600677	17	3.4	NA	NA
DMRcontig01629:3398601	3398601	3399657	1057	1	5.77E-05	53	5.014191107	LOC105902522;PPP1CA	Signaling
DMRcontig01649:3437001	3437001	3437300	300	1	0.000482416	16	5.333333333	NA	NA
DMRcontig01666:3470701	3470701	3471000	300	2	2.48E-05	14	4.666666667	NA	NA
DMRcontig01684:3504301	3504301	3505100	800	2	0.000514189	39	4.875	NA	NA
DMRcontig01694:3524901	3524901	3525200	300	1	0.000105846	8	2.666666667	NA	NA
DMRcontig01729:3593901	3593901	3594300	400	1	0.000401125	13	3.25	NA	NA
DMRcontig01741:3615569	3615569	3616000	432	2	0.000272708	12	2.777777778	COX1	Metabolism
DMRcontig01749:3631101	3631101	3631800	700	1	0.000722014	19	2.714285714	NA	NA
DMRcontig01752:3638301	3638301	3638700	400	1	0.000482444	15	3.75	NA	NA
DMRcontig01754:3642001	3642001	3642300	300	2	4.25E-05	16	5.333333333	NA	NA
DMRcontig01775:3682301	3682301	3683300	1000	1	0.000452382	34	3.4	NA	NA
DMRcontig01780:3692502	3692502	3692700	199	2	0.000191847	4	2.010050251	NA	NA
DMRcontig01783:3698201	3698201	3698500	300	1	0.00017756	10	3.333333333	NA	NA
DMRcontig01787:3706801	3706801	3707000	200	1	0.000172489	1	0.5	NA	NA
DMRcontig01802:3736501	3736501	3736900	400	2	0.000129871	21	5.25	NA	NA
DMRcontig01811:3754701	3754701	3756100	1400	1	0.000529493	46	3.285714286	NA	NA
DMRcontig01828:3788101	3788101	3788200	100	1	0.000621158	5	5	CpipJ_CPIJ002413;uba52	Unknown;Translation
DMRcontig01829:3789501	3789501	3790600	1100	5	4.83E-05	31	2.818181818	NA	NA
DMRcontig01833:3798101	3798101	3798400	300	1	0.000798302	4	1.333333333	NA	NA
DMRcontig01842:3816501	3816501	3817000	500	1	0.000346221	29	5.8	NA	NA
DMRcontig01851:3833101	3833101	3834300	1200	1	0.000937256	31	2.583333333	NA	NA
DMRcontig01852:3835650	3835650	3836000	351	1	0.000103294	10	2.849002849	NA	NA
DMRcontig01858:3846101	3846101	3846400	300	1	0.000954531	2	0.666666667	trnal-cag;LOC106957352;sstr1a	Transcription;Receptor;Epigenetic
DMRcontig01873:3875701	3875701	3875800	100	1	0.000895327	2	2	NA	NA
DMRcontig01874:3877401	3877401	3877600	200	1	0.000920011	10	5	NA	NA
DMRcontig01876:3882001	3882001	3882400	400	1	1.76E-05	22	5.5	LOC106609902	Metabolism
DMRcontig01901:3929801	3929801	3930079	279	1	6.28E-06	22	7.885304659	LOC102234399	Metabolism
DMRcontig01908:3942301	3942301	3942500	200	1	0.000949091	13	6.5	PTSG_11670;LOC100076533;MAJ_05279;GAPDH	Metabolism;Unknown
DMRcontig01909:3945101	3945101	3945300	200	2	0.000288851	9	4.5	NA	NA
DMRcontig01918:3961701	3961701	3962900	1200	3	1.20E-06	44	3.666666667	NA	NA
DMRcontig01924:3972801	3972801	3973100	300	1	0.000803579	14	4.666666667	NA	NA
DMRcontig01925:3975101	3975101	3975900	800	1	4.91E-05	37	4.625	NA	NA
DMRcontig01935:3994301	3994301	3994500	200	1	0.000389849	3	1.5	NA	NA
DMRcontig01939:4002701	4002701	4002900	200	1	0.000320369	10	5	Dsec\GM16740;RpLP1	Unknown;Translation
DMRcontig01944:4011401	4011401	4011600	200	1	0.000658404	11	5.5	NA	NA
DMRcontig01946:4015901	4015901	4016300	400	1	0.000380828	7	1.75	NA	NA
DMRcontig01951:4025601	4025601	4025800	200	1	9.41E-05	13	6.5	NA	NA
DMRcontig01952:4027901	4027901	4029000	1100	1	0.000164758	52	4.727272727	NA	NA
DMRcontig01964:4053401	4053401	4054140	740	2	9.54E-06	17	2.297297297	NA	NA
DMRcontig01976:4075901	4075901	4076400	500	1	0.000942682	17	3.4	NA	NA
DMRcontig01984:4090801	4090801	4091800	1000	1	3.18E-05	30	3	NA	NA
DMRcontig01987:4096401	4096401	4097454	1054	4	3.67E-07	43	4.079696395	NA	NA
DMRcontig02006:4134701	4134701	4135300	600	1	5.90E-05	38	6.333333333	NA	NA
DMRcontig02031:4180301	4180301	4181051	751	2	0.000712973	28	3.728362184	NA	NA

DMRcontig02034:4185801	4185801	4186300	500	1	0.000315859	20	4	NA	NA
DMRcontig02042:4202801	4202801	4202952	152	1	0.000907391	1	0.657894737	NA	NA
DMRcontig02043:4203953	4203953	4205000	1048	3	0.000178538	46	4.389312977	NA	NA
DMRcontig02049:4215501	4215501	4216000	500	1	0.00072878	31	6.2	NA	NA
DMRcontig02052:4220201	4220201	4220500	300	2	0.000272738	6	2	NA	NA
DMRcontig02061:4235820	4235820	4236700	881	1	0.000744584	20	2.27014756	NA	NA
DMRcontig02064:4243501	4243501	4244400	900	2	0.000717506	40	4.444444444	LOC101656948;HIPK2	Signaling;Development
DMRcontig02078:4270301	4270301	4271000	700	4	0.000107489	46	6.571428571	NA	NA
DMRcontig02081:4276188	4276188	4276800	613	1	0.000647748	33	5.383360522	NA	NA
DMRcontig02082:4278401	4278401	4278900	500	1	6.34E-05	31	6.2	NA	NA
DMRcontig02110:4333849	4333849	4335500	1652	9	3.86E-05	61	3.692493947	NA	NA
DMRcontig02129:4367501	4367501	4368700	1200	3	4.97E-05	34	2.833333333	NA	NA
DMRcontig02132:4373601	4373601	4374200	600	1	0.00014024	27	4.5	NA	NA
DMRcontig02171:4448601	4448601	4449400	800	2	0.000317996	26	3.25	NA	NA
DMRcontig02182:4468601	4468601	4468800	200	1	5.85E-06	20	10	NA	NA
DMRcontig02193:4488428	4488428	4488700	273	1	0.000257772	10	3.663003663	NA	NA
DMRcontig02197:4495301	4495301	4495500	200	1	5.61E-05	6	3	LOC103528366;pgk1	Signaling
DMRcontig02221:4540701	4540701	4541206	506	1	3.40E-05	30	5.928853755	NA	NA
DMRcontig02231:4559401	4559401	4559500	100	1	0.000911933	3	3	NA	NA
DMRcontig02246:4586201	4586201	4586700	500	2	1.68E-07	10	2	NA	NA
DMRcontig02247:4587801	4587801	4588358	558	3	8.88E-06	42	7.52688172	NA	NA
DMRcontig02260:4614001	4614001	4614300	300	1	0.000150154	18	6	NA	NA
DMRcontig02268:4627401	4627401	4627700	300	1	0.000181944	17	5.666666667	NA	NA
DMRcontig02291:4669801	4669801	4669900	100	1	0.000270054	1	1	NA	NA
DMRcontig02337:4755201	4755201	4755500	300	1	0.000727512	5	1.666666667	NA	NA
DMRcontig02340:4760401	4760401	4760961	561	3	4.50E-06	6	1.069518717	COX1	Metabolism
DMRcontig02341:4762001	4762001	4762630	630	4	2.01E-05	30	4.761904762	NA	NA
DMRcontig02365:4804301	4804301	4805300	1000	1	7.82E-05	22	2.2	NA	NA
DMRcontig02373:4820401	4820401	4821600	1200	2	0.000349953	52	4.333333333	cyc1.L	Metabolism
DMRcontig02385:4842536	4842536	4842900	365	2	0.000308724	8	2.191780822	BRAFLDRAFT_81006	Unknown
DMRcontig02388:4848201	4848201	4849000	800	1	0.00071447	45	5.625	NA	NA
DMRcontig02400:4872619	4872619	4873600	982	2	4.15E-07	28	2.851323829	NA	NA
DMRcontig02401:4875101	4875101	4875500	400	1	0.00018967	26	6.5	NA	NA
DMRcontig02412:4894701	4894701	4895400	700	2	3.83E-07	22	3.142857143	NA	NA
DMRcontig02426:4921101	4921101	4921900	800	2	0.000171222	33	4.125	NA	NA
DMRcontig02436:4939474	4939474	4940500	1027	2	0.000472875	13	1.265822785	NA	NA
DMRcontig02439:4946001	4946001	4946300	300	2	1.30E-05	17	5.666666667	NA	NA
DMRcontig02445:4956101	4956101	4956400	300	1	0.000991638	17	5.666666667	LOC106495449;PSMA5	Unknown;Protease
DMRcontig02446:4957716	4957716	4958500	785	2	2.06E-06	16	2.038216561	NA	NA
DMRcontig02447:4959801	4959801	4960300	500	1	1.76E-05	14	2.8	NA	NA
DMRcontig02449:4963801	4963801	4964000	200	1	0.00023587	9	4.5	NA	NA
DMRcontig02469:4999601	4999601	5000100	500	2	1.39E-05	20	4	NA	NA
DMRcontig02472:5004701	5004701	5005300	600	2	5.11E-06	31	5.166666667	NA	NA
DMRcontig02474:5008133	5008133	5008691	559	2	2.98E-05	24	4.293381038	NA	NA
DMRcontig02476:5011101	5011101	5012500	1400	1	0.000687754	57	4.071428571	tcpb	Development
DMRcontig02478:5015312	5015312	5016700	1389	2	4.45E-05	48	3.455723542	NA	NA
DMRcontig02490:5038001	5038001	5038400	400	2	3.87E-06	28	7	NA	NA
DMRcontig02495:5046401	5046401	5046700	300	1	0.000130212	12	4	NA	NA
DMRcontig02513:5075901	5075901	5076175	275	1	0.000828693	12	4.363636364	VOLCADRAFT_115993	Unknown
DMRcontig02549:5141401	5141401	5142300	900	1	9.31E-05	63	7	kmt2d	Epigenetic
DMRcontig02552:5147901	5147901	5148391	491	1	0.000877101	18	3.66598778	NA	NA
DMRcontig02562:5164801	5164801	5166000	1200	2	0.000215071	30	2.5	NA	NA
DMRcontig02573:5188001	5188001	5188800	800	2	3.40E-06	60	7.5	NA	NA
DMRcontig02574:5190001	5190001	5190400	400	1	0.000153219	11	2.75	NA	NA
DMRcontig02579:5200101	5200101	5200400	300	1	0.000426479	15	5	NA	NA
DMRcontig02581:5203301	5203301	5203800	500	1	0.000405964	21	4.2	NA	NA
DMRcontig02629:5292701	5292701	5294000	1300	1	0.000968621	53	4.076923077	LOC106052646;rab21	Signaling
DMRcontig02664:5359142	5359142	5359700	559	2	9.64E-05	15	2.683363148	NA	NA
DMRcontig02690:5406617	5406617	5407400	784	1	0.000368529	27	3.443877551	NA	NA
DMRcontig02693:5413201	5413201	5413639	439	1	0.000448333	11	2.505694761	NA	NA
DMRcontig02713:5447887	5447887	5448800	914	1	1.47E-06	44	4.814004376	NA	NA
DMRcontig02718:5457201	5457201	5458200	1000	2	0.000336478	42	4.2	NA	NA
DMRcontig02757:5527901	5527901	5528700	800	1	0.000977398	43	5.375	LOC105896849	Protein Modification
DMRcontig02780:5570701	5570701	5571500	800	3	4.33E-07	65	8.125	NA	NA
DMRcontig02799:5605066	5605066	5605600	535	3	1.32E-05	14	2.61682243	NA	NA
DMRcontig02805:5617401	5617401	5617968	568	1	0.000380828	11	1.936619718	NA	NA
DMRcontig02806:5618969	5618969	5619300	332	1	7.82E-06	12	3.614457831	NA	NA
DMRcontig02810:5626301	5626301	5626795	495	1	0.000539776	40	8.080808081	NA	NA
DMRcontig02833:5669101	5669101	5669400	300	2	0.000118515	12	4	NA	NA
DMRcontig02839:5679301	5679301	5679600	300	1	0.000117484	11	3.666666667	pkd2	Transport
DMRcontig02850:5698201	5698201	5698500	300	1	2.13E-05	20	6.666666667	NA	NA
DMRcontig02854:5704518	5704518	5704700	183	1	1.39E-05	6	3.278688525	NA	NA
DMRcontig02885:5761901	5761901	5762066	166	1	0.000241992	7	4.21686747	NA	NA
DMRcontig02892:5776201	5776201	5776800	600	2	5.30E-05	28	4.666666667	LOC106814012	Signaling
DMRcontig02909:5808901	5808901	5809300	400	1	0.000878043	21	5.25	NA	NA
DMRcontig02935:5857701	5857701	5857900	200	1	0.000346111	7	3.5	NA	NA

DMRcontig02944:5872220	5872220	5872700	481	4	3.47E-07	33	6.860706861	NA	NA
DMRcontig02964:5906601	5906601	5907600	1000	1	1.66E-05	47	4.7	NA	NA
DMRcontig02972:5921001	5921001	5921500	500	1	4.13E-06	9	1.8	NA	NA
DMRcontig02991:5954101	5954101	5954500	400	2	3.14E-06	9	2.25	LOC107092765;SETD1A	Epigenetic;Transcription
DMRcontig02999:5968939	5968939	5969600	662	1	2.33E-05	17	2.567975831	NA	NA
DMRcontig03004:5978301	5978301	5978500	200	1	1.28E-06	10	5	NA	NA
DMRcontig03015:5996801	5996801	5997300	500	1	3.00E-05	28	5.6	NA	NA
DMRcontig03021:6008901	6008901	6009166	266	1	3.92E-05	12	4.511278195	NA	NA
DMRcontig03027:6019801	6019801	6020600	800	4	2.56E-06	49	6.125	NA	NA
DMRcontig03091:6134601	6134601	6135000	400	2	8.64E-05	7	1.75	NA	NA
DMRcontig03092:6136222	6136222	6137300	1079	3	4.03E-05	36	3.336422614	NA	NA
DMRcontig03109:6166401	6166401	6166900	500	2	0.00024498	9	1.8	NA	NA
DMRcontig03111:6170001	6170001	6171300	1300	2	0.000405958	66	5.076923077	NA	NA
DMRcontig03126:6198601	6198601	6199000	400	1	0.000773413	20	5	NA	NA
DMRcontig03149:6241601	6241601	6242143	543	1	0.000977759	18	3.314917127	PCHAS_104270	Unknown
DMRcontig03189:6309830	6309830	6310900	1071	1	8.43E-05	17	1.587301587	NA	NA
DMRcontig03248:6417501	6417501	6419000	1500	1	0.000191095	59	3.933333333	CNDP2	Protease
DMRcontig03256:6433876	6433876	6434500	625	1	0.000858223	30	4.8	NA	NA
DMRcontig03279:6474201	6474201	6474500	300	1	0.000641412	1	0.333333333	NA	NA
DMRcontig03283:6481301	6481301	6481600	300	2	0.000354537	10	3.333333333	NA	NA
DMRcontig03285:6485101	6485101	6485835	735	3	3.04E-05	38	5.170068027	LOC100160485	Unknown
DMRcontig03298:6508901	6508901	6509323	423	1	0.000805914	15	3.546099291	NA	NA
DMRcontig03308:6526631	6526631	6527100	470	3	0.000453646	28	5.957446809	LOC108190067;mvk	Unknown;Signaling
DMRcontig03319:6545443	6545443	6545900	458	1	0.000344692	24	5.240174672	CGGC5_2627;rpsa	Translation
DMRcontig03340:6583268	6583268	6584000	733	1	0.00033573	20	2.72851296	NA	NA
DMRcontig03360:6619701	6619701	6619900	200	1	5.71E-06	9	4.5	NA	NA
DMRcontig03361:6621901	6621901	6622300	400	2	1.26E-06	24	6	NA	NA
DMRcontig03374:6643301	6643301	6643700	400	1	0.000160584	27	6.75	NA	NA
DMRcontig03402:6693970	6693970	6695000	1031	3	5.13E-07	25	2.424830262	NA	NA
DMRcontig03403:6696501	6696501	6696700	200	1	0.000778891	10	5	NA	NA
DMRcontig03413:6713301	6713301	6714400	1100	2	0.000339833	47	4.272727273	NA	NA
DMRcontig03468:6808601	6808601	6809300	700	1	0.000126927	49	7	LOC105451083;LOC105569752	Protease
DMRcontig03469:6810601	6810601	6810800	200	1	0.000422127	16	8	NA	NA
DMRcontig03471:6814301	6814301	6814700	400	1	0.000848451	23	5.75	NA	NA
DMRcontig03477:6824701	6824701	6825800	1100	2	5.15E-06	33	3	NA	NA
DMRcontig03480:6830833	6830833	6831435	603	5	1.59E-05	31	5.140961857	NA	NA
DMRcontig03481:6832601	6832601	6833686	1086	2	2.02E-06	49	4.511970534	NA	NA
DMRcontig03503:6872401	6872401	6872800	400	2	0.000507355	24	6	NA	NA
DMRcontig03516:6894001	6894001	6894300	300	1	0.000101139	11	3.666666667	NA	NA
DMRcontig03524:6909101	6909101	6909900	800	2	4.35E-06	27	3.375	NA	NA
DMRcontig03534:6926893	6926893	6927300	408	1	0.000527158	16	3.921568627	NA	NA
DMRcontig03535:6928582	6928582	6928700	119	1	0.000236555	10	8.403361345	NA	NA
DMRcontig03551:6958001	6958001	6958997	997	1	0.000150834	38	3.811434303	NA	NA
DMRcontig03562:6977501	6977501	6977900	400	2	0.000361571	24	6	SLC25A37	Binding Protein
DMRcontig03570:6993401	6993401	6993600	200	1	0.00064638	5	2.5	NA	NA
DMRcontig03603:7048301	7048301	7048844	544	1	0.000642028	6	1.102941176	NA	NA
DMRcontig03624:7084001	7084001	7084300	300	2	7.48E-07	31	10.33333333	NA	NA
DMRcontig03663:7151701	7151701	7152800	1100	2	3.51E-07	26	2.363636364	NA	NA
DMRcontig03696:7212101	7212101	7212500	400	1	0.000866321	16	4	NA	NA
DMRcontig03706:7232101	7232101	7232600	500	1	0.000357289	32	6.4	NA	NA
DMRcontig03718:7252201	7252201	7252400	200	1	0.000729413	6	3	NA	NA
DMRcontig03723:7259901	7259901	7260400	500	1	5.75E-05	27	5.4	NA	NA
DMRcontig03724:7261601	7261601	7262000	400	2	1.31E-05	13	3.25	NA	NA
DMRcontig03727:7267601	7267601	7267675	75	1	0.000313703	3	4	NA	NA
DMRcontig03737:7284501	7284501	7284700	200	1	0.000293435	4	2	NA	NA
DMRcontig03740:7289701	7289701	7290100	400	1	0.000223031	16	4	NA	NA
DMRcontig03746:7300401	7300401	7300600	200	1	0.000308815	1	0.5	NA	NA
DMRcontig03758:7321261	7321261	7321400	140	2	0.000192836	2	1.428571429	NA	NA
DMRcontig03770:7342201	7342201	7343000	800	2	0.000649417	21	2.625	NA	NA
DMRcontig03778:7356701	7356701	7357500	800	2	1.90E-05	33	4.125	NA	NA
DMRcontig03780:7360141	7360141	7360900	760	2	4.01E-05	44	5.789473684	MICPUN_101755;MICPUN_768 56	Epigenetic;Unknown
DMRcontig03784:7367801	7367801	7368200	400	1	0.000275243	12	3	LOC105214743;rtn4	Transport
DMRcontig03791:7379801	7379801	7380900	1100	2	0.000533244	37	3.363636364	NA	NA
DMRcontig03798:7392540	7392540	7393000	461	1	4.05E-05	13	2.819956616	ETH_00034195	Signaling
DMRcontig03809:7412233	7412233	7412700	468	2	0.000191393	8	1.709401709	NA	NA
DMRcontig03812:7417501	7417501	7418400	900	1	0.000408357	39	4.333333333	LOC106932392	Unknown
DMRcontig03818:7428301	7428301	7428900	600	1	0.000691279	29	4.833333333	NA	NA
DMRcontig03822:7435401	7435401	7436100	700	1	6.15E-06	5	0.714285714	NA	NA
DMRcontig03824:7439801	7439801	7440000	200	1	7.74E-05	16	8	NA	NA
DMRcontig03830:7449901	7449901	7450300	400	2	1.74E-06	7	1.75	NA	NA
DMRcontig03844:7476618	7476618	7477200	583	4	2.34E-05	14	2.401372213	NA	NA
DMRcontig03853:7492529	7492529	7493300	772	1	0.000281404	39	5.051813472	NA	NA
DMRcontig03868:7519101	7519101	7519300	200	1	0.000500081	9	4.5	NA	NA
DMRcontig03871:7523401	7523401	7524200	800	1	5.57E-07	29	3.625	TXNP5	Unknown
DMRcontig03873:7527801	7527801	7528400	600	2	0.000573725	28	4.666666667	LOC103374021	Signaling

DMRcontig03899:7572701	7572701	7572900	200	1	0.000245027	6	3	NA	NA
DMRcontig03909:7589901	7589901	7590200	300	1	0.000887944	13	4.333333333	NA	NA
DMRcontig03954:7668101	7668101	7668200	100	1	0.000386324	5	5	NA	NA
DMRcontig03962:7681001	7681001	7681400	400	1	0.000489707	33	8.25	NA	NA
DMRcontig03974:7702113	7702113	7703000	888	1	0.00018061	22	2.477477477	NA	NA
DMRcontig03976:7705535	7705535	7705800	266	1	0.000412969	9	3.383458647	NA	NA
DMRcontig03981:7713601	7713601	7713900	300	1	0.00032833	6	2	NA	NA
DMRcontig04002:7753501	7753501	7754100	600	1	0.000169943	25	4.166666667	NA	NA
DMRcontig04009:7766153	7766153	7767268	1116	3	1.09E-05	46	4.121863799	NA	NA
DMRcontig04015:7776401	7776401	7777160	760	1	0.000103688	27	3.552631579	LOC101855319	Binding Protein
DMRcontig04018:7781801	7781801	7783038	1238	1	0.000302879	36	2.907915994	NA	NA
DMRcontig04040:7821601	7821601	7822700	1100	4	3.18E-07	25	2.272727273	NA	NA
DMRcontig04057:7850501	7850501	7850900	400	1	0.0005476	9	2.25	NA	NA
DMRcontig04064:7862201	7862201	7862300	100	1	0.000277832	4	4	NA	NA
DMRcontig04068:7870101	7870101	7870666	566	2	5.21E-05	17	3.003533569	NA	NA
DMRcontig04071:7875301	7875301	7875500	200	1	0.000401735	5	2.5	NA	NA
DMRcontig04089:7906701	7906701	7906964	264	1	3.93E-05	10	3.787878788	DICSQDRAFT_60362;DICSQDRAFT_170084;RPL7A	Translation
DMRcontig04092:7912601	7912601	7912800	200	1	0.000309824	7	3.5	NA	NA
DMRcontig04098:7923101	7923101	7923800	700	1	0.000289133	30	4.285714286	NA	NA
DMRcontig04157:8024601	8024601	8025300	700	1	5.17E-07	39	5.571428571	NA	NA
DMRcontig04177:8058381	8058381	8059000	620	4	2.72E-09	27	4.35483871	NA	NA
DMRcontig04180:8063301	8063301	8063400	100	1	0.000686306	7	7	LOC104947728;epb41l3	Cytoskeleton;Unknown
DMRcontig04206:8108701	8108701	8109100	400	1	0.000339173	20	5	NA	NA
DMRcontig04254:8190401	8190401	8191300	900	3	0.000337611	38	4.222222222	NA	NA
DMRcontig04267:8213120	8213120	8213600	481	2	9.46E-05	28	5.821205821	LOC101845985	Cytoskeleton
DMRcontig04286:8247414	8247414	8248092	679	2	0.000232287	16	2.35640648	NA	NA
DMRcontig04291:8256032	8256032	8256700	669	1	0.000890531	37	5.53064275	NA	NA
DMRcontig04321:8310101	8310101	8310400	300	1	0.000884208	19	6.333333333	BRAFLDRAFT_218343	Unknown
DMRcontig04344:8347766	8347766	8348092	327	2	5.09E-06	10	3.058103976	NA	NA
DMRcontig04374:8402501	8402501	8403200	700	1	0.000383581	45	6.428571429	NA	NA
DMRcontig04401:8451101	8451101	8451200	100	1	0.000281845	8	8	NA	NA
DMRcontig04415:8475156	8475156	8475800	645	5	7.00E-06	26	4.031007752	NA	NA
DMRcontig04429:8498001	8498001	8498600	600	1	3.47E-05	28	4.666666667	NA	NA
DMRcontig04433:8505301	8505301	8505700	400	1	8.91E-05	15	3.75	NA	NA
DMRcontig04435:8509501	8509501	8509660	160	1	0.000266007	3	1.875	NA	NA
DMRcontig04444:8525501	8525501	8525604	104	1	0.000536676	0	0	NA	NA
DMRcontig04454:8542340	8542340	8543400	1061	1	0.00017097	17	1.602262017	NA	NA
DMRcontig04495:8614401	8614401	8614600	200	1	0.000184952	11	5.5	LOC103574110	Translation
DMRcontig04534:8682701	8682701	8683400	700	1	0.000530052	34	4.857142857	NA	NA
DMRcontig04540:8693801	8693801	8694100	300	1	0.000360472	15	5	LOC106164039	Metabolism
DMRcontig04542:8697701	8697701	8698089	389	2	0.000513739	8	2.05655527	NA	NA
DMRcontig04564:8733101	8733101	8733800	700	3	5.50E-05	28	4	NA	NA
DMRcontig04569:8742113	8742113	8742500	388	2	1.88E-06	10	2.577319588	NA	NA
DMRcontig04621:8831001	8831001	8831700	700	5	1.16E-06	46	6.571428571	NA	NA
DMRcontig04645:8870701	8870701	8871100	400	2	0.000432604	4	1	NA	NA
DMRcontig04658:8893301	8893301	8893800	500	2	7.14E-07	13	2.6	NA	NA
DMRcontig04664:8903901	8903901	8904494	594	3	3.51E-05	16	2.693602694	NA	NA
DMRcontig04678:8928401	8928401	8929500	1100	1	0.000255755	21	1.909090909	LOC101070845	Metabolism
DMRcontig04714:8993250	8993250	8993800	551	3	2.58E-05	20	3.629764065	NA	NA
DMRcontig04726:9014001	9014001	9014600	600	1	2.82E-05	24	4	NA	NA
DMRcontig04743:9042901	9042901	9043100	200	2	0.000291653	5	2.5	NA	NA
DMRcontig04745:9046601	9046601	9047200	600	4	1.65E-06	7	1.166666667	NA	NA
DMRcontig04750:9055001	9055001	9056000	1000	2	0.000465059	41	4.1	NA	NA
DMRcontig04752:9058801	9058801	9059100	300	1	6.87E-05	17	5.666666667	NA	NA
DMRcontig04756:9066773	9066773	9067300	528	1	2.09E-05	27	5.113636364	LOC106612978	Extracellular Matrix
DMRcontig04758:9070501	9070501	9070800	300	1	0.000618345	20	6.666666667	NA	NA
DMRcontig04782:9113401	9113401	9114200	800	1	5.61E-05	30	3.75	NA	NA
DMRcontig04783:9115801	9115801	9116100	300	1	0.000231523	14	4.666666667	NA	NA
DMRcontig04790:9126601	9126601	9127100	500	4	9.43E-08	24	4.8	NA	NA
DMRcontig04804:9150459	9150459	9150800	342	2	1.99E-05	17	4.970760234	NA	NA
DMRcontig04814:9166666	9166666	9167000	335	1	5.90E-05	5	1.492537313	NA	NA
DMRcontig04837:9206225	9206225	9207500	1276	2	0.000119268	21	1.645768025	NA	NA
DMRcontig04858:9242001	9242001	9242200	200	1	0.000409054	3	1.5	NA	NA
DMRcontig04886:9288101	9288101	9288400	300	1	0.000988136	20	6.666666667	NA	NA
DMRcontig04894:9301501	9301501	9302000	500	2	0.000280893	16	3.2	NA	NA
DMRcontig04904:9318201	9318201	9318500	300	1	6.04E-05	6	2	NA	NA
DMRcontig04920:9345211	9345211	9346100	890	2	1.75E-06	41	4.606741573	NA	NA
DMRcontig04958:9410055	9410055	9410400	346	3	2.38E-07	17	4.913294798	NA	NA
DMRcontig04974:9439001	9439001	9439300	300	1	0.00044418	15	5	NA	NA
DMRcontig04975:9441201	9441201	9441500	300	1	5.01E-05	6	2	NA	NA
DMRcontig04988:9463101	9463101	9463400	300	1	0.000101693	16	5.333333333	BRAFLDRAFT_120096	Epigenetic
DMRcontig05029:9532001	9532001	9532480	480	2	4.03E-06	13	2.708333333	NA	NA
DMRcontig05036:9543001	9543001	9543100	100	1	0.000689167	4	4	NA	NA
DMRcontig05047:9562701	9562701	9563000	300	1	0.000799006	14	4.666666667	NA	NA
DMRcontig05077:9615901	9615901	9616300	400	1	4.41E-05	23	5.75	LOC106746683	Signaling

DMRcontig05079:9618974	9618974	9619427	454	3	5.62E-05	34	7.488986784	NA	NA
DMRcontig05096:9648301	9648301	9648900	600	3	4.42E-05	28	4.666666667	NA	NA
DMRcontig05132:9713301	9713301	9713700	400	1	0.00017414	9	2.25	NA	NA
DMRcontig05156:9754512	9754512	9755200	689	4	2.98E-05	24	3.483309144	NA	NA
DMRcontig05187:9807936	9807936	9808700	765	1	0.000225844	33	4.31372549	TRIADDRAFT_38444	Unknown
DMRcontig05189:9811445	9811445	9811900	456	2	0.000231379	25	5.48245614	NA	NA
DMRcontig05196:9823151	9823151	9823500	350	4	1.62E-07	18	5.142857143	NA	NA
DMRcontig05216:9856301	9856301	9856500	200	2	0.000402788	9	4.5	NA	NA
DMRcontig05220:9863201	9863201	9863400	200	1	2.59E-05	7	3.5	NA	NA
DMRcontig05235:9887262	9887262	9888319	1058	4	2.64E-06	27	2.551984877	NA	NA
DMRcontig05278:9962001	9962001	9962300	300	1	0.000296661	12	4	NA	NA
DMRcontig05289:9979567	9979567	9980272	706	1	0.000764342	12	1.699716714	NA	NA
DMRcontig05297:9992801	9992801	9993900	1100	1	0.000384812	33	3	NA	NA
DMRcontig05299:9996840	9996840	9997465	626	3	0.000130515	36	5.750798722	NA	NA
DMRcontig05305:1000650	1E+07	10007242	742	1	0.000155134	25	3.369272237	NA	NA
DMRcontig05312:1001860	1E+07	10019400	800	3	9.80E-06	40	5	NA	NA
DMRcontig05317:1002770	1E+07	10028100	400	1	0.000939011	21	5.25	NA	NA
DMRcontig05361:1010290	1E+07	10103176	276	2	5.97E-05	12	4.347826087	NA	NA
DMRcontig05367:1011410	1E+07	10114500	400	3	7.81E-06	27	6.75	NA	NA
DMRcontig05378:1013220	1E+07	10133100	900	1	0.00050252	42	4.666666667	NA	NA
DMRcontig05381:1013810	1E+07	10138483	383	2	0.000167368	20	5.221932115	NA	NA
DMRcontig05405:1017854	1E+07	10179200	653	2	6.04E-05	34	5.206738132	copa	Unknown
DMRcontig05439:1023400	1E+07	10234900	900	3	3.13E-05	45	5	NA	NA
DMRcontig05478:1030050	1E+07	10300700	200	1	0.000750339	10	5	CHLNCDRAFT_59713;pdhb	Unknown;Metabolism
DMRcontig05491:1032151	1E+07	10322100	589	1	0.000742706	24	4.074702886	NA	NA
DMRcontig05494:1032642	1E+07	10326800	377	1	0.000154024	8	2.122015915	NA	NA
DMRcontig05498:1033250	1E+07	10333400	900	1	0.000255452	43	4.777777778	NA	NA
DMRcontig05518:1036850	1E+07	10369100	600	2	3.36E-06	33	5.5	LOC101850811	Unknown
DMRcontig05530:1038830	1E+07	10388700	400	2	0.000532079	10	2.5	NA	NA
DMRcontig05533:1039340	1E+07	10393800	400	1	0.000761118	27	6.75	LOC101845884	Unknown
DMRcontig05538:1040200	1E+07	10402502	502	2	7.07E-05	35	6.972111554	NA	NA
DMRcontig05562:1044230	1E+07	10442900	600	2	1.28E-05	30	5	NA	NA
DMRcontig05564:1044600	1E+07	10446900	900	1	0.00095934	23	2.555555556	NA	NA
DMRcontig05565:1044810	1E+07	10449200	1100	1	0.000960276	15	1.363636364	NA	NA
DMRcontig05583:1048013	1E+07	10480663	531	1	0.000378007	24	4.519774011	NA	NA
DMRcontig05597:1050233	1.1E+07	10502800	471	1	0.000619063	10	2.123142251	NA	NA
DMRcontig05651:1059050	1.1E+07	10591200	700	1	0.000832561	45	6.428571429	NA	NA
DMRcontig05660:1060640	1.1E+07	10606700	300	2	3.61E-05	6	2	NA	NA
DMRcontig05666:1061660	1.1E+07	10616900	300	1	5.31E-05	5	1.666666667	NA	NA
DMRcontig05700:1067333	1.1E+07	10674500	1165	3	6.56E-05	32	2.746781116	NA	NA
DMRcontig05701:1067600	1.1E+07	10676500	500	2	2.00E-05	18	3.6	NA	NA
DMRcontig05709:1068840	1.1E+07	10688800	400	1	4.69E-05	22	5.5	NA	NA
DMRcontig05714:1069683	1.1E+07	10697700	864	2	5.38E-06	18	2.083333333	NA	NA
DMRcontig05715:1069900	1.1E+07	10699660	660	3	2.31E-05	21	3.181818182	GLOTRDRAFT_130935	Unknown
DMRcontig05738:1073780	1.1E+07	10738500	700	2	7.92E-05	19	2.714285714	NA	NA
DMRcontig05751:1075940	1.1E+07	10760000	600	2	0.000662457	31	5.166666667	NA	NA
DMRcontig05763:1078040	1.1E+07	10781000	600	1	0.000458262	16	2.666666667	NA	NA
DMRcontig05764:1078230	1.1E+07	10782700	400	1	0.000519367	8	2	NA	NA
DMRcontig05850:1092890	1.1E+07	10929400	500	2	0.000242962	13	2.6	NA	NA
DMRcontig05852:1093210	1.1E+07	10932900	800	1	0.000691195	19	2.375	NA	NA
DMRcontig05860:1094580	1.1E+07	10946300	500	1	3.57E-05	17	3.4	NA	NA
DMRcontig05862:1094940	1.1E+07	10949600	200	1	0.000912492	11	5.5	NA	NA
DMRcontig05872:1096640	1.1E+07	10966900	500	1	0.000159571	16	3.2	NA	NA
DMRcontig05938:1107870	1.1E+07	11078984	284	1	0.000647478	15	5.281690141	NA	NA
DMRcontig05941:1108370	1.1E+07	11084016	316	2	8.38E-07	18	5.696202532	NA	NA
DMRcontig05975:1113970	1.1E+07	11139900	200	1	0.000214477	14	7	NA	NA
DMRcontig05989:1116250	1.1E+07	11162775	275	1	8.94E-05	16	5.818181818	NA	NA
DMRcontig06059:1127900	1.1E+07	11279400	400	1	0.000168915	22	5.5	NA	NA
DMRcontig06062:1128380	1.1E+07	11284100	300	2	3.68E-06	12	4	NA	NA
DMRcontig06063:1128580	1.1E+07	11286679	879	1	0.000126818	49	5.574516496	NA	NA
DMRcontig06095:1133870	1.1E+07	11338900	200	1	0.000689167	3	1.5	NA	NA
DMRcontig06102:1135010	1.1E+07	11350459	359	2	7.17E-06	9	2.506963788	NA	NA
DMRcontig06135:1140572	1.1E+07	11406000	277	3	4.06E-07	7	2.527075812	NA	NA
DMRcontig06145:1142260	1.1E+07	11423200	600	1	4.80E-05	8	1.333333333	NA	NA
DMRcontig06157:1144404	1.1E+07	11444400	359	1	0.000258544	18	5.013927577	RAB11B	Signaling
DMRcontig06167:1146160	1.1E+07	11461800	200	1	0.000537963	15	7.5	NA	NA
DMRcontig06187:1149532	1.1E+07	11496000	679	2	0.000388525	18	2.65095729	NA	NA
DMRcontig06211:1153474	1.2E+07	11535300	555	1	0.00017075	9	1.621621622	NA	NA
DMRcontig06221:1155030	1.2E+07	11550800	500	1	0.000136542	16	3.2	NA	NA
DMRcontig06227:1156025	1.2E+07	11560900	648	1	0.00032871	8	1.234567901	NA	NA
DMRcontig06228:1156230	1.2E+07	11562900	600	5	4.19E-06	42	7	NA	NA
DMRcontig06287:1166120	1.2E+07	11661900	700	1	0.000729575	10	1.428571429	LOC105379109	Unknown
DMRcontig06292:1167003	1.2E+07	11670300	267	1	0.000981997	1	0.374531835	NA	NA
DMRcontig06304:1169022	1.2E+07	11690800	573	1	2.02E-05	39	6.806282723	Dmoj\GI14614;Dpse\GA14719	Unknown
DMRcontig06319:1171630	1.2E+07	11716500	200	1	0.000993783	12	6	NA	NA
DMRcontig06324:1172470	1.2E+07	11725600	900	1	0.000618606	26	2.888888889	NA	NA

DMRcontig06334:1174101	1.2E+07	11741300	283	1	0.000309471	2	0.706713781	NA	NA
DMRcontig06362:1178690	1.2E+07	11787500	600	4	3.68E-06	33	5.5	NA	NA
DMRcontig06367:1179571	1.2E+07	11796600	884	4	2.58E-06	37	4.185520362	NA	NA
DMRcontig06375:1180930	1.2E+07	11809700	400	2	2.70E-06	15	3.75	NA	NA
DMRcontig06377:1181282	1.2E+07	11813500	676	2	0.000463247	16	2.366863905	NA	NA
DMRcontig06381:1181926	1.2E+07	11819774	506	1	0.000318914	28	5.533596838	NA	NA
DMRcontig06422:1188411	1.2E+07	11884500	382	1	2.16E-05	7	1.832460733	NA	NA
DMRcontig06436:1190730	1.2E+07	11907600	300	1	2.63E-05	11	3.666666667	NA	NA
DMRcontig06440:1191470	1.2E+07	11915100	400	1	0.000307322	21	5.25	NA	NA
DMRcontig06441:1191640	1.2E+07	11917097	697	2	0.000920011	20	2.869440459	NA	NA
DMRcontig06442:1191830	1.2E+07	11918600	300	1	0.00014785	10	3.333333333	NA	NA
DMRcontig06478:1197811	1.2E+07	11978889	775	1	7.50E-05	71	9.161290323	NA	NA
DMRcontig06483:1198730	1.2E+07	11987488	188	1	7.90E-05	13	6.914893617	NA	NA
DMRcontig06494:1200583	1.2E+07	12006400	570	1	0.000961231	38	6.666666667	IscW_ISCW024073	Unknown
DMRcontig06504:1202186	1.2E+07	12022900	1036	3	2.53E-06	73	7.046332046	NA	NA
DMRcontig06508:1202923	1.2E+07	12029400	169	1	0.000403212	8	4.733727811	NA	NA
DMRcontig06512:1203550	1.2E+07	12035900	400	2	6.72E-06	10	2.5	NA	NA
DMRcontig06529:1206420	1.2E+07	12064700	500	1	0.00015027	22	4.4	NA	NA
DMRcontig06530:1206580	1.2E+07	12066200	400	1	0.000866321	23	5.75	NA	NA
DMRcontig06535:1207370	1.2E+07	12074270	570	1	0.000559491	10	1.754385965	NA	NA
DMRcontig06555:1210650	1.2E+07	12106900	400	1	0.000497188	19	4.75	NA	NA
DMRcontig06576:1214075	1.2E+07	12141200	444	2	4.68E-05	20	4.504504505	NA	NA
DMRcontig06587:1215860	1.2E+07	12158800	200	1	0.000458048	15	7.5	NA	NA
DMRcontig06588:1215990	1.2E+07	12160200	300	1	2.19E-05	10	3.333333333	NA	NA
DMRcontig06591:1216470	1.2E+07	12164900	200	1	0.000771824	12	6	NA	NA
DMRcontig06603:1218570	1.2E+07	12186090	390	1	9.42E-05	6	1.538461538	NA	NA
DMRcontig06626:1222170	1.2E+07	12222600	900	2	0.000534191	54	6	LOC105398638;hspa5	Protein Binding;Signaling
DMRcontig06645:1225325	1.2E+07	12253800	549	3	3.16E-05	41	7.468123862	NA	NA
DMRcontig06647:1225680	1.2E+07	12257300	500	5	1.08E-07	34	6.8	NA	NA
DMRcontig06648:1225870	1.2E+07	12259400	698	2	1.85E-05	28	4.011461318	NA	NA
DMRcontig06681:1231120	1.2E+07	12311494	294	1	0.000805124	4	1.360544218	NA	NA
DMRcontig06735:1239980	1.2E+07	12400300	493	2	0.000262667	22	4.462474645	NA	NA
DMRcontig06768:1245313	1.2E+07	12453600	467	1	8.16E-05	31	6.638115632	NA	NA
DMRcontig06777:1246780	1.2E+07	12468566	766	1	0.000187871	46	6.005221932	NA	NA
DMRcontig06823:1254460	1.3E+07	12545200	600	2	0.000438241	38	6.333333333	NA	NA
DMRcontig06845:1257960	1.3E+07	12579800	200	2	7.59E-05	18	9	NA	NA
DMRcontig06879:1263530	1.3E+07	12635673	373	3	4.75E-07	35	9.383378016	NA	NA
DMRcontig06886:1264640	1.3E+07	12647572	1172	3	8.17E-06	21	1.791808874	NA	NA
DMRcontig06887:1264890	1.3E+07	12649027	127	1	0.00024123	5	3.937007874	NA	NA
DMRcontig06916:1269580	1.3E+07	12696700	900	1	0.000911176	24	2.666666667	NA	NA
DMRcontig06961:1276850	1.3E+07	12769297	797	1	1.50E-05	21	2.634880803	NA	NA
DMRcontig06981:1280180	1.3E+07	12802437	637	5	1.63E-05	27	4.238618524	NA	NA
DMRcontig06984:1280670	1.3E+07	12807000	300	2	0.000480977	3	1	NA	NA
DMRcontig07016:1286010	1.3E+07	12860500	400	1	0.000245701	24	6	NA	NA
DMRcontig07017:1286230	1.3E+07	12862947	647	1	5.51E-05	27	4.173106646	NA	NA
DMRcontig07035:1289150	1.3E+07	12891800	300	1	9.62E-05	5	1.666666667	NA	NA
DMRcontig07061:1293310	1.3E+07	12933400	300	1	2.67E-05	6	2	UPF3B	Development
DMRcontig07068:1294520	1.3E+07	12945500	300	1	1.25E-08	8	2.666666667	NA	NA
DMRcontig07093:1298600	1.3E+07	12986300	300	1	0.000127826	12	4	NA	NA
DMRcontig07106:1300742	1.3E+07	13008000	579	2	0.000209504	24	4.14507772	NA	NA
DMRcontig07108:1301080	1.3E+07	13011693	893	1	0.00028787	20	2.239641657	NA	NA
DMRcontig07109:1301300	1.3E+07	13013314	314	1	6.16E-05	21	6.687898089	NA	NA
DMRcontig07114:1302090	1.3E+07	13021900	1000	1	0.000307337	46	4.6	NA	NA
DMRcontig07131:1304870	1.3E+07	13049000	300	2	8.23E-07	19	6.333333333	NA	NA
DMRcontig07161:1309770	1.3E+07	13098154	454	1	0.000201525	9	1.982378855	NA	NA
DMRcontig07170:1311140	1.3E+07	13111700	300	1	0.000132697	17	5.666666667	NA	NA
DMRcontig07187:1313860	1.3E+07	13139400	800	4	1.02E-05	31	3.875	NA	NA
DMRcontig07190:1314440	1.3E+07	13144600	200	1	0.000640363	1	0.5	TEKT4	Cytoskeleton
DMRcontig07203:1316486	1.3E+07	13165292	432	2	0.000647478	16	3.703703704	NA	NA
DMRcontig07244:1322970	1.3E+07	13230000	300	2	4.09E-10	18	6	NA	NA
DMRcontig07245:1323130	1.3E+07	13231500	200	1	0.000621158	5	2.5	NA	NA
DMRcontig07249:1323773	1.3E+07	13238100	363	1	0.000884208	23	6.336088154	NA	NA
DMRcontig07269:1327080	1.3E+07	13271200	400	2	0.000401489	33	8.25	NA	NA
DMRcontig07275:1328070	1.3E+07	13281200	500	1	1.43E-05	37	7.4	NA	NA
DMRcontig07328:1336390	1.3E+07	13364600	700	1	3.85E-05	41	5.857142857	NA	NA
DMRcontig07343:1338730	1.3E+07	13387598	298	1	0.000261006	17	5.704697987	NA	NA
DMRcontig07344:1338860	1.3E+07	13389300	700	2	0.000103572	49	7	NA	NA
DMRcontig07350:1339866	1.3E+07	13399121	454	1	0.000102051	19	4.185022026	NA	NA
DMRcontig07397:1347432	1.3E+07	13474700	375	2	7.92E-05	26	6.933333333	NA	NA
DMRcontig07417:1350710	1.4E+07	13507300	200	1	0.000140794	9	4.5	NA	NA
DMRcontig07468:1358719	1.4E+07	13587800	610	2	2.70E-05	36	5.901639344	NA	NA
DMRcontig07476:1359976	1.4E+07	13600488	725	2	2.35E-05	20	2.75862069	PODANSg7319;PODANSg7114;TBLA0F00780;TBLA0B09170;PTSG_10201;PTSG_05049;Gm31106;ncl;LOC100846034;LOC100827569	Unknown;Epigenetic

DMRcontig07485:1361320	1.4E+07	13613400	200	1	0.000448504	12	6	NA	NA
DMRcontig07501:1363933	1.4E+07	13640300	967	1	0.000529951	30	3.10237849	NA	NA
DMRcontig07544:1370914	1.4E+07	13709600	456	3	2.18E-07	21	4.605263158	NA	NA
DMRcontig07553:1372320	1.4E+07	13723500	300	2	0.000428882	12	4	NA	NA
DMRcontig07575:1375912	1.4E+07	13759300	177	2	4.25E-06	3	1.694915254	NA	NA
DMRcontig07589:1378190	1.4E+07	13782900	1000	1	0.000247193	52	5.2	NA	NA
DMRcontig07598:1379700	1.4E+07	13797700	700	1	0.000429285	45	6.428571429	MNS1	Development
DMRcontig07624:1384040	1.4E+07	13840600	200	1	0.000880312	1	0.5	NA	NA
DMRcontig07643:1386950	1.4E+07	13869878	378	2	5.80E-05	13	3.439153439	NA	NA
DMRcontig07645:1387300	1.4E+07	13873300	300	1	0.000200699	15	5	HELRODRAFT_187486;Pka-C1	Unknown;Signaling
DMRcontig07651:1388253	1.4E+07	13883000	464	1	0.000106377	17	3.663793103	NA	NA
DMRcontig07656:1389040	1.4E+07	13891200	800	2	0.000268403	38	4.75	NA	NA
DMRcontig07667:1390880	1.4E+07	13909574	774	1	0.000664159	36	4.651162791	NA	NA
DMRcontig07679:1393010	1.4E+07	13930745	645	4	6.37E-05	39	6.046511628	NA	NA
DMRcontig07687:1394352	1.4E+07	13943700	172	1	0.000128551	2	1.162790698	NA	NA
DMRcontig07711:1398250	1.4E+07	13982988	488	2	0.000202335	4	0.819672131	NA	NA
DMRcontig07715:1399010	1.4E+07	13990400	300	1	0.000999471	11	3.666666667	NA	NA
DMRcontig07723:1400200	1.4E+07	14002172	172	2	1.05E-05	5	2.906976744	NA	NA
DMRcontig07733:1401810	1.4E+07	14018300	200	1	0.000239958	12	6	NA	NA
DMRcontig07757:1405810	1.4E+07	14058500	400	1	0.000550064	23	5.75	NA	NA
DMRcontig07779:1409371	1.4E+07	14094500	788	1	0.000924577	23	2.918781726	NA	NA
DMRcontig07786:1410530	1.4E+07	14105500	200	1	0.000155329	7	3.5	NA	NA
DMRcontig07789:1411009	1.4E+07	14110400	309	1	0.000359585	14	4.530744337	NA	NA
DMRcontig07811:1414622	1.4E+07	14146600	375	1	4.37E-05	16	4.266666667	NA	NA
DMRcontig07818:1415770	1.4E+07	14157900	200	1	0.000612313	5	2.5	NA	NA
DMRcontig07825:1416930	1.4E+07	14169900	600	3	3.61E-05	12	2	COX1;ND3	Metabolism
DMRcontig07828:1417440	1.4E+07	14175000	600	1	0.000819594	22	3.666666667	LOC105392625	Transport
DMRcontig07840:1419351	1.4E+07	14194200	684	2	1.11E-06	14	2.046783626	LOC103241789;trnan-guu;LOC109086969	Protease
DMRcontig07861:1422790	1.4E+07	14228100	200	1	0.000313703	4	2	NA	NA
DMRcontig07896:1428390	1.4E+07	14284400	500	1	0.000482635	41	8.2	RPL15	Translation
DMRcontig07897:1428560	1.4E+07	14286500	900	1	4.68E-05	49	5.444444444	LOC100380865	Metabolism
DMRcontig07908:1430370	1.4E+07	14304213	513	2	2.73E-06	33	6.432748538	NA	NA
DMRcontig07913:1431110	1.4E+07	14311300	200	1	0.000243484	9	4.5	NA	NA
DMRcontig07922:1432500	1.4E+07	14325200	200	1	0.000780421	13	6.5	NA	NA
DMRcontig07932:1433983	1.4E+07	14340400	571	2	9.89E-05	18	3.152364273	NA	NA
DMRcontig07938:1434970	1.4E+07	14350200	500	2	4.59E-06	34	6.8	NA	NA
DMRcontig07945:1435975	1.4E+07	14359900	151	1	0.000723751	7	4.635761589	NA	NA
DMRcontig07953:1437460	1.4E+07	14374900	300	1	0.000861775	10	3.333333333	NA	NA
DMRcontig07962:1439000	1.4E+07	14390200	200	2	8.98E-06	9	4.5	NA	NA
DMRcontig07967:1439850	1.4E+07	14398700	200	1	0.000994871	13	6.5	NA	NA
DMRcontig07970:1440330	1.4E+07	14403575	275	1	0.000318242	4	1.454545455	NA	NA
DMRcontig08031:1450152	1.5E+07	14502021	498	1	5.63E-06	31	6.224899598	NA	NA
DMRcontig08060:1454850	1.5E+07	14548700	200	1	0.000911458	14	7	MNEG_4574	Unknown
DMRcontig08067:1456020	1.5E+07	14560700	500	2	2.41E-05	30	6	NA	NA
DMRcontig08095:1460430	1.5E+07	14604700	400	1	0.000105206	10	2.5	NA	NA
DMRcontig08096:1460592	1.5E+07	14606500	581	1	0.000440621	19	3.270223752	NA	NA
DMRcontig08098:1460910	1.5E+07	14609800	700	2	5.23E-06	29	4.142857143	NA	NA
DMRcontig08103:1461690	1.5E+07	14617100	200	1	5.13E-05	8	4	NA	NA
DMRcontig08123:1464780	1.5E+07	14648000	200	1	0.000631463	12	6	NA	NA
DMRcontig08155:1469820	1.5E+07	14698400	200	1	0.000693089	1	0.5	NA	NA
DMRcontig08166:1471540	1.5E+07	14715973	573	1	0.000827444	29	5.061082024	NA	NA
DMRcontig08168:1471950	1.5E+07	14719700	200	1	0.000516205	5	2.5	NA	NA
DMRcontig08171:1472510	1.5E+07	14726191	1091	1	9.64E-05	63	5.77451879	NA	NA
DMRcontig08191:1475760	1.5E+07	14758000	400	2	6.02E-06	16	4	NA	NA
DMRcontig08217:1479980	1.5E+07	14800000	200	1	0.000266669	7	3.5	NA	NA
DMRcontig08222:1480850	1.5E+07	14808957	457	2	1.09E-05	17	3.719912473	NA	NA
DMRcontig08247:1484720	1.5E+07	14847692	492	3	6.27E-05	30	6.097560976	NA	NA
DMRcontig08259:1486680	1.5E+07	14867200	400	1	0.00022145	19	4.75	NA	NA
DMRcontig08276:1489470	1.5E+07	14894900	200	1	0.000563709	14	7	NA	NA
DMRcontig08308:1494640	1.5E+07	14946700	292	2	0.000173048	8	2.739726027	NA	NA
DMRcontig08317:1496170	1.5E+07	14962000	300	1	0.000401943	17	5.666666667	LOC106569430;VCP	Unknown;Cytoskeleton
DMRcontig08341:1500080	1.5E+07	15001000	200	1	0.000374135	6	3	NA	NA
DMRcontig08346:1500957	1.5E+07	15009900	328	1	0.000394937	7	2.134146341	NA	NA
DMRcontig08360:1503140	1.5E+07	15031600	199	2	0.000215825	8	4.020100503	NA	NA
DMRcontig08374:1505430	1.5E+07	15054700	400	1	5.01E-05	20	5	LOC102223325	Unknown
DMRcontig08377:1505859	1.5E+07	15058800	206	1	0.000741797	4	1.941747573	NA	NA
DMRcontig08379:1506250	1.5E+07	15062800	300	1	0.000469413	22	7.333333333	NA	NA
DMRcontig08449:1517680	1.5E+07	15176969	169	1	0.000478974	7	4.142011834	NA	NA
DMRcontig08472:1521230	1.5E+07	15212700	400	1	0.000427288	5	1.25	NA	NA
DMRcontig08475:1521670	1.5E+07	15217000	300	1	1.16E-05	10	3.333333333	PHSY_005264;PPP1CA	Unknown;Signaling
DMRcontig08488:1523700	1.5E+07	15237500	500	1	0.000334602	23	4.6	NA	NA
DMRcontig08517:1528160	1.5E+07	15281800	200	1	0.00058849	1	0.5	NA	NA
DMRcontig08522:1528930	1.5E+07	15289500	200	1	0.000348146	15	7.5	NA	NA
DMRcontig08523:1529090	1.5E+07	15291200	300	1	0.000647349	5	1.666666667	NA	NA
DMRcontig08534:1530950	1.5E+07	15309771	271	1	0.000225903	16	5.904059041	NA	NA

DMRcontig08559:1534835	1.5E+07	15348897	545	2	4.71E-07	18	3.302752294	NA	NA
DMRcontig08571:1536890	1.5E+07	15369514	614	1	5.40E-05	38	6.188925081	NA	NA
DMRcontig08582:1538620	1.5E+07	15387026	821	2	9.16E-05	24	2.923264312	NA	NA
DMRcontig08583:1538820	1.5E+07	15388500	300	2	6.66E-06	12	4	NA	NA
DMRcontig08588:1539590	1.5E+07	15397000	1100	2	0.00037962	28	2.545454545	NA	NA
DMRcontig08594:1540610	1.5E+07	15406300	200	1	0.000177868	9	4.5	NA	NA
DMRcontig08616:1544130	1.5E+07	15441800	500	1	1.81E-05	7	1.4	NA	NA
DMRcontig08643:1548496	1.5E+07	15485600	636	1	0.000235162	34	5.34591195	NA	NA
DMRcontig08729:1562150	1.6E+07	15622000	500	3	5.56E-06	21	4.2	NA	NA
DMRcontig08783:1570740	1.6E+07	15708500	1100	2	0.000441571	28	2.545454545	NA	NA
DMRcontig08794:1572540	1.6E+07	15725785	385	1	0.000633122	13	3.376623377	NA	NA
DMRcontig08800:1573429	1.6E+07	15734800	505	4	2.92E-06	41	8.118811881	NA	NA
DMRcontig08802:1573780	1.6E+07	15738026	226	2	1.83E-06	13	5.752212389	LOC101857837;AP1B1	Development
DMRcontig08896:1588440	1.6E+07	15884900	500	2	1.31E-05	13	2.6	SPRG_12594	Cytoskeleton
DMRcontig08913:1591060	1.6E+07	15911279	679	2	0.000145976	15	2.209131075	NA	NA
DMRcontig08917:1591750	1.6E+07	15917800	300	2	0.00066583	12	4	NA	NA
DMRcontig08923:1592740	1.6E+07	15927700	300	1	1.02E-05	14	4.666666667	NA	NA
DMRcontig08967:1599780	1.6E+07	15998000	200	2	4.84E-06	15	7.5	NA	NA
DMRcontig09001:1604968	1.6E+07	16050200	516	1	9.02E-06	12	2.325581395	NA	NA
DMRcontig09019:1607740	1.6E+07	16077800	400	1	0.000102352	8	2	NA	NA
DMRcontig09036:1610620	1.6E+07	16106700	500	1	0.000295266	4	0.8	NA	NA
DMRcontig09055:1613550	1.6E+07	16135817	317	3	8.00E-06	4	1.261829653	NA	NA
DMRcontig09058:1613984	1.6E+07	16140348	505	1	5.33E-06	20	3.96039604	cttn	Cytoskeleton
DMRcontig09075:1616490	1.6E+07	16165400	493	2	2.83E-05	12	2.434077079	NA	NA
DMRcontig09080:1617310	1.6E+07	16173400	300	1	0.000672603	12	4	NA	NA
DMRcontig09086:1618240	1.6E+07	16182664	264	1	8.73E-05	14	5.303030303	NA	NA
DMRcontig09087:1618400	1.6E+07	16184368	368	1	0.000417108	6	1.630434783	NA	NA
DMRcontig09091:1618996	1.6E+07	16190500	539	1	0.000177338	39	7.235621521	NA	NA
DMRcontig09112:1622560	1.6E+07	16226100	500	1	0.000373367	17	3.4	NA	NA
DMRcontig09116:1623200	1.6E+07	16232455	455	1	0.000474619	27	5.934065934	NA	NA
DMRcontig09129:1625240	1.6E+07	16252600	200	1	0.000481439	13	6.5	CpipJ_CPIJ017745;FAXDC2	Metabolism
DMRcontig09148:1628392	1.6E+07	16284100	178	1	0.000866321	1	0.561797753	NA	NA
DMRcontig09150:1628710	1.6E+07	16287700	600	3	1.62E-05	43	7.166666667	NA	NA
DMRcontig09155:1629560	1.6E+07	16296053	453	2	9.53E-06	25	5.518763797	NA	NA
DMRcontig09183:1633970	1.6E+07	16339846	146	1	0.000977398	4	2.739726027	NA	NA
DMRcontig09228:1640970	1.6E+07	16410200	500	2	1.77E-05	21	4.2	NA	NA
DMRcontig09241:1642950	1.6E+07	16429700	200	1	0.00023587	3	1.5	NA	NA
DMRcontig09246:1643630	1.6E+07	16436700	400	1	0.000288604	31	7.75	NA	NA
DMRcontig09247:1643780	1.6E+07	16438400	600	2	5.73E-06	29	4.833333333	NA	NA
DMRcontig09249:1644120	1.6E+07	16441477	277	2	0.000220383	11	3.971119134	NA	NA
DMRcontig09266:1646730	1.6E+07	16467586	286	1	0.000145231	2	0.699300699	NA	NA
DMRcontig09268:1647040	1.6E+07	16470624	224	2	0.000150357	10	4.464285714	NA	NA
DMRcontig09282:1649230	1.6E+07	16492600	300	1	3.31E-05	14	4.666666667	NA	NA
DMRcontig09305:1652910	1.7E+07	16529599	499	2	6.27E-05	11	2.204408818	NA	NA
DMRcontig09319:1655020	1.7E+07	16550538	338	2	4.09E-06	20	5.917159763	NA	NA
DMRcontig09336:1657720	1.7E+07	16577800	600	1	0.000737626	27	4.5	KAT8	Unknown
DMRcontig09342:1658711	1.7E+07	16587500	387	1	0.000279197	6	1.550387597	NA	NA
DMRcontig09351:1660240	1.7E+07	16602700	300	1	0.000102051	8	2.666666667	NA	NA
DMRcontig09377:1664450	1.7E+07	16644900	400	2	8.22E-05	8	2	NA	NA
DMRcontig09444:1674848	1.7E+07	16749700	1217	4	5.00E-05	31	2.547247329	NA	NA
DMRcontig09481:1680690	1.7E+07	16807100	200	1	0.00098484	3	1.5	NA	NA
DMRcontig09487:1681610	1.7E+07	16816300	200	1	0.000285515	8	4	NA	NA
DMRcontig09502:1683798	1.7E+07	16838500	515	2	0.000246686	13	2.524271845	NA	NA
DMRcontig09547:1690966	1.7E+07	16910245	577	3	2.21E-06	28	4.852686308	NA	NA
DMRcontig09571:1694710	1.7E+07	16947300	200	1	0.000868042	5	2.5	NA	NA
DMRcontig09576:1695491	1.7E+07	16955479	566	4	3.89E-06	25	4.416961131	NA	NA
DMRcontig09584:1696770	1.7E+07	16968100	400	2	0.00023677	26	6.5	NA	NA
DMRcontig09618:1702270	1.7E+07	17023000	300	1	0.00047017	18	6	NA	NA
DMRcontig09622:1702880	1.7E+07	17029000	200	1	0.000221726	2	1	NA	NA
DMRcontig09649:1707096	1.7E+07	17071400	436	3	4.71E-07	21	4.816513761	NA	NA
DMRcontig09660:1708770	1.7E+07	17087900	200	1	0.000142804	9	4.5	LOC106070317	Unknown
DMRcontig09665:1709584	1.7E+07	17096200	360	3	0.000115981	36	10	NA	NA
DMRcontig09685:1712700	1.7E+07	17127500	500	1	0.000312616	17	3.4	NA	NA
DMRcontig09686:1712851	1.7E+07	17128900	387	1	2.88E-05	7	1.80878553	NA	NA
DMRcontig09698:1714911	1.7E+07	17149575	464	1	0.000708238	33	7.112068966	NA	NA
DMRcontig09702:1715590	1.7E+07	17156300	400	1	0.000567118	30	7.5	NA	NA
DMRcontig09717:1717710	1.7E+07	17177600	500	2	6.07E-05	6	1.2	NA	NA
DMRcontig09719:1718020	1.7E+07	17180400	200	1	0.000509967	5	2.5	NA	NA
DMRcontig09723:1718673	1.7E+07	17187100	369	2	3.99E-05	12	3.25203252	NA	NA
DMRcontig09741:1721566	1.7E+07	17216000	341	1	0.00098254	11	3.225806452	NA	NA
DMRcontig09742:1721730	1.7E+07	17217900	600	1	0.000522951	31	5.166666667	NA	NA
DMRcontig09743:1721920	1.7E+07	17219400	200	1	0.000857075	7	3.5	NA	NA
DMRcontig09751:1723210	1.7E+07	17232681	581	1	0.000576562	34	5.851979346	NA	NA
DMRcontig09776:1727090	1.7E+07	17271300	400	2	7.00E-06	12	3	NA	NA
DMRcontig09780:1727750	1.7E+07	17277800	300	1	0.000148684	12	4	NA	NA
DMRcontig09790:1729430	1.7E+07	17294600	300	1	0.00017097	12	4	NA	NA

DMRcontig09810:1732470	1.7E+07	17325200	495	1	0.000563503	19	3.838383838	LOC105376234;Ankfn1	Cytoskeleton;Unknown
DMRcontig09816:1733475	1.7E+07	17335200	449	2	1.48E-05	33	7.349665924	NA	NA
DMRcontig09860:1740285	1.7E+07	17403288	430	3	9.51E-05	18	4.186046512	NA	NA
DMRcontig09865:1741010	1.7E+07	17410564	464	1	2.29E-05	21	4.525862069	LOC103363483;LOC105912044	Development
DMRcontig09893:1745170	1.7E+07	17451900	200	1	0.000572938	8	4	NA	NA
DMRcontig09894:1745310	1.7E+07	17453300	200	1	0.000280636	7	3.5	NA	NA
DMRcontig09899:1746024	1.7E+07	17460730	483	3	3.92E-05	9	1.863354037	NA	NA
DMRcontig09906:1747050	1.7E+07	17470800	300	1	5.75E-07	15	5	NA	NA
DMRcontig09937:1751727	1.8E+07	17517700	428	1	0.000106834	11	2.570093458	NA	NA
DMRcontig09960:1755321	1.8E+07	17553500	288	1	4.09E-06	8	2.777777778	NA	NA
DMRcontig09962:1755610	1.8E+07	17556372	272	1	0.000178017	4	1.470588235	NA	NA
DMRcontig09976:1757910	1.8E+07	17579300	200	1	0.000711416	6	3	NA	NA
DMRcontig10006:1762590	1.8E+07	17626400	500	1	9.28E-05	14	2.8	NA	NA
DMRcontig10009:1763091	1.8E+07	17631465	555	1	0.000229541	30	5.405405405	NA	NA
DMRcontig10024:1765435	1.8E+07	17655000	647	2	3.16E-06	33	5.100463679	NA	NA
DMRcontig10093:1776150	1.8E+07	17762290	790	2	3.61E-05	11	1.392405063	NA	NA
DMRcontig10095:1776460	1.8E+07	17764900	300	1	0.000845647	13	4.333333333	LOC106154174	Signaling
DMRcontig10144:1784122	1.8E+07	17841758	538	2	5.85E-05	32	5.94795539	NA	NA
DMRcontig10172:1788540	1.8E+07	17885600	200	1	0.000854045	11	5.5	NA	NA
DMRcontig10178:1789440	1.8E+07	17894800	400	1	0.000192836	15	3.75	NA	NA
DMRcontig10188:1790990	1.8E+07	17910400	500	1	8.29E-06	21	4.2	NA	NA
DMRcontig10213:1794780	1.8E+07	17948800	994	2	1.68E-07	29	2.91750503	NA	NA
DMRcontig10218:1795572	1.8E+07	17956100	375	1	0.000160152	25	6.666666667	NA	NA
DMRcontig10221:1796040	1.8E+07	17960600	200	1	0.000220086	4	2	NA	NA
DMRcontig10235:1798200	1.8E+07	17982100	100	1	0.000510592	7	7	NA	NA
DMRcontig10237:1798540	1.8E+07	17985586	186	1	0.000311592	0	0	NA	NA
DMRcontig10246:1799950	1.8E+07	17999681	181	1	0.000364032	9	4.972375691	NA	NA
DMRcontig10285:1805783	1.8E+07	18058100	271	1	0.000684939	13	4.79704797	LOC101846510	Transcription
DMRcontig10286:1805960	1.8E+07	18059878	278	2	4.12E-05	10	3.597122302	NA	NA
DMRcontig10290:1806534	1.8E+07	18065900	552	2	0.000262154	31	5.615942029	NA	NA
DMRcontig10293:1807080	1.8E+07	18071727	927	6	3.48E-07	30	3.236245955	NA	NA
DMRcontig10326:1811980	1.8E+07	18119900	100	1	0.000177811	6	6	NA	NA
DMRcontig10328:1812290	1.8E+07	18123100	200	1	0.000566908	2	1	NA	NA
DMRcontig10362:1817570	1.8E+07	18176389	689	1	0.000157361	15	2.177068215	NA	NA
DMRcontig10370:1818746	1.8E+07	18187800	334	2	6.54E-08	16	4.790419162	LOC103035785	Signaling
DMRcontig10422:1826650	1.8E+07	18266900	400	1	0.000585124	26	6.5	BRAFLDRAFT_283521;psmd14	Unknown;Protease
DMRcontig10428:1827562	1.8E+07	18276000	376	1	0.000811757	24	6.382978723	NA	NA
DMRcontig10430:1827854	1.8E+07	18278800	253	2	1.80E-06	18	7.114624506	NA	NA
DMRcontig10452:1831222	1.8E+07	18312921	695	1	0.000224772	32	4.604316547	LOC106577905;LOC103036640	Translation
DMRcontig10480:1835538	1.8E+07	18355800	413	2	0.00027511	40	9.685230024	NA	NA
DMRcontig10548:1845840	1.8E+07	18458600	200	1	0.000928637	2	1	NA	NA
DMRcontig10551:1846310	1.8E+07	18463275	175	1	0.000153244	5	2.857142857	NA	NA
DMRcontig10571:1849380	1.8E+07	18493900	100	1	0.000562601	4	4	NA	NA
DMRcontig10572:1849561	1.8E+07	18495900	288	1	0.000313703	18	6.25	NA	NA
DMRcontig10575:1850073	1.9E+07	18501118	384	1	0.000360472	19	4.947916667	NA	NA
DMRcontig10630:1858590	1.9E+07	18586300	400	1	9.08E-05	5	1.25	NA	NA
DMRcontig10650:1861780	1.9E+07	18618018	218	1	0.000392899	5	2.293577982	NA	NA
DMRcontig10681:1866411	1.9E+07	18664600	487	2	0.000250919	4	0.821355236	NA	NA
DMRcontig10718:1872200	1.9E+07	18722600	600	1	0.000441813	40	6.666666667	BMP5	Growth Factors & Cytokines
DMRcontig10725:1873320	1.9E+07	18734000	800	8	3.21E-06	44	5.5	NA	NA
DMRcontig10727:1873680	1.9E+07	18737000	200	1	0.000725938	4	2	NA	NA
DMRcontig10731:1874282	1.9E+07	18743249	424	3	1.26E-05	36	8.490566038	NA	NA
DMRcontig10773:1880720	1.9E+07	18808178	978	2	0.00037805	31	3.169734151	NA	NA
DMRcontig10782:1882125	1.9E+07	18821600	345	2	1.94E-05	5	1.449275362	NA	NA
DMRcontig10848:1892131	1.9E+07	18922000	685	2	0.000107412	20	2.919708029	NA	NA
DMRcontig10860:1894000	1.9E+07	18940200	200	1	0.000184629	18	9	LOC104952122	Signaling
DMRcontig10869:1895330	1.9E+07	18953700	400	1	0.000106956	27	6.75	NA	NA
DMRcontig10874:1896070	1.9E+07	18961400	700	2	0.000315859	34	4.857142857	NA	NA
DMRcontig10893:1898950	1.9E+07	18989800	300	1	3.33E-05	7	2.333333333	NA	NA
DMRcontig10894:1899091	1.9E+07	18991100	186	1	3.74E-05	20	10.75268817	NA	NA
DMRcontig10917:1902550	1.9E+07	19025800	300	1	0.00094086	13	4.333333333	NA	NA
DMRcontig10920:1903020	1.9E+07	19030577	377	1	0.000669689	11	2.917771883	NA	NA
DMRcontig10931:1904740	1.9E+07	19047700	300	3	9.04E-07	2	0.666666667	NA	NA
DMRcontig10987:1913220	1.9E+07	19133000	799	3	0.000162539	8	1.001251564	NA	NA
DMRcontig11000:1915210	1.9E+07	19152200	100	1	0.000845317	1	1	NA	NA
DMRcontig11012:1917070	1.9E+07	19171100	400	2	0.000285869	15	3.75	NA	NA
DMRcontig11019:1918101	1.9E+07	19181500	484	1	0.00016885	17	3.512396694	NA	NA
DMRcontig11020:1918280	1.9E+07	19183000	200	1	0.000284913	16	8	NA	NA
DMRcontig11060:1924331	1.9E+07	19243700	383	2	0.000111015	32	8.355091384	NA	NA
DMRcontig11064:1924950	1.9E+07	19250026	526	2	3.92E-07	14	2.661596958	NA	NA
DMRcontig11098:1930010	1.9E+07	19300568	468	2	7.35E-07	23	4.914529915	NA	NA
DMRcontig11173:1941220	1.9E+07	19412685	485	1	0.00018523	11	2.268041237	NA	NA
DMRcontig11180:1942273	1.9E+07	19423300	562	2	7.40E-06	23	4.09252669	NA	NA
DMRcontig11199:1945260	1.9E+07	19452854	254	1	0.00012682	13	5.118110236	NA	NA
DMRcontig11227:1949600	1.9E+07	19496100	100	1	9.20E-05	1	1	NA	NA
DMRcontig11243:1951930	2E+07	19519500	200	1	0.000769993	6	3	NA	NA

DMRcontig11248:1952784	2E+07	19528382	543	2	4.27E-05	6	1.104972376	NA	NA
DMRcontig11255:1953900	2E+07	19539200	200	1	0.000774926	16	8	NA	NA
DMRcontig11326:1964530	2E+07	19645600	300	1	0.000421492	19	6.333333333	LOC101481297	Epigenetic
DMRcontig11351:1968288	2E+07	19683500	615	1	9.70E-05	25	4.06504065	NA	NA
DMRcontig11361:1969860	2E+07	19699071	471	1	0.000525042	13	2.760084926	NA	NA
DMRcontig11422:1978959	2E+07	19789700	105	1	0.000729815	7	6.666666667	NA	NA
DMRcontig11455:1984000	2E+07	19840435	435	2	1.81E-05	23	5.287356322	NA	NA
DMRcontig11459:1984581	2E+07	19846059	243	1	1.48E-06	5	2.057613169	NA	NA
DMRcontig11475:1986960	2E+07	19870194	594	4	0.000289435	36	6.060606061	LOC106582085;fam46c	Unknown
DMRcontig11491:1989480	2E+07	19895074	274	1	0.000734055	9	3.284671533	NA	NA
DMRcontig11501:1991008	2E+07	19910200	120	1	0.000140585	9	7.5	NA	NA
DMRcontig11519:1993809	2E+07	19938342	250	1	0.000969043	4	1.6	NA	NA
DMRcontig11521:1994090	2E+07	19941199	299	1	4.57E-05	16	5.351170569	NA	NA
DMRcontig11547:1997915	2E+07	19979600	445	2	4.79E-06	16	3.595505618	NA	NA
DMRcontig11590:2004240	2E+07	20042577	177	1	0.0004249	14	7.90960452	NA	NA
DMRcontig11622:2008832	2E+07	20088831	503	2	0.000348723	17	3.37972167	NA	NA
DMRcontig11642:2011820	2E+07	20118672	472	2	9.18E-05	13	2.754237288	NA	NA
DMRcontig11658:2014250	2E+07	20142680	180	1	0.000580361	6	3.333333333	NA	NA
DMRcontig11695:2019860	2E+07	20199100	500	5	3.72E-12	12	2.4	NA	NA
DMRcontig11767:2030723	2E+07	20307700	471	1	0.000801085	11	2.335456476	NA	NA
DMRcontig11819:2038719	2E+07	20387500	307	1	4.41E-06	8	2.605863192	NA	NA
DMRcontig11847:2043030	2E+07	20430500	200	2	6.33E-05	8	4	NA	NA
DMRcontig11865:2045770	2E+07	20457900	200	1	0.000540231	13	6.5	NA	NA
DMRcontig11969:2061240	2.1E+07	20612741	341	1	0.000889827	8	2.346041056	NA	NA
DMRcontig11994:2065010	2.1E+07	20650500	400	1	0.000940641	22	5.5	NA	NA
DMRcontig12018:2068520	2.1E+07	20685500	300	1	0.000720462	16	5.333333333	NA	NA
DMRcontig12053:2073970	2.1E+07	20740300	600	4	9.06E-06	19	3.166666667	NA	NA
DMRcontig12118:2083830	2.1E+07	20838700	400	1	0.000846265	22	5.5	NA	NA
DMRcontig12119:2083977	2.1E+07	20840300	530	2	1.97E-05	45	8.490566038	NA	NA
DMRcontig12120:2084144	2.1E+07	20841700	258	1	6.22E-05	0	0	NA	NA
DMRcontig12144:2087765	2.1E+07	20877900	249	2	0.000489433	21	8.43373494	NA	NA
DMRcontig12149:2088530	2.1E+07	20885500	200	1	0.000706805	6	3	NA	NA
DMRcontig12154:2089230	2.1E+07	20892500	200	1	0.000702925	14	7	LOC101849038	Unknown
DMRcontig12166:2091007	2.1E+07	20910300	229	2	0.00037489	14	6.113537118	NA	NA
DMRcontig12167:2091180	2.1E+07	20912044	244	1	0.000442717	24	9.836065574	NA	NA
DMRcontig12185:2093780	2.1E+07	20938132	332	2	6.71E-05	9	2.710843373	NA	NA
DMRcontig12259:2105080	2.1E+07	21051086	286	1	7.65E-05	11	3.846153846	NA	NA
DMRcontig12272:2107000	2.1E+07	21070200	200	1	0.000726779	4	2	NA	NA
DMRcontig12293:2110120	2.1E+07	21101500	300	1	0.000259684	7	2.333333333	NA	NA
DMRcontig12315:2113450	2.1E+07	21134789	289	2	0.000103618	11	3.806228374	NA	NA
DMRcontig12344:2118000	2.1E+07	21180365	359	3	1.82E-06	13	3.621169916	NA	NA
DMRcontig12357:2119990	2.1E+07	21200043	143	1	0.000476556	10	6.993006993	NA	NA
DMRcontig12370:2121900	2.1E+07	21219300	300	1	0.000688633	16	5.333333333	NA	NA
DMRcontig12376:2122840	2.1E+07	21229142	742	1	0.000567663	23	3.099730458	NA	NA
DMRcontig12387:2124570	2.1E+07	21245900	200	2	4.76E-05	1	0.5	NA	NA
DMRcontig12389:2124851	2.1E+07	21248900	390	2	8.43E-06	22	5.641025641	NA	NA
DMRcontig12426:2130314	2.1E+07	21303700	556	2	9.58E-05	12	2.158273381	NA	NA
DMRcontig12476:2137922	2.1E+07	21379600	375	1	0.000405004	8	2.133333333	NA	NA
DMRcontig12498:2141490	2.1E+07	21415104	204	1	0.000890577	8	3.921568627	NA	NA
DMRcontig12522:2144950	2.1E+07	21449600	100	1	0.000221726	1	1	NA	NA
DMRcontig12534:2146690	2.1E+07	21467300	400	1	0.000198566	10	2.5	NA	NA
DMRcontig12541:2147819	2.1E+07	21478400	205	1	0.0009164	8	3.902439024	NA	NA
DMRcontig12653:2164371	2.2E+07	21644184	466	2	1.07E-05	10	2.145922747	NA	NA
DMRcontig12667:2166420	2.2E+07	21664500	300	1	0.000701357	3	1	NA	NA
DMRcontig12714:2173340	2.2E+07	21733600	200	1	0.000483426	2	1	NA	NA
DMRcontig12723:2174670	2.2E+07	21747200	500	3	1.99E-05	23	4.6	NA	NA
DMRcontig12747:2178290	2.2E+07	21783072	172	1	0.000176124	8	4.651162791	NA	NA
DMRcontig12766:2181113	2.2E+07	21811700	570	3	1.77E-06	21	3.684210526	NA	NA
DMRcontig12794:2185220	2.2E+07	21852500	300	1	0.000288186	6	2	NA	NA
DMRcontig12809:2187580	2.2E+07	21875995	195	1	0.00062702	11	5.641025641	NA	NA
DMRcontig12813:2188110	2.2E+07	21881500	400	2	0.000596319	11	2.75	NA	NA
DMRcontig12845:2192780	2.2E+07	21928000	200	1	0.000473732	7	3.5	NA	NA
DMRcontig12932:2205610	2.2E+07	22056594	494	2	6.55E-05	16	3.238866397	NA	NA
DMRcontig12960:2209716	2.2E+07	22097376	210	1	4.86E-06	3	1.428571429	NA	NA
DMRcontig13020:2218520	2.2E+07	22185536	336	1	0.000401072	16	4.761904762	NA	NA
DMRcontig13123:2233800	2.2E+07	22338500	500	2	1.41E-05	15	3	NA	NA
DMRcontig13127:2234450	2.2E+07	22344700	194	1	0.000690707	10	5.154639175	NA	NA
DMRcontig13197:2244720	2.2E+07	22447500	300	1	0.000160274	15	5	LOC109089225;bnip3lb	Unknown
DMRcontig13215:2247260	2.2E+07	22473050	450	1	0.000339298	15	3.333333333	NA	NA
DMRcontig13272:2255651	2.3E+07	22557000	487	2	0.000325697	18	3.696098563	NA	NA
DMRcontig13276:2256270	2.3E+07	22563000	300	2	0.000631178	6	2	NA	NA
DMRcontig13288:2258103	2.3E+07	22581517	486	5	1.04E-08	25	5.144032922	NA	NA
DMRcontig13291:2258550	2.3E+07	22585800	300	2	8.58E-06	11	3.666666667	NA	NA
DMRcontig13301:2260070	2.3E+07	22601100	400	1	0.000619286	9	2.25	NA	NA
DMRcontig13316:2262284	2.3E+07	22623300	453	2	7.08E-05	18	3.973509934	NA	NA
DMRcontig13323:2263410	2.3E+07	22635137	1037	4	5.14E-07	57	5.496624879	NA	NA

DMRcontig13353:2267950	2.3E+07	22680100	600	2	0.000572781	21	3.5	NA	NA
DMRcontig13362:2269380	2.3E+07	22694400	600	2	4.10E-05	30	5	NA	NA
DMRcontig13534:2294800	2.3E+07	22948342	342	1	0.00014719	11	3.216374269	NA	NA
DMRcontig13586:2302564	2.3E+07	23026300	657	2	0.000115499	22	3.348554033	NA	NA
DMRcontig13607:2305860	2.3E+07	23059200	600	1	0.000599789	19	3.166666667	NA	NA
DMRcontig13624:2308420	2.3E+07	23084601	401	4	8.72E-07	19	4.738154613	NA	NA
DMRcontig13662:2314240	2.3E+07	23142800	400	1	0.000141572	13	3.25	NA	NA
DMRcontig13665:2314701	2.3E+07	23147500	482	2	0.000526505	25	5.186721992	NA	NA
DMRcontig13690:2318430	2.3E+07	23184577	277	2	0.000251685	19	6.859205776	NA	NA
DMRcontig13696:2319300	2.3E+07	23193100	100	1	0.000528339	0	0	NA	NA
DMRcontig13699:2319720	2.3E+07	23197600	400	2	4.72E-05	5	1.25	NA	NA
DMRcontig13754:2327735	2.3E+07	23277600	251	2	5.84E-05	17	6.772908367	NA	NA
DMRcontig13774:2330760	2.3E+07	23307900	300	2	0.000602861	22	7.333333333	NA	NA
DMRcontig13815:2337000	2.3E+07	23370288	288	1	0.000695384	15	5.208333333	NA	NA
DMRcontig13854:2342720	2.3E+07	23427400	200	1	0.000423803	4	2	NA	NA
DMRcontig13882:2346750	2.3E+07	23467700	200	1	0.00052587	8	4	NA	NA
DMRcontig13916:2351970	2.4E+07	23519900	200	1	0.000981093	6	3	NA	NA
DMRcontig13919:2352371	2.4E+07	23524374	656	1	4.20E-05	22	3.353658537	NA	NA
DMRcontig13952:2357340	2.4E+07	23573700	300	1	0.00039491	17	5.666666667	NA	NA
DMRcontig13992:2363300	2.4E+07	23633200	200	1	0.000772364	10	5	NA	NA
DMRcontig13997:2364050	2.4E+07	23640776	276	2	4.59E-05	10	3.623188406	NA	NA
DMRcontig14064:2373820	2.4E+07	23738400	197	2	6.13E-05	7	3.553299492	NA	NA
DMRcontig14095:2378440	2.4E+07	23784600	200	1	0.000795861	16	8	NA	NA
DMRcontig14121:2382205	2.4E+07	23822600	550	1	0.000608807	24	4.363636364	NA	NA
DMRcontig14129:2383460	2.4E+07	23835000	400	3	2.21E-05	17	4.25	LOC105398638	Signaling
DMRcontig14150:2386650	2.4E+07	23866624	124	1	0.000635609	8	6.451612903	NA	NA
DMRcontig14218:2396571	2.4E+07	23966091	376	3	1.01E-07	3	0.79787234	NA	NA
DMRcontig14240:2399793	2.4E+07	23998400	466	2	1.14E-06	33	7.081545064	NA	NA
DMRcontig14247:2400850	2.4E+07	24008700	200	1	0.00024204	7	3.5	NA	NA
DMRcontig14270:2404215	2.4E+07	24042400	243	1	0.000867954	9	3.703703704	NA	NA
DMRcontig14301:2408730	2.4E+07	24087700	398	2	0.000160844	16	4.020100503	NA	NA
DMRcontig14337:2413970	2.4E+07	24139900	200	1	0.000688019	9	4.5	NA	NA
DMRcontig14363:2417870	2.4E+07	24178843	143	1	0.000607087	0	0	NA	NA
DMRcontig14382:2420592	2.4E+07	24206350	428	5	1.67E-09	16	3.738317757	NA	NA
DMRcontig14393:2422300	2.4E+07	24223200	200	1	0.000539776	9	4.5	IscW_ISCW020686;suclg1	Metabolism
DMRcontig14395:2422585	2.4E+07	24226071	221	2	0.000215384	8	3.619909502	NA	NA
DMRcontig14411:2424960	2.4E+07	24249856	256	1	7.72E-05	20	7.8125	LOC101848057;IDH3B	Metabolism
DMRcontig14427:2427370	2.4E+07	24274078	378	2	4.13E-06	15	3.968253968	NA	NA
DMRcontig14496:2437550	2.4E+07	24375867	367	2	0.000510101	15	4.08719346	NA	NA
DMRcontig14508:2439370	2.4E+07	24394000	300	1	0.000352655	8	2.666666667	NA	NA
DMRcontig14588:2451130	2.5E+07	24511400	100	1	0.000686044	8	8	NA	NA
DMRcontig14592:2451750	2.5E+07	24517600	100	1	0.000473415	8	8	NA	NA
DMRcontig14608:2454022	2.5E+07	24540751	531	4	9.62E-06	73	13.74764595	NA	NA
DMRcontig14618:2455580	2.5E+07	24556070	270	3	1.91E-06	5	1.851851852	NA	NA
DMRcontig14627:2456940	2.5E+07	24570200	800	2	5.62E-06	38	4.75	NA	NA
DMRcontig14673:2463570	2.5E+07	24636054	354	1	0.000292748	16	4.519774011	ZMYND11	Transcription
DMRcontig14678:2464330	2.5E+07	24643600	300	1	0.000532547	8	2.666666667	NA	NA
DMRcontig14690:2466090	2.5E+07	24661533	633	1	7.83E-06	34	5.371248025	NA	NA
DMRcontig14746:2474310	2.5E+07	24743300	200	1	5.02E-05	7	3.5	IscW_ISCW001013	Unknown
DMRcontig14755:2475730	2.5E+07	24757800	493	2	7.71E-05	9	1.825557809	NA	NA
DMRcontig14763:2477064	2.5E+07	24770800	157	1	4.52E-06	6	3.821656051	NA	NA
DMRcontig14782:2479840	2.5E+07	24798600	200	1	0.000411787	11	5.5	NA	NA
DMRcontig14793:2481426	2.5E+07	24814858	595	3	7.86E-06	39	6.554621849	NA	NA
DMRcontig14799:2482350	2.5E+07	24823600	100	1	0.000428904	1	1	NA	NA
DMRcontig14806:2483390	2.5E+07	24834184	284	1	0.000716074	9	3.169014085	NA	NA
DMRcontig14821:2485601	2.5E+07	24856356	344	1	5.37E-05	5	1.453488372	NA	NA
DMRcontig14877:2493920	2.5E+07	24939300	100	1	0.000534818	2	2	NA	NA
DMRcontig14882:2494620	2.5E+07	24946437	237	1	0.000175832	3	1.265822785	NA	NA
DMRcontig14893:2496270	2.5E+07	24963200	500	2	0.000513016	21	4.2	NA	NA
DMRcontig14905:2498086	2.5E+07	24981054	186	1	4.99E-05	5	2.688172043	NA	NA
DMRcontig14915:2499530	2.5E+07	24995500	200	1	0.000363253	13	6.5	NA	NA
DMRcontig14918:2499970	2.5E+07	24999900	200	1	0.000983165	3	1.5	NA	NA
DMRcontig14936:2502670	2.5E+07	25027000	300	1	9.20E-05	16	5.333333333	NA	NA
DMRcontig14971:2507770	2.5E+07	25078000	300	2	0.000123569	4	1.333333333	NA	NA
DMRcontig14980:2509180	2.5E+07	25092173	373	3	0.000131882	20	5.361930295	NA	NA
DMRcontig14999:2511850	2.5E+07	25118700	200	1	0.000830121	10	5	NA	NA
DMRcontig15030:2516280	2.5E+07	25163400	600	2	3.74E-05	37	6.166666667	NA	NA
DMRcontig15052:2519461	2.5E+07	25194774	164	1	0.000722245	0	0	NA	NA
DMRcontig15064:2521350	2.5E+07	25213608	108	1	0.000635291	9	8.333333333	NA	NA
DMRcontig15105:2527431	2.5E+07	25275000	689	2	0.000214158	32	4.644412192	NA	NA
DMRcontig15281:2553210	2.6E+07	25532266	165	2	0.000232097	5	3.03030303	NA	NA
DMRcontig15291:2554640	2.6E+07	25546600	200	2	0.000106552	8	4	NA	NA
DMRcontig15297:2555510	2.6E+07	25555700	600	3	0.000201793	52	8.666666667	NA	NA
DMRcontig15309:2557211	2.6E+07	25572300	182	1	0.000384	7	3.846153846	NA	NA
DMRcontig15312:2557700	2.6E+07	25577940	940	2	0.000517732	60	6.382978723	NA	NA
DMRcontig15315:2558300	2.6E+07	25583200	200	1	8.51E-05	7	3.5	NA	NA

DMRcontig15332:2560792	2.6E+07	25608400	475	1	0.000982339	22	4.631578947	NA	NA
DMRcontig15350:2563403	2.6E+07	25634560	522	1	0.000752525	16	3.0651341	NA	NA
DMRcontig15375:2567072	2.6E+07	25671100	380	2	2.99E-05	29	7.631578947	NA	NA
DMRcontig15378:2567550	2.6E+07	25675700	200	1	0.000518217	5	2.5	NA	NA
DMRcontig15391:2569410	2.6E+07	25694300	200	1	0.000291743	5	2.5	NA	NA
DMRcontig15410:2572200	2.6E+07	25722600	600	2	0.00026941	21	3.5	NA	NA
DMRcontig15418:2573420	2.6E+07	25734300	100	1	0.000755434	4	4	NA	NA
DMRcontig15461:2579610	2.6E+07	25796600	500	4	3.59E-06	21	4.2	NA	NA
DMRcontig15499:2585291	2.6E+07	25853300	382	2	9.14E-05	11	2.879581152	NA	NA
DMRcontig15503:2585870	2.6E+07	25859352	652	1	7.22E-05	43	6.595092025	NA	NA
DMRcontig15506:2586380	2.6E+07	25864400	600	1	2.85E-05	30	5	NA	NA
DMRcontig15507:2586544	2.6E+07	25865669	229	1	0.000119154	9	3.930131004	NA	NA
DMRcontig15545:2591883	2.6E+07	25919200	364	2	9.12E-05	11	3.021978022	NA	NA
DMRcontig15559:2593970	2.6E+07	25939900	200	1	0.00047413	2	1	NA	NA
DMRcontig15561:2594242	2.6E+07	25942899	477	3	0.000109949	4	0.838574423	NA	NA
DMRcontig15587:2598000	2.6E+07	25980100	100	1	0.000523183	4	4	NA	NA
DMRcontig15604:2600480	2.6E+07	26005200	400	2	2.57E-05	8	2	NA	NA
DMRcontig15616:2602190	2.6E+07	26022200	300	2	0.000259823	30	10	NA	NA
DMRcontig15624:2603400	2.6E+07	26034400	398	1	0.000178557	14	3.51758794	NA	NA
DMRcontig15627:2603831	2.6E+07	26038882	565	1	0.000451319	13	2.300884956	NA	NA
DMRcontig15631:2604412	2.6E+07	26044500	372	2	0.00034617	5	1.344086022	NA	NA
DMRcontig15635:2604962	2.6E+07	26050062	439	1	2.29E-06	18	4.10022779	NA	NA
DMRcontig15648:2606900	2.6E+07	26069300	300	2	0.000352541	5	1.666666667	NA	NA
DMRcontig15656:2608045	2.6E+07	26080800	342	1	0.000534818	5	1.461988304	NA	NA
DMRcontig15696:2613640	2.6E+07	26136800	400	1	0.000741318	9	2.25	NA	NA
DMRcontig15728:2618380	2.6E+07	26183900	100	1	0.000195753	2	2	NA	NA
DMRcontig15734:2619218	2.6E+07	26192795	607	3	4.89E-07	38	6.26029654	NA	NA
DMRcontig15740:2620050	2.6E+07	26200800	300	1	0.000398095	12	4	NA	NA
DMRcontig15782:2626210	2.6E+07	26262300	200	2	0.000284681	12	6	NA	NA
DMRcontig15793:2627744	2.6E+07	26277900	459	1	0.000754457	21	4.575163399	NA	NA
DMRcontig15819:2631540	2.6E+07	26315909	509	2	0.000401498	24	4.715127701	NA	NA
DMRcontig15823:2632130	2.6E+07	26321600	300	1	0.000681658	5	1.666666667	NA	NA
DMRcontig15825:2632480	2.6E+07	26325300	500	1	0.000268871	16	3.2	NA	NA
DMRcontig15843:2635150	2.6E+07	26351800	300	1	0.000955863	8	2.666666667	NA	NA
DMRcontig15863:2638040	2.6E+07	26380572	172	1	0.000163299	8	4.651162791	NA	NA
DMRcontig15970:2653290	2.7E+07	26533200	300	1	9.45E-05	1	0.333333333	NA	NA
DMRcontig15989:2656136	2.7E+07	26561600	239	1	0.000130943	7	2.928870293	NA	NA
DMRcontig16014:2659770	2.7E+07	26597995	295	1	0.000942657	16	5.423728814	NA	NA
DMRcontig16039:2663439	2.7E+07	26634500	102	1	0.000221273	0	0	NA	NA
DMRcontig16078:2669110	2.7E+07	26691400	300	1	0.000684237	6	2	NA	NA
DMRcontig16085:2670170	2.7E+07	26701954	254	1	0.000625559	19	7.480314961	NA	NA
DMRcontig16114:2674348	2.7E+07	26744000	520	1	0.00014187	16	3.076923077	NA	NA
DMRcontig16116:2674667	2.7E+07	26747047	369	2	0.000215888	2	0.54200542	NA	NA
DMRcontig16139:2677970	2.7E+07	26779900	200	1	0.000497995	1	0.5	NA	NA
DMRcontig16221:2690026	2.7E+07	26900600	334	3	6.23E-06	18	5.389221557	NA	NA
DMRcontig16283:2699190	2.7E+07	26992400	500	2	8.11E-07	30	6	NA	NA
DMRcontig16289:2700060	2.7E+07	27000800	199	2	1.03E-05	8	4.020100503	NA	NA
DMRcontig16331:2706103	2.7E+07	27061554	517	1	0.000408395	48	9.284332689	NA	NA
DMRcontig16394:2715334	2.7E+07	27153700	355	3	7.25E-05	22	6.197183099	NA	NA
DMRcontig16414:2718280	2.7E+07	27183100	300	1	0.000284605	20	6.666666667	TRIVIDRAFT_208649	Unknown
DMRcontig16493:2729430	2.7E+07	27294500	200	1	0.000320742	6	3	NA	NA
DMRcontig16582:2742360	2.7E+07	27423991	391	2	1.90E-05	10	2.557544757	NA	NA
DMRcontig16583:2742500	2.7E+07	27425200	200	1	0.000257215	2	1	NA	NA
DMRcontig16599:2744780	2.7E+07	27448156	356	3	6.09E-05	15	4.213483146	NA	NA
DMRcontig16614:2746870	2.7E+07	27468900	200	1	0.000620079	2	1	NA	NA
DMRcontig16689:2757770	2.8E+07	27577900	200	1	0.000621663	11	5.5	NA	NA
DMRcontig16729:2763680	2.8E+07	27636928	128	2	8.22E-05	0	0	CARUB_v10020777mg;L484_001827	Unknown
DMRcontig16768:2769250	2.8E+07	27692700	200	1	0.000212233	16	8	NA	NA
DMRcontig16773:2769950	2.8E+07	27699782	280	2	0.000581297	7	2.5	NA	NA
DMRcontig16782:2771340	2.8E+07	27713800	400	3	1.28E-07	22	5.5	NA	NA
DMRcontig16789:2772380	2.8E+07	27724300	500	1	0.000730431	11	2.2	NA	NA
DMRcontig16808:2775271	2.8E+07	27753000	284	1	6.98E-05	17	5.985915493	NA	NA
DMRcontig16889:2786794	2.8E+07	27868567	627	2	1.42E-05	32	5.103668262	NA	NA
DMRcontig16914:2790490	2.8E+07	27905380	480	2	0.000592257	17	3.541666667	NA	NA
DMRcontig16988:2801330	2.8E+07	28013500	200	1	0.000247765	1	0.5	NA	NA
DMRcontig17020:2805890	2.8E+07	28059000	100	1	0.000176188	0	0	NA	NA
DMRcontig17045:2809420	2.8E+07	28094335	135	2	7.90E-06	8	5.925925926	NA	NA
DMRcontig17049:2809920	2.8E+07	28099849	649	1	1.44E-05	9	1.386748844	NA	NA
DMRcontig17086:2815210	2.8E+07	28152261	161	1	0.000747783	10	6.211180124	NA	NA
DMRcontig17096:2816619	2.8E+07	28166500	302	2	5.61E-05	10	3.311258278	NA	NA
DMRcontig17125:2820790	2.8E+07	28208100	199	1	0.000399381	4	2.010050251	NA	NA
DMRcontig17135:2822132	2.8E+07	28221600	281	1	0.00062283	20	7.117437722	NA	NA
DMRcontig17182:2828810	2.8E+07	28288659	559	4	1.89E-08	5	0.894454383	NA	NA
DMRcontig17195:2830697	2.8E+07	28307625	648	2	1.79E-05	27	4.166666667	NA	NA
DMRcontig17219:2834050	2.8E+07	28341300	800	1	0.000248275	17	2.125	NA	NA

DMRcontig17283:2843250	2.8E+07	28432890	390	1	0.000544468	18	4.615384615	NA	NA
DMRcontig17375:2856230	2.9E+07	28562600	300	2	0.000159473	15	5	NA	NA
DMRcontig17460:2868118	2.9E+07	28681600	421	1	2.98E-05	33	7.83847981	NA	NA
DMRcontig17522:2876870	2.9E+07	28768900	200	1	0.000876667	6	3	NA	NA
DMRcontig17543:2879880	2.9E+07	28799200	400	1	0.000494689	6	1.5	NA	NA
DMRcontig17545:2880174	2.9E+07	28801900	158	1	0.000372241	4	2.53164557	NA	NA
DMRcontig17604:2888626	2.9E+07	28886632	373	3	1.51E-06	8	2.144772118	NA	NA
DMRcontig17694:2901516	2.9E+07	29015379	217	1	0.000189038	8	3.686635945	NA	NA
DMRcontig17713:2904030	2.9E+07	29040400	100	1	0.0004249	0	0	NA	NA
DMRcontig17736:2907320	2.9E+07	29073500	300	1	0.000441916	6	2	NA	NA
DMRcontig17788:2914980	2.9E+07	29150400	593	1	0.000218026	37	6.239460371	NA	NA
DMRcontig17833:2921282	2.9E+07	29213100	275	1	7.52E-06	13	4.727272727	NA	NA
DMRcontig17893:2929690	2.9E+07	29297100	200	2	3.28E-06	8	4	NA	NA
DMRcontig17895:2929983	2.9E+07	29300100	267	1	0.000964188	9	3.370786517	NA	NA
DMRcontig17901:2930821	2.9E+07	29308500	283	1	0.000313703	4	1.413427562	NA	NA
DMRcontig17955:2938270	2.9E+07	29382900	200	1	0.000262377	12	6	NA	NA
DMRcontig17974:2941030	2.9E+07	29410600	300	1	0.000180533	3	1	NA	NA
DMRcontig17978:2941600	2.9E+07	29416300	300	3	0.000154123	28	9.333333333	NA	NA
DMRcontig17991:2943440	2.9E+07	29434700	300	1	0.000914463	5	1.666666667	NA	NA
DMRcontig17995:2944052	2.9E+07	29440700	177	1	0.000977398	2	1.129943503	NA	NA
DMRcontig18023:2947950	2.9E+07	29479800	300	1	0.000914463	4	1.333333333	NA	NA
DMRcontig18035:2949610	2.9E+07	29496500	400	1	0.000310161	14	3.5	NA	NA
DMRcontig18047:2951260	3E+07	29512777	177	1	2.40E-05	5	2.824858757	NA	NA
DMRcontig18068:2954091	3E+07	29541100	191	1	0.000344692	9	4.712041885	NA	NA
DMRcontig18079:2955610	3E+07	29556500	400	2	6.70E-05	16	4	NA	NA
DMRcontig18084:2956370	3E+07	29563988	288	2	0.000374843	6	2.083333333	NA	NA
DMRcontig18114:2960650	3E+07	29606800	300	2	0.000128311	21	7	NA	NA
DMRcontig18177:2969540	3E+07	29695900	500	2	6.94E-05	19	3.8	NA	NA
DMRcontig18195:2972200	3E+07	29722200	200	1	0.000293859	2	1	NA	NA
DMRcontig18236:2977817	3E+07	29778300	131	1	0.000124777	1	0.763358779	NA	NA
DMRcontig18240:2978420	3E+07	29784400	200	1	0.000280136	10	5	NA	NA
DMRcontig18248:2979590	3E+07	29796000	100	1	0.000760745	3	3	NA	NA
DMRcontig18273:2983200	3E+07	29832600	600	2	0.000674412	7	1.166666667	NA	NA
DMRcontig18291:2985680	3E+07	29857300	499	1	4.77E-05	45	9.018036072	NA	NA
DMRcontig18316:2989290	3E+07	29893000	100	1	0.000827677	7	7	NA	NA
DMRcontig18359:2995250	3E+07	29953000	495	4	6.45E-14	17	3.434343434	NA	NA
DMRcontig18366:2996290	3E+07	29963136	236	1	0.000160634	8	3.389830508	NA	NA
DMRcontig18391:2999810	3E+07	29998592	492	2	4.02E-05	6	1.219512195	NA	NA
DMRcontig18395:3000390	3E+07	30004800	900	2	9.84E-07	49	5.444444444	cgn	Cytoskeleton
DMRcontig18451:3008555	3E+07	30086000	446	1	0.000335458	12	2.69058296	NA	NA
DMRcontig18481:3013070	3E+07	30131000	300	1	1.89E-05	7	2.333333333	NA	NA
DMRcontig18494:3015010	3E+07	30150628	528	1	0.000179066	28	5.303030303	NA	NA
DMRcontig18497:3015480	3E+07	30155000	200	1	0.000823897	5	2.5	NA	NA
DMRcontig18511:3017514	3E+07	30175300	155	1	0.000661361	2	1.290322581	NA	NA
DMRcontig18601:3030463	3E+07	30305000	370	1	0.000944684	8	2.162162162	NA	NA
DMRcontig18619:3032890	3E+07	30329141	241	2	4.06E-05	1	0.414937759	NA	NA
DMRcontig18627:3034049	3E+07	30340600	109	1	0.000621158	1	0.917431193	NA	NA
DMRcontig18635:3035140	3E+07	30351800	400	1	3.55E-05	23	5.75	NA	NA
DMRcontig18639:3035810	3E+07	30358400	300	1	8.43E-05	23	7.666666667	NA	NA
DMRcontig18671:3039990	3E+07	30400296	396	1	0.000225377	17	4.292929293	NA	NA
DMRcontig18681:3041459	3E+07	30415466	874	4	3.75E-06	73	8.352402746	NA	NA
DMRcontig18706:3045400	3E+07	30454100	100	1	0.000857075	5	5	BRAFLDRAFT_288811	Unknown
DMRcontig18720:3047780	3E+07	30477900	100	1	0.0005476	6	6	BRAFLDRAFT_114927	Unknown
DMRcontig18737:3050560	3.1E+07	30506200	600	1	0.000325539	44	7.333333333	NA	NA
DMRcontig18751:3052950	3.1E+07	30529780	280	2	3.72E-07	0	0	NA	NA
DMRcontig18824:3064440	3.1E+07	30644900	500	1	0.000932159	21	4.2	NA	NA
DMRcontig18848:3068260	3.1E+07	30683637	1037	2	3.94E-05	19	1.832208293	NA	NA
DMRcontig18892:3075170	3.1E+07	30752400	700	2	1.14E-05	34	4.857142857	NA	NA
DMRcontig18897:3075944	3.1E+07	30759641	198	1	0.000165922	0	0	NA	NA
DMRcontig18911:3078132	3.1E+07	30781500	175	1	0.000652376	1	0.571428571	COX1;ATP8	Metabolism
DMRcontig18931:3081133	3.1E+07	30811700	363	1	0.000228938	15	4.132231405	NA	NA
DMRcontig18932:3081310	3.1E+07	30813500	400	1	0.000677477	2	0.5	NA	NA
DMRcontig19030:3096232	3.1E+07	30962500	181	1	0.000213425	8	4.419889503	NA	NA
DMRcontig19032:3096540	3.1E+07	30965757	357	1	4.21E-05	9	2.521008403	NA	NA
DMRcontig19048:3098991	3.1E+07	30990300	387	4	4.30E-07	15	3.875968992	NA	NA
DMRcontig19092:3105466	3.1E+07	31054900	237	1	0.000806223	7	2.953586498	NA	NA
DMRcontig19104:3107290	3.1E+07	31073300	392	1	0.000362633	32	8.163265306	NA	NA
DMRcontig19213:3123412	3.1E+07	31234300	172	2	5.79E-05	5	2.906976744	NA	NA
DMRcontig19240:3127530	3.1E+07	31275449	149	1	0.000268871	5	3.355704698	NA	NA
DMRcontig19262:3130825	3.1E+07	31308800	548	1	1.93E-05	17	3.102189781	NA	NA
DMRcontig19294:3135880	3.1E+07	31359600	800	1	0.000884085	25	3.125	EMWEY_00012150;cno	Unknown
DMRcontig19369:3147010	3.1E+07	31470300	200	1	0.000902602	11	5.5	NA	NA
DMRcontig19388:3149890	3.1E+07	31499400	500	1	0.000302514	33	6.6	NA	NA
DMRcontig19463:3160850	3.2E+07	31608800	300	2	0.000357289	8	2.666666667	NA	NA
DMRcontig19491:3164930	3.2E+07	31649800	496	1	0.000103081	4	0.806451613	NA	NA
DMRcontig19521:3169420	3.2E+07	31694900	700	1	0.000903165	29	4.142857143	NA	NA

DMRcontig19522:3169591	3.2E+07	31696199	290	1	0.000823897	2	0.689655172	NA	NA
DMRcontig19552:3173760	3.2E+07	31738000	400	1	8.21E-05	22	5.5	NA	NA
DMRcontig19559:3174840	3.2E+07	31748940	540	1	0.000555861	8	1.481481481	NA	NA
DMRcontig19561:3175150	3.2E+07	31751870	370	1	0.000657249	10	2.702702703	NA	NA
DMRcontig19564:3175580	3.2E+07	31756100	300	1	0.000496473	5	1.666666667	NA	NA
DMRcontig19577:3177450	3.2E+07	31774900	400	1	2.65E-05	27	6.75	NA	NA
DMRcontig19584:3178410	3.2E+07	31784653	553	2	2.49E-05	14	2.53164557	NA	NA
DMRcontig19590:3179268	3.2E+07	31792973	291	2	1.72E-07	19	6.529209622	NA	NA
DMRcontig19604:3181203	3.2E+07	31812200	163	1	0.000666587	3	1.840490798	NA	NA
DMRcontig19613:3182600	3.2E+07	31826400	400	2	0.000155987	6	1.5	NA	NA
DMRcontig19670:3190830	3.2E+07	31908500	200	1	0.000579112	1	0.5	NA	NA
DMRcontig19744:3201490	3.2E+07	32015100	200	1	0.000944176	10	5	NA	NA
DMRcontig19764:3204450	3.2E+07	32044657	157	1	2.07E-05	5	3.184713376	NA	NA
DMRcontig19766:3204700	3.2E+07	32047375	375	1	0.000313703	11	2.933333333	NA	NA
DMRcontig19791:3208360	3.2E+07	32083700	100	1	0.000729815	0	0	NA	NA
DMRcontig19817:3212233	3.2E+07	32123051	719	4	0.00017273	41	5.702364395	NA	NA
DMRcontig19833:3214600	3.2E+07	32146400	400	2	4.38E-05	16	4	NA	NA
DMRcontig19851:3217201	3.2E+07	32172798	780	2	5.19E-05	39	5	NA	NA
DMRcontig19867:3219620	3.2E+07	32196300	100	1	0.000944176	1	1	NA	NA
DMRcontig19891:3223080	3.2E+07	32231300	500	1	0.000640363	24	4.8	NA	NA
DMRcontig19899:3224390	3.2E+07	32244259	359	1	0.000436661	16	4.456824513	NA	NA
DMRcontig19934:3229410	3.2E+07	32294200	100	1	0.000915364	0	0	NA	NA
DMRcontig19935:3229556	3.2E+07	32295800	236	1	0.000362061	8	3.389830508	NA	NA
DMRcontig19942:3230575	3.2E+07	32306000	246	1	6.39E-05	5	2.032520325	NA	NA
DMRcontig19946:3231150	3.2E+07	32311993	493	1	5.92E-05	38	7.707910751	NA	NA
DMRcontig19964:3233900	3.2E+07	32339471	471	2	5.98E-05	28	5.944798301	NA	NA
DMRcontig20017:3241900	3.2E+07	32419134	134	1	3.21E-06	7	5.223880597	NA	NA
DMRcontig20097:3254590	3.3E+07	32546100	200	1	0.000115457	6	3	NA	NA
DMRcontig20107:3256170	3.3E+07	32562400	700	1	0.000507217	20	2.857142857	NA	NA
DMRcontig20111:3256840	3.3E+07	32568600	200	1	0.000206513	9	4.5	NA	NA
DMRcontig20130:3259855	3.3E+07	32599000	444	3	7.14E-06	20	4.504504505	NA	NA
DMRcontig20229:3275260	3.3E+07	32753100	500	1	0.000306705	5	1	NA	NA
DMRcontig20248:3278180	3.3E+07	32782300	500	1	0.000158812	33	6.6	NA	NA
DMRcontig20267:3281080	3.3E+07	32811122	322	1	9.24E-06	20	6.211180124	NA	NA
DMRcontig20286:3283990	3.3E+07	32840100	200	2	7.89E-05	11	5.5	NA	NA
DMRcontig20296:3285484	3.3E+07	32855375	527	3	6.51E-05	33	6.261859583	NA	NA
DMRcontig20358:3294475	3.3E+07	32945100	345	4	1.04E-06	17	4.927536232	CNPV234	Cytoskeleton
DMRcontig20610:3328932	3.3E+07	33289500	181	2	2.85E-06	0	0	NA	NA
DMRcontig20618:3329970	3.3E+07	33299900	193	2	6.52E-10	3	1.554404145	NA	NA
DMRcontig20698:3340450	3.3E+07	33404700	200	1	0.000127628	13	6.5	NA	NA
DMRcontig20735:3344870	3.3E+07	33449200	500	3	1.27E-06	23	4.6	NA	NA
DMRcontig20837:3357980	3.4E+07	33580060	260	1	0.000485839	4	1.538461538	NA	NA
DMRcontig20925:3369250	3.4E+07	33692800	300	1	0.000565202	4	1.333333333	NA	NA
DMRcontig20988:3377337	3.4E+07	33773700	324	2	0.00017602	12	3.703703704	NA	NA
DMRcontig21153:3397861	3.4E+07	33979000	386	1	0.000208	11	2.849740933	NA	NA
DMRcontig21163:3399288	3.4E+07	33993174	295	1	0.000151059	19	6.440677966	NA	NA
DMRcontig21204:3405340	3.4E+07	34053700	300	1	0.000537767	14	4.666666667	NA	NA
DMRcontig21247:3411070	3.4E+07	34110858	158	1	0.000473732	3	1.898734177	NA	NA
DMRcontig21700:3460733	3.5E+07	34607586	256	1	0.000437068	7	2.734375	NA	NA
DMRcontig21710:3461893	3.5E+07	34619200	270	1	0.000395609	10	3.703703704	NA	NA
DMRcontig22059:3501410	3.5E+07	35014300	200	1	4.73E-05	14	7	LOC101857814	Unknown
DMRcontig22290:3528010	3.5E+07	35280262	162	1	0.000715017	15	9.259259259	NA	NA
DMRcontig22440:3545378	3.5E+07	35453900	112	1	0.00017756	0	0	NA	NA
DMRcontig22489:3551153	3.6E+07	35511574	40	1	0.000270054	1	2.5	NA	NA
DMRcontig22598:3563990	3.6E+07	35640070	170	1	3.94E-05	2	1.176470588	NA	NA
DMRcontig22676:3573460	3.6E+07	35734833	233	1	5.45E-06	4	1.716738197	NA	NA
DMRcontig22706:3576793	3.6E+07	35768300	364	2	5.07E-06	21	5.769230769	NA	NA
DMRcontig22771:3584394	3.6E+07	35844200	261	1	9.03E-05	21	8.045977011	NA	NA
DMRcontig22783:3585852	3.6E+07	35858800	278	1	0.000516888	22	7.913669065	NA	NA
DMRcontig22832:3591620	3.6E+07	35916413	213	2	1.78E-05	4	1.877934272	NA	NA
DMRcontig23049:3617060	3.6E+07	36170800	200	1	0.000528858	5	2.5	NA	NA
DMRcontig23158:3629874	3.6E+07	36298887	142	2	3.61E-05	4	2.816901408	NA	NA
DMRcontig23175:3631811	3.6E+07	36318224	115	1	1.09E-05	2	1.739130435	NA	NA
DMRcontig23275:3643510	3.6E+07	36435595	495	2	2.78E-06	53	10.70707071	NA	NA
DMRcontig23331:3650032	3.7E+07	36500500	176	1	1.38E-06	3	1.704545455	NA	NA
DMRcontig23343:3651390	3.7E+07	36514089	189	1	0.000952129	5	2.645502646	NA	NA
DMRcontig23348:3651970	3.7E+07	36519991	291	1	4.26E-05	11	3.780068729	NA	NA
DMRcontig23378:3655590	3.7E+07	36556170	270	1	0.000914463	10	3.703703704	NA	NA
DMRcontig23389:3656836	3.7E+07	36568500	141	2	0.000122243	4	2.836879433	NA	NA
DMRcontig23683:3690689	3.7E+07	36907000	109	1	0.000396532	0	0	NA	NA
DMRcontig24039:3732790	3.7E+07	37328300	400	2	1.57E-06	17	4.25	NA	NA
DMRcontig24044:3733630	3.7E+07	37336700	400	1	0.000944019	16	4	NA	NA
DMRcontig24067:3737340	3.7E+07	37373600	200	1	0.000106594	7	3.5	NA	NA
DMRcontig24082:3739706	3.7E+07	37397300	232	1	5.05E-05	16	6.896551724	NA	NA
DMRcontig24120:3745655	3.7E+07	37457135	586	1	2.83E-05	15	2.559726962	NA	NA
DMRcontig24154:3750820	3.8E+07	37508588	388	1	0.000127769	7	1.804123711	NA	NA

DMRcontig24162:3752140	3.8E+07	37521600	200	1	0.000159318	8	4	NA	NA
DMRcontig24216:3760005	3.8E+07	37600500	445	2	3.67E-06	17	3.820224719	NA	NA
DMRcontig24286:3770140	3.8E+07	37701700	294	1	0.000715102	20	6.802721088	NA	NA
DMRcontig24552:3806401	3.8E+07	38064100	90	1	0.000120487	1	1.111111111	NA	NA
DMRcontig24604:3812722	3.8E+07	38127700	479	1	0.00098166	18	3.75782881	NA	NA
DMRcontig24789:3836642	3.8E+07	38366796	370	1	4.66E-05	15	4.054054054	NA	NA
DMRcontig24875:3847380	3.8E+07	38474177	377	1	0.000577562	9	2.387267905	NA	NA
DMRcontig24999:3863510	3.9E+07	38635457	357	1	0.00035531	24	6.722689076	NA	NA
DMRcontig25431:3911441	3.9E+07	39114700	283	3	2.05E-06	23	8.127208481	LEMA_P124570.1;LEMA_P124550.1	Signaling
DMRcontig25699:3942167	3.9E+07	39421900	231	1	0.000830748	9	3.896103896	NA	NA
DMRcontig25726:3945346	3.9E+07	39453541	80	2	0.000463609	0	0	NA	NA
DMRcontig25785:3952112	4E+07	39521400	280	1	0.000647151	2	0.714285714	NA	NA
DMRcontig25962:3972480	4E+07	39725100	300	1	0.000268871	15	5	NA	NA
DMRcontig26015:3978805	4E+07	39788270	214	2	1.27E-07	8	3.738317757	NA	NA
DMRcontig26030:3980520	4E+07	39805351	151	1	0.000681618	4	2.649006623	NA	NA
DMRcontig26217:4003043	4E+07	40030560	130	1	0.000948168	8	6.153846154	NA	NA
DMRcontig26255:4007550	4E+07	40075757	257	2	9.80E-06	11	4.280155642	NA	NA
DMRcontig26323:4015535	4E+07	40155700	351	1	0.00054102	10	2.849002849	NA	NA
DMRcontig26480:4034110	4E+07	40341400	300	2	2.05E-07	9	3	NA	NA
DMRcontig26698:4059461	4.1E+07	40594666	57	1	0.000876545	1	1.754385965	NA	NA
DMRcontig26805:4072080	4.1E+07	40721100	300	1	0.000793837	15	5	NA	NA
DMRcontig26840:4076130	4.1E+07	40761400	100	1	0.000318216	9	9	NA	NA
DMRcontig26932:4087091	4.1E+07	40871299	383	2	9.19E-06	7	1.82767624	NA	NA
DMRcontig27014:4096782	4.1E+07	40968243	415	2	0.000116899	13	3.13253012	NA	NA
DMRcontig27117:4108720	4.1E+07	41087343	143	1	0.000827444	7	4.895104895	NA	NA
DMRcontig27327:4133040	4.1E+07	41330900	500	1	0.000721088	20	4	NA	NA
DMRcontig27386:4143050	4.1E+07	41431400	900	1	0.000217643	24	2.666666667	NA	NA
DMRcontig27396:4144800	4.1E+07	41448098	98	1	5.58E-06	9	9.183673469	NA	NA
DMRcontig27398:4145120	4.1E+07	41451500	300	1	7.93E-05	17	5.666666667	NA	NA
DMRcontig27405:4146185	4.1E+07	41462300	445	1	0.000644504	9	2.02247191	NA	NA
DMRcontig27541:4167630	4.2E+07	41676500	200	1	0.000260887	3	1.5	NA	NA
DMRcontig27595:4175610	4.2E+07	41756500	400	2	2.53E-06	14	3.5	NA	NA
DMRcontig27626:4180240	4.2E+07	41802573	173	1	3.85E-05	12	6.936416185	NA	NA
DMRcontig27694:4190420	4.2E+07	41904467	267	1	0.000928448	18	6.741573034	NA	NA
DMRcontig27701:4191400	4.2E+07	41914355	355	1	1.44E-06	22	6.197183099	NA	NA
DMRcontig27753:4198880	4.2E+07	41989100	300	2	0.000303719	8	2.666666667	NA	NA
DMRcontig27759:4199700	4.2E+07	41997200	200	1	0.000153997	4	2	NA	NA
DMRcontig27827:4209380	4.2E+07	42093900	100	1	0.000432466	6	6	NA	NA
DMRcontig27828:4209513	4.2E+07	42095700	566	1	0.000749176	9	1.590106007	NA	NA
DMRcontig27889:4218070	4.2E+07	42180900	199	1	0.000570304	12	6.030150754	NA	NA
DMRcontig27901:4219830	4.2E+07	42198700	400	2	4.45E-05	11	2.75	NA	NA
DMRcontig27909:4220970	4.2E+07	42210100	400	2	5.18E-05	6	1.5	NA	NA
DMRcontig27989:4231750	4.2E+07	42317893	393	2	0.000103253	17	4.325699746	NA	NA
DMRcontig28049:4239572	4.2E+07	42395800	81	1	4.76E-05	3	3.703703704	NA	NA
DMRcontig28050:4239710	4.2E+07	42397177	77	1	3.92E-05	4	5.194805195	NA	NA
DMRcontig28143:4251340	4.3E+07	42513770	370	2	3.34E-05	17	4.594594595	NA	NA
DMRcontig28192:4258290	4.3E+07	42583028	128	1	0.000315147	3	2.34375	NA	NA
DMRcontig28204:4259980	4.3E+07	42600089	289	1	0.00070881	5	1.730103806	NA	NA
DMRcontig28241:4264930	4.3E+07	42649690	390	1	0.000269839	23	5.897435897	NA	NA
DMRcontig28261:4267680	4.3E+07	42677300	500	2	5.65E-05	7	1.4	NA	NA
DMRcontig28285:4271010	4.3E+07	42710300	200	1	0.000300398	1	0.5	NA	NA
DMRcontig28408:4286960	4.3E+07	42870100	500	1	0.000857075	11	2.2	NA	NA
DMRcontig28419:4288357	4.3E+07	42884000	431	1	0.000320705	13	3.016241299	NA	NA
DMRcontig28482:4295950	4.3E+07	42959995	495	1	0.000295266	10	2.02020202	NA	NA
DMRcontig28568:4307719	4.3E+07	43077998	806	1	0.00064307	26	3.225806452	NA	NA
DMRcontig28588:4310653	4.3E+07	43107000	463	1	0.000243354	7	1.51187905	NA	NA
DMRcontig28616:4315010	4.3E+07	43150800	700	1	0.00048339	27	3.857142857	NA	NA
DMRcontig28623:4316120	4.3E+07	43161700	500	1	0.000891209	4	0.8	NA	NA
DMRcontig28631:4317420	4.3E+07	43174600	400	2	1.07E-05	20	5	NA	NA
DMRcontig28659:4321660	4.3E+07	43217000	400	1	2.41E-05	6	1.5	NA	NA
DMRcontig28672:4323600	4.3E+07	43236400	400	2	5.56E-05	6	1.5	NA	NA
DMRcontig28851:4343490	4.3E+07	43435000	100	1	0.000305571	3	3	NA	NA
DMRcontig29161:4378455	4.4E+07	43785000	449	2	0.000525694	24	5.345211581	NA	NA
DMRcontig29199:4382907	4.4E+07	43829147	75	1	0.000977401	3	4	NA	NA
DMRcontig29210:4384200	4.4E+07	43842300	300	1	0.000722604	9	3	NA	NA
DMRcontig29492:4416500	4.4E+07	44165200	200	1	0.000547767	1	0.5	NA	NA
DMRcontig29582:4427187	4.4E+07	44272100	228	2	4.99E-06	15	6.578947368	NA	NA
DMRcontig29689:4439733	4.4E+07	44397542	209	1	0.000831768	0	0	NA	NA
DMRcontig29753:4447275	4.4E+07	44473000	243	1	0.000274542	7	2.880658436	NA	NA
DMRcontig29813:4454513	4.5E+07	44545173	42	1	0.000428679	0	0	NA	NA
DMRcontig29853:4459314	4.5E+07	44593400	255	1	0.000692981	17	6.666666667	NA	NA
DMRcontig29916:4466630	4.5E+07	44666472	172	1	0.000576149	5	2.906976744	NA	NA
DMRcontig29944:4469990	4.5E+07	44700200	300	2	0.000326411	27	9	NA	NA
DMRcontig30050:4482373	4.5E+07	44823900	166	1	0.000351169	4	2.409638554	NA	NA
DMRcontig30070:4484747	4.5E+07	44847631	155	1	4.44E-05	3	1.935483871	NA	NA

DMRcontig30129:4491920	4.5E+07	44919400	200	1	0.000405747	11	5.5	NA	NA
DMRcontig30440:4529200	4.5E+07	45292300	300	2	5.58E-06	3	1	NA	NA
DMRcontig30452:4530670	4.5E+07	45306998	298	1	0.000400653	3	1.006711409	NA	NA
DMRcontig30592:4547440	4.5E+07	45474600	200	1	7.81E-05	10	5	NA	NA
DMRcontig30725:4563300	4.6E+07	45633352	352	2	0.000534772	21	5.965909091	NA	NA
DMRcontig30779:4569890	4.6E+07	45699240	333	2	2.47E-05	10	3.003003003	NA	NA
DMRcontig30826:4575520	4.6E+07	45755585	385	1	0.000396553	6	1.558441558	NA	NA
DMRcontig30866:4580310	4.6E+07	45803257	157	1	4.06E-05	3	1.910828025	NA	NA
DMRcontig30932:4587996	4.6E+07	45880036	68	2	0.000122081	3	4.411764706	NA	NA
DMRcontig31168:4617230	4.6E+07	46172600	300	3	7.63E-05	17	5.666666667	NA	NA
DMRcontig31212:4624161	4.6E+07	46242169	558	2	2.92E-05	31	5.555555556	LOC105888664	Epigenetic
DMRcontig31249:4629769	4.6E+07	46297800	108	1	0.000756084	2	1.851851852	NA	NA
DMRcontig31306:4638370	4.6E+07	46383900	200	1	0.000644232	4	2	NA	NA
DMRcontig31318:4640210	4.6E+07	46402317	217	2	2.70E-07	7	3.225806452	NA	NA
DMRcontig31346:4644260	4.6E+07	46442700	100	1	0.00017756	2	2	NA	NA
DMRcontig31365:4646956	4.6E+07	46469700	139	1	0.000329567	10	7.194244604	NA	NA
DMRcontig31397:4651430	4.7E+07	46514670	370	2	4.27E-05	30	8.108108108	NA	NA
DMRcontig31409:4653080	4.7E+07	46531100	300	1	0.000376446	7	2.333333333	NA	NA
DMRcontig31412:4653552	4.7E+07	46535600	80	1	0.000680896	3	3.75	NA	NA
DMRcontig31449:4658710	4.7E+07	46587400	300	2	0.000445447	19	6.333333333	NA	NA
DMRcontig31471:4661720	4.7E+07	46617600	400	2	0.000165827	7	1.75	NA	NA
DMRcontig31594:4678453	4.7E+07	46784702	170	1	0.000122636	9	5.294117647	NA	NA
DMRcontig31602:4679471	4.7E+07	46795290	575	1	0.000268425	16	2.782608696	NA	NA
DMRcontig31721:4694543	4.7E+07	46945696	263	2	2.53E-05	2	0.760456274	NA	NA
DMRcontig31930:4721090	4.7E+07	47211200	300	1	0.00014151	24	8	NA	NA
DMRcontig32135:4746893	4.7E+07	47469400	466	1	0.000859713	26	5.579399142	NA	NA
DMRcontig33021:4846140	4.8E+07	48461600	197	1	0.000218253	9	4.568527919	NA	NA
DMRcontig33170:4863311	4.9E+07	48633493	383	2	6.03E-05	12	3.133159269	NA	NA
DMRcontig33232:4870402	4.9E+07	48704273	252	1	0.000279967	13	5.158730159	NA	NA
DMRcontig33515:4903040	4.9E+07	49030881	481	5	9.58E-09	26	5.405405405	NA	NA
DMRcontig33988:4957633	5E+07	49576600	269	1	0.000604804	19	7.063197026	NA	NA
DMRcontig34116:4972500	5E+07	49725367	367	1	0.000130301	24	6.539509537	NA	NA
DMRcontig34126:4973705	5E+07	49737469	415	2	2.46E-05	7	1.686746988	NA	NA
DMRcontig34229:4985840	5E+07	49858891	489	1	0.000791469	33	6.748466258	NA	NA
DMRcontig34576:5025770	5E+07	50257900	199	1	8.30E-05	9	4.522613065	NA	NA
DMRcontig34608:5029520	5E+07	50295500	300	1	0.000113973	15	5	NA	NA
DMRcontig34684:5038490	5E+07	50385100	200	1	0.000990822	4	2	NA	NA
DMRcontig34725:5043124	5E+07	50431341	95	1	6.11E-05	1	1.052631579	NA	NA
DMRcontig34755:5046610	5E+07	50466398	292	1	0.000642764	7	2.397260274	NA	NA
DMRcontig34963:5070113	5.1E+07	50701300	168	1	0.000840835	0	0	NA	NA
DMRcontig34993:5073940	5.1E+07	50739600	200	2	7.01E-05	15	7.5	NA	NA
DMRcontig35190:5105802	5.1E+07	51058200	175	1	0.000195843	7	4	NA	NA
DMRcontig35250:5115416	5.1E+07	51154500	339	3	3.72E-05	29	8.554572271	NA	NA
DMRcontig35266:5117940	5.1E+07	51179856	456	1	1.17E-05	15	3.289473684	NA	NA
DMRcontig35288:5121244	5.1E+07	51212700	253	2	7.72E-06	12	4.743083004	NA	NA
DMRcontig35306:5124010	5.1E+07	51240400	300	2	0.00046116	21	7	NA	NA
DMRcontig35330:5127677	5.1E+07	51277200	422	3	7.68E-05	33	7.819905213	NA	NA
DMRcontig35348:5130530	5.1E+07	51305800	500	2	4.73E-05	16	3.2	NA	NA
DMRcontig35381:5135840	5.1E+07	51358900	500	3	4.68E-06	41	8.2	NA	NA
DMRcontig35382:5136010	5.1E+07	51360400	292	2	0.000128569	18	6.164383562	NA	NA
DMRcontig35383:5136180	5.1E+07	51362000	200	1	0.000944176	13	6.5	NA	NA
DMRcontig35446:5145870	5.1E+07	51459000	300	2	2.81E-05	10	3.333333333	NA	NA
DMRcontig35519:5156890	5.2E+07	51569200	294	3	8.12E-06	15	5.102040816	NA	NA
DMRcontig35526:5157961	5.2E+07	51579900	289	1	0.000852682	5	1.730103806	NA	NA
DMRcontig35549:5161445	5.2E+07	51614700	244	1	0.000621354	18	7.37704918	NA	NA
DMRcontig35636:5174871	5.2E+07	51749000	285	1	9.87E-05	16	5.614035088	NA	NA
DMRcontig35670:5180174	5.2E+07	51801900	153	2	0.000156944	12	7.843137255	NA	NA
DMRcontig35725:5188613	5.2E+07	51886300	169	1	0.000860107	8	4.733727811	NA	NA
DMRcontig35746:5191800	5.2E+07	51918492	492	2	0.000504308	14	2.845528455	NA	NA
DMRcontig35787:5198060	5.2E+07	51980858	258	2	4.25E-05	10	3.875968992	NA	NA
DMRcontig35795:5199185	5.2E+07	51992400	550	3	3.82E-06	23	4.181818182	NA	NA
DMRcontig35827:5203960	5.2E+07	52039788	188	1	0.0008021	14	7.446808511	NA	NA
DMRcontig35836:5205331	5.2E+07	52053500	189	1	0.000927546	4	2.116402116	NA	NA
DMRcontig35965:5224495	5.2E+07	52245300	342	1	0.000567663	19	5.555555556	NA	NA
DMRcontig35979:5226620	5.2E+07	52266600	400	1	0.000590747	22	5.5	NA	NA
DMRcontig36041:5236000	5.2E+07	52360368	368	3	1.89E-07	15	4.076086957	NA	NA
DMRcontig36064:5239471	5.2E+07	52394900	191	1	7.65E-05	11	5.759162304	NA	NA
DMRcontig36093:5243666	5.2E+07	52437100	437	1	0.000835627	14	3.203661327	NA	NA
DMRcontig36144:5251370	5.3E+07	52513989	289	2	2.57E-05	7	2.422145329	NA	NA
DMRcontig36183:5257240	5.3E+07	52572500	100	1	0.000547636	5	5	NA	NA
DMRcontig36194:5258823	5.3E+07	52588700	467	3	6.80E-05	31	6.638115632	NA	NA
DMRcontig36196:5259130	5.3E+07	52591837	537	2	3.96E-06	42	7.82122905	NA	NA
DMRcontig36205:5260490	5.3E+07	52605100	200	1	0.000248717	12	6	NA	NA
DMRcontig36206:5260610	5.3E+07	52606300	196	1	0.000734055	6	3.06122449	NA	NA
DMRcontig36208:5260940	5.3E+07	52609600	200	2	0.000174672	8	4	NA	NA
DMRcontig36246:5266502	5.3E+07	52665588	564	6	7.03E-10	30	5.319148936	NA	NA

DMRcontig36279:5271580	5.3E+07	52716000	200	1	7.80E-05	10	5	XRN1	Transcription
DMRcontig36296:5274070	5.3E+07	52740900	200	1	0.000788562	4	2	NA	NA
DMRcontig36382:5286620	5.3E+07	52866398	198	1	0.00046261	3	1.515151515	NA	NA
DMRcontig36423:5292710	5.3E+07	52927500	400	1	5.53E-05	22	5.5	NA	NA
DMRcontig36515:5306380	5.3E+07	53064190	390	2	0.000355878	3	0.769230769	NA	NA
DMRcontig36557:5312520	5.3E+07	53125545	345	2	3.68E-08	22	6.376811594	NA	NA
DMRcontig36576:5315360	5.3E+07	53154066	466	1	0.000508299	20	4.291845494	NA	NA
DMRcontig36597:5318540	5.3E+07	53185600	200	1	0.00031883	14	7	NA	NA
DMRcontig36628:5323200	5.3E+07	53232270	270	1	0.000151029	10	3.703703704	NA	NA
DMRcontig36686:5331990	5.3E+07	53320280	380	1	0.00018439	25	6.578947368	NA	NA
DMRcontig36701:5334245	5.3E+07	53342888	432	2	5.61E-05	23	5.324074074	NA	NA
DMRcontig36712:5335861	5.3E+07	53359300	690	1	1.82E-05	46	6.666666667	LOC105906487;SMAD3	Epigenetic;Transcription
DMRcontig36854:5356880	5.4E+07	53569300	500	1	0.000470765	11	2.2	NA	NA
DMRcontig36904:5364190	5.4E+07	53642300	400	1	8.97E-06	9	2.25	NA	NA
DMRcontig36912:5365346	5.4E+07	53653600	136	1	0.000268871	7	5.147058824	NA	NA
DMRcontig36955:5371600	5.4E+07	53716300	300	1	0.000738497	10	3.333333333	NA	NA
DMRcontig36996:5377730	5.4E+07	53777500	200	1	0.000270916	4	2	NA	NA
DMRcontig37023:5381705	5.4E+07	53817781	728	2	0.000430935	30	4.120879121	NA	NA
DMRcontig37026:5382190	5.4E+07	53822168	268	2	0.000152833	11	4.104477612	NA	NA
DMRcontig37082:5390313	5.4E+07	53903467	338	1	0.000566279	11	3.25443787	plcg2	Metabolism
DMRcontig37105:5393670	5.4E+07	53936952	252	2	4.37E-06	7	2.777777778	NA	NA
DMRcontig37106:5393795	5.4E+07	53938400	448	1	0.000400625	26	5.803571429	NA	NA
DMRcontig37144:5399520	5.4E+07	53995500	300	1	6.80E-05	15	5	NA	NA
DMRcontig37187:5405930	5.4E+07	54059700	400	2	0.000605149	15	3.75	NA	NA
DMRcontig37189:5406216	5.4E+07	54062500	336	2	1.53E-05	23	6.845238095	NA	NA
DMRcontig37266:5417690	5.4E+07	54177100	200	1	3.87E-05	5	2.5	NA	NA
DMRcontig37281:5419900	5.4E+07	54199300	300	1	0.000981865	12	4	NA	NA
DMRcontig37315:5424981	5.4E+07	54250100	287	2	0.000112758	13	4.529616725	NA	NA
DMRcontig37384:5434960	5.4E+07	54349990	390	1	0.000594216	27	6.923076923	NA	NA
DMRcontig37481:5449074	5.4E+07	54491193	450	5	4.59E-05	14	3.111111111	NA	NA
DMRcontig37487:5450030	5.5E+07	54500700	400	1	5.57E-05	15	3.75	NA	NA
DMRcontig37545:5458550	5.5E+07	54585971	466	1	0.00045467	28	6.008583691	NA	NA
DMRcontig37551:5459450	5.5E+07	54595200	700	3	2.30E-05	41	5.857142857	NA	NA
DMRcontig37573:5462744	5.5E+07	54627891	450	4	2.70E-08	31	6.888888889	NA	NA
DMRcontig37575:5463034	5.5E+07	54630700	357	2	6.15E-05	20	5.602240896	NA	NA
DMRcontig37626:5470460	5.5E+07	54705200	600	3	1.44E-06	18	3	NA	NA
DMRcontig37659:5475260	5.5E+07	54752700	100	1	0.000644232	2	2	myo9b	Cytoskeleton
DMRcontig37665:5476110	5.5E+07	54761597	497	2	5.56E-05	29	5.83501006	NA	NA
DMRcontig37696:5480660	5.5E+07	54806900	292	1	0.000107057	6	2.054794521	NA	NA
DMRcontig37702:5481530	5.5E+07	54815600	300	2	4.24E-05	12	4	NA	NA
DMRcontig37763:5490510	5.5E+07	54905300	200	1	0.000128081	6	3	NA	NA
DMRcontig37774:5492150	5.5E+07	54921734	234	1	0.000270054	10	4.273504274	NA	NA
DMRcontig37801:5496190	5.5E+07	54962200	300	1	0.000158749	12	4	NA	NA
DMRcontig37818:5498760	5.5E+07	54987890	290	1	0.000940401	4	1.379310345	NA	NA
DMRcontig37881:5508041	5.5E+07	55080600	183	2	4.84E-05	17	9.289617486	NA	NA
DMRcontig37906:5511710	5.5E+07	55117500	400	2	3.83E-05	13	3.25	NA	NA
DMRcontig37937:5516270	5.5E+07	55163100	400	1	3.12E-05	9	2.25	TAL2	Transcription
DMRcontig38092:5539060	5.5E+07	55390900	300	1	0.000538243	2	0.666666667	NA	NA
DMRcontig38102:5540550	5.5E+07	55405823	323	1	0.000453961	25	7.73993808	NA	NA
DMRcontig38128:5544380	5.5E+07	55444100	300	2	0.000213444	7	2.333333333	NA	NA
DMRcontig38142:5546480	5.5E+07	55464900	100	1	0.000472025	2	2	NA	NA
DMRcontig38213:5556790	5.6E+07	55568288	388	3	2.82E-05	14	3.608247423	TRIATDRAFT_160795	Unknown
DMRcontig38228:5559060	5.6E+07	55591309	709	1	0.000389534	20	2.820874471	NA	NA
DMRcontig38250:5562286	5.6E+07	55623331	468	1	0.000109111	26	5.555555556	NA	NA
DMRcontig38295:5568823	5.6E+07	55688612	383	1	0.00010569	21	5.483028721	NA	NA
DMRcontig38347:5576451	5.6E+07	55764899	384	1	0.000689167	8	2.083333333	NA	NA
DMRcontig38359:5578142	5.6E+07	55781848	422	1	0.000305023	6	1.421800948	NA	NA
DMRcontig38379:5581062	5.6E+07	55811092	471	3	3.78E-08	31	6.581740977	NA	NA
DMRcontig38408:5585292	5.6E+07	55853200	273	2	9.28E-05	13	4.761904762	NA	NA
DMRcontig38424:5587610	5.6E+07	55876500	400	3	2.48E-05	18	4.5	NA	NA
DMRcontig38441:5590130	5.6E+07	55901400	100	1	6.65E-05	6	6	NA	NA
DMRcontig38514:5600690	5.6E+07	56007100	200	1	0.000480994	3	1.5	NA	NA
DMRcontig38542:5604734	5.6E+07	56047500	160	1	0.000473732	10	6.25	NA	NA
DMRcontig38564:5607927	5.6E+07	56079700	430	3	1.71E-05	17	3.953488372	NA	NA
DMRcontig38565:5608070	5.6E+07	56080800	97	1	0.000122243	5	5.154639175	NA	NA
DMRcontig38578:5609950	5.6E+07	56099800	300	1	0.000593278	6	2	NA	NA
DMRcontig38597:5612690	5.6E+07	56127200	300	1	0.000993107	3	1	NA	NA
DMRcontig38604:5613660	5.6E+07	56137000	400	1	4.83E-05	16	4	NA	NA
DMRcontig38639:5618670	5.6E+07	56187000	300	2	2.52E-05	20	6.666666667	NA	NA
DMRcontig38656:5621122	5.6E+07	56211500	275	1	0.000580461	12	4.363636364	NA	NA
DMRcontig38682:5624893	5.6E+07	56249400	469	1	2.77E-07	22	4.690831557	NA	NA
DMRcontig38699:5627370	5.6E+07	56274000	300	1	0.000204156	17	5.666666667	NA	NA
DMRcontig38705:5628240	5.6E+07	56282800	400	1	1.53E-05	16	4	NA	NA
DMRcontig38719:5630300	5.6E+07	56303462	462	1	0.000309186	16	3.463203463	NA	NA
DMRcontig38751:5634902	5.6E+07	56349540	517	2	0.000237419	25	4.835589942	NA	NA
DMRcontig38765:5636940	5.6E+07	56369700	300	2	0.000141601	19	6.333333333	NA	NA

DMRcontig38777:5638670	5.6E+07	56387051	351	2	9.01E-06	21	5.982905983	NA	NA
DMRcontig38781:5639225	5.6E+07	56392700	445	1	6.05E-08	15	3.370786517	NA	NA
DMRcontig38790:5640554	5.6E+07	56405800	256	2	9.08E-05	16	6.25	NA	NA
DMRcontig38814:5644044	5.6E+07	56440900	459	3	1.31E-05	32	6.97167756	NA	NA
DMRcontig38819:5644765	5.6E+07	56448146	497	1	0.000288902	25	5.030181087	NA	NA
DMRcontig38822:5645240	5.6E+07	56452500	100	1	0.000647748	2	2	NA	NA
DMRcontig38834:5646943	5.6E+07	56469848	419	3	1.17E-05	14	3.341288783	NA	NA
DMRcontig38837:5647380	5.6E+07	56474100	300	1	0.000997985	17	5.666666667	NA	NA
DMRcontig38851:5649420	5.6E+07	56494530	330	2	2.32E-06	16	4.848484848	NA	NA
DMRcontig38896:5655950	5.7E+07	56560009	509	1	0.00093053	11	2.161100196	NA	NA
DMRcontig38904:5657080	5.7E+07	56571100	300	1	0.000503066	14	4.666666667	NA	NA
DMRcontig38962:5665610	5.7E+07	56656500	400	3	2.08E-05	27	6.75	NA	NA
DMRcontig39010:5672511	5.7E+07	56725600	489	1	0.000596317	13	2.658486708	NA	NA
DMRcontig39037:5676350	5.7E+07	56763800	296	1	0.000795615	28	9.459459459	NA	NA
DMRcontig39048:5678006	5.7E+07	56780400	338	1	0.000534818	3	0.887573964	NA	NA
DMRcontig39066:5680630	5.7E+07	56806600	300	1	0.000902058	3	1	NA	NA
DMRcontig39069:5681080	5.7E+07	56811100	300	2	0.000311503	20	6.666666667	NA	NA
DMRcontig39096:5684953	5.7E+07	56849800	262	2	9.70E-05	10	3.816793893	NA	NA
DMRcontig39100:5685530	5.7E+07	56855894	594	1	3.48E-05	14	2.356902357	NA	NA
DMRcontig39113:5687403	5.7E+07	56874499	470	2	1.86E-05	23	4.893617021	NA	NA
DMRcontig39131:5689990	5.7E+07	56900100	200	1	0.000386725	9	4.5	NA	NA
DMRcontig39290:5712760	5.7E+07	57127749	149	1	0.000488783	9	6.040268456	NA	NA
DMRcontig39308:5715304	5.7E+07	57153442	400	1	0.000263735	14	3.5	NA	NA
DMRcontig39313:5716040	5.7E+07	57160592	192	1	0.00054536	16	8.333333333	NA	NA
DMRcontig39325:5717700	5.7E+07	57177400	400	3	7.08E-07	29	7.25	NA	NA
DMRcontig39334:5719010	5.7E+07	57190472	372	1	0.000229687	9	2.419354839	NA	NA
DMRcontig39336:5719290	5.7E+07	57193300	400	1	6.26E-05	24	6	NA	NA
DMRcontig39365:5723440	5.7E+07	57234800	400	1	0.000757822	20	5	NA	NA
DMRcontig39394:5727580	5.7E+07	57276200	400	2	3.79E-05	18	4.5	NA	NA
DMRcontig39397:5728047	5.7E+07	57280897	420	2	3.77E-05	24	5.714285714	NA	NA
DMRcontig39402:5728760	5.7E+07	57287800	200	1	1.09E-05	14	7	NA	NA
DMRcontig39443:5734730	5.7E+07	57347600	300	1	0.000760745	11	3.666666667	NA	NA
DMRcontig39532:5747540	5.7E+07	57475811	411	3	7.71E-06	30	7.299270073	NA	NA
DMRcontig39568:5752810	5.8E+07	57528365	265	2	0.000427494	9	3.396226415	NA	NA
DMRcontig39635:5762400	5.8E+07	57624291	291	1	8.42E-05	18	6.18556701	NA	NA
DMRcontig39723:5775010	5.8E+07	57750535	435	1	0.000977759	25	5.747126437	NA	NA
DMRcontig39729:5775864	5.8E+07	57758900	256	2	2.82E-08	13	5.078125	NA	NA
DMRcontig39731:5776151	5.8E+07	57762000	488	1	0.000933526	27	5.532786885	NA	NA
DMRcontig39779:5782980	5.8E+07	57830000	200	2	2.88E-05	4	2	NA	NA
DMRcontig39815:5788130	5.8E+07	57881700	397	1	0.0009776	16	4.0302267	NA	NA
DMRcontig39819:5788692	5.8E+07	57887100	175	1	0.000496691	9	5.142857143	NA	NA
DMRcontig39825:5789565	5.8E+07	57896200	543	1	0.000370473	30	5.524861878	NA	NA
DMRcontig39886:5798250	5.8E+07	57982800	300	2	4.16E-05	21	7	NA	NA
DMRcontig39892:5799096	5.8E+07	57991400	432	1	0.000967142	18	4.166666667	NA	NA
DMRcontig40014:5816667	5.8E+07	58166900	227	1	7.37E-07	9	3.964757709	NA	NA
DMRcontig40096:5828460	5.8E+07	58284765	165	1	4.11E-05	4	2.424242424	NA	NA
DMRcontig40115:5831190	5.8E+07	58312153	253	1	0.0004298	22	8.695652174	NA	NA
DMRcontig40122:5832190	5.8E+07	58322400	493	1	0.00092305	24	4.868154158	NA	NA
DMRcontig40243:5849310	5.8E+07	58493200	100	1	0.000435992	4	4	NA	NA
DMRcontig40247:5849895	5.8E+07	58499100	150	1	1.28E-05	2	1.333333333	NA	NA
DMRcontig40252:5850621	5.9E+07	58506500	290	1	9.65E-05	6	2.068965517	si:dkeyp-61b2.1	Unknown
DMRcontig40257:5851330	5.9E+07	58513500	200	1	0.0009311	6	3	NA	NA
DMRcontig40313:5859390	5.9E+07	58594100	200	1	0.000409314	10	5	NA	NA
DMRcontig40315:5859647	5.9E+07	58596999	526	1	0.000757822	20	3.802281369	NA	NA
DMRcontig40349:5864461	5.9E+07	58644900	282	1	0.000415896	13	4.609929078	NA	NA
DMRcontig40378:5868620	5.9E+07	58686400	200	1	0.00026673	4	2	NA	NA
DMRcontig40404:5872304	5.9E+07	58723568	529	2	1.04E-06	25	4.725897921	NA	NA
DMRcontig40451:5878930	5.9E+07	58789476	176	1	0.000806223	2	1.136363636	NA	NA
DMRcontig40506:5886703	5.9E+07	58867300	269	1	4.08E-05	6	2.230483271	NA	NA
DMRcontig40534:5890640	5.9E+07	58906600	200	1	0.000387322	10	5	NA	NA
DMRcontig40588:5898285	5.9E+07	58983100	242	1	5.11E-05	13	5.371900826	NA	NA
DMRcontig40637:5905172	5.9E+07	59052100	381	1	0.000297986	11	2.887139108	NA	NA
DMRcontig40646:5906380	5.9E+07	59064000	200	1	0.000819553	12	6	FAM160A1	Unknown
DMRcontig40649:5906790	5.9E+07	59068200	300	1	0.000530589	12	4	NA	NA
DMRcontig40736:5919060	5.9E+07	59191092	492	2	8.59E-06	25	5.081300813	NA	NA
DMRcontig40764:5923010	5.9E+07	59230546	446	2	3.24E-05	31	6.950672646	NA	NA
DMRcontig40808:5929176	5.9E+07	59292309	550	3	7.49E-06	30	5.454545455	NA	NA
DMRcontig40858:5936170	5.9E+07	59361990	290	1	1.78E-05	19	6.551724138	NA	NA
DMRcontig40863:5936894	5.9E+07	59369300	352	1	0.000440985	25	7.102272727	NA	NA
DMRcontig40946:5948640	5.9E+07	59486626	226	1	0.000624049	4	1.769911504	NA	NA
DMRcontig41001:5956470	6E+07	59564900	200	1	0.000153569	6	3	NA	NA
DMRcontig41062:5964920	6E+07	59649600	400	1	0.00080986	3	0.75	NA	NA
DMRcontig41072:5966330	6E+07	59663600	300	2	1.87E-05	27	9	NA	NA
DMRcontig41152:5977610	6E+07	59776256	156	1	0.000145566	5	3.205128205	NA	NA
DMRcontig41168:5979883	6E+07	59799200	368	2	8.09E-07	18	4.891304348	NA	NA
DMRcontig41301:5998550	6E+07	59985800	297	2	1.50E-05	17	5.723905724	NA	NA

DMRcontig41328:6002310	6E+07	60023400	300	1	7.28E-06	8	2.666666667	NA	NA
DMRcontig41329:6002450	6E+07	60024884	384	1	5.54E-05	6	1.5625	NA	NA
DMRcontig41338:6003730	6E+07	60037500	200	1	0.000534818	9	4.5	NA	NA
DMRcontig41364:6007445	6E+07	60074700	244	1	0.000660296	12	4.918032787	NA	NA
DMRcontig41408:6013710	6E+07	60137420	314	2	2.92E-06	23	7.324840764	NA	NA
DMRcontig41444:6018750	6E+07	60187682	182	1	0.000466349	12	6.593406593	NA	NA
DMRcontig41451:6019720	6E+07	60197400	200	2	5.36E-05	2	1	NA	NA
DMRcontig41461:6021120	6E+07	60211600	400	3	5.67E-05	39	9.75	NA	NA
DMRcontig41498:6026260	6E+07	60262772	172	1	0.000972796	1	0.581395349	NA	NA
DMRcontig41778:6064930	6.1E+07	60649589	289	1	0.00027511	14	4.844290657	NA	NA
DMRcontig41800:6068022	6.1E+07	60680700	473	2	0.000104798	13	2.748414376	NA	NA
DMRcontig41812:6069840	6.1E+07	60698700	300	2	7.44E-05	14	4.666666667	NA	NA
DMRcontig41832:6072590	6.1E+07	60726200	300	2	0.000134551	15	5	NA	NA
DMRcontig41857:6076010	6.1E+07	60760600	500	3	6.82E-07	14	2.8	NA	NA
DMRcontig41861:6076530	6.1E+07	60765600	295	2	7.38E-05	12	4.06779661	NA	NA
DMRcontig41902:6082238	6.1E+07	60822700	314	2	1.57E-09	22	7.006369427	NA	NA
DMRcontig41960:6090340	6.1E+07	60903599	199	2	9.56E-06	1	0.502512563	NA	NA
DMRcontig41999:6095812	6.1E+07	60958500	376	1	0.000208857	10	2.659574468	NA	NA
DMRcontig42083:6107595	6.1E+07	61076100	148	1	0.000734647	7	4.72972973	NA	NA
DMRcontig42125:6113445	6.1E+07	61134987	535	3	1.25E-05	22	4.112149533	NA	NA
DMRcontig42131:6114347	6.1E+07	61143952	479	1	0.000660603	27	5.636743215	NA	NA
DMRcontig42133:6114641	6.1E+07	61146616	207	1	0.000266643	3	1.449275362	NA	NA
DMRcontig42135:6114920	6.1E+07	61149456	256	1	6.09E-06	13	5.078125	NA	NA
DMRcontig42195:6123117	6.1E+07	61231400	231	2	0.000235034	18	7.792207792	NA	NA
DMRcontig42198:6123540	6.1E+07	61235871	471	1	0.00040439	23	4.883227176	NA	NA
DMRcontig42307:6138760	6.1E+07	61388000	400	1	0.000985909	14	3.5	NA	NA
DMRcontig42308:6138902	6.1E+07	61389200	173	1	0.000750748	4	2.312138728	NA	NA
DMRcontig42321:6140670	6.1E+07	61407049	349	1	0.000768458	23	6.59025788	NA	NA
DMRcontig42422:6154900	6.2E+07	61549300	300	2	5.97E-05	9	3	NA	NA
DMRcontig42522:6168680	6.2E+07	61687185	385	2	5.18E-06	19	4.935064935	IscW_ISCW023383;sox2	Development;Transcription
DMRcontig42646:6185660	6.2E+07	61856800	200	2	0.000247898	2	1	NA	NA
DMRcontig42655:6186864	6.2E+07	61868900	260	1	0.000647478	17	6.538461538	NA	NA
DMRcontig42783:6204600	6.2E+07	62046552	552	1	3.89E-07	26	4.710144928	NA	NA
DMRcontig42879:6217758	6.2E+07	62177800	216	1	0.000133987	5	2.314814815	NA	NA
DMRcontig42895:6219985	6.2E+07	62200200	346	3	6.43E-07	7	2.023121387	NA	NA
DMRcontig42923:6223920	6.2E+07	62239400	194	1	0.000937618	10	5.154639175	NA	NA
DMRcontig43062:6242990	6.2E+07	62430100	200	1	9.66E-05	3	1.5	NA	NA
DMRcontig43087:6246410	6.2E+07	62464600	500	1	6.93E-05	11	2.2	NA	NA
DMRcontig43092:6247111	6.2E+07	62471400	283	1	0.000376117	20	7.067137809	NA	NA
DMRcontig43095:6247560	6.2E+07	62475830	230	1	4.56E-05	10	4.347826087	NA	NA
DMRcontig43100:6248240	6.2E+07	62482700	300	2	7.15E-05	11	3.666666667	NA	NA
DMRcontig43151:6255170	6.3E+07	62552171	471	1	1.23E-05	21	4.458598726	NA	NA
DMRcontig43182:6259406	6.3E+07	62594800	740	4	1.39E-05	50	6.756756757	NA	NA
DMRcontig43275:6272284	6.3E+07	62723100	256	1	9.56E-05	5	1.953125	NA	NA
DMRcontig43296:6275160	6.3E+07	62751800	200	2	7.90E-05	10	5	NA	NA
DMRcontig43325:6279033	6.3E+07	62790500	168	1	4.37E-05	4	2.380952381	NA	NA
DMRcontig43329:6279575	6.3E+07	62796100	343	2	1.33E-05	9	2.623906706	NA	NA
DMRcontig43403:6289776	6.3E+07	62898200	434	1	0.000209601	26	5.99078341	NA	NA
DMRcontig43434:6294020	6.3E+07	62940672	472	2	9.35E-05	11	2.330508475	NA	NA
DMRcontig43499:6302970	6.3E+07	63029900	200	1	1.66E-05	11	5.5	LOC101851392;CNOT6	Transcription;Unknown
DMRcontig43512:6304784	6.3E+07	63047965	121	1	0.000237504	0	0	NA	NA
DMRcontig43545:6309157	6.3E+07	63092095	523	5	0.000108015	22	4.206500956	NA	NA
DMRcontig43581:6314030	6.3E+07	63140800	500	1	0.000120756	16	3.2	NA	NA
DMRcontig43621:6319540	6.3E+07	63195700	300	2	1.13E-05	13	4.333333333	NA	NA
DMRcontig43626:6320200	6.3E+07	63202145	145	1	0.000535886	8	5.517241379	NA	NA
DMRcontig43665:6325600	6.3E+07	63256200	200	1	0.000942268	13	6.5	NA	NA
DMRcontig43673:6326700	6.3E+07	63267100	100	1	0.000537963	1	1	NA	NA
DMRcontig43825:6347610	6.3E+07	63476770	665	2	2.79E-06	44	6.616541353	eif4a1b	Transcription
DMRcontig43862:6352629	6.4E+07	63526574	280	2	1.63E-05	6	2.142857143	NA	NA
DMRcontig43864:6352938	6.4E+07	63529600	218	1	2.00E-05	17	7.798165138	NA	NA
DMRcontig43881:6355237	6.4E+07	63552586	210	2	8.41E-06	6	2.857142857	NA	NA
DMRcontig43901:6357921	6.4E+07	63579400	185	1	0.000624049	2	1.081081081	NA	NA
DMRcontig43911:6359240	6.4E+07	63592600	200	1	4.58E-05	9	4.5	NA	NA
DMRcontig43949:6364304	6.4E+07	63643400	356	1	5.81E-05	6	1.685393258	NA	NA
DMRcontig43979:6368420	6.4E+07	63684400	200	2	0.000388802	5	2.5	NA	NA
DMRcontig44009:6372551	6.4E+07	63725900	390	1	0.000593278	15	3.846153846	NA	NA
DMRcontig44030:6375403	6.4E+07	63754235	201	2	3.63E-08	14	6.965174129	NA	NA
DMRcontig44064:6380029	6.4E+07	63800500	210	1	0.000821308	9	4.285714286	NA	NA
DMRcontig44112:6386664	6.4E+07	63866939	296	2	2.51E-06	10	3.378378378	NA	NA
DMRcontig44149:6391640	6.4E+07	63916700	300	1	0.000827444	6	2	NA	NA
DMRcontig44196:6398001	6.4E+07	63980300	287	1	1.27E-05	14	4.87804878	NA	NA
DMRcontig44257:6406685	6.4E+07	64067210	355	2	8.58E-06	28	7.887323944	NA	NA
DMRcontig44265:6407780	6.4E+07	64078100	300	1	0.000855922	16	5.333333333	NA	NA
DMRcontig44314:6414477	6.4E+07	64145000	231	1	0.0009893	10	4.329004329	NA	NA
DMRcontig44330:6416682	6.4E+07	64167000	180	1	2.88E-07	10	5.555555556	NA	NA
DMRcontig44346:6418775	6.4E+07	64187900	148	1	0.000880312	6	4.054054054	NA	NA

DMRcontig44543:6445363	6.4E+07	64453840	209	2	0.000525042	2	0.956937799	NA	NA
DMRcontig44586:6451060	6.5E+07	64510984	384	2	0.000163995	13	3.385416667	NA	NA
DMRcontig44853:6487320	6.5E+07	64873300	95	1	0.0005476	5	5.263157895	NA	NA
DMRcontig44960:6501700	6.5E+07	65017300	300	1	0.000788357	8	2.666666667	NA	NA
DMRcontig45046:6513190	6.5E+07	65132100	200	1	0.000200734	8	4	NA	NA
DMRcontig45187:6532428	6.5E+07	65324600	318	1	1.47E-05	12	3.773584906	NA	NA
DMRcontig45189:6532680	6.5E+07	65327066	259	1	0.000512726	13	5.019305019	NA	NA
DMRcontig45201:6534380	6.5E+07	65344000	200	1	5.45E-05	12	6	NA	NA
DMRcontig45234:6538822	6.5E+07	65388300	77	1	0.000977759	1	1.298701299	NA	NA
DMRcontig45266:6543100	6.5E+07	65431488	488	4	5.56E-09	21	4.303278689	NA	NA
DMRcontig45276:6544470	6.5E+07	65444800	100	1	0.000605553	1	1	NA	NA
DMRcontig45362:6555690	6.6E+07	65557300	400	2	0.000277648	6	1.5	NA	NA
DMRcontig45377:6557713	6.6E+07	65577300	166	1	0.000587681	6	3.614457831	NA	NA
DMRcontig45520:6577243	6.6E+07	65772777	344	1	0.000180533	19	5.523255814	NA	NA
DMRcontig45692:6600060	6.6E+07	66000800	200	1	0.000312099	3	1.5	NA	NA
DMRcontig45703:6601541	6.6E+07	66015600	184	1	0.000108725	11	5.97826087	NA	NA
DMRcontig45856:6621860	6.6E+07	66218818	218	1	0.000153877	8	3.669724771	NA	NA
DMRcontig45867:6623370	6.6E+07	66233900	200	1	3.12E-05	3	1.5	NA	NA
DMRcontig45877:6624743	6.6E+07	66247600	168	1	0.000802942	5	2.976190476	NA	NA
DMRcontig45935:6632580	6.6E+07	66326345	545	3	1.30E-06	35	6.422018349	NA	NA
DMRcontig45948:6634350	6.6E+07	66343700	200	2	0.000481801	4	2	NA	NA
DMRcontig46026:6644690	6.6E+07	66447300	400	1	2.12E-06	17	4.25	NA	NA
DMRcontig46074:6651140	6.7E+07	66511700	299	1	0.000944858	9	3.010033445	NA	NA
DMRcontig46082:6652160	6.7E+07	66521853	253	2	4.38E-06	17	6.719367589	NA	NA
DMRcontig46087:6652870	6.7E+07	66529100	400	1	0.000774168	8	2	NA	NA
DMRcontig46094:6653940	6.7E+07	66539780	380	2	5.67E-05	11	2.894736842	NA	NA
DMRcontig46106:6655550	6.7E+07	66555800	300	1	0.00088072	23	7.666666667	NA	NA
DMRcontig46156:6662322	6.7E+07	66623500	273	1	1.48E-05	15	5.494505495	NA	NA
DMRcontig46196:6667670	6.7E+07	66676800	100	1	0.000412969	5	5	NA	NA
DMRcontig46315:6683720	6.7E+07	66837300	100	1	0.000848154	1	1	NA	NA
DMRcontig46318:6684121	6.7E+07	66841559	350	2	0.000159958	13	3.714285714	NA	NA
DMRcontig46341:6687170	6.7E+07	66872000	300	2	4.55E-08	28	9.333333333	NA	NA
DMRcontig46344:6687553	6.7E+07	66875900	362	2	4.22E-05	17	4.696132597	NA	NA
DMRcontig46387:6693179	6.7E+07	66931962	168	1	0.00043456	3	1.785714286	NA	NA
DMRcontig46400:6694973	6.7E+07	66950000	268	1	0.000159516	12	4.47761194	NA	NA
DMRcontig46479:6705462	6.7E+07	67054800	174	1	0.000921062	7	4.022988506	NA	NA
DMRcontig46505:6708890	6.7E+07	67089000	101	1	0.00017097	0	0	NA	NA
DMRcontig46538:6713074	6.7E+07	67131209	468	4	7.86E-05	30	6.41025641	NA	NA
DMRcontig46551:6714850	6.7E+07	67148700	200	1	0.000272738	7	3.5	NA	NA
DMRcontig46612:6723040	6.7E+07	67230654	254	1	0.000152394	11	4.330708661	NA	NA
DMRcontig46641:6726820	6.7E+07	67268600	400	1	0.000807638	10	2.5	NA	NA
DMRcontig46780:6744973	6.7E+07	67449900	164	1	5.93E-05	10	6.097560976	NA	NA
DMRcontig46902:6761040	6.8E+07	67610500	100	1	0.000145282	2	2	NA	NA
DMRcontig46912:6762362	6.8E+07	67623900	281	1	5.66E-05	7	2.491103203	NA	NA
DMRcontig46980:6771435	6.8E+07	67714586	232	1	0.000640363	7	3.017241379	NA	NA
DMRcontig47003:6774400	6.8E+07	67744441	441	1	9.95E-05	37	8.390022676	NA	NA
DMRcontig47019:6776496	6.8E+07	67765387	424	3	1.64E-05	12	2.830188679	NA	NA
DMRcontig47151:6793584	6.8E+07	67936100	260	2	7.79E-05	9	3.461538462	NA	NA
DMRcontig47312:6815180	6.8E+07	68152395	595	4	3.75E-06	45	7.56302521	NA	NA
DMRcontig47326:6817010	6.8E+07	68170400	300	1	0.000843485	16	5.333333333	NA	NA
DMRcontig47367:6822520	6.8E+07	68225500	300	1	0.000262203	11	3.666666667	NA	NA
DMRcontig47429:6830430	6.8E+07	68304500	200	2	0.00028539	8	4	NA	NA
DMRcontig47455:6833800	6.8E+07	68338200	196	1	3.79E-05	10	5.102040816	NA	NA
DMRcontig47482:6837433	6.8E+07	68374500	163	1	2.56E-05	1	0.613496933	NA	NA
DMRcontig47498:6839487	6.8E+07	68395024	148	1	0.000309989	1	0.675675676	NA	NA
DMRcontig47565:6848260	6.8E+07	68483000	400	1	0.000156944	13	3.25	NA	NA
DMRcontig47578:6850180	6.9E+07	68502400	600	1	0.000582047	16	2.666666667	NA	NA
DMRcontig47618:6855511	6.9E+07	68555480	370	2	0.000172981	12	3.243243243	NA	NA
DMRcontig47651:6859852	6.9E+07	68598900	372	1	0.000676502	18	4.838709677	NA	NA
DMRcontig47730:6870150	6.9E+07	68701700	200	1	0.00061639	1	0.5	NA	NA
DMRcontig47741:6871640	6.9E+07	68716700	300	1	0.000261023	16	5.333333333	NA	NA
DMRcontig47829:6882960	6.9E+07	68830200	600	1	0.000192987	5	0.833333333	NA	NA
DMRcontig47848:6885440	6.9E+07	68854871	471	3	8.37E-05	29	6.157112527	NA	NA
DMRcontig47852:6885934	6.9E+07	68859600	253	1	0.000502977	6	2.371541502	NA	NA
DMRcontig47977:6902360	6.9E+07	69023800	192	2	0.00039919	0	0	NA	NA
DMRcontig48267:6940030	6.9E+07	69400450	150	1	5.89E-07	2	1.333333333	NA	NA
DMRcontig48335:6948830	6.9E+07	69488500	200	1	0.00089419	1	0.5	NA	NA
DMRcontig48430:6961345	7E+07	69613700	248	2	9.09E-05	9	3.629032258	NA	NA
DMRcontig48449:6963816	7E+07	69638573	413	1	0.000522026	32	7.748184019	NA	NA
DMRcontig48473:6966837	7E+07	69668600	231	1	4.78E-05	11	4.761904762	NA	NA
DMRcontig48480:6967790	7E+07	69678100	200	1	0.000942841	3	1.5	NA	NA
DMRcontig48615:6985340	7E+07	69853660	260	1	0.0005476	12	4.615384615	NA	NA
DMRcontig48674:6992910	7E+07	69929294	194	1	0.000238049	8	4.12371134	NA	NA
DMRcontig48774:7005940	7E+07	70059800	400	1	0.000288304	19	4.75	NA	NA
DMRcontig48805:7009990	7E+07	70100098	198	1	0.000578778	8	4.04040404	NA	NA
DMRcontig48837:7014070	7E+07	70140900	200	2	0.000175016	8	4	NA	NA

DMRcontig48895:7021370	7E+07	70214000	300	2	8.11E-05	14	4.666666667	NA	NA
DMRcontig48900:7022020	7E+07	70220539	339	2	0.000209378	3	0.884955752	NA	NA
DMRcontig48985:7032880	7E+07	70329100	300	2	6.09E-06	12	4	NA	NA
DMRcontig48987:7033130	7E+07	70331580	280	1	0.000747738	14	5	LOC101740724	Unknown
DMRcontig49113:7049040	7E+07	70490700	300	2	0.000144113	22	7.333333333	NA	NA
DMRcontig49139:7052406	7.1E+07	70524183	124	1	0.000484532	4	3.225806452	NA	NA
DMRcontig49171:7056290	7.1E+07	70563000	100	1	0.000746335	1	1	NA	NA
DMRcontig49311:7074291	7.1E+07	70743049	140	1	0.000914463	8	5.714285714	NA	NA
DMRcontig49346:7078740	7.1E+07	70787600	192	2	8.84E-05	13	6.770833333	NA	NA
DMRcontig49448:7091640	7.1E+07	70916700	300	1	0.0004249	14	4.666666667	NA	NA
DMRcontig49553:7105135	7.1E+07	71051600	251	1	0.000595937	4	1.593625498	NA	NA
DMRcontig49674:7120160	7.1E+07	71201900	300	1	0.000252805	20	6.666666667	NA	NA
DMRcontig49746:7129030	7.1E+07	71290830	530	5	3.09E-08	40	7.547169811	NA	NA
DMRcontig49786:7133873	7.1E+07	71339200	465	1	0.000432604	17	3.655913978	NA	NA
DMRcontig49844:7141130	7.1E+07	71411900	600	6	6.37E-06	27	4.5	NA	NA
DMRcontig49909:7149333	7.1E+07	71494000	665	2	0.00014268	34	5.112781955	NA	NA
DMRcontig50038:7165410	7.2E+07	71654300	200	1	3.14E-05	5	2.5	NA	NA
DMRcontig50043:7166041	7.2E+07	71660670	259	1	0.00045173	1	0.386100386	NA	NA
DMRcontig50050:7166816	7.2E+07	71668387	219	1	0.000162428	5	2.283105023	NA	NA
DMRcontig50230:7189220	7.2E+07	71892400	200	1	0.000344692	11	5.5	LOC101407462;MRPS12	Translation
DMRcontig50303:7198180	7.2E+07	71982778	978	1	0.000357252	68	6.952965235	NA	NA
DMRcontig50384:7208860	7.2E+07	72088800	200	1	0.000108011	9	4.5	NA	NA
DMRcontig50417:7212950	7.2E+07	72129853	353	1	0.000934069	13	3.682719547	NA	NA
DMRcontig50459:7218330	7.2E+07	72183800	500	1	0.000770595	30	6	NA	NA
DMRcontig50492:7222810	7.2E+07	72228192	92	1	0.000967684	1	1.086956522	NA	NA
DMRcontig50499:7223625	7.2E+07	72236384	131	1	0.000333119	0	0	NA	NA
DMRcontig50569:7232579	7.2E+07	72326062	269	3	2.47E-11	6	2.230483271	NA	NA
DMRcontig50621:7239381	7.2E+07	72394200	386	1	0.000295959	39	10.10362694	NA	NA
DMRcontig50719:7251721	7.3E+07	72517600	383	2	0.000103234	23	6.005221932	NA	NA
DMRcontig50788:7260992	7.3E+07	72610100	180	1	0.000223141	16	8.888888889	NA	NA
DMRcontig50798:7262330	7.3E+07	72623500	200	1	0.00051954	8	4	NA	NA
DMRcontig50824:7265660	7.3E+07	72657178	578	2	2.20E-05	30	5.190311419	NA	NA
DMRcontig50826:7265960	7.3E+07	72659800	200	1	0.000932818	15	7.5	NA	NA
DMRcontig51138:7305790	7.3E+07	73058300	400	1	0.000980557	22	5.5	NA	NA
DMRcontig51151:7307586	7.3E+07	73076200	337	2	2.10E-09	12	3.560830861	NA	NA
DMRcontig51153:7307878	7.3E+07	73079000	216	1	0.00068207	4	1.851851852	NA	NA
DMRcontig51159:7308650	7.3E+07	73086668	168	2	1.37E-25	2	1.19047619	NA	NA
DMRcontig51248:7319500	7.3E+07	73195085	85	1	0.000385583	6	7.058823529	NA	NA
DMRcontig51282:7323610	7.3E+07	73236311	211	2	4.56E-06	6	2.843601896	NA	NA
DMRcontig51292:7324913	7.3E+07	73249700	568	3	0.000157481	31	5.457746479	NA	NA
DMRcontig51353:7332645	7.3E+07	73326557	105	2	5.67E-06	4	3.80952381	NA	NA
DMRcontig51372:7334940	7.3E+07	73349600	200	1	0.000795825	2	1	NA	NA
DMRcontig51376:7335450	7.3E+07	73354700	200	1	0.000962319	6	3	NA	NA
DMRcontig51412:7339960	7.3E+07	73399800	200	1	3.46E-05	2	1	NA	NA
DMRcontig51469:7347630	7.3E+07	73476600	300	2	1.53E-08	9	3	NA	NA
DMRcontig51496:7351190	7.4E+07	73512100	200	1	0.000876294	6	3	NA	NA
DMRcontig51522:7354510	7.4E+07	73545300	200	1	0.000623636	7	3.5	NA	NA
DMRcontig51620:7367180	7.4E+07	73672100	300	1	0.000822722	17	5.666666667	NA	NA
DMRcontig51635:7369170	7.4E+07	73692100	400	1	0.000122794	5	1.25	NA	NA
DMRcontig51642:7370200	7.4E+07	73702400	400	1	6.40E-05	24	6	NA	NA
DMRcontig51684:7375490	7.4E+07	73755100	200	1	0.000154891	8	4	NA	NA
DMRcontig51734:7381710	7.4E+07	73817300	200	1	0.000144107	7	3.5	NA	NA
